# Methyltransferase DnmA is responsible for genome-wide N6-methyladenosine modifications at non-palindromic recognition sites in *Bacillus subtilis*

**DOI:** 10.1093/nar/gkaa266

**Published:** 2020-04-23

**Authors:** Taylor M Nye, Lieke A van Gijtenbeek, Amanda G Stevens, Jeremy W Schroeder, Justin R Randall, Lindsay A Matthews, Lyle A Simmons

**Affiliations:** Department of Molecular, Cellular, and Developmental Biology University of Michigan, Ann Arbor, MI 48109-1055, USA

## Abstract

The genomes of organisms from all three domains of life harbor endogenous base modifications in the form of DNA methylation. In bacterial genomes, methylation occurs on adenosine and cytidine residues to include N6-methyladenine (m6A), 5-methylcytosine (m5C), and N4-methylcytosine (m4C). Bacterial DNA methylation has been well characterized in the context of restriction-modification (RM) systems, where methylation regulates DNA incision by the cognate restriction endonuclease. Relative to RM systems less is known about how m6A contributes to the epigenetic regulation of cellular functions in Gram-positive bacteria. Here, we characterize site-specific m6A modifications in the non-palindromic sequence GACG^m^AG within the genomes of *Bacillus subtilis* strains. We demonstrate that the *yeeA* gene is a methyltransferase responsible for the presence of m6A modifications. We show that methylation from YeeA does not function to limit DNA uptake during natural transformation. Instead, we identify a subset of promoters that contain the methylation consensus sequence and show that loss of methylation within promoter regions causes a decrease in reporter expression. Further, we identify a transcriptional repressor that preferentially binds an unmethylated promoter used in the reporter assays. With these results we suggest that m6A modifications in *B. subtilis* function to promote gene expression.

## INTRODUCTION

DNA methylation is pervasive across all three domains of life. In eukaryotes, 5-methylcytosine (m5C) modifications have been shown to function in development and the regulation of gene expression, with aberrant methylation implicated in human health, including cancer, autoimmune diseases, and metabolic disorders [for review, (1,2)]. m5C in promoter regions has been linked to the repression of downstream gene transcription, whereas gene body methylation has been positively correlated with gene expression [for review (3)]. A lesser-studied modification in the genomes of eukaryotes is N6-methyladenine (m6A). Recent studies have identified m6A in the genomes of *Chlamydomonas*, *Caenorhabditis elegans* and *Drosophila melanogaster* (4–6). In contrast to promoter m5C, m6A modifications appear to function in gene activation in the algae *Chlamydomonas* (4) and promoter m6A is also important in early *Drosophila* development (5). Further, m6A was positively correlated with gene expression in a diverse set of fungi (7). Thus, there is a growing recognition that m6A is critical for the regulation of gene expression in a broad range of eukaryotic organisms.

Bacterial genomes are known to harbor N4-methylcytosine (m4C) in addition to m5C and m6A [(8) and references therein]. All three modifications impart consequences to bacterial cells when methylation is lost (9). The most well understood example of DNA methylation in eubacteria is in the context of restriction-modification (RM) systems [for review (10,11)]. RM systems function as a bacterial host defense mechanism to prevent the invasion of foreign DNA, including phages and other mobile genetic elements (10,11). In organisms with RM systems, unmethylated foreign DNA is targeted for site-specific cleavage by a restriction endonuclease while the host chromosome is protected at the recognition sequence by site-specific DNA methylation (12). Methylation is achieved through the activity of DNA methyltransferases (MTases). MTases catalyze the transfer of a methyl group from the donor *S*-adenosylmethionine (SAM) to adenosine or cytidine residues in DNA (13,14). MTases that lack a cognate endonuclease and do not function in RM systems are referred to as ‘orphan MTases’ (15). In a limited set of Gram-negative bacteria, orphan MTases have been shown to function in critical processes including cell cycle control (16), origin sequestration (17,18), DNA mismatch repair (19–21), and the regulation of gene expression [for review (22)]. DNA methylation from orphan and RM-based MTases has also been shown to establish epigenetic inheritance through phase variation primarily in Gram-negative pathogens (23–25). While much work has been done to characterize RM and orphan MTases from Gram-negative bacteria, much less is known about how m6A contributes to the regulation of the cell cycle or gene expression in Gram-positive bacteria (26).

Until recently, tools for unbiased detection and functional characterization of DNA methylation were limited. Available tools for detection, such as methylation-sensitive restriction endonuclease treatment and bisulfite sequencing, are limited to the sequence context and modification type that can be detected (27). The recent development of the Pacific Biosciences (PacBio) Single Molecule, Real-Time (SMRT) sequencing platform allows for detection of modifications without *a priori* knowledge of their existence (28). SMRT sequencing enables the analysis of real-time DNA polymerase kinetics for inference of DNA base modifications. Base modifications in the template strand result in changes in DNA polymerase kinetics compared to their unmodified counterparts, allowing for reliable, sequence-context specific detection of methylated bases during sequencing reactions (29). While differences in kinetic signatures for m5C modified cytidine residues are modest, SMRT sequencing is adept for m6A and m4C detection (29).

Using the SMRT sequencing platform, a recent study of 230 diverse prokaryotes detected base modifications in 93% of the genomes surveyed (8). Of the genomes with detected modifications, 75% of the modifications were m6A, which is due in part to the robust signal of m6A modifications in SMRT sequencing relative to other modifications (29). Given the high percentage of prokaryotic genomes with m6A detected and the contribution of m6A to the regulation of eukaryotic gene expression, it seems unlikely that the prevalent m6A modifications in prokaryotes are used exclusively in the context of regulating DNA cleavage by RM systems. As mentioned above, in *Escherichia coli* and *Caulobacter crescentus* m6A from orphan MTases occurs in palindromic recognition sequences and has been shown to mediate protein-DNA interactions (9,30), regulating important cellular processes including gene expression (31–34). Deletion of Dam methyltransferase (*dam*), which is responsible for m6A at GATC sites in *E. coli*, has severe pleiotropic effects (35,36). In *C. crescentus* deletion of the CcrM methyltransferase, which catalyzes the formation of m6A at GA(N)TC sites, is lethal when the CcrM-deficient strain is grown in rich media (16,37).

Much less is known about how m6A regulates cellular functions in Gram-positive bacteria. Recent work in *Streptococcus pyogenes* found that m6A from an active Type I RM system regulates virulence gene expression in a clinical isolate, suggesting that m6A could have important roles for regulating gene expression in Gram-positive systems (26).Therefore, the importance of m6A in *E. coli* and *C. crescentus* and the pervasive occurrence of m6A in prokaryotes (8) highlights the importance of understanding how m6A regulates cellular functions in the numerous and diverse set of bacterial genomes that contain the modification.

Here, we characterize m6A modifications in the Gram-positive bacterium *Bacillus subtilis* strains PY79 and NCIB 3610. Using SMRT sequencing, we show that m6A is present at non-palindromic GACG**^m^**AG sites throughout the *B. subtilis* chromosome. Further, we characterize the methyltransferase, referred to herein as DnmA, as responsible for detectable m6A modifications in the *B. subtilis* genome of both strains. We found that DnmA does not function as part of an active, canonical Type I or Type II RM system. Moreover, we show that the promoter regions for a subset of genes contain the consensus sequence and that loss of methylation in these *cis* regulatory elements results in a decrease in gene expression. Further, we show that the transcriptional repressor ScoC preferentially binds a promoter region that is unmethylated. Together, our results show that m6A can function as an epigenetic signal in *B. subtilis*.

## MATERIALS AND METHODS

### General bacteriology

The antibiotic concentrations used in this study are as follows: 5 μg/ml chloramphenicol, 0.5 μg/ml erythromycin, 100 μg/ml spectinomycin. Unless otherwise indicated, strains were grown in either LB (10 g/l tryptone, 5 g/l yeast extract, 10 g/l NaCl) or defined S7_50_ minimal media supplemented with 1% glucose (1× S7_50_ salts diluted from 10× S7_50_ salts (104.7 g/l MOPS, 13.2 g/l ammonium sulfate, 6.8 g/l monobasic potassium phosphate, adjusted to pH 7 with potassium hydroxide), 0.1% potassium glutamate, 1% glucose, 40 μg/ml phenylalanine, 40 μg/ml tryptophan, 2 mM MgCl_2_, 0.7 mM CaCl_2_, 50 μM MnCl_2_, 1 μM ZnCl_2_, 1 μg/ml thiamine-HCl, 20 μM HCl, and 5 μM FeCl_3_) at 30°C with shaking at 200 rpm.

### Strain construction

The strains, plasmids and oligos used in this study can be found in Supplementary Tables S1–S3. Individual strain and plasmid construction can also be found in the Supplemental Materials and Methods. Deletions were created by ordering *Bacillus subtilis* 168 strains from the Bacillus Genetic Stock Center (http://www.bgsc.org/) where the respective genes were replaced with a *loxP* flanked erythromycin (erm) resistance cassette (BKE strains). Genomic DNA from the BKE strains was purified and used to transform *B. subtilis* strain PY79, and the erm resistance cassette was subsequently removed with Cre recombinase (38). Overexpression strains and all promoter GFP fusions were integrated in the PY79 *amyE* locus via double crossover (39). Three colonies containing the crossover were selected and colony purified on LB plates containing 100 μg/ml spectinomycin. Successful integration of the constructs was verified by PCR, Sanger sequencing, and screening for the ability to utilize starch.

### Chromosomal DNA purification

Genomic DNA for Pacific Biosciences SMRT sequencing was purified as follows. Strains were struck out on LB and grown overnight at 30°C. 500 ml LB cultures were inoculated at OD_600_ 0.05 and grown at 37°C. During mid-exponential phase (OD_600_ 0.6–0.8) an equal volume of methanol was added to each culture and centrifuged at 4000 rpm for 30 min. The supernatant was discarded and cells were resuspended in 12.5 ml of 10% sucrose Tris/HCl pH 8 buffer and transferred to Oakridge tubes. Resuspensions were then treated with 310 μl lysozyme (40 mg/ml in 10% sucrose Tris/HCl pH 8 buffer) for 30 min at 37°C and mixed every 5 min. 1.25 ml of 0.5 M EDTA was added to each tube and incubated on ice for five minutes followed by addition of 10 ml of freshly prepared lysis solution (0.1% Triton X-100, 62.5 mM EDTA, 50 mM Tris/HCl pH 8). Solutions were centrifuged at 15 000 rpm for 30 min and decanted into chilled graduated cylinders. To each lysate 0.95 g/ml of cesium chloride (CsCl) was added and dissolved followed by a 1/10 volume addition of 10 mg/ml ethidium bromide. Solutions were balanced and centrifuged at 44 000 (131 600 × g) rpm for 24 h. Chromosomal DNA was extracted and subjected to a second round of CsCl purification as described above. Solutions were centrifuged at 44 000 rpm (131 600 × g) for 48 h. Ethidium bromide was removed by extraction 4× with water-saturated butanol. The aqueous phase was transferred to an Oakridge tube and 1 volume of water and 2 volumes ethanol were then added. The solution was centrifuged at 15 000 rpm for 20 min and the supernatant was aspirated. The pellet was washed with 70% ethanol and resuspended in 1 ml TE buffer.

In all other experiments, frozen strains were struck out and grown at 30°C. The plates were washed in S7_50_ minimal media and 25 ml cultures were inoculated at an OD_600_ 0.05 and grown at 37°C with shaking to mid-exponential growth phase (OD_600_ 0.6–0.8). Genomic DNA was purified via phenol chloroform extraction method.

### PacBio SMRT sequencing and methylation analysis

Chromosomal DNA was prepared for sequencing as described above. Library preparation and subsequent sequencing was performed as previously described (40,41). Modification and motif analyses were performed using RS_Modification_and_Motif_Analysis.1 version 2.3.0 with the appropriate *B. subtilis* reference genomes. The initial parameters used for modification analysis were performed using 0.75 minimum high quality reads, 50 bps minimum length, and a minimum ModQV call of 30. We also increased minimum high quality reads to >0.85 and minimum length to >1000 bp in subsequent analysis. Modification graphs were generated using functions from BaseModFunctions.v2.1.R available at: https://github.com/PacificBiosciences/Bioinformatics-Training/tree/master/basemods.

### Motif distribution analysis

Motif distribution analysis was performed using the DistAMo web based server (42) available at http://computational.bio.uni-giessen.de/distamo searching the GACGAG motif for the *B. subtilis* PY79 genome via accession number NC_022898.1.

### Protein purification (DnmA, DnmA (Y645A) and YabB)

Recombinant proteins were purified from *E. coli* BL21_DE3_ cells containing a pE-SUMO vector with the *B. subtilis* gene inserted (*dnmA, dnmA (Y465A)* or *yabB*). Cultures were grown in 4 l of terrific broth (2.4% yeast extract, 1.2% tryptone, 0.4% glycerol, 250 mM (NH_4_)_2_SO_4_, 500 mM KH_2_PO_4_, 1× metals (1000× metals: 2.5 mM FeCl_3_, 1 mM CaCl_2_, 0.5 mM ZnCl_2_, 0.1 mM CoCl_2_, 0.1 mM CuCl_2_, 0.1 mM NiCl_2_, 0.1 mM Na_2_MoO_4_, 0.1 mM Na_2_SeO_3_, 1 mM H_3_BO_3_) and 25 μg/ml kanamycin) at 37°C with orbital rotation for 2 h until reaching an OD_600_ of ∼0.7. Overexpression was induced by adding IPTG to 1 mM and the cultures were grown for three additional hours at 37°C. Cells were then pelleted by centrifugation and frozen in liquid nitrogen to be stored at −80°C. Once thawed, the pellet was resuspended in lysis buffer (50 mM Tris–HCl pH 8, 300 mM NaCl, 10% sucrose, 10 mM imidazole, 1× protease inhibitors (Roche 11873580001)) and cells were sonicated on ice. Cell debris was pelleted by centrifugation. Supernatant was then poured through a 3 ml Ni^2+^-NTA agarose gravity-flow column. The column was washed with wash buffer (20 mM Tris–HCl pH 8, 10% glycerol, 20 mM imidazole, 2 M NaCl) and eluted with elution buffer (50 mM Tris–HCl pH 8, 150 mM NaCl, 400 mM imidazole). SDS-PAGE was performed to confirm the presence of desired protein. The sample was then dialyzed into anion exchange start buffer (50 mM Tris–HCl pH 8, 25 mM NaCl, 5% glycerol, 1 mM β-mercaptoethanol) and the sample was applied to a Q column (GE: 17115301) using an elution gradient of 50–750 mM NaCl. SDS-PAGE was performed and fractions containing desired protein were pooled and incubated with ULP1 protease at 25°C for 30 min. The digestion product was applied to another 3 ml Ni^2+^-NTA gravity-flow column, washed, and eluted using the same buffers as above. SDS-PAGE was again performed to confirm the SUMO tag was removed and the protein was concentrated and buffer exchanged into protein storage buffer (50 mM Tris–HCl pH 8, 150 mM NaCl, 50% glycerol), aliquoted, flash frozen in liquid nitrogen and stored at −80°C.

### ScoC purification

Primers oTMN62 and 63 were used to amplify *scoC* from the *B. subtilis* chromosome and were subsequently combined with the pE-SUMO expression vector via Gibson assembly. Recombinant proteins were purified from *E. coli* BL21_DE3_ cells grown in 2 l of LB with 25 μg/ml kanamycin at 37°C with orbital rotation until an OD_600_ of 0.7 was reached. Overexpression was induced by adding 0.5 mM IPTG followed by culture growth for an additional three hours at 37°C with orbital rotation and cultures were subsequently pelleted via centrifugation and stored at −80°C. The pellet was re-suspended in lysis buffer and sonicated on ice as described for DnmA and YabB. Subsequent to centrifugation, the supernatant was applied to a 4 ml Ni^2+^-NTA agarose gravity-flow column. The column was washed with wash buffer (50 mM Tris–HCl pH 8, 25 mM imidazole, 2 M NaCl, 5% glycerol) and eluted with elution buffer (50 mM Tris–HCl pH 8, 400 mM imidazole, 150 mM NaCl, 5% glycerol). Following elution, 1 mM DTT and SUMO ULP1 protease were added to the elution fraction and incubated for 2 h at room temperature. The sample was then dialyzed into storage buffer (50 mM Tris–HCl pH 8, 150 mM NaCl, 5% glycerol) overnight at 4°C. The dialyzed sample was then applied to another 4 ml Ni^2+^-NTA gravity-flow column to separate the recombinant protein from the SUMO tag. SDS-PAGE was performed to confirm the SUMO tag was removed. Glycerol was added to 25% and the protein was aliquoted and flash frozen for storage at −80°C

### Methylation assays

All methylation reactions were performed in a buffer containing 50 mM Tris–HCl pH 8, 50 mM NaCl and 200 μM MgSO_4_. The following substrates were annealed in the same buffer at 2.5 μM concentration by heating primers to 100°C for 30 s and then cooling to room temperature on the bench top: dsDNA target (oTNM38, oTMN39); dsDNA non-target (oTMN40, oTMN41); and dsRNA (oJR270, oJR271). The H^3^-SAM (Perkin Elmer: NET155H001MC) was used at a concentration of 1 μM in solution. The purified DnmA, YabB, or DnmA (Y465A) was added to a concentration of 1 μM and all substrates were used at 0.25 μM in solution. The proteins were added in excess to determine if there was any off target methylation activity at higher protein concentrations. The total reaction solution came to 10 μl. All reactions were incubated at 37°C for 150 min unless otherwise specified. Reactions were stopped using 450 μl of 10% TCA and placed on ice. The samples were filtrated using Glass microfiber filters (GE: 1822-025), washed with cold 70% ethanol, dried, and placed in a scintillation counter to measure mmol incorporation.

### Growth curves

Strains were plated on LB and grown overnight at 30°C. Plates were washed in LB and diluted to an OD_600_ of 0.05 in 10 ml of LB in side-armed flasks. Cultures were grown in shaking water baths at 37°C and optical density was measured using a Klett meter every half hour through late stationary phase. Growth curve experiments were done in triplicate and data was subsequently fit to a Gompertz growth (43) model {$y\ = \ Aexp\{ { - \exp [ {\frac{{{\mu _{m\ \times \ e}}}}{A}\ ( {\lambda - t} ) + 1} ]} \}$ (where the parameters A, *μ_m_*, and *λ* represent the time (*t*) when the growth rate equals zero (asymptote), the maximum growth rate, and the lag time, respectively), to obtain growth rate estimates (*μ_m_*) for each strain.

### Transformation efficiency assays

Strains were plated on LB and grown overnight at 30°C. Plates were washed with phosphate buffered saline (PBS) pH 7.4 and the cells were pelleted, the supernatant was aspirated, and a second PBS wash was completed before the cells were resuspended in PBS. The cells were used to inoculate a culture at an OD_600_ of 0.05 into 1 ml of 1× MC media (10× MC media: 615 mM K_2_HPO_4_, 380 mM KH_2_PO_4_, 1.11 M dextrose anhydrous, 30 mM sodium citrate dihydrate, 840 μM ferric ammonium citrate, 0.5 g casein hydrolysate, and 125 mM sodium aspartate monohydrate, to 50 ml with ddH_2_O and filter sterilize) with 3 μl of 1M MgSO_4_ and grown at 37°C with aeration for 4 h. After 4 h, 3 μl of 1M MgSO_4_ and 300 ng of pHP13 purified from *E. coli* MC1061 cells was added to 300 μl of cells and grown for an additional 1.5 h at 37°C. 10× serial dilutions were performed into PBS and appropriate dilutions were plated onto LB plates for colony forming unit (CFU) counts and chloramphenicol plates for transformation forming unit (TFU) counts. Transformation efficiencies were calculated as TFU/CFU and the average transformation efficiency for replicates performed over three separate days was plotted along with the corresponding standard errors.

### Flow cytometry

Cells were grown overnight at 30°C on LB plates containing 100 μg/ml spectinomycin. Exponentially growing colonies were washed from the plates using S7_50_ medium, and washed two more times to remove residual LB agar before diluting the cells in pre-warmed S7_50_ medium to an OD_600_ of 0.05. Cells were grown to an OD_600_ of 0.4 at 30°C after which fluorescence of 200 000 cells was measured using an Attune™ NxT Acoustic Focusing Cytometer (ThermoFisher Scientific) using the following settings: Flow rate, 25 μl/min; FSC voltage, 200; SSC voltage, 250; BL1 voltage, 250.

### Streptavidin pull-down

5′ biotinylated primers were used to amplify the 233 bp region of the *scpA* promoter via PCR using genomic DNA from strains LVG087 and LVG102 as a template, which correspond to the GACGAG and GACGTG promoter, respectively. To obtain total cell lysate, 4 l of strain TMN85 (Δ*dnmA*) was grown in S7_50_ medium at 37°C with shaking until the culture reached an OD_600_ of 1.0. After the cells were harvested the pellets were washed with 1× PBS (pH 7.5) and then subsequently washed with Pull- Down Binding Buffer (PDBB; 50 mM Tris–HCl pH 7.5, 0.5 mM EDTA, 100 mM NaCl, 0.01% (v/v) Triton X-100, 25% (v/v) glycerol and 1 mM DTT) and resuspended in ice-cold 20 mL PDBB supplemented with one tablet of cOmplete™, EDTA-free Protease Inhibitor Cocktail (Roche, Mannheim, Germany). The cell suspensions were sonicated on ice (10 s on, 40 s off, 70 Hz) until the solutions cleared. Cell debris was removed from the lysate by two subsequent washing steps and the protein content of the supernatant was estimated using a Bradford assay (∼20 mg/ml protein). For each pull-down experiment, 100 μl of Dynabeads™ M-270 Streptavidin magnetic bead slurry (ThermoFisher Scientific) was washed three times with 500 μl Pull-Down Wash Buffer (PDWB; 10 mM Tris–HCl pH 7.5, 1 mM EDTA, and 1 M NaCl). The beads were re-suspended in 250 μl PDWB, mixed with 200 pmol biotinylated probe DNA dissolved in 250 μl nuclease-free water, and incubated for 30 min at 25°C with gentle rotation. The DNA-coated beads were washed three times with PDBB before 100 mg protein and 100 μg salmon sperm DNA (Millipore Sigma) were mixed and added to the DNA-bound beads. After 2 h of incubation at room temperature with gentle rotation, the beads were separated and washed once with PDBB, once with PDBB plus 100 μg salmon sperm DNA, and again with PDBB. Bound proteins were eluted using Pull-Down Elution Buffer (PDEB; 50 mM Tris–HCl pH 7.5, 0.5 mM EDTA, 1 M NaCl, 0.01% (v/v) Triton X-100, 25% (v/v) glycerol and 1 mM DTT). The eluted proteins were desalted and concentrated using TCA precipitation and separated on a 4–20% Mini-PROTEAN TGX precast protein gel (Bio-Rad, Hercules, USA). Bands in the 20 and 40 kDa size range were excised from the gel followed by protein identification using mass spectrometry through the University of Michigan Proteomics Resource Facility, project PRF-2019-L-SIMM-29.

### ScoC EMSA

5′ IR dye end-labeled substrates oTN67/oTN68 and oTN70/oTN71, corresponding to the GACGAG and GACGTG oligos, respectively, were annealed at a concentration of 50 nM by heating at 95°C for 1 minute and then snap-cooled on ice. Care was taken to avoid subjecting the IR dye labeled oligos to light. Annealed oligos were mixed at a final concentration of 5 nM with indicated concentrations of purified ScoC in 1× EMSA reaction buffer (5× EMSA reaction buffer: 250 mM Tris×HCl pH 8, 5 mM EDTA, 150 mM KCl, 10 mM MgCl_2_, 5 mM DTT, 1% Tween 20, 125 μg/ml sheared salmon sperm DNA) to a final volume of 10 μl. Reactions were incubated at 37°C for 15 min and subsequently loaded onto and resolved via 6% Native-PAGE, which was performed covered and on ice for 60 min at 100 V. The samples were visualized with the LI-COR Odyssey imager. The intensity of the shifted band was normalized to the no protein control for each sample to calculate the percent band shifted. Three replicates were completed and quantified across separate days and the average and standard errors for percent band shifted was reported in Figure 6.

## RESULTS

### Characterization of *B. subtilis* PY79 and NCIB 3610 methylomes

It was previously published that *B. subtilis* does not have m6A at the *E. coli* Dam MTase recognition site, GATC, and that ectopic expression of Dam in *B. subtilis* induced the DNA damage response (44,45). However, until recently it remained unknown if *B. subtilis* contains m6A in another sequence context because the detection of m6A without *a priori* knowledge of the sequence context would require a new experimental approach. PacBio SMRT sequencing was used to determine if DNA modifications were present in the genome of several *B. subtilis* strains with the results deposited on the publicly available web resource REBASE maintained by New England Biolabs. This resource reports m6A occurring in various sequence motif contexts in 19 of 23 *B. subtilis* strains where SMRT sequencing was used. Among the *B. subtilis* strains analyzed, methylation at GACG^m^AG sites was reported in four of the 23 strains (http://rebase.neb.com). Previously, our group performed PacBio sequencing on the widely used *B. subtilis* laboratory strain PY79 for whole-genome assembly (41). As part of our effort to study DNA methyltransferases, we used PacBio sequencing to characterize the PY79 methylome. We purified genomic DNA from the wild type (WT) *B. subtilis* strain PY79 and analyzed our results using the SMRT sequencing platform, allowing for genome-wide base modification detection in sequence-specific contexts (29).

SMRT sequencing of the *B. subtilis* PY79 chromosome showed that the second adenosine residue within the sequence context 5′-GACG^m^AG showed high modification quality values (modQVs), which indicates a statistically significant difference in DNA polymerase kinetics from the expected background at particular loci (Supplementary Figure S1A, Table 1). The interpulse duration (IPD) ratios, which are a comparison of DNA polymerase kinetics at a base within a particular sequence context compared to an unmethylated *in silico* control, were far higher for the second adenosine residue in the GACG^m^AG motif compared to any other modified motifs in the *B. subtilis* chromosome (Table 1, Supplementary Figure S1A). Thus, we identify m6A in the sequence context 5′-GACG^m^AG in the chromosome of *B. subtilis* PY79, herein referred to as the m6A motif.

**Table 1.** Relevant modified motifs detected in *B. subtilis* by PacBio SMRT sequencing

Motif^a^	Type	%Detected	Mean QV	Mean Cov.	Mean IPD ratio
**WT PY79**					
GACGAG	m6A	99.7	388	286	6.72
CTCGARB	m5C^b^	70.8	74	270	1.89
**WT 3610**					
GACGAG	m6A	94.7	362	313	4.84

^a^All motif calls by SMRT sequencing are reported in Supplementary Table S4.

^b^Modification type confirmed via methylation sensitive restriction endonuclease digest as described in the supporting document.

We found that 99.7% of m6A motifs (1215/1219) were called as methylated in the PacBio SMRT sequencing analysis at the 3′-adenosine during exponential growth in defined minimal medium. While our sequencing analysis identified other motifs in the *B. subtilis* PY79 chromosome, the average modQVs, IPD ratios, and the percentage of motifs called as modified were far lower compared to m6A identified within the GACG^m^AG sequence (Supplementary Table S4 and Figure S2). It is likely that most of the other motifs called represent DNA secondary structures that affect DNA polymerase kinetics or sequencing noise instead of genuine nucleic acid modifications (Supplementary Table S4). For completeness we chose to report all motifs called during analysis of the SMRT sequencing data (Supplementary Table S4).

Of the 1219 m6A motifs that occur in the *B. subtilis* PY79 genome, 1118 (91.7%) occur in protein coding regions. Intergenic regions, which compose 11.2% of the genome, contain 7% (85 motifs in 76 regions) of the m6A motifs. With the exception of only a few sites, the majority of m6A sites had >75% of sequencing reads called as methylated independent of genome position or occurrence on the plus or minus strand of the chromosome (Supplementary Figure S2 and Supplementary Table S4).

*B. subtilis* PY79 is a commonly used laboratory strain, however selection in the lab has caused PY79 to lose many of the robust phenotypes associated with ancestral strains of *B. subtilis* (46). To determine whether m6A is present in the ancestral strain, we purified genomic DNA from *B. subtilis* strain NCIB 3610 (40) for SMRT sequencing and found m6A within the same GACGAG sequence context (Supplementary Figure S1B and Table 1). In NCIB 3610 94.7% (1208/1275) of m6A sites were called as methylated in the PacBio SMRT sequencing analysis. The chromosome of the ancestral strain is considerably larger than PY79 and harbors an 84-kb plasmid, both of which account for the increased number of m6A motifs (40). The decrease in the percentage of motifs called as modified between PY79 and NCIB 3610 (99.7% → 94.7%) could be the result of biological variation, such as an increase in protein binding or other factors that may occlude methylation of recognition sites. The decrease in motifs called could also be due to technical variation in sequencing reactions. We note that we also detected many additional motifs in the ancestral strain that did not appear in the lab strain PY79, with each motif called listed in supplementary Table S4. Further, m6A at GACGAG sequences has also been reported for three *B. subtilis* strains other than PY79 and NCIB 3610 on REBASE.

In addition to m6A modifications, SMRT sequencing of the PY79 genome identified cytidine modifications in the sequence ^m^CTCGARB (where R represents a purine and B either a cytidine or a guanosine). These results are described in the supplementary results section, where we show using methylation-sensitive restriction digest that m5C formation occurs in the *B. subtilis* PY79 genome through the BsuMI RM system **(**Supplementary Figure S3) previously described for *B. subtilis* Marburg (47).

### Distribution of m6A sites across the *B. subtilis* genome shows enrichment on the lagging strand of the left chromosomal arm

**Figure 1.**
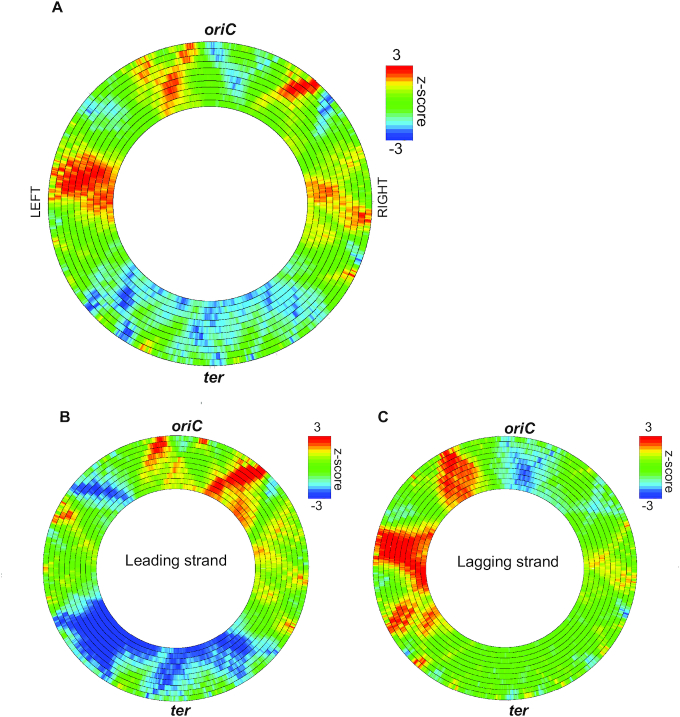
Motif enrichment analysis for m6A sites in the *B. subtilis* PY79 chromosome. Motif enrichment analysis was performed using the DistAMo web based server tool (42). Sliding windows of 50 kb to 500 kb are represented by the rings from outside (large) to inside (small) rings scaling in 50 kb increment increases. Over (red) and under (blue) enrichment are represented by z-scores in the scale indicated. (**A**) m6A motif enrichment for all motifs with *ori* and *ter* regions indicated; (**B**) m6A motif enrichment on the leading strand; (**C**) m6A motif enrichment on the lagging strand.

To begin to understand the function of m6A in *B. subtilis*, we used the motif enrichment program DistAMo (42) to determine the location of m6A sites on the *B. subtilis* chromosome. This was done to determine if m6A sites are uniform or showed areas of enrichment and de-enrichment throughout the chromosome (Figure 1). We present the analysis using sliding windows of 50 kb to 500 kb over the length of the chromosome by the rings from outside (large) to inside (small) scaling in 50 kb increments. Over (red) and under (blue) enrichment are colored by z-scores in the scale as shown. From the analysis we determine that the locations of m6A sites are certainly not uniform across the chromosome and instead show patterns of enrichment in particular regions. We find that several areas are largely devoid of m6A sites, including the terminus region and the origin of replication (Figure 1). Analysis of enrichment shows that locations in the *B. subtilis* chromosome with high z-scores includes the right and left chromosomal arms with the largest enrichment on the lagging strand of the left chromosomal arm (Figure 1C). With these results we suggest that m6A is unlikely to function in origin sequestration or DNA mismatch repair as described for Dam methylation in *E. coli* (17,18) due to our finding that the origin does not contain m6A sites and because m6A is non-palindromic and not uniform across the *B. subtilis* chromosome. To be certain, we empirically test if m6A contributes to replication timing, mutagenesis, or recombination in the supplementary results and show no effect (Supplementary Figures S4, S8 and Supplementary Table S5).

### Methyltransferase YeeA is necessary for m6A formation *in vivo*

DNA methylation is catalyzed by DNA methyltransferases (MTases) (48). To identify putative MTase(s) responsible for the observed m6A modification, we searched all protein coding sequences for the conserved DNA m6A MTase catalytic motif (D/N/S)PPY (48). This search yielded two uncharacterized MTases, coded for by the genes *yabB* and *yeeA* (*dnmA*) (41). We created clean deletions of the Δ*yabB* and Δ*yeeA* (*dnmA*) coding regions as well as a Δ*yabB*Δ*yeeA* double deletion. Each of these strains was viable and none of the deletions conferred a growth defect on *B. subtilis* under the conditions used here (Figure 3A, described later in the results).

To identify the MTase responsible for genomic m6A, DNA was harvested from each strain when cultures reached an OD_600_ of ∼0.7 followed by SMRT sequencing. Subsequent methylation analysis revealed that chromosomal DNA from Δ*yeeA* (*dnmA*) cells lost all detectable methylation at the m6A motif previously identified in WT cells in both PY79 and NCIB 3610 strain backgrounds (Table 2, Supplementary Figure S5, and Supplementary Tables S6, S7 and S8 for all PY79 Δ*yeeA* (*dnmA*) GACGAG sites). Expression of *yeeA* (*dnmA*) from an ectopic locus in the Δ*yeeA* (*dnmA*) background restored methylation at the m6A site (Supplementary Figure S5C and Table 2). Computational analysis from sequencing data posted on REBASE also predicted YeeA (DnmA) as the MTase responsible for m6A detected in strains of *B. subtilis* with modifications at the m6A motif described here.

**Table 2.** Relevant modified motifs detected in *B. subtilis* by PacBio SMRT sequencing

Motif^a^	Type	%Detected	Mean QV	Mean Cov.	Mean IPD ratio
**Δ*dnmA* WT PY79**					
CTCGARB	m5C^b^	46.7	51	120	2.00
**Δ*dnmA* WT 3610**					
None^c^				358	
**Δ*dnmA, amyE*::*Pspac dnmA* PY79**					
GACGAG	m6A	99.7	213	152	6.32
CTCGARB	m5C	52.7	59	149	2.00

^a^All motif calls by SMRT sequencing are reported in Supplementary Table S6.

^b^Modification type confirmed via methylation sensitive restriction endonuclease treatment as described in the supporting document.

^c^GACGAG and CTCGARB were not detected in NCIB 3610 Δ*dnmA*. All other motifs called are reported in Supplementary Table S6. The average coverage is reported for each spurious motif detected.

Genomic DNA from Δ*yabB* cells retained the methylation at m6A sites (Supplementary Figure S6, Supplementary Table S9) whereas detectable modifications at the m6A site were lost in the double deletion strain (Supplementary Figure S6B, Supplementary Table S9). Interestingly, while methylation is maintained at the m6A site in the Δ*yabB* strain, we noticed additional motifs not present in the WT or Δ*yeeA* (*dnmA*) strains that were detected upon loss of *yabB* in the single or double deletion strains (Supplementary Table S9). These additional motifs are likely to result from sequencing noise and/or DNA secondary structure given the low IPD ratios (Supplementary Table S9). With these results we show that *yeeA* (*dnmA*) is necessary for genomic m6A formation in the sequence context GACG^m^AG *in vivo* and we refer to YeeA herein as DNA methyltransferase A (DnmA), with the formal name of M.BsuPY79I and M.Bsu3610I for strains PY79 and NCIB 3610, respectively. For simplicity, we will collectively refer to M.BsuPY79I and M.Bsu3610I as DnmA in the work presented below.

### DnmA is sufficient for methylation of m6A sites in double stranded (ds)DNA *in vitro*

**Figure 2.**
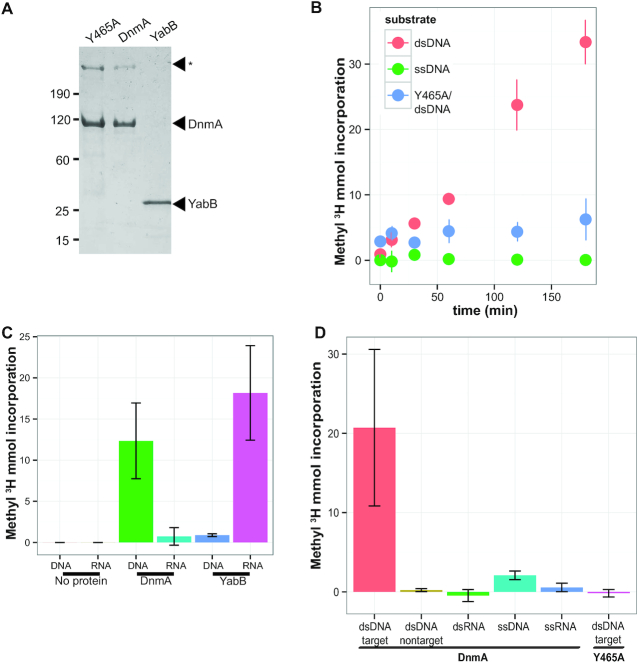
DnmA is sufficient for methylation of dsDNA at 5′GACGAG sites. (**A**) SDS-polyacrylamide gel of purified catalytically inactive DnmA variant Y465A, WT DnmA (M.BsuPY79I), and YabB. (*) indicates DnmA multimer. (**B**) DnmA (M.BsuPY79I) incorporation of tritiated SAM into dsDNA and ssDNA substrates carrying the GACGAG sequence over time. Y465A (indicated in blue) is a DnmA (M.BsuPY79I) catalytically inactive variant. (**C**) Incorporation of tritiated SAM into DNA and RNA substrates by MTases DnmA (M.BsuPY79I) and YabB. (**D**) DnmA (M.BsuPY79I) incorporation of tritiated SAM onto indicated substrates. The DnmA catalytically inactive variant is indicated.

DNA MTases typically use SAM to catalyze the transfer of a methyl group to a DNA base (9). DnmA (M.BsuPY79I), YabB, and a DnmA catalytically inactive variant (Y465A) were purified (Figure 2A). In addition to the predicted ∼120-kDa band corresponding to the DnmA monomer, a high molecular weight species was observed in the DnmA purifications. The slower migrating protein was analyzed by mass spectrometry identifying it as multimer of DnmA. We speculate that the DnmA multimer is caused by irreversible disulfide bonding or another crosslink that forms between two purified DnmA monomers during isolation (Supplementary Table S10).

A time course methylation experiment was performed to determine if DnmA is sufficient to catalyze methylation of the m6A motif in DNA (Figure 2B). The purified proteins were incubated with tritiated SAM and an oligonucleotide sequence from the *B. subtilis addA* locus containing the m6A (target) motif. Incorporation of the labeled methyl group over time indicates that DnmA is indeed sufficient for methylation at m6A motifs in dsDNA (Figure 2B). With the results from the time course methylation experiment we suggest that purified DnmA does not have significant activity on single-strand (ss)DNA. As a control we show that the Y465A catalytically inactive variant was unable to methylate the substrate indicating that the MTase activity we detect is specific to DnmA.

With the *in vitro* methylation assay established, we tested the activity of DnmA and YabB on DNA containing the target sequence and whole cell RNA extracted from a Δ*dnmA*Δ*yabB* double mutant strain by assaying for incorporation of methylation from tritiated SAM. As expected, DnmA showed activity on the dsDNA substrate with the target sequence, but had minimal activity when whole cell RNA was used as a substrate (Figure 2C). In support of the *in vivo* results, we show that purified YabB had very little activity on a DNA substrate, whereas YabB did show incorporation when whole cell RNA was used as a substrate. With these results we suggest that YabB may function as an RNA methyltransferase (Figure 2C). To test if the m6A motif was necessary for DnmA methylation *in vitro*, the 3′-adenosine residue was substituted with thymidine (non-target sequence) and incubated with DnmA and tritiated SAM. As shown in Figure 2D, there was no appreciable incorporation of the methyl group by DnmA to the non-target sequence, demonstrating that methylation is specific for the target sequence (m6A motif). We also tested DnmA for methylation of dsRNA, ssDNA and ssRNA bearing the target sequence. The results show little to no methylation for any of these substrates with the exception of ssDNA, which yielded only weak methylation activity relative to dsDNA (Figure 2D). Together, these results provide strong evidence that DnmA is specific for dsDNA containing the m6A motif.

To determine if the lack of methylation at the non-target sequence was caused by an inability of DnmA to bind DNA, an electrophoretic mobility shift assay (EMSA) was performed on 5′ end-labeled target (GACGAG), non-target (GACGTG), and a degenerate sequence where the entire target sequence had been removed. Incubation of DnmA with the target, non-target, and degenerate sequences each resulted in a shift, indicating that the methylation specificity is not due to a loss of DNA binding at other sequences (Supplementary Figure S7). Additionally, the Y465A catalytically inactive variant still bound the target sequence, suggesting that this variant is only dysfunctional for methyltransferase activity (Supplementary Figure S7). We conclude that DnmA is necessary and sufficient to methylate dsDNA that carries the GACGAG sequence *in vivo* and *in vitro* and that Y465 is an important residue for activity.

### DnmA does not function as part of an active RM system

**Figure 3.**
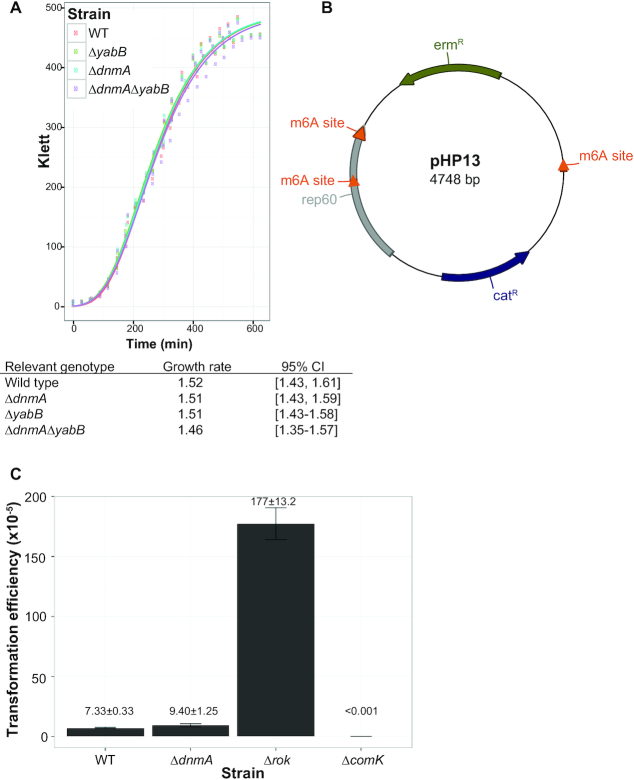
Loss of DnmA does not affect growth rate or transformation efficiency of foreign methylated DNA. (**A**) Growth curves for WT, Δ*dnmA*, Δ*yabB*, and Δ*dnmA*Δ*yabB* were performed in triplicate and fit to a Gompertz growth model (43) to calculate growth rate. Growth rate and the corresponding 95% confidence interval for each strain are indicated. (**B**) Plasmid map of pHP13 with the location of each m6A site shown. The orange carrots indicate the relative position and strand orientation for each site. (**C**) Transformation efficiency assays were performed using pHP13 plasmid purified from *E. coli* as donor DNA in WT, Δ*dnmA*, Δ*rok*, and Δ*comK* recipient strains. The average transformation efficiency and standard error for each strain is indicated.

We next asked if DnmA functions as part of an active RM system. DnmA shares 38% identity and 57% similarity with the MmeI enzyme, which is a bifunctional protein with a methyltransferase domain and a PD-ExK endonuclease motif in the amino terminal domain. MmeI belongs to a subgroup of Type II RM systems that use DNA hemi-methylation for host chromosome protection (49). DnmA was included in a set of MmeI homologs that lack the endonuclease motif in the amino terminal domain but are flanked by conserved genes similar to *yeeB* and *yeeC*, which are immediately downstream of *dnmA* (49). It was found that under the conditions tested for other MmeI homologs DnmA lacked endonuclease activity, however it is important to note that the downstream *yeeB* and *yeeC* gene products are annotated as a putative helicase and an endonuclease, respectively (49). Deletion of *dnmA* does not result in a growth defect (Figure 3A), which would suggest that *yeeB* or *yeeC* lacks endonuclease activity associated with typical Type II RM systems, where endonuclease activity is achieved independent of the MTase.

It has been suggested that DnmA, along with YeeB and YeeC, comprise a Type I-like RM system, where restriction endonuclease activity requires the MTase subunit and DNA cleavage would not occur efficiently in the absence of DnmA (49). To test this possibility, we performed a transformation efficiency assay in WT and Δ*dnmA* cells with the plasmid pHP13, which is a 4.7 kb plasmid containing three m6A sites as the donor DNA (Figure 3B). Plasmid purified from *E. coli* cells was used to transform competency deficient (Δ*comK*), hyper-competent (Δ*rok*), WT and Δ*dnmA* strains followed by selection for transformants conferring resistance to chloramphenicol. We found that compared to Δ*comK* and Δ*rok* strains, with transformation efficiencies of < 1 × 10^−8^ and 177 × 10^−5^ (SE 13.2 × 10^−5^), respectively, the transformation efficiencies of WT [7.33 × 10^−5^ (SE 3.30 × 10^−6^)] and Δ*dnmA* [9.44 × 10^−5^ (SE 1.25 × 10^−5^)] were nearly indistinguishable (Figure 3C). We show that DnmA, YeeB and YeeC do not function to restrict DNA update during natural genetic competence. Based on the transformation results and the conservation of these three genes clustering together, we suggest that DnmA, YeeB and YeeC could be part of an inactive or inefficient Type I-like RM system or perhaps a noncanonical RM system. We also cannot exclude the possibility that restriction activity could be measured under some other circumstance, such as phage predation.

### Proximity of m6A sites to -35 boxes of housekeeping sigma factor SigA regulates promoter activity

Due to the enrichment of m6A within particular genomic locations (Figure 1), we considered a role for m6A in regulating gene expression. Several prior studies have shown that DNA methylation from RM systems can also regulate gene expression (23,25,26). Accordingly, DNA MTase targets that occur within promoter or operator regions have the potential to influence transcription (50). Thus, we hypothesized that DnmA-dependent methylation might exhibit a similar function in *B. subtilis*.

**Figure 4.**
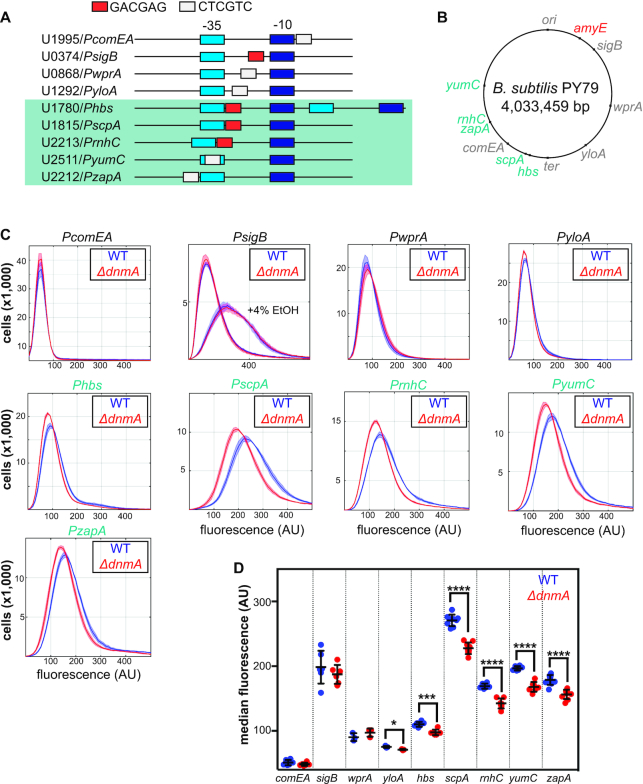
Methylation of DnmA motifs in proximity of -35 boxes affects downstream gene expression. (**A**) Schematic overview of the promoter regions containing DnmA sites that were selected for analysis using transcriptional GFP fusions. Indicated are the locations of the predicted sigma factor -35 and -10 boxes with respect to the DnmA motifs. U numbers correspond to the transcribed regions 5′ of ORFs identified by Nicolas *et al.* (51). (**B**) The location of the studied promoters on the PY79 chromosome map with respect to the *amyE* site used for integration and analysis of the promoter-GFP constructs. (**C**) Histograms depicting the GFP fluorescence in 200 000 WT (blue) or Δ*dnmA* (red) cells in three biological replicates that were grown in S7_50_ medium to an OD_600_ of 0.5 at 30°C and measured using flow cytometry. For U0374/P*sigB*, an additional experiment was performed in which the cells were treated with 4% EtOH an hour before analysis with flow cytometry. The standard deviations are represented as shaded areas. Promoter regions that appear methylation sensitive are shown in green. (**D**) Scatter dot plots, with indicated mean and standard deviation, depicting the median fluorescence of each strain taken from the histograms shown in (C) and appended with similar measurements taken on at least one different day. A standard T-test was performed to evaluate differential GFP expression between WT and Δ*dnmA* for each promoter. *P*-values: *P < 0.05, ***P < 0.005, ****P < 0.001.

To identify genes that might be affected by DnmA-dependent methylation, we used the list of transcribed regions 5′ of *B. subtilis* 168 open reading frames (ORFs) reported previously (51) to prioritize the subset of promoters in *B. subtilis* with m6A sites located on the left chromosomal arm where we observed m6A enrichment. The promoters chosen for analysis included those of non-coding and anti-sense RNAs as well as promoters embedded inside transcriptional units and we excluded promoters where the target site occurs downstream of the transcriptional start site (Supplementary Table S11). *B. subtilis* PY79 contains 32 transcribed regions 5′ of ORFs with the m6A motif in the vicinity of known or predicted sigma factor binding sites (Supplementary Table S11). To examine if m6A in promoter regions influences gene expression in *B. subtilis*, we constructed a series of transcriptional fusions where a *gfp* allele was introduced downstream of the respective m6A motif-containing promoter (Figure 4A). All transcriptional fusions were introduced at the ectopic *amyE* locus to separate the promoter from other potential *cis*-acting regulatory elements or chromosome structure contexts that could affect expression (Figure 4B). Promoter activity was monitored in WT and Δ*dnmA* strains using fluorescence as a reporter in single cells during mid-exponential growth by flow cytometry (please see Materials and Methods).

We found that loss of m6A in a subset of *B. subtilis* promoters, specifically those that contain an m6A motif in or slightly downstream of the –35 region of the SigA-binding box (*PscpA, Phbs, PrnhC, PyumC, PzapA*), consistently resulted in decreased activity from the unmethylated promoter relative to the methylated counterpart (Figure 4C and D). The m6A sites in the promoter region for *PscpA, Phbs, PrnhC, PyumC, PzapA* in PY79 are identical to the promoter regions in *B. subtilis* strain NCIB 3610.

We did not observe this trend for the promoter fusions that contained m6A sites away from the -35 box. For example, the activation level of the SigB-inducible *rsbV-rsbW-sigB-rsbX* promoter (PrsbV), with an m6A site directly upstream of the –10 box, was not influenced by the presence of methylation during normal growth or even after stressing the cells with 4% ethanol for 1-hour as described (52) (Figure 4C and D). Similarly, we did not observe differences in *gfp* expression with the *PcomEA*, *PwprA* or *PyloA* fusions in the Δ*dnmA* background relative to WT.

The m6A motif was present just upstream and overlapping the –35 region of the SigA binding box for *PzapA* (transcription unit: *zapA*-*yshB*-*polX*-*mutSB*-*yshE*) and *PyumC*, respectively, and both reporters showed a decrease in activity in Δ*dnmA* cells relative to WT (Figure 4C and D). ZapA is involved in FtsZ ring assembly and YumC is an essential ferrodoxin/flavodoxin reductase (53,54). The m6A site for the remaining three promoter fusions that showed decreased expression upon loss of m6A, *PscpA* (transcription unit: *scpA*-*scpB*-*ypuI*), *Phbs* (transcription unit: *S861-hbs*), and *PrnhC*, was located just downstream of the -35 region of the SigA binding box. Interestingly, the gene products for two of the differentially expressed promoter regions, *scpA* and *hbs*, have important roles in chromosome segregation, chromosome structure, and organization (55–60). The changes in *Phbs* activity were mild, which is likely due to the fact that *Phbs* contains two SigA-binding boxes, of which the m6A-positive box is the least dominant of the two promoters (61). Another promoter fusion that exhibited a DnmA-dependent increase in expression was P*rnhC*, which codes for RNase HIII, an enzyme important for cleavage of RNA-DNA hybrids (62,63). One type of RNA-DNA hybrid is an R-loop, which could affect local chromosome structure and transcription (64). Together, decreased expression from *PscpA*, *Phbs* and *PrnhC* could have impacts on global chromosome structure, altering the expression of other genes.

**Figure 5.**
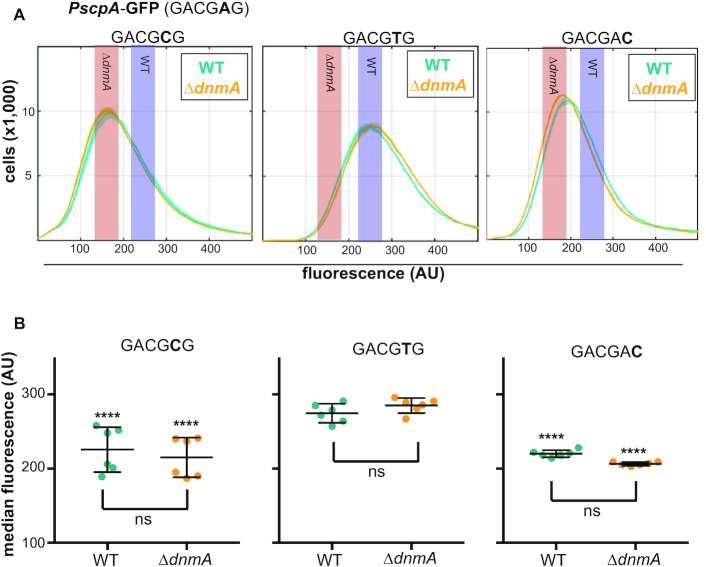
Mutating the DnmA recognition motif is sufficient for differential gene expression in the *PscpA* promoter. (**A**) Analysis of the effect of mutating WT GACG**A**G to GACG**C**G (first graph), GACG**T**G (second graph), or GACGA**C** (third graph) on the activity of P*scpA*-GFP in WT (teal) and Δ*dnmA* (orange) cells. (**B**) Scatter dot plots, with the indicated mean and standard deviation, of the median GFP fluorescence of each strain taken from the histograms shown in (A) and appended with measurements from a similar experiment taken on a separate day. The median values were tested against each other, including the median values from the strain expressing P*scpA*-GFP in WT cells, for differential expression using a one-way ANOVA post-hoc Tuckey test. *P*-values: *P < 0.05, ***P < 0.005, ****P < 0.001, ns = not significant.

To further investigate how m6A methylation affects transcription, the m6A site within the P*scpA*-GFP promoter was mutated to GACG**C**G, ensuring loss of methylation at this site in both the WT and Δ*dnmA* backgrounds. The GACG**C**G containing promoter adopted the same activity as observed in the Δ*dnmA* strain, indicating that m6A at the fifth position of the motif stimulates gene expression (Figure 5A and B). Interestingly, an A→T at the fifth position of the m6A site (GACGTG) made P*scpA*-GFP behave as if it were m6A (GACG^m^AG) in both WT and Δ*dnmA* backgrounds (Figure 5A and B middle panel). The reason for how thymidine in the fifth position of the motif stimulates gene expression to the same extent as m6A is unclear. To further test how integrity of the motif modulates P*scpA* activity, the motif was subsequently changed to GACGAC so that the fifth position was unchanged but the DnmA recognition site was lost. The promoter adopted the same activity as quantified in the Δ*dnmA* strain, indicating that m6A or T at the fifth position of the motif stimulates gene expression for the *scpA* promoter (Figure 5A and B). With these data we suggest that m6A is capable of regulating gene expression when located near the -35 binding site for SigA with methylation promoting gene expression from a subset of promoters in *B. subtilis*.

### Transcriptional repressor ScoC binds GACGAG sites

**Figure 6.**
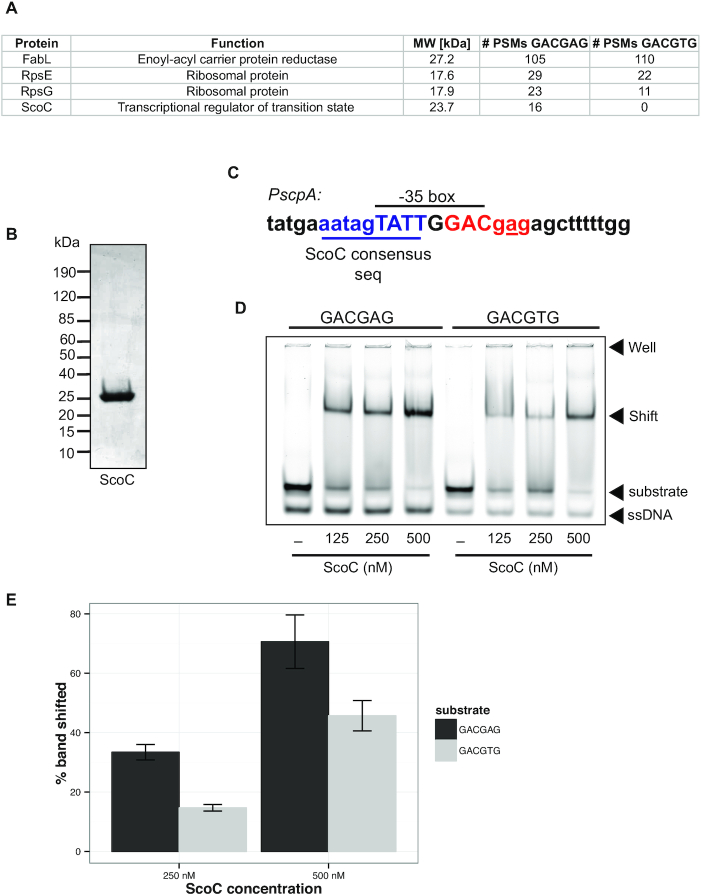
Transcription factor ScoC binds the *scpA* promoter with an unmodified GACGAG site. (**A**) Top protein hits identified in the pull-down of the biotinylated *scpA* promoter regions with GACGAG and GACGTG sites. The #PSMs indicates the total number of peptide spectra identified for each protein using the indicated oligo in the lysate pull-down assay. (**B**) SDS-polyacrylamide gel of ScoC overexpressed and purified from *E. coli* and stained with Coomassie. (**C**) Schematic of the *scpA* promoter region. The ScoC binding consensus sequence is shown in blue, the m6A site is in red, and the -35 box is also indicated. (**D**) ScoC binding to 5′ IR dye end-labeled *scpA* promoter region containing a GACGAG or GACGTG site was determined via EMSA. Representative electrophoretic mobility shift assay (EMSA) of ScoC binding to *scpA* promoter regions is shown. The concentration of ScoC is shown with (−) indicating the absence of ScoC from the reaction. Oligos containing the GACGAG or GACGTG site are also indicated at the top of the gel. The DNA substrates used in the reaction are otherwise identical. (**E**) Quantification of the percent band shifted using 250 and 500 nM concentrations of ScoC for the GACGAG and GACGTG oligos as indicated on the graph. The percent band shifted was normalized to the no protein control for each substrate. Three replicates were completed with the error bars representing the standard error between reactions.

The mechanism for m6A-dependent promotion of gene expression could be explained by an increase in SigA binding at methylated promoter regions or a less direct mechanism, such as competition for SigA binding with a methylation-sensitive transcriptional regulator. To determine if proteins in *B. subtilis* differentially associate with unmethylated DNA, we performed a pull-down in cell extracts using two different oligos. We amplified biotinylated oligos corresponding to the *PscpA* promoter region containing the GACGAG site. We could not obtain complete methylation of the substrate *in vitro* using purified DnmA. Therefore, we amplified the region and introduced a mutation in the m6A motif to GACG**T**G, which behaved like the WT methylated promoter in our reporter assay using the same promoter region (Figure 5A, B, middle panel). We isolated protein lysates from exponentially growing *B. subtilis* cells, incubated the lysates with our biotinylated oligos, performed a streptavidin pull-down, and visualized the proteins from each pull-down experiment via SDS-PAGE. We noted differences in the protein bands for the GACGAG relative to GACGTG oligo in the 20 and 40 kDa molecular weight range. These regions were excised from the gel and the proteins were identified using mass spectrometry. Of the top four most abundant proteins across the samples, the transcriptional regulator of the transition state, ScoC (65,66), was the only protein that did not appear in both pull-down experiments (Figure 6A). We found that ScoC was only present in the pull-down with the oligo that contained the GACGAG site, representing the unmethylated promoter state. No peptides corresponding to ScoC were identified in the pull-down of the GACGTG control site (Figure 6A).

To directly test if ScoC binding is affected by the A→T mutation, we purified ScoC (Figure 6B) and performed electrophoretic mobility shift assays (EMSAs). We used labeled oligos representing the *PscpA* promoter that only differed in the GACGAG and GACGTG sites, which overlap the –35 box but occur just outside of the ScoC consensus binding site (Figure 6C). The intensity of the shifted band was quantified and normalized to a no protein control for three independent experiments across a range of protein concentrations and the percent band shifted was compared at 250 nM and 500 nM ScoC. Consistent with the results from our pull-down experiment, we observed a 33.4% (S.E. ±2.6) and 14.7% (S.E. ±1.1) percent band shift at 250 nM ScoC for the GACGAG and GACGTG oligos, respectively (Figure 6D-E). We also observed percent band shifts of 70.6% (S.E. ±9.0) and 45.7% (S.E. ±5.1) at 500 nM ScoC for the GACGAG and GACGTG oligos, respectively (Figure 6D, E). The increased binding of ScoC to the oligo with the GACGAG site compared to the oligo with the GACGTG site (Figure 6E) and the decrease in expression we observed from the GACGAG promoter region compared to the GACGTG or GACG^m^AG promoter (Figure 5) supports the model that ScoC is a transcriptional repressor (65,66) and that ScoC shows preferential binding to an unmethylated promoter with the m6A site proximal to the ScoC binding site. With these results we suggest that ScoC binds to unmethylated GACGAG sites in promoter regions repressing transcription. When the GACGAG site overlaps or is adjacent to the ScoC binding site we suggest that methylation or A→T mutation at the fifth position could weaken ScoC binding leading to an increase in gene transcription.

## DISCUSSION

We report that DnmA (M.BsuPY79I or M.Bsu3610I) is responsible for endogenous m6A modifications that promote gene expression in *B. subtilis* strain PY79. We have shown that m6A in *B. subtilis* occurs at non-palindromic GACG^m^AG sites in the chromosome with enrichment on the left chromosomal arm. In *B. subtilis* PY79 there are only 1,219 chromosomal m6A sites in contrast to the ∼20 000 and ∼4500 palindromic m6A sites in *E. coli* and *C. crescentus*, respectively (67,68). While non-palindromic sites have been described (8) and have been shown to affect gene expression (25), the palindromic nature of m6A sites in *E. coli* and *C. crescentus* is necessary for function in DNA mismatch repair, origin sequestration, and cell cycle control (67). During these processes, protein binding or activity is dictated by full versus hemi-methylated states of m6A motifs, which determines the downstream regulatory role (67,69). Here, we have shown that loss of m6A at the non-palindromic GACG^m^AG sites in *B. subtilis* also affects the regulation of gene expression, with loss of methylation resulting in decreased expression of genes, including *scpA* and *hbs*, which code for proteins important for chromosome structure, organization, and maintenance (55–60) (Figure 4C and D). Our data indicate that the presence of m6A promotes the expression of a subset of genes in PY79 that could have important downstream effects on gene expression and chromosome structure.

One mechanism by which m6A regulates gene expression is through dictating transcription factor binding to promoter regions. In prototypical *E. coli* the methylation state of recognition sites for Dam methyltransferase in promoter regions has been shown to affect expression of a subset of genes, including virulence factors (67,69). One such example is the *agn43* promoter, where methylation at the promoter blocks binding of the redox sensitive repressor OxyR, thereby stimulating production of Agn43, which is important for non-fimbrical adhesion (70). Also, uropathogenic *E. coli* use phase variation to evade the host immune system by altering the expression of the pyelonephritis-associated pilus (pap) in a Dam methylation-dependent manner (24). In the Gram-negative pathogen *Neisseria meningitidis* non-palindromic m6A sites from an active Type III RM system also function in phase variation (25). The Gram-negative bacterium *C. crescentus* has a transcriptional activator, GcrA, which associates with RNA polymerase-σ^70^ and recognizes a subset of promoter regions that are methylated at palindromic recognition sites by the CcrM MTase (71).

Here, we have demonstrated that m6A regulated promoters in *B. subtilis* PY79 contain the methylation site at or slightly downstream of the -35 region of the housekeeping SigA binding site (72). We have shown that, in the absence of modification at the m6A site, we observe increased binding of the transcriptional repressor ScoC in the promoter region for the gene *scpA* (Figure 6A–E). The increased binding of the transcriptional repressor ScoC at the *scpA* promoter containing a GACGAG site relative to the GACGTG site supports our reporter results, showing that the GACGTG site phenocopied the higher expression levels in a wild type strain relative to the Δ*dnmA* strain (Figure 5A, B). We speculate that increased binding of the ScoC repressor to unmethylated GACGAG sites is responsible for the decreased gene expression we observe from the *scpA* promoter, representing one mechanism by which m6A could regulate gene expression in *B. subtilis* PY79.

While m6A-mediated binding of ScoC represents one mechanism by which m6A regulates gene expression, we find it likely that many other mechanisms exist. The methylation-responsive promoters identified in the current study do not share an obvious ScoC consensus binding sequence. Future work will be necessary to determine the additional regulatory mechanism(s) that result in increased gene expression at methylated promoter regions in *B. subtilis* PY79 and 3610.

Each of the promoter fusions tested was ectopically expressed at the *amyE* locus, which allowed us to assay for the effect of promoter methylation status independent of the effects of chromosomal location and local chromosome architecture. This experimental design allows for identification of promoter region activities that were affected by loss of methylation at the m6A site but did not account for other factors. Interestingly, as shown (Figure 4B), the genes for many of the downregulated promoter fusions occur toward the terminus (*hbs*, *scpA*, *rnhC* and *zapA*) and on the left arm of the chromosome, whereas the *amyE* locus is origin proximal and occurs on the right arm of the chromosome. Thus, we are able to conclude that methylation at the m6A site in *B. subtilis* PY79 promotes gene expression for a subset of genes but we cannot rule out other factors that control gene expression at the endogenous loci or indirect regulatory functions of m6A elsewhere in the chromosome.

In addition to its direct regulatory function at select promoter regions, m6A may have indirect effects on gene expression. It has been shown that m6A can increase the curvature of the DNA that may, in turn, influence protein binding and chromosome architecture (73,74). Alternatively, m6A might directly influence the expression of DNA binding proteins that contribute to chromosome architecture. Consistent with this hypothesis, we observe slight but significant downregulation of the *hbs* gene, which codes for the essential and highly abundant histone-like protein HBsu (Figure 4C). A potential decrease in HBsu levels concomitant with the preference of HBsu for highly curved regions of DNA creates the possibility for an m6A-dependent mechanism for changes in overall DNA topology and chromosome architecture. Thus, loss of m6A may affect protein occupancy throughout the chromosome to influence chromosome architecture in such a way that results in more changes to gene expression. It is important to note that both direct and indirect models of m6A-dependent changes are possible and that they are not mutually exclusive

Genomic m6A from orphan and active RM system MTases has been shown to function in the regulation of gene expression [e.g. (23–26)]. Here, we demonstrate that loss of MTase DnmA does not affect the natural transformation efficiency of foreign methylated DNA from a plasmid with multiple recognition sites in competent cells. Therefore, we suggest that DnmA is an MTase from an inefficient or inactive RM system. We have also discovered that DnmA-dependent m6A in the promoter regions of a subset of genes promotes gene expression in *B. subtilis* PY79 and we show that transcriptional repressor ScoC binds unmethylated DNA. In addition to influencing ScoC binding, we find it interesting that m6A promotes expression of several genes involved in chromosome structure and maintenance, which could in turn have effects on the expression of other genes. In total, we have shown that DNA methylation from DnmA has an effect on gene expression, prompting further investigation of RM systems and their possible regulatory contribution outside of DNA restriction.

## DATA AVAILABILITY

The SMRT sequencing data for PY79 and 3610 are available (40,41) with accession number CP006881 for PY79 and accession numbers CP020102 for the NCIB 3610 chromosome and pBS32 plasmid CP020103. The SMRT sequencing data for PY79 strains Δ*dnmA*, Δ*yabB*, P_spac_*dnmA*; Δ*dnmA*, Δ*dnmA*Δ*yabB*, and NCIB 3610 strain Δ*dnmA* is available [GEO GSE130695] at https://www.ncbi.nlm.nih.gov/geo. The raw data, equations, and descriptions of calculations used to generate Supplementary Figure S4F and Supplementary Table S5 have been deposited to http//figureshare.com DOI 10.6084/m9.figshare.8070995 and are publicly available.

## Supplementary Material

gkaa266_Supplemental_Files
