# Supplementary material for: Methyltransferase DnmA is responsible for genome-wide N6-methyladenosine modifications at non-palindromic recognition sites in Bacillus subtilis
Source: Nucleic Acids Res. 2020 Apr 23;48(10):5332–48. doi: 10.1093/nar/gkaa266 (PMC7261158; doi:10.1093/nar/gkaa266)
Supplement: gkaa266_Supplemental_Files [file gkaa266_supplemental_files.zip › DnmA_suppinfoFinalR4.pdf]

Supplementary Information

Methyltransferase DnmA is responsible for genome-wide N6-methyladenosine modifications at non-palindromic recognition sites in *Bacillus subtilis*

Taylor M. Nye, Lieke A. van Gijtenbeek, Amanda G. Stevens, Jeremy W. Schroeder, Justin R. Randall, Lindsay A. Matthews, Lyle A. Simmons*

Department of Molecular, Cellular, and Developmental Biology
University of Michigan, Ann Arbor, Michigan USA.

*To whom correspondence should be addressed: Department of Molecular, Cellular, and Developmental Biology, University of Michigan, Ann Arbor, Michigan 48109-1055, United States. Phone: (734) 647-2016, Fax: (734) 615-6337
E-mail: lasimm@umich.edu

Running Title: *Bacillus subtilis* m6A affects gene expression
Keywords: DNA methyltransferase, gene expression, *Bacillus subtilis,* SMRT sequencing, SigA

Supplementary Materials and Methods

Chromosomal DNA digestion by MspJI: Genomic DNA was purified from PY79, Δ*dnmA*, Δ*ydiOP*Δ*ydiR*, Δ*ydiOP*Δ*ydiS*, and Δ*ydiOP*Δ*ydjA* strains as described above and treated for six hours with MspJI according to the manufacturer's recommendations (New England BioLabs). For each control the reaction was set up exactly like the experimental group with an equivalent amount of water added instead of MspJI. Each reaction was then loaded on a 0.7% agarose gel and electrophoresed, stained with ethidium bromide, and visualized by illumination with UV.

DnmA (M.BsuPY79I) Y465A: A PCR reaction was performed using specially designed primers to create two overlapping blocks of DNA coding for *dnmA* with an alanine in the place of the tyrosine usually found in the NPPY catalytic motif. The 5' block was created by PCR using oTMN5 and oAS1 with *B. subtilis* genomic DNA as the template. The 3' block was created by PCR using oAS2 and oTMN7 with *B. subtilis* genomic DNA as the template. PCR products were gel extracted, purified, and combined with pE-SUMO vector via Gibson assembly to create pAS2. The resulting plasmid was used to transform *E. coli* MC1061 cells and plated on LB agar containing 25 µg/ml kanamycin. Resulting colonies were PCR screened for presence of the *dnmA* gene using oTMN5 and oTMN7 and further verified by Sanger sequencing. $BL21_{DE3}$ cells containing this plasmid were then tested for their ability to overexpress the mutant protein with addition of 200 µM IPTG.

Electrophoretic Mobile Shift Assay (EMSA): EMSAs were performed using 1 µM DnmA and 5' IR dye labeled substrates at 0.62 µM in a buffer containing 100 mM Tris-HCl pH 8, 250 mM NaCl, and 1 mM $MgSO_4$. The substrates were annealed in the same buffer by heating to 100°C for 30 seconds and then allowed to cool back to room

temperature on the bench top. Substrates included the target sequence (oAS09, oTMN39), non-target (oAS10, oTMN41), and a degenerate sequence (oAS11, oJR269). A no protein control was used for each substrate and catalytically inactive DnmA (Y465A) was assayed with the target sequence. These assays were performed at 30°C for 15 minutes. Samples were then loaded onto, and resolved via 6% native-PAGE electrophoresed on ice at 100V and visualized with a LI-COR Odyssey imager.

Spot titer assays: The indicated strains were struck from frozen stocks onto LB agar plates and incubated overnight at 30°C. Single colonies were inoculated into 2 mL of LB media and grown in a rolling rack at 37°C to an OD_{600} of 0.6-0.8. Strains were then diluted to an OD_{600} of 0.5 and subsequent 10-fold serial dilutions were performed in 0.85% saline solution. The dilutions (4 µL) were then spotted on LB agar and LB agar plus the indicated concentrations of exogenous DNA damaging agent or HU. Spots were allowed to dry, and the plates were incubated at 30°C overnight.

Mass Spectrometry: Mass spectrometry was performed by The University of Michigan Proteomics & Peptide Synthesis Core, project number MS976/M1516-086. Briefly, the band of interest was excised from SDS-PAGE and placed in 50 µl of distilled water. The band was then digested with trypsin and analyzed using LC/MS/MS on a ThermoFisher Orbitrap mass spectrometer. Resulting data was searched against the NCBI protein database and presented in Supplementary Table S10.

Spontaneous mutagenesis assay: Protocol was followed essentially as described (1). Briefly, frozen strains were struck out on LB and grown at 30°C overnight. Single colonies were inoculated into 3 mL of LB media and grow at 37°C to an OD_{600} between 1 and 1.2. At this point, 1.5 mL of culture was pelleted by centrifugation and the supernatant was aspirated. Cells were resuspended in 0.85% saline and two 1,000-fold

serial dilutions were performed in 0.85% saline. 100 µL of the original solution was plated on LB plates containing 100 µg/mL rifampin and grown at 30°C overnight and 100 µL from the 10^{-6} dilution was plated on LB and grown at 30°C overnight. The number of single colonies on each plate was counted the next morning and mutation rate was calculated using the Ma-Sandri-Sarkar Maximum Likelihood Estimator Method through the FALCOR fluctuation analysis calculator (2). All strains were independently grown and plated on at least three different days.

Live cell microscopy: Protocol was followed essentially as described (3). Frozen strains were struck on LB plates and grown overnight at 37°C. Plates were washed with defined $S7_{50}$ minimal media and diluted back to an OD_{600} of 0.05 in 2 mL of defined $S7_{50}$ minimal media and grown at 37°C to mid-exponential growth phase (OD_{600} between 0.6-0.8). 1 mL aliquots were then treated with 1 µL of FM4-64, the vital membrane strain, and spotted onto 1% agarose pads containing 1X Spizizen's salts. Fluorescence microscopy was performed with an Olympus BX61 microscope. The Olympus 100X oil immersion 1.45 numerical aperture (NA) total internal reflection fluorescence microscopy (TIRFM) objective lens was used for all imaging and all strains were independently imaged on at least three different days.

Strain construction

JWS261 (Δ*ydiOP,* Δ*ydiR*): PY79 was transformed with genomic DNA from BKE06090 to make strain JWS245. JWS245 was transformed with pDR224 to make JWS248. JWS248 was transformed with pJS146.

JWS262 (Δ*ydiOP,* Δ*ydiS*): PY79 was transformed with genomic DNA from BKE06100 to make strain JWS246. JWS246 was transformed with pDR224 to make JWS249. JWS249 was transformed with pJS146.

JWS263 (Δ*ydiOP,* Δ*ydjA*): PY79 was transformed with genomic DNA from BKE06110 to make strain JWS247. JWS247 was transformed with pDR224 to make JWS250. JWS250 was transformed with pJS146.

TMN1 and TMN2 (Δ*yabB)*: PY79 was transformed with genomic DNA from BKE00340 to make strain JWS230. JWS230 was transformed with pDR224.

TMN5 and TMN6 (Δ*dnmA)*: PY79 was transformed with genomic DNA from BKE06760 to make strain JWS230. JWS230 was transformed with pDR224.

TMN16 (Δ*dnmA, amyE::Pspac dnmA*): TMN5 was transformed with pTN003.

TMN47 (Δ*dnmA* in NCIB 3610*)*: DK1042 was transformed with genomic DNA from JWS230. JWS230 was transformed with pDR224.

JWS260 (Δ*dnmA, spo0J::spo0J-gfp*): TMN5 was transformed with genomic DNA from JWS259.

TMN80 (Δ*dnmA, spo0J::spo0J-gfp)*: TMN2 was transformed with genomic DNA from JWS259.

LVG066 (*amyE*::*PrbsV-GFP*): PY79 was transformed with plasmid pLVG1-374.

LVG067 (Δ*dnmA*, *amyE*::*PrbsV-GFP*): TMN06 was transformed with plasmid pLVG1-0374.

LVG068 (*amyE*::*PwprA-GFP*): PY79 was transformed with plasmid pLVG1-0868.

LVG069 (Δ*dnmA*, *amyE*::*PwprA-GFP*): TMN06 was transformed with plasmid pLVG1-0868.

LVG070 (*amyE*::*PyloA-GFP*): PY79 was transformed with plasmid pLVG1-1292.

LVG071 (Δ*dnmA*, *amyE*::*PyloA-GFP*): TMN06 was transformed with plasmid pLVG1-1292.

LVG072 (*amyE*::*PzapA-GFP*): PY79 was transformed with Gibson assembled fragment fLVG-2213.

LVG073 (Δ*dnmA*, *amyE*::*PzapA-GFP*): TMN06 was transformed with Gibson assembled fragment fLVG-2213.

LVG074 (*amyE*::*PrnhC-GFP*): PY79 was transformed with Gibson assembled fragment fLVG-2212.

LVG075 (Δ*dnmA*, *amyE*::*PrnhC-GFP*): TMN06 was transformed with Gibson assembled fragment fLVG-2212.

LVG079 (*amyE*::*PcomEA-GFP*): PY79 was transformed with plasmid pLVG1-1995.

LVG080 (Δ*dnmA*, *amyE*::*PcomEA-GFP*): TMN06 was transformed with plasmid pLVG1-1995.

LVG081 (*amyE*::*PezrA-GFP*): PY79 was transformed with plasmid pLVG1-2292.

LVG082 (Δ*dnmA*, *amyE*::*PezrA-GFP*): TMN06 was transformed with plasmid pLVG1-2292.

LVG087 (*amyE*::*PscpA-GFP*): PY79 was transformed with Gibson assembled fragment fLVG-1815.

LVG088 (Δ*dnmA*, *amyE*::*PscpA-GFP*): TMN06 was transformed with Gibson assembled fragment fLVG-1815.

LVG108 (Δ*dnmA operon*, *amyE*::*PscpA-GFP*): TMN17 was transformed with Gibson assembled fragment fLVG-1815.

LVG102 (*amyE*::*PscpA*^{mut1}*-GFP*): PY79 was transformed with Gibson assembled fragment fLVG-1815mut1.

LVG103 (Δ*dnmA*, *amyE*::*PscpA*^{mut1}*-GFP*): TMN06 was transformed with Gibson assembled fragment fLVG-1815mut1.

LVG109 (Δ*dnmA operon*, *amyE*::*PscpA*^{mut1}*-GFP*): TMN17 was transformed with Gibson assembled fragment fLVG-1815mut1.

LVG105 (*amyE*::*Phbs-GFP*): PY79 was transformed with Gibson assembled fragment fLVG-1780.

LVG106 (Δ*dnmA*, *amyE*::*Phbs-GFP*): TMN06 was transformed with Gibson assembled fragment fLVG-1780.

LVG118 (*amyE*::*PscpA*^{mut2}*-GFP*): PY79 was transformed with Gibson assembled fragment fLVG-1815mut2.

LVG119 (Δ*dnmA operon*, *amyE*::*PscpA*^{mut2}*-GFP*): TMN17 was transformed with Gibson assembled fragment fLVG-1815mut2.

LVG120 (*amyE*::*PscpA*^{mut3}*-GFP*): PY79 was transformed with Gibson assembled fragment fLVG-1815mut3.

LVG121(Δ*dnmA operon*, *amyE*::*PscpA*^{mut3}*-GFP*): TMN17 was transformed with Gibson assembled fragment fLVG-1815mut3.

Plasmid construction

General cloning techniques

All pLVG1-derived plasmids and *amyE*-containing linear fragments were assembled using Gibson assembly (4). Enzymatic assembly of overlapping DNA fragments, or overlap extension PCR. Gibson assemblies consisted of 30-80 ng of each PCR product

and 1X Gibson assembly mastermix (0.1 M Tris pH 8.0, 5% PEG-8000, 10 mM MgCl2, 10 mM DTT, 0.2 mM dNTPs, 1 mM NAD+, 4 units/mL T5 exonuclease, 25 units/mL Phusion DNA polymerase, 4,000 units/mL Taq DNA ligase) in a total reaction volume of 10-12 μL. The reactions were incubated at 50°C for 90 minutes. Gibson-assembled plasmids were used to transform *E. coli* MC1061. Gibson-assembled linear fragments were purified using spin columns, re-amplified using Phusion polymerase and used to transform PY79 or PY79 derivatives. For overlap extension PCR, 500 ng of each PCR product was mixed and standard PCR cycling was performed using end primers and Q5 polymerase (NEB). PCR fragments were routinely obtained using Phusion polymerase (NEB) or Q5 polymerase (NEB) and gel-purified before Gibson assembly or overlap extension PCR.

Individual plasmid (p) construction

pJS146: The regions 500 base pairs upstream and downstream of the *ydiOP* operon were amplified from PY79 genomic DNA using primers oJS650 and oJS651 (upstream region) and oJS653 and oJS657 (downstream region). The fragments were then combined with the pminiMAD vector using Gibson assembly.

pTN02: The *dnmA* gene was cloned from PY79 genomic DNA using primers oTN3 and oTN8 with overlapping regions to the pDR110 vector. The pDR110 vector and insert were combined using Gibson assembly.

pTN03: The *dnmA* gene was cloned from PY79 genomic DNA using primers oTN5 and oTN7 with overlapping regions to the pE-SUMO vector. The pE-SUMO vector and insert were combined using Gibson assembly.

pAS2: Overlap PCR was used to make the Y→A substitution. The 5' block was created by using oTMN5 and oAS1 with PY79 genomic DNA as a template. The 3' block was created by using oTMN7 and oAS2 with PY79 genomic DNA as a template. PCR products were gel purified and combined with the pE-SUMO vector using Gibson assembly.

pTN12: The *yabB* gene was cloned from PY79 genomic DNA using primers oTN36 and oTN37 with overlapping regions to the pE-SUMO vector. The pE-SUMO vector and insert were combined using Gibson assembly.

pTN13: The *scoC* gene was cloned from PY79 genomic DNA using primers oTN62 and oTN63 with overlapping regions to the pE-SUMO vector. The pE-SUMO vector and insert were combined using Gibson assembly.

pLVG1: To remove the *lacI* gene from pDR111_GFP(Sp) (5), the plasmid was amplified using primers oLVGLS024A and oLVGLS024B, restricted with BamHI and self-ligated with T4 DNA ligase.

pLVG1-0374: The backbone of pLVG1 without P*xyl* was amplified with primers oLVGLS023A and oLVGLS023B and combined with a DNA fragment containing U0374 (P*rsbV*), amplified from PY79 genomic DNA using primers oLVG_U0374F and oLVG_U0374R.

pLVG1-0868: The backbone of pLVG1 without P*xyl* was amplified with primers oLVGLS023A and oLVGLS023B and combined with a DNA fragment containing U0868 (P*wprA*), amplified from PY79 genomic DNA using primers oLVG_U0868F and oLVG_U0868R.

pLVG1-1292: The backbone of pLVG1 without P*xyl* was amplified with primers oLVGLS023A and oLVGLS023B and combined with a DNA fragment containing U1292 (P*yloA*), amplified from PY79 genomic DNA using primers oLVG_U1292F and oLVG_U1292R.

pLVG1-1995: The backbone of pLVG1 without P*xyl* was amplified with primers oLVGLS023A and oLVGLS023B and combined with a DNA fragment containing U1995 (P*comEA*), amplified from PY79 genomic DNA using primers oLVG_U1995F and oLVG_U1995R.

pLVG1-2292: The backbone of pLVG1 without P*xyl* was amplified with primers oLVGLS023A and oLVGLS023B and combined with a DNA fragment containing U2292 (P*ezrA*), amplified from PY79 genomic DNA using primers oLVG_U2292F and oLVG_U2292R.

Individual DNA fragment (f) construction

fLVG-1780: An upstream DNA fragment was amplified from pLVG1 using primers oLVGLS023C and oLVGLS023A. A downstream DNA was amplified from pLVG1 using primers oLVGLS023B and oLVGLS023D. A DNA fragment containing U1780(P*hbs*) was amplified from PY79 genomic DNA using primers oLVG_U1780F and oLVG_U1780R. The three fragments were assembled using Gibson assembly and the correct construct was enriched using end primers oLVGLS034 and oKJW090.

fLVG-1815: An upstream DNA fragment was amplified from pLVG1 using primers oLVGLS023C and oLVGLS023A. A downstream DNA was amplified from pLVG1 using primers oLVGLS023B and oLVGLS023D. A DNA fragment containing U1815 (P*scpA*) was amplified from PY79 genomic DNA using primers oLVG_U1815F and oLVG_U1815R. The three fragments were assembled using Gibson assembly and the correct construct was enriched using end primers oLVGLS034 and oKJW090.

fLVG-1815mut1: To replace 5'-GACGAG with 5'-GACGTG in the *scpA* promoter, an upstream and downstream DNA fragment was amplified from LVG087 genomic DNA using primer pair oLVGLS042A/oKJW89 and oLVGLS042B/oKJW090, respectively. The fragments were assembled by overlap extension PCR.

fLVG-1815mut2: To replace 5'-GACGAG with 5'-GACGCG in the *scpA* promoter, an upstream and downstream DNA fragment was amplified from LVG087 genomic DNA using primer pair oLVGLS044A/oKJW89 and oLVGLS044B/oKJW090, respectively. The fragments were assembled by overlap extension PCR.

fLVG-1815mut3: To replace 5'-GACGAC with 5'-GACGAC in the *scpA* promoter, an upstream and downstream DNA fragment was amplified from LVG087 genomic DNA using primer pair oLVGLS045A/oKJW89 and oLVGLS045B/oKJW090, respectively. The fragments were assembled by overlap extension PCR.

fLVG-2212: An upstream DNA fragment was amplified from pLVG1 using primers oLVGLS023C and oLVGLS023A. A downstream DNA was amplified from pLVG1 using primers oLVGLS023B and oLVGLS023D. A DNA fragment containing U2212 (P*rnhC*) was amplified from PY79 genomic DNA using primers oLVG_U2212F and oLVG_U2212R. The three fragments were assembled using Gibson assembly and the correct construct was enriched using end primers oLVGLS034 and oLVGLS090.

fLVG-2213: An upstream DNA fragment was amplified from pLVG1 using primers oLVGLS023C and oLVGLS023A. A downstream DNA was amplified from pLVG1 using primers oLVGLS023B and oLVGLS023D. A DNA fragment containing U2213 (P*zapA*) was amplified from PY79 genomic DNA using primers oLVG_U2213F and oLVG_U2213R. The three fragments were assembled using Gibson assembly and the correct construct was enriched using end primers oLVGLS034 and oKWJ90.

Supplementary Results

m5C modifications function as part of the BsuMI restriction-modification system.

The analysis of SMRT sequencing detected cytidine methylation in the PY79 genome **(Table 1)**. In *B. subtilis* Marburg the BsuMI RM system was first found to recognize 5' YTCGAR sites and later refined using analysis of transformation efficiency to recognize 5' CTCGAG (6). This work showed that in *B. subtilis* Marburg the *ydiO-ydiP* operon codes for the methyltransferase (MTase) responsible for m5C modifications of the BsuMI RM system and that an adjacent operon, *ydiR-ydiS-ydjA,* codes for the cognate endonuclease (6). Given the sequence similarity between the mC motif detected in the WT strain PY79, 5' CTCGARB, and the site identified in the Marburg strain, 5' CTCGAG, we decided to test whether YdiO-YdiP was responsible for cytidine methylation in PY79. Because PacBio does not robustly detect m5C methylation, we experimentally determined the modification type by treating DNA with the m5C- and 5-

hydroxymethylcytosine-specific endonuclease, MspJI (Supplementary **Figure S3)**. We created PY79 strains with deletions of *ydiO-ydiP* and each subunit of the putative endonuclease, *ydiR, ydiS,* or *ydjA.* DNA was purified from each of these strains in addition to WT and a strain with a deletion of N6-methyladenosine methyltransferase Δ*dnmA*, as controls. DNA from WT, Δ*dnmA*, and strains lacking *ydiO-ydiP* plus the respective restriction endonuclease subunits were treated with MspJI, recognizes 5-5hmC and m5C at 5' ^{m}CNNR sites, followed by electrophoresis on an agarose gel. Smearing in WT and Δ*dnmA* strains indicates the presence of m5C modifications whereas distinct bands in Δ*ydiOP*Δ*ydiR,* Δ*ydiOP*Δ*ydiS,* Δ*ydiOP*Δ*ydjA* strains indicates loss of m5C modifications, implicating *ydiOP* as the MTase responsible for all m5C methylation in the *B. subtilis* genome (Supplementary **Figure S3**). The results we present here confirm the BsuMI RM recognition site as 5'CTCGARB in *B. subtilis* strain PY79. The m5C motif identified in PY79 was not detected as modified in NCIB 3610 by PacBio SMRT sequencing **(Table 1)**.

***B. subtilis* m6A does not function in replication timing.** We sought to determine the consequence of m6A loss in *B. subtilis* cells. In the Gram-negative bacterium *E. coli*, GATC-specific m6A functions in origin sequestration (7-9), DNA mismatch repair (10,11), and the regulation of gene expression (12). The methylation status of palindromic GATC sites in the *E. coli* origin of replication regulates the binding of SeqA, which inhibits origin firing by sequestering the origin region (7-9). Whereas GATC sites are enriched in the *E. coli* origin, as discussed in the main text the m6A motif is not present in the *B. subtilis* replication origin, although a high density of m6A sites flank the origin on the left arm **(Fig 1)**. To empirically determine if m6A sites located in the origin proximal region on the left arm influence origin duplication, we assessed the origin proximal copy number in exponentially growing WT and Δ*dnmA* (M.BsuPY79I) cells

using Spo0J-GFP (*parB-gfp*) as a marker for origin copy number as done previously (13,14). We show that in WT, Δ*dnmA,* Δ*yabB* cells, 65%, 64%, 66% of cells showed two Spo0J-GFP foci, respectively. As controls we used a deletion of *yabA*, a negative regulator of origin firing (15), and show that 74% of cells have four or more foci as expected (14). As a hypo-initiation control we used an IPTG regulated promoter (P_{spac}*dnaAN*) to deplete the replication initiation protein *dnaA* and the replication sliding clamp *dnaN*. We show a near 8-fold increase in the percentage of cells with a single Spo0J-GFP focus, demonstrating an inhibition of DNA replication initiation (14) **(**Supplementary **Figure S4)**. With these results we show no difference in origin proximal copy number between WT, Δ*dnmA,* or Δ*yabB* cells as determined by fluorescence microscopy and we conclude that m6A does not contribute to the regulation of DNA replication initiation.

***B. subtilis* m6A does not function in DNA mismatch repair.** In addition to origin sequestration, methylation at GATC sites in *E. coli* also functions in strand discrimination during DNA mismatch repair, thereby ensuring removal of mismatched bases from the nascent strand (10). Both the loss of adenosine methylation at GATC sites and hyper-methylation of the chromosome by overexpression of Dam resulted in an increase in spontaneous mutation rate (16,17). m6A sites are non-palindromic in *B. subtilis* and occur far less frequently (~1,200 sites relative to ~20,000 GATC sites in *E. coli*). The lack of an even distribution on the leading and lagging strands across the genome and the low number of sites does not support a contribution of m6A to strand discrimination during mismatch repair. To be certain, we conducted rifampin resistance assays as a measure for mutation rate (1,18,19) in WT and Δ*dnmA* strains. No difference in mutation rate between these strains was observed as compared to a mismatch repair deleted control **(**Supplementary **Table S5)**. These results indicated that the presence or absence

of m6A does not influence spontaneous mutagenesis in *B. subtilis* (20,21). Furthermore, because the m6A sites occur multiple times at the *addA* locus and AddA is important for recombinational repair (22), we performed spot titer assays to determine if Δ*dnmA* cells were more sensitized to DNA damaging agents relative to WT cells and found no increase in sensitivity **(**Supplementary **Figure S8)**.

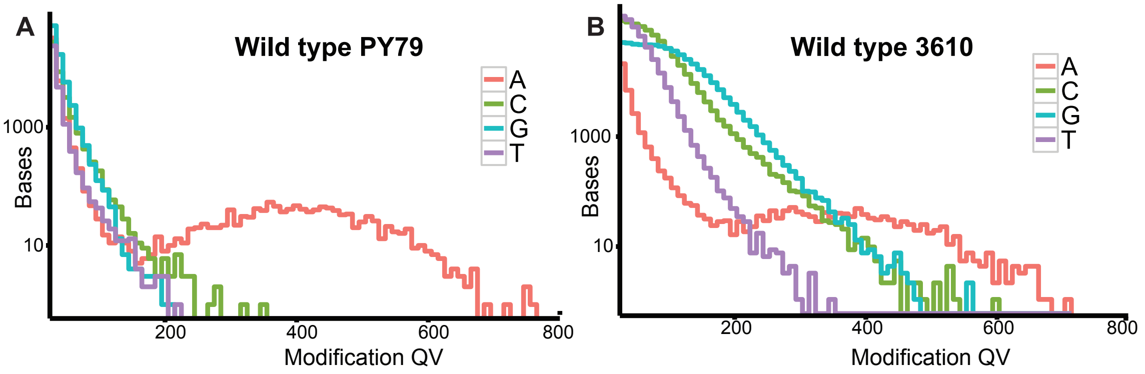

Supplementary Figure S1. The genome of *B. subtilis* strains contain m6A modifications. (A) PacBio SMRT sequencing of genomic DNA isolated from WT PY79 cells. Modification quality values (modQVs) indicate if the kinetics of the DNA polymerase differs from the expected background at a particular locus, where a modQV of 30 represents a p-value of 0.001. ModQVs are indicated on the x-axis and the number of bases is indicated on the y-axis. Each line represents the modification quality values for a particular nucleotide. **(B)** PacBio SMRT sequencing of genomic DNA isolated from the WT ancestral strain NCIB 3610.

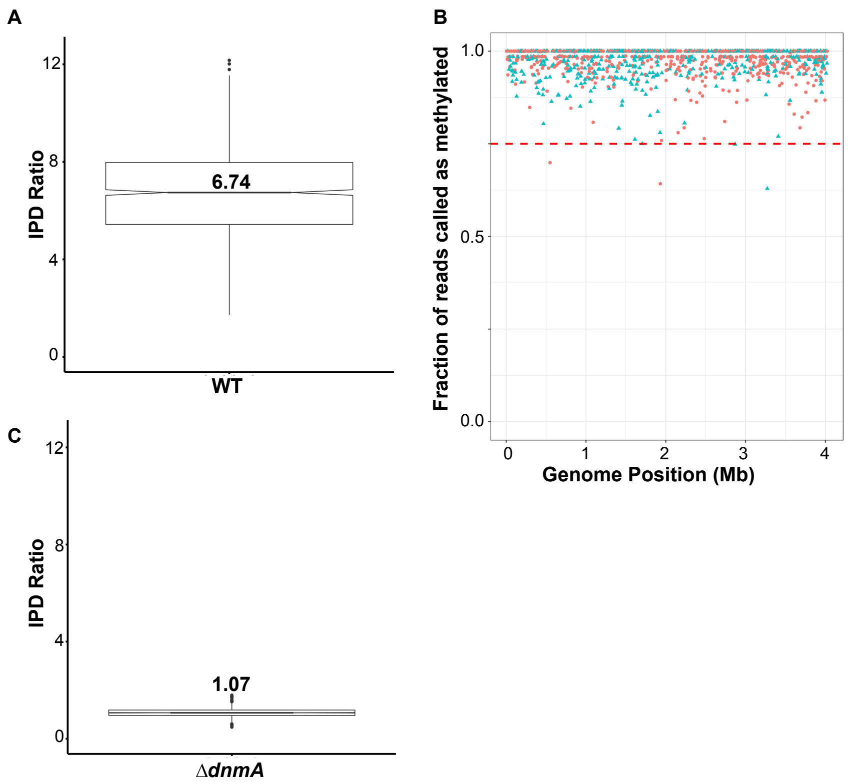

Supplementary Figure S2. GACG^{m}AG sites have high modification scores throughout the *B. subtilis* PY79 genome. (A) Representative boxplot of interpulse duration (IPD) ratio values at GACGAG sites throughout the genome in WT cells. The median IPD ratio value is indicated. **(B)** The genomic location of each GACG^{m}A̲G site (x-axis) and the corresponding fraction of reads that were called as methylated at that position (y-axis) from PacBio SMRT sequencing is plotted. Sites that appear on the plus strand are indicated as a green triangle and those that appear on the minus strand are indicated as red dots. **(C)** Representative boxplot of the IPD ratio values at GACGAG sites throughout the genome in Δ*dnmA* (M.BsuPY79I) cells. Median IPD ratio value is indicated.

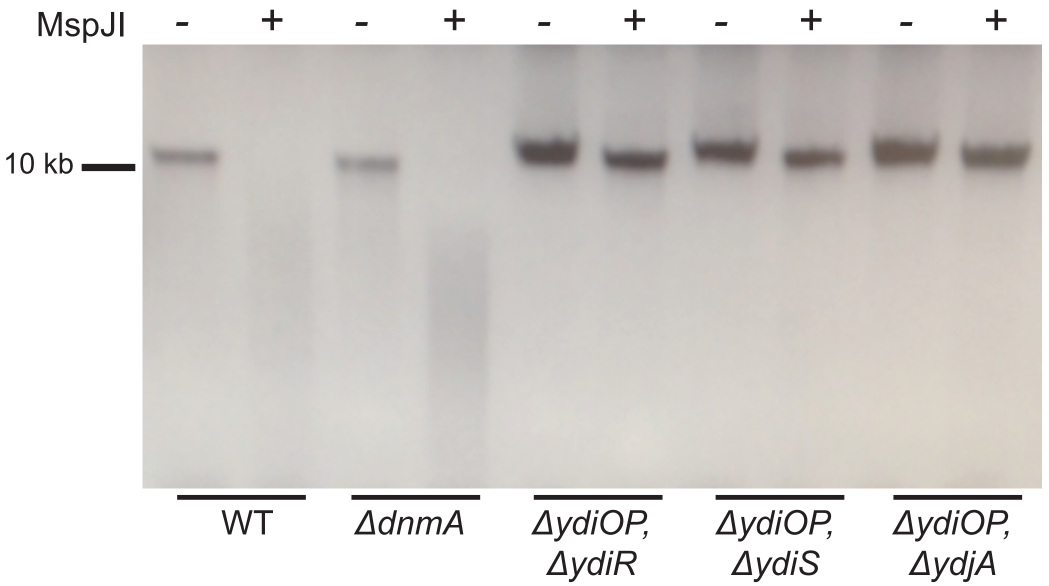

Supplementary Figure S3. Deletion of the BsuMI RM system eliminates m5C from the *B. subtilis* chromosome. Clean deletions were made for the coding regions of both subunits of the putative methyltransferase (*ydiOP*) in conjunction with separate deletions for each gene in a nearby operon coding for a putative restriction endonuclease (*ydiR, ydiS, ydjA*). DNA purified from these strains was subjected to 6 hours of treatment with a 5-methylcytidine and 5-hydroxymethylcytidine specific endonuclease MspJI. (-) indicates no treatment, (+) indicates treatment with MspJI.

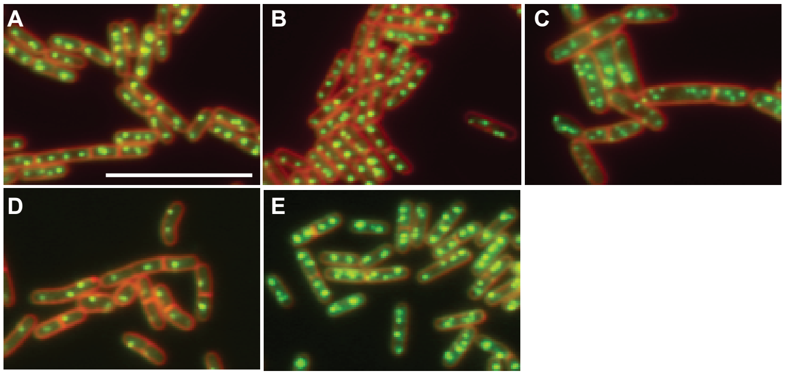

F

Relevant genotype	No. of cells	Percentage of cells with *n* Spo0J-GFP foci				
		1	2	3	4	>4
wild type	1079	6	65	15	14	<1
Δ*dnmA*	1083	7	64	13	15	1
yabA::cat	1059	3	8	9	12	74
DnaAN depletion	1072	47	40	7	3	5
Δ*yabB*	1029	2	66	15	15	2

Supplementary Figure S4. Origin firing in *B. subtilis* is not regulated m6A. (A-E) Representative images of fluorescence microscopy for (A) WT, (B) Δ*dnmA*, (C) *yabA::cat*, (D) *dnaAN* depletion, and (E) Δ*yabB* strains expressing *spo0J::spo0J-gfp*, respectively. White bar = 10 μm. **(F)** Quantification of Spo0J-GFP foci for strains A-E.

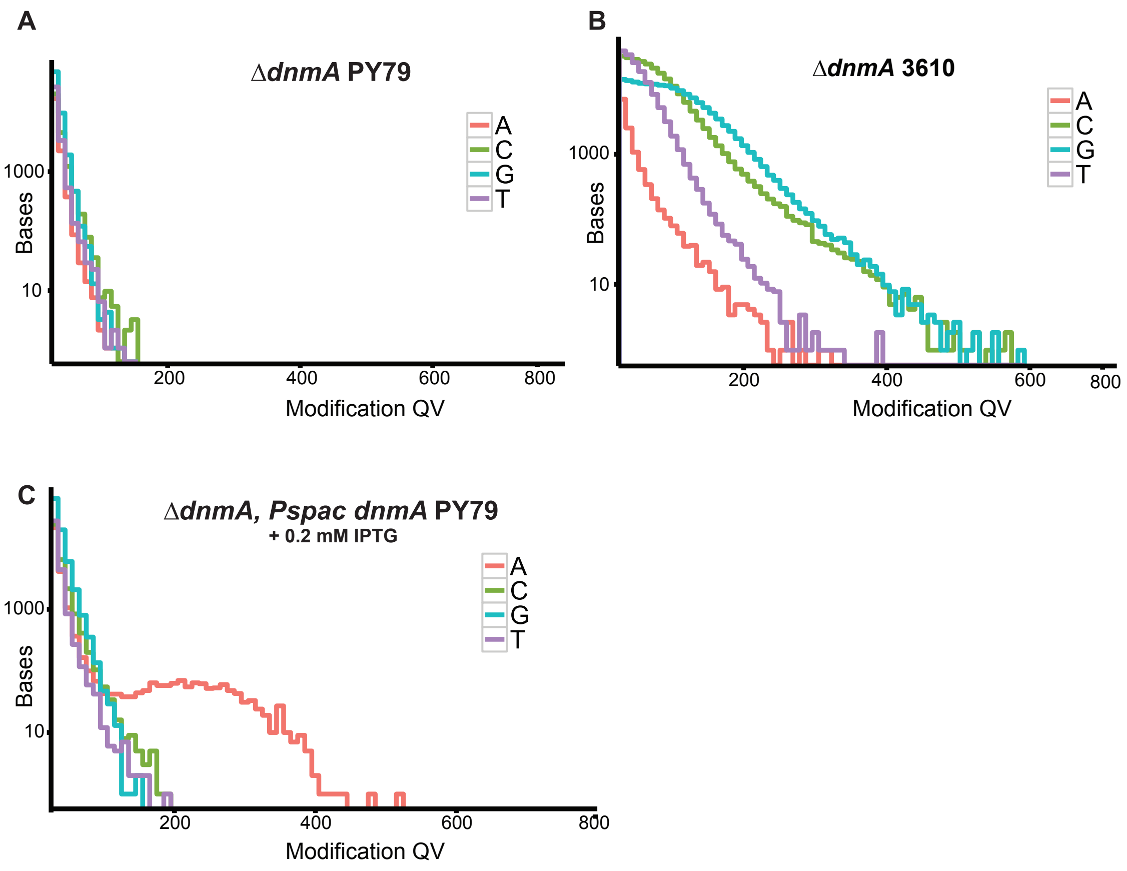

Supplementary Figure S5. *Bacillus subtilis* m6A modifications are dependent on methyltransferase DnmA. (A) PacBio SMRT sequencing of genomic DNA isolated from the Δ*dnmA* PY79 strain. **(B)** PacBio SMRT sequencing of genomic DNA isolated from the ancestral strain NCIB 3610 with a *dnmA* deletion. **(C)** PacBio SMRT sequencing of genomic DNA isolated from Δ*dnmA* cells ectopically expressing *dnmA* from the *amyE* locus with 0.2 mM IPTG.

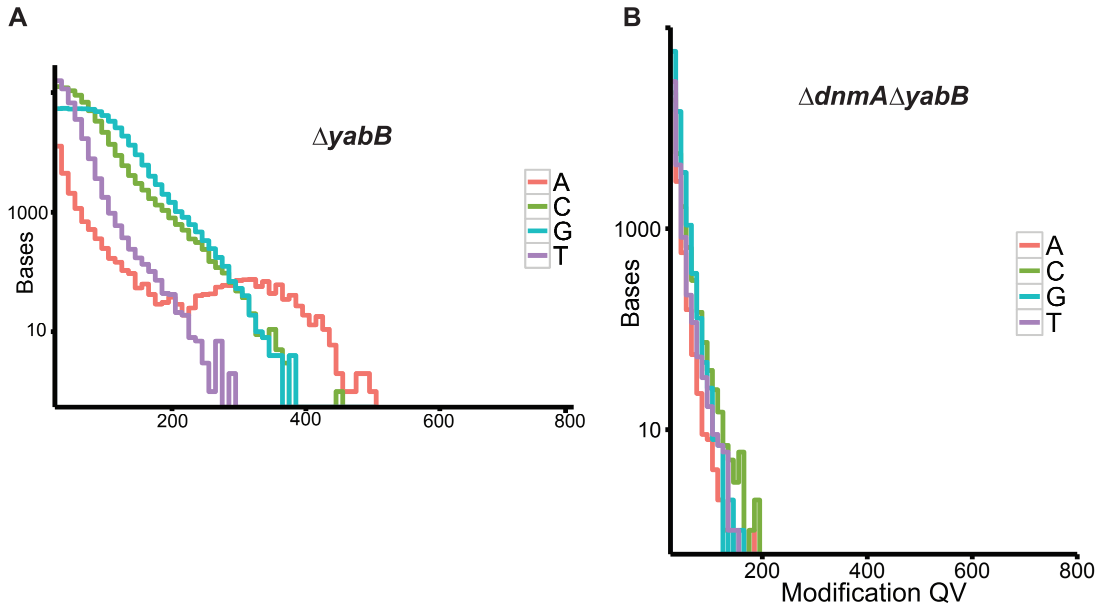

Supplementary Figure S6. Genomic m6A is present in a *yabB* deletion strain. (A) PacBio SMRT sequencing of genomic DNA isolated fromPY79 Δ*yabB* cells. **(B)** PacBio SMRT sequencing of genomic DNA isolated from PY79 Δ*dnmA*Δ*yabB* cells.

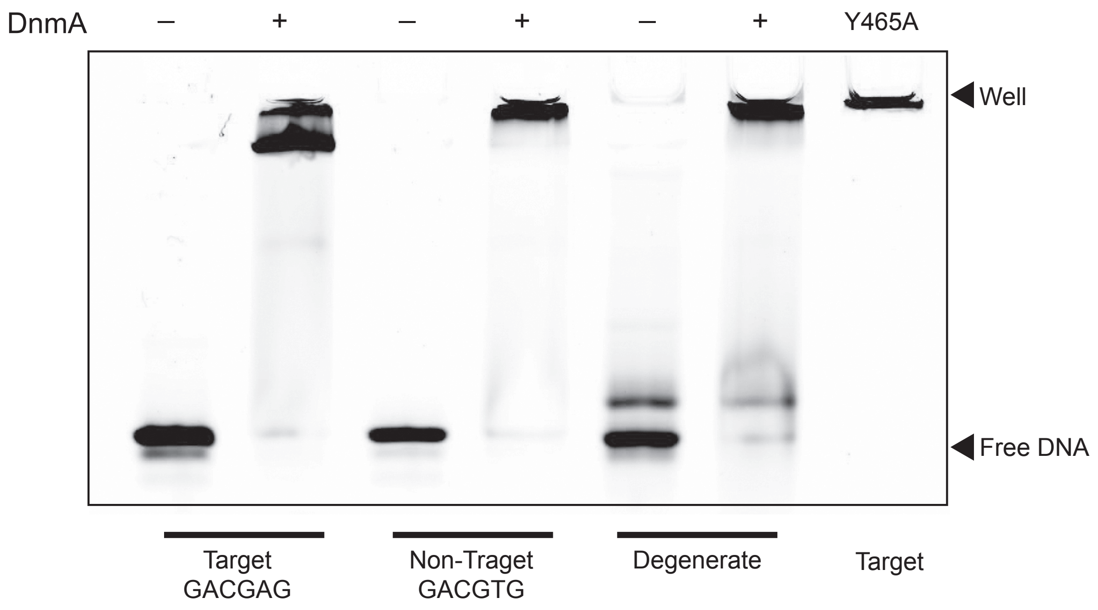

Supplementary Figure S7. DnmA binds DNA without the m6A motif. DnmA (M.BsuPY79I) substrate binding was determined by electrophoretic mobility shift assay (EMSA) with purified DnmA and varying substrates. 5' IR-labeled substrates include: target substrate (GACGAG), non-target substrate (GACG**T**G) and a degenerate sequence substrate, which are indicated at the bottom. The (-) indicates the absence of DnmA from the reaction, (+) indicates addition of DnmA to the reaction. As indicated, the final lane includes the DnmA catalytic inactive variant (Y465A) incubated with the target substrate.

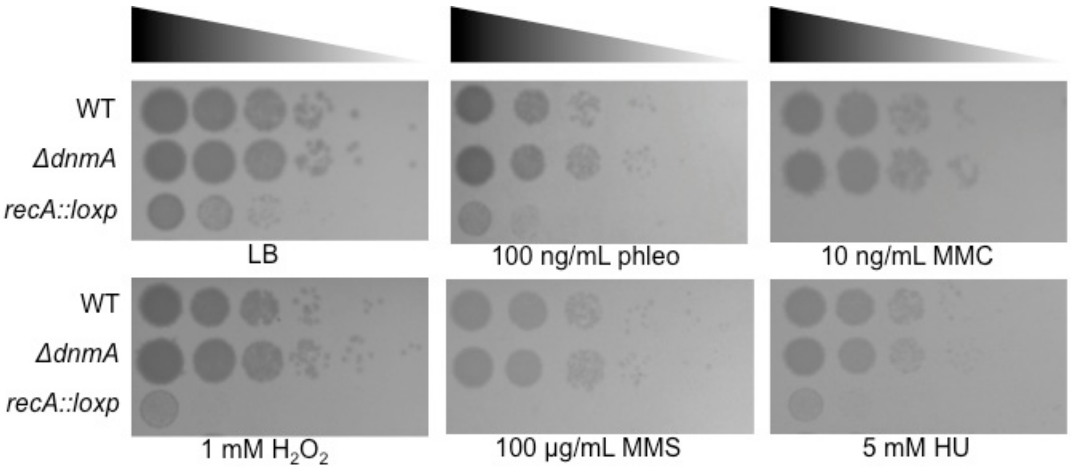

Supplementary Figure S8. Loss of m6A does not cause an increased susceptibility to genotoxic stress. WT and Δ*dnmA* cells were tested for their sensitivity to several DNA damaging agents and replication fork stress caused by hydroxyurea (HU). Cells were grown to mid-exponential growth phase, serially diluted, and plated on LB agar plates with the following concentrations of DNA damaging agents: 100 ng/mL phleomycin (phleo), 10 ng/mL mitomycin C (MMC), 1 mM hydrogen peroxide (H_2O_2), 100 µg/mL methyl methanesulfonate (MMS), and 5 mM hydroxyurea (HU). Cells with a *recA::loxP* disruption were used as a control.

Supplementary Table S1. Strains used in this study

Strain	Genotype	Source
JWS10	PY79	(23)
JWS261	Δ*ydiOP*, Δ*ydiR*	This work
JWS262	Δ*ydiOP*, Δ*ydiS*	This work
JWS263	Δ*ydiOP*, Δ*ydjA*	This work
TMN1	Δ*yabB*	This work
TMN5	Δ*dnmA* (*M.BsuPY79I*)	This work
TMN7	Δ*dnmA*, Δ*yabB*	This work
DK1042	NCIB 3610 *comIQ12I*	(24)
TMN47	NCIB 3610, Δ*dnmA* (*M.Bsu3610I*)	This work
TMN16	Δ*dnmA*, *amyE::*P_{spac}*dnmA*	This work
JWS259	*spo0J::spo0J-gfp*	
JWS260	Δ*dnmA*, *spo0J::spo0J-gfp*	This work
TMN80	Δ*yabB*, *spo0J::spo0J-gfp*	This work
AK42	*yabA::cat*, *spo0J::spo0J-gfp*	Lab stock
LAS254	P_{spac}*dnaAN::cat*, *spo0J::spo0J-gfp*	(14)
BTS13	Δ*mutSL::spc*	(25)
LVG066	*amyE::PrbsV-GFP*	This work
LVG067	Δ*dnmA*, *amyE::PrbsV-GFP*	This work
LVG068	*amyE::PwprA-GFP*	This work
LVG069	Δ*dnmA*, *amyE::PwprA-GFP*	This work
LVG070	*amyE::PyloA-GFP*	This work
LVG071	Δ*dnmA*, *amyE::PyloA-GFP*	This work
LVG072	*amyE::PzapA-GFP*	This work
LVG073	Δ*dnmA*, *amyE::PzapA-GFP*	This work
LVG074	*amyE::PrnhC-GFP*	This work
LVG075	Δ*dnmA*, *amyE::PrnhC-GFP*	This work
LVG079	*amyE::PcomEA-GFP*	This work
LVG080	Δ*dnmA*, *amyE::PcomEA-GFP*	This work
LVG081	*amyE::PezrA-GFP*	This work
LVG082	Δ*dnmA*, *amyE::PezrA-GFP*	This work
LVG087	*amyE::PscpA-GFP*	This work
LVG088	Δ*dnmA*, *amyE::PscpA-GFP*	This work
LVG102	*amyE::PscpA mut1-GFP*	This work
LVG103	Δ*dnmA*, *amyE::PscpA mut1-GFP*	This work
LVG105	*amyE::Phbs-GFP*	This work
LVG106	Δ*dnmA*, *amyE::Phbs-GFP*	This work
LVG108	Δ*dnmA operon*, *amyE::PscpA-GFP*	This work
LVG109	Δ*dnmA operon*, *amyE::PscpAmut1-GFP*	This work
LVG118	*amyE::PscpA mut2-GFP*	This work
LVG119	Δ*dnmA operon*, *amyE::PscpAmut2-GFP*	This work

LVG120	*amyE::PscpA mut3-GFP*	This work
LVG121	Δ*dnmA operon, amyE::PscpAmut3-GFP*	This work

Supplementary Table S2. Plasmids used in this study

Plasmid	Vector	Insert	Source
pJS146	pminiMAD	*ydiOP*	
pTN02	pE-SUMO	*dnmA* (*M.BsuPY79I*)	
pTN03	pDR110	*dnmA* (*M.BsuPY79I*)	
pAS2	pE-SUMO	*dnmA (Y465A)* (*M.BsuPY79I*)	
pTN12	pE-SUMO	*yabB*	
pTN13	pE-SUMO	*scoC*	
pLVG1	pDR111_GFP(Sp)	w/o lacI	(5)
pLVG1-0374	pLVG1	P*rsbV*/U0374	
pLVG1-0868	pLVG1	P*wprA*/U0868	
pLVG1-1292	pLVG1	P*yloA*/U1292	
pLVG1-1995	pLVG1	P*comE*/U1995	
pLVG1-2292	pLVG1	P*ezrA*/U2292	
pHP13		None	BGSC (http://www.bgsc.org)

Supplementary Table S3. Oligonucleotides used in this study

Oligo name	Oligo sequence
oAS1	CCAAGAGCCGGTGGATTACCAAAAACATATACTTCTTC
oAS2	CCACCGGCTCTTGGTTCAAAAAAACAAAACAAAGAACATAAATC
oAS9	/5IRD700/GTGCAGCGTATCCGGACAATGACGAGACGGAAACAGAGCTGCTGCTG ATC
oAS10	/5IRD700/GTGCAGCGTATCCGGACAATGACGTGACGGAAACAGAGCTGCTGCTGATC
oAS11	/5IRD700/ATATAAACATACATACATACATTATTATATAAACATACATACATACATTA
oTMN5	GGCTCACCGCGAACAGATTGGAGGTATGGCGCTCATTGATTTAGAAGA TAAAATTGC
oTMN7	TGGTGGTGGTGGTGGTGCTCGACTACCGTTCTGTCATTTCTTGATACAA TTTAAGCAATAC
oTMN36	CACCGCGAACAGATTGGAGGTATGGTTTCATTACATGATGATGAAAGATTAGATTA
oTMN37	TGGTGGTGGTGGTGCTCGATTATTTGTCTCCATATAAAATGGTCCTGATTTC
oTMN38	GTGCAGCGTATCCGGACAATGACGAGACGGAAACAGAGCTGCTGCTGATC
oTMN39	GATCAGCAGCAGCTCTGTTTCCGTCTCGTCATTGTCCGGATACGCTGCAC
oTMN40	GTGCAGCGTATCCGGACAATGACGTGACGGAAACAGAGCTGCTGCTGATC
oTMN41	GATCAGCAGCAGCTCTGTTTCCGTCACGTCATTGTCCGGATACGCTGCAC
oTMN62	CACCGCGAACAGATTGGAGGTATGAATCGAGTGGAACCGCCCTATG
oTMN63	GGTGGTGGTGGTGGTGCTCGATTAACTGTTTACAGGTTCGAGCTCTTCAG
oTMN67	/5IRD700/CA AAACAGGATATGAAATAGTATTGGACGAGAGCTTTTTGGTGGCTTATACTATAG
oTMN68	/5IRD800/CTATAGTATAAGCCACCAAAAAGCTCTCGTCCAATACTATTTCATATCCTGTTTTG
oTMN70	/5IRD700/CAAAACAGGATATGAAATAGTATTGGACGTGAGCTTTTTGGTGGCTTATACTATAG
oTMN71	/5IRD800/CTATAGTATAAGCCACCAAAAAGCTCACGTCCAATACTATTTCATATCCTGTTTTG
oJR269	TAATGTATGTATGTATGTTTATATAATAATGTATGTATGTATGTTTATAT
oJR270	GUGCAGCGUAUCCGGACAAUGACGAGACGGAAACAGAGCUGCUGCUGAUC
oJR271	GAUCAGCAGCAGCUCUGUUUCCGUCUCGUCAUUGUCCGGAUACGCUGCAC
oLVGLS023A	GCTAGCTGATTAACTAATAAGGAGGACAAAC
oLVGLS023B	GAGAGTCGAATTCCTGCAGC
oLVGLS024A	CCGGGATCCGATGACCTCGTTTCCACCGAATTAGC
oLVGLS024B	CCGGGATCCGCAGGCCATGTCTGCCCGTATTTC
oLVGLS034	CGTATCACGAGGCCCTTTCG
oLVGLS042A	GAAATAGTATTGGACGTGAGCTTTTTGGTGGCTTATAC
oLVGLS042B	GCCACCAAAAAGCTCACGTCCAATACTATTTCATATCCTG
oLVGLS044A	GAAATAGTATTGGACGCGAGCTTTTTGGTGGCTTATAC
oLVGLS044B	GCCACCAAAAAGCTCGCGTCCAATACTATTTCATATCCTG
oLVGLS045A	GAAATAGTATTGGACGACAGCTTTTTGGTGGCTTATAC
oLVGLS045B	GCCACCAAAAAGCTGTCGTCCAATACTATTTCATATCCTG
oKWJ89	TTCTTCGCTTGGCTGAAAAT
oKWJ90	CACCAGGTTTTTGGTTTGCT
oLVG52A	5'Biotin-CTGCAGGAATTCGACTCT
oLVG52B	5'-Biotin-CCTTATTAGTTAATCAGCTAGC
oLVG_U0374F	CGCCATTCGCCAGGGCTGCAGGAATTCGACTCTCCCTGATCTGCAGAAGCTCATTG
oLVG_U0374R	CATGTTTGTCCTCCTTATTAGTTAATCAGCTAGCCTTCAAATCACTAGTTGCTTTATAC

oLVG_U0868F	CGCCATTCGCCAGGGCTGCAGGAATTCGACTCTCGGTCTGCATTTGCCAATTG
oLVG_U0868R	CATGTTTGTCCTCCTTATTAGTTAATCAGCTAGCAAAATAATGAATCTCCTTGAAGG
oLVG_U1292F	CGCCATTCGCCAGGGCTGCAGGAATTCGACTCTCGTTAATCCTTGTTTCATGGACG
oLVG_U1292R	CATGTTTGTCCTCCTTATTAGTTAATCAGCTAGCTCTCATTCTTCCTGCATTCGAT
oLVG_U1995F	CGCCATTCGCCAGGGCTGCAGGAATTCGACTCTCGCGTGACAGCTGATTTTACGG
oLVG_U1995R	CATGTTTGTCCTCCTTATTAGTTAATCAGCTAGCCGCAGTGAAAAAGCAGTTTC
oLVG_U2292F	CGCCATTCGCCAGGGCTGCAGGAATTCGACTCTCCGGAAGTATTGAAGTCGAG
oLVG_U2292R	CATGTTTGTCCTCCTTATTAGTTAATCAGCTAGCCGGAGTATCTATTCTTCCATTG
oLVG_U1780F	CGCCATTCGCCAGGGCTGCAGGAATTCGACTCTCGAATCATAAACGAAGGCTCTGG
oLVG_U1780R	CATGTTTGTCCTCCTTATTAGTTAATCAGCTAGCGTAGAGTAACACATATAAAAAGCCAT
oLVG_U1815F	CGCCATTCGCCAGGGCTGCAGGAATTCGACTCTCCTCTAGTGCTTCTTAGAAAGG
oLVG_U1815R	CATGTTTGTCCTCCTTATTAGTTAATCAGCTAGCTCACTCTCATTGCCGGAAAAAC
oLVG_U2212F	CGCCATTCGCCAGGGCTGCAGGAATTCGACTCTCGTAAGTGAACCGCTGTACG
oLVG_U2212R	CATGTTTGTCCTCCTTATTAGTTAATCAGCTAGCATTCCGCGAGAATCCTAG
oLVG_U2213F	CGCCATTCGCCAGGGCTGCAGGAATTCGACTCTCGTGAAGTGCTGGCCGTAAATG
oLVG_U2213R	CATGTTTGTCCTCCTTATTAGTTAATCAGCTAGCACTTTTCGCTGTATATACCAGTG

Oligo sequences in red represent RNA. /5IRD700/ indicates 5' infrared dye label with excitation at 700 nm.

Supplementary Table S4. Modified motifs detected in *B. subtilis* by PacBio SMRT sequencing.

Motif	Type	%Detected	Mean QV	Mean Cov.	Mean IPD Ratio
**WT PY79**					
GACG**A**G	m6A	99.7	388	286	6.72
V**T**TCGAGNR	NA	79.2	75	284	1.90
**C**TCGARB	m5C*	70.8	74	270	1.89
V**T**TVGAGNBY	NA	40.1	55	283	1.67
**G**GNB	NA	5.6	41	290	1.67
**WT 3610**					
GACG**A**G	m6A	94.7	362	313	4.84
R**A**WKYAGYA	m6A	28.7	98	309	1.66
D**T**NRADDDG	NA	22.8	61	305	1.78
D**T**WTWGAAG	NA	21.4	57	327	1.71
**A**GCNMAAAWH	m6A	15.8	107	322	1.53
**T**NNNDNNH	NA	12.6	61	303	1.78
D**T**SNVCNTWNH	NA	11.7	58	304	1.75
**T**WGCNNNG	NA	10.6	58	313	1.75
**T**NRGCYNH	NA	10.1	56	309	1.72
**T**NNNCRVH	NA	9.6	58	304	1.76
**T**SNNNNNG	NA	6.1	57	305	1.75
**A**GDNNNNW	m6A	4.3	104	325	1.71

*Modification type confirmed via methylation sensitive restriction endonuclease digest.

The motifs shown in this table are comprehensive to those presented in Table 1 in the main text.

Supplementary Table S5. Cells with Δ*dnmA* are wild type for mutation rate

Strain	No. of cultures	Mutations per culture	Mutation rate (Mutations per generation 10^{-8} $\pm$ [95%CI]	Relative mutation rate
Wild Type	20	0.60	1.7 [0.95-1.68]	1
Δ*dnmA*	22	0.72	1.8 [0.94-2.2]	1.05
Δ*mutSL*	22	41.3	120.8 [109-131]	71.1

Mutagenesis assays were done as described using rif^R as an indicator. Mutation rate and mutations per culture were calculated using the Ma-Sandri-Sarkar Maximum Likelihood Estimator with the web-based tool FALCOR (2). Data and calculations supporting this table are publicly available https://figshare.com/articles/Nye_et_al_DnmA_methyltransferase/8070995.

Supplementary Table S6. Modified motifs detected in *B. subtilis* by PacBio SMRT sequencing.

Motif	Type	%Detected	Mean QV	Mean Cov.	Mean IPD Ratio
***ΔdnmA* WT PY79**					
V**T**TCGAGNR	NA	62.5	53	118	2.00
**C**TCGARB	m5C*	46.7	51	120	2.00
***ΔdnmA* WT 3610**					
V**A**TATRGCA	m6A	54.0	88	363	2.00
R**A**HKYAGYA	m6A	31.0	110	357	1.67
DD**T**NRGCNTHNH	NA	20.4	60	356	1.72
DNND**T**GYAADNG	NA	20.3	65	348	1.81
D**T**NRVDDDG	NA	15.2	61	355	1.73
**T**NNNDNNH	NA	12.3	62	353	1.74
**T**NNNCRVH	NA	9.4	60	359	1.72
**A**GNNMRNA	m6A	9.1	109	359	1.55
**T**NNSCBDH	NA	7.2	58	363	1.68
**T**SNNBNNG	NA	6.4	58	361	1.71
**A**GNNDNNW	m6A	3.4	101	365	1.54
**A**NDNNNNH	m6A	0.8	98	367	1.53
***ΔdnmA, amyE::Pspac dnmA* PY79**					
GACG**A**G	m6A	99.7	213	152	6.32
V**T**TCGAGNR	NA	67.2	59	146	1.98
**C**TCGARB	m5C	52.7	59	149	2.00
MNGACG**A**WCC	NA	47.3	58	152	2.20
V**T**TCGAGBB	NA	38.1	53	157	1.82
WAGACG**A**WB	NA	21.7	53	148	2.19
**G**GNNB	NA	6.6	40	168	1.86

*Modification type confirmed via methylation sensitive restriction endonuclease treatment.

The motifs shown in this table are comprehensive to those presented in Table 2 in the main text.

Supplementary Table S7. PacBio SMRT sequencing at GACGAG motifs in WT PY79.

Please see attached excel file providing information on all GACGAG motifs in PY79.

Supplementary Table S8. PacBio SMRT sequencing at GACGAG motifs in Δ*dnmA* in PY79. Please see attached excel file providing information on all GACGAG motifs in PY79 Δ*dnmA*.

Supplementary Table S9. Modified motifs detected in *B. subtilis* by PacBio SMRT sequencing.

Motif	Type	%Detected	Mean QV	Mean Cov.	Mean IPD Ratio
ΔyabB					
GACG**A**G	m6A	96.9	304	240	5.15
**A**TATRGCA	m6A	74.0	80	238	2.09
**A**DGYACYTV	m6A	34.7	85	238	2.01
**A**DKYASYA	m6A	29.8	88	238	1.88
**A**GCNAAAAWH	m6A	17.3	95	237	1.58
G**A**NNBNRCA	m6A	13.7	98	241	1.90
**T**NNNNNNH	NA	12.2	46	234	1.69
D**T**VVVNNDG	NA	11.1	44	234	1.67
**A**NVBANYW	m6A	6.4	78	238	1.81
**A**GDNVDNW	m6A	5.4	87	236	1.81
**T**BNNDNNG	NA	5.2	43	235	1.67
**A**GBB	m6A	2.7	99	238	2.02
ΔdnmA ΔyabB					
V**T**TCGAGNR	NA	71.9	60	166	1.92
**C**TCGARB	NA	57.3	57	168	1.91
V**T**TCGAGBY	NA	33.2	46	181	1.70
**G**G	NA	3.1	39	185	1.77

Supplementary Table S10. Identification of protein species in DnmA protein purification

Identified Protein Accession Number	Molecular Weight	Total Spectrum Count
YEEA_BACSU	101 kDa	190
SMT3_YEAST	12 kDa	42
K2C1_HUMAN	66 kDa	34
HORN_HUMAN	282 kDa	34
TRYP_PIG	24 kDa	18
K1C10_HUMAN	59 kDa	19
K22E_HUMAN	65 kDa	21
K1C9_HUMAN	62 kDa	15
K2C5_HUMAN	62 kDa	4
CYTA_HUMAN	11 kDa	3
K1C14_HUMAN	52 kDa	8
ALBU_HUMAN	69 kDa	2
ARGI1_HUMAN	35 kDa	3
ANXA2_BOVIN (+8)	39 kDa	2
FABP5_HUMAN	15 kDa	2
SBSN_HUMAN	61 kDa	2

Mass spectrometry was completed by the University of Michigan Core on the high molecular weight species, confirming the presence of DnmA (YeeA). The SMT3_Yeast contaminant is likely the result of trace SUMO-tagged DnmA from the protein purification process (see Materials and Methods).

Supplementary Table S11. Promoter upshifts containing the m6A motif

Upshift	Sequence context	Downstream TU
U374	cgatgattttacgttaattgttttgcggagaaaGGTTTAAcgtctgtcagacgaGGGTATAAAGCAACTAGTGatttgaaggaaaatttgaggtgatacga	**rsbV, rsbW, sigB, rsbX**
U868	atattccaaatcatttaaaataaccttaaaattccctgtaagcggtatctcgtcctatgaaatTATGATACCTTCAAGGAGATtcattattttgcaggagg	**wprA**
U1292	gctctcttatagagatatcactctataagcatgcttaTTCTGActcgtcccatttCATGCTATAATTATCGAATGcaggaagaatgagagggtgtattgca	**yloA**
U1780	aaaaaaggaatattcgttcggtaaatcaccttaaatcCTTGACgagcaagggattgacgCTTTAAAATGCTTGATATGGctttttatatgtgttactctac	**S861, hbs**
U1815	ctgtacaaactccttcaaaacaggatatgaaatagTATTGGACgagagctttttggTGGCTTATACTATAGGGTAGccagtttttccggcaatgagagtga	**scpA, spcB, ypuI**
U1995	taaaacgatggtttttaaaatgctttttatgCTTTTGCAgtacagacgaacgTATGACATACTCGTCTACACatgaaactgctttttcactgcggaaat	**S963, comEA-EC, S962, holA**
U2212	tgtatataccagtgtatcataacagcgggaggctcgtcTTTCCATTcatttaataaaCGTGTTATGATAAGAACTAGgattctcgcggaatggaggagaaa	**yshA (zapA), yshB, S1080, polX, mutSB**
U2213	gttcttatcataacacgtttattaaatgaatggaaagacgagcctcccgctgtTATGATACACTGGTATATACagcgaaaagtgtaaaaaaaggagattat	**rnhC**
U2292	aatttttggacgagtgtgatgtgaccgactcttttTGGCTTATaaacgccgagaGATGCTACAATGGAAGAATAgatactccggtaatattgttcatatac	**S1127, S1125, ezrA**
U2511	acggatgggcacgttagatccttacgatttttttctgctgacTCGTCAATttgaacggcaaTATGGTATAATTAATAACAAttttcatttaggaggcaattt	**yumC**
U624	cactaaagttgatcaaatgacctaagtgcgccaaacgtGTTACGggacgagctatctCATGGTATAAATGGAATTGTaaacgttatcaaggaggtcgtcat	**malA, malR-Q**
U792	atattcagctcagtccttttgatgcgtctttcccgCACATAactcgtctcattcccGCATATGGTTAAGAATAAAgaatctatgcaaaggggggaggagcc	**yhaL**
U1042	agcggtctgaggctttttattagataaaagacctaattgttctaTGAAACTTTCtgacgagatatCCGTATATCATACAGAttgacatcattcacatcaga	**S462, S461**
U1293	tacaccctctcattcttcctgcattcgataattatAGCATGaaatgggacgagtcaGAATAAGCATGCTTATAGAgtgatatctctataagagagcagggg	**yloB, S571, yloC, ylzA, gmk, rpoZ**
U1406	atgtgttcataaaaaactaaaaaaaaTATTGAAAatactgacgaggttATATAAGATGAAAATAAGTTagtttgtttaaacaacaaactaataggtgatgt	**S634, xylA, S635, xylB, S636**
U1861	ctttcccgtcatataaactgctcaaatgaaccgcttttgtcaaacaTTTCTGTATAATAGACGAGAaatcagtttatttttcagtatagaagcatttttat	**yqjN**
U2116	atcaagggaataaagatcgtttttatgacgagccaaaagggtgaccatAACATAtcaagcataaaaaagACATAGACTGTTAACAGAAccagcaaacaaat	**S1041, glnQ, glnH, glnM, glnP**
U2235	gtgtattggctttgcggaaaaaagggtggaacCacgattccgtttattcaaCCTCGTCCCTttcatagggggcggggtttttatatgcaaaaaaaggagtg	**thrS**
U2676	cttactttaaaaagccacgcaacacggttctCGtcacagacgaaggagccgcAaagaagttattaagggatgaataatccctcaatataaatatctctcac	**gapA, S1301, S1300, pgk, tpiA, pgm, eno**
U3059	gcgaaacacacatgttctccactaaaaaagAGTATAtccggtatagatagaCGAGAAACTGAAAGGGAAAcctcattcgtttacatattggcttcagcgga	**aldY**
U75	cctgatttccaaatcatttgcggatcgcctgaacTGCttgtcagcaaaaagggcAAGCTATTAGAGACGAGACcgattgcgggcacccgttccagagggaa	**secondary internal pabB upshift**
U568	aaaagagtatctcgtatagaacataagaagaatgacgagtttttttaAGATTAtcatcaattatgtgaGAATAAAATATTATAAGGGaaaatgaaggctgtc	**S257 - as-fecF**
U797	ctatttttctttttttgcagatgattgGCAGGACATggatgactttaTGTACAAATAAAATAACGAcgagcaaaccgccgaataaagatacatattcatcg	**as-yhaJ; secondary yhal upshift**
U809	tttgctgcggaagacatatacagataaagaacagccATAATGacatagacgagaagcGCATACATATAAACAGATTggaaaacaaaataaagaacgagtgt	**as-ecsB (ecsB internal)**
U895	aagaaggtccgtattaatttttcccaCTCATAAaccttactttacccCACCATACTATTgaagacgactcatctcgtcaaagtatggaagggggcagtctc	**S397, yizC**
U911	tcctgagaaggttttaacaaatcatgatcttgaaaaaatggtTGAAACTTCTgacgagtggatTCGTACAAGAACAGGAatagaagaaagaagaatcgcag	**secondary internal fabHA upshift**
U936	ataaaacattctaaaggcggtgtttccgacggcttcggctcgtcgccaAGCATCtcaaataaaatttctTCATATACATCATATGAGTagctgccaggaac	**S416 - as-spxH**
U1010	cgggtagccgaccttcggattatcaAACTTGACgagcagcggcatcACGGCCACACTTGTGATAAAcaaggcgccaagtgatccgatttggcggaacgtcg	**as-yjmB**
U1122	gttcttaacggttatatgaaccaattcattcgaaaagacaCCTTTACAaacatacgttcgttaAATTATAATAAAACAGACGAgctgccatccagcatccc	**S498; independent transcript**
U1389	ttgttgttgatattcttgaataaaaaaacccggtttctCGCGAtgaggagccgggttttttttatGAGACGCTCGTCCCCGTCtcggctatgattctaggat	**S623 - as-cwlC**
U1429	gaatgtaggtctctttgaggctttagcacgaattctctcgtcctccttgttaaatttttgttaaattcaCAATATTATAtaccattagcccgggcgctgtt	**S653 - secondary internal surA upshift**
U1984	aaatattttattcaaagtcagccagtcgacgagTATTTAAacgcactcgaacaGGAAATGATAAAtacaatagcaaaaaccgggcagcctgatctcaaaac	**secondary hrcA-grpE-dnaK upshift**
U2466	tcatttcttttggcgttacgtcattcatcctcgtcatggcctttcgcgcttatcaggaattgaaatcggacgatatggatcaaatgaggggaaatgatcaa	**secondary mrpD-G upshift**
U1405	ttcatcttatataacctcgtcagtattttcaatattttttTTAGttttttatgaacacATTAGATATAATAAAGGGAAgattcgctatgtactatgttgat	**S633, xylR**
U1996	ttttacctgacgagtttgtaaaaaatatttttcatattACACctgagaaattaaaggAACGAAATGTAAAAGGAAttattactgacctggataatacgctt	**yqeH, aroD, yqeI, nadD, yqeK, yqeL, yqeM**
U2030	gagattttggatatagacgaggctcttggttcacGAATTCACCAgatgtgccgtgatTACATAGTGATTATTAGAGGcgatcgaatgcaattaaatcatag	**yqzO, yqaN, S982, yqaO, S980**
U2255	cctcctctcgtcagcatgtcctattttttatatgTATTCACGCtgcggctgaataTGAATACATTCATCTTAAAGgagggatggcatgtgtttacacaagc	**ytwI**
U2486	cggaggcaggatgacgagccacagccctctgttGGTTTGAtcgctctcctgagaGGGAAAACTGAAAGAAACGcggtcatccggcagatcgtataccatcc	**S1225; as-yukBC**
U3058	ttcagtttctcgtctatctataccggatatactctttttTAGTGGAGaacatgtgtgtttCGCCTTATACTGAATATACAgatccttacataagagaggag	**yxkF, S1490, msmX**
U3138	agacgaggtttcttataagcctttTTCATccttttcCCTCCTTCCTTGTAAaaaaataggctatcacgcacaactaaatattataatcctctgataattct	**yxbC**

U3060	agatagacgagaaactgaaagggaaacctcattCGTTTACatattggcttcagcGGAAATAGAAGAAGACATGcaggaccaaaggagggtcatcttatgag	**aldY**
U118	tttgaaaataaaaaatttaattttcctCTTTACAAacagggggtgacctGTATATAATAACTTTTGTCAgctcgacgagaacacaacggcccgttggtcaa	**trnSL-Glu2, -Val1,-Thr1, -Tyr1, -Gln2, S67**
U301	cagcagcgatcgcggcctatgcgaatcaaaacGGATTACttttgctgacagcGGGAATTAACGGTAATATCatccgctttttgacaccgctcgtcatctca	**secondary gabD upshift**
U902	atcagactcttttgtcacctcactttctgctaaaattggaTTCCCCCTtcgctttttgTATGGTATGATAACTTTTAGaatagaatgagaaggacgaggtg	**yjzD, S399**
U934	acaacctctatgcttaatgttcatatttttgtcacaaAACATAacgaagtgcattcacTCATATCCTTATAAGGAAAaaggacgaggaggaccgccatgat	**yizD**
U1428	agcttacttttcataaattcaaaaatgagaagAACAGCGCCCgggctaatggtatATAATATTGTGAATTTAACAaaaatttaacaaggaggacgagagaa	**S654, yndL, S657, fosB, S658, S659**
U1534	tatagatcagaacaaaagttcgatgtaaatgttggtaataaaatataaaggtcaataatgaTATCCGTAGTATTAATAAAGgagagattcttttcgacgag	**yozM**
U1794	atttcgagcttttcacttgaactgaaaaaGGTTTGatgcgtcgggtaattGTAAATACTGTTAACGACAttgcctttttcgatgacgagggcatcaatgcc	**S869 - sporulation sRNA - as-ypdA**
U1806	taaggctcttttttagttgctattcatataatagaaATTTtcaaaaaaaagttgTACGTGTATAATAAAACAAGgtaaagattgaaaggatttgagacgag	**aroC**
U1860	cagcaattcgtccttcgtctgccaattcattgattccgcccctcctaTGAATAAaaatgcttctataCTGAAAAATAAActgatttctcgtctattataca	**yqjM**
U2140	tgtaagaaaaaccgattgcatttcacaaagcttttaCGTCTAattcatgggataaggGAATACATTTTTACAAAGAcgagccatcagcatgtctgacggtt	**yrzE**
U2221	aattgttaaaatgcgtgatatttcatcagtattCCTCGGAGCAatcacggcatcaGGCATAGACTGATACTGAGGcgtcgcatcatatgaatagacgagac	**S1083; as-ysfB**
U2256	aaacacatgccatccctcctttaagatgaatgtaTTCATAttcagccgcagcgtGAATACATATAAAAAATAGgacatgctgacgagaggaggaccgtttt	**ytvI**
U2654	tgtttctctttctatattttatcaatcacgctTGCATGCCctccctcgttattTGCGTTATAATAGTGACAGAcgaggtgaaaagtatgaaccaatcagaa	**yvaP, S1288, yvaQ**
U3003	tacggacaattcagagcatattggctcttcattgcgCCTTTtctttcaatatttgaTGCGTTAAAATGGTAACCGTgtgaaaagatgctagacgaggaaaa	**S1473, S1472, S1471, qoxA-D**
U3203	gaaggtggtcttcaaggaaaaaacgagcaggtgctcgaacagatagagcaggaaatgctagcttcggggcttgatatagaggaacaggacgaggagaaggt	**internal yzzl upshift**

Subset of transcribed regions 5' of ORFs identified in Nicholas *et al.* (26) that contain the m6A motif (indicated in red). Capitalized letters and underscores indicate predicted sigma factor binding sites. Downstream transcriptional units are listed.

References

1. Bolz, N.J., Lenhart, J.S., Weindorf, S.C. and Simmons, L.A. (2012) Residues in the N-terminal domain of MutL required for mismatch repair in *Bacillus subtilis*. *Journal of Bacteriology*, **194**, 5361-5367.

2. Hall, B.M., Ma, C.X., Liang, P. and Singh, K.K. (2009) Fluctuation analysis CalculatOR: a web tool for the determination of mutation rate using Luria-Delbruck fluctuation analysis. *Bioinformatics*, **25**, 1564-1565.

3. Lenhart, J.S., Brandes, E.R., Schroeder, J.W., Sorenson, R.J., Showalter, H.D. and Simmons, L.A. (2014) RecO and RecR are necessary for RecA loading in response to DNA damage and replication fork stress. *J Bacteriol*, **196**, 2851-2860.

4. Gibson, D.G., Young, L., Chuang, R.Y., Venter, J.C., Hutchison, C.A., 3rd and Smith, H.O. (2009) Enzymatic assembly of DNA molecules up to several hundred kilobases. *Nat Methods*, **6**, 343-345.

5. Overkamp, W., Beilharz, K., Detert Oude Weme, R., Solopova, A., Karsens, H., Kovacs, A., Kok, J., Kuipers, O.P. and Veening, J.W. (2013) Benchmarking various green fluorescent protein variants in *Bacillus subtilis*, *Streptococcus pneumoniae*, and *Lactococcus lactis* for live cell imaging. *Appl Environ Microbiol*, **79**, 6481-6490.

6. Ohshima, H., Matsuoka, S., Asai, K. and Sadaie, Y. (2002) Molecular organization of intrinsic restriction and modification genes BsuM of *Bacillus subtilis* Marburg. *J Bacteriol*, **184**, 381-389.

7. Han, J.S., Kang, S., Kim, S.H., Ko, M.J. and Hwang, D.S. (2004) Binding of SeqA protein to hemi-methylated GATC sequences enhances their interaction and aggregation properties. *J Biol Chem*, **279**, 30236-30243.

8. Lu, M., Campbell, J.L., Boye, E. and Kleckner, N. (1994) SeqA: a negative modulator of replication initiation in *E. coli*. *Cell*, **77**, 413-426.

9. Nievera, C., Torgue, J.J., Grimwade, J.E. and Leonard, A.C. (2006) SeqA blocking of DnaA-oriC interactions ensures staged assembly of the *E. coli* pre-RC. *Mol Cell*, **24**, 581-592.

10. Bale, A., d'Alarcao, M. and Marinus, M.G. (1979) Characterization of DNA adenine methylation mutants of *Escherichia coli* K12. *Mutat Res*, **59**, 157-165.

11. Pukkila, P.J., Peterson, J., Herman, G., Modrich, P. and Meselson, M. (1983) Effects of high levels of DNA adenine methylation on methyl-directed mismatch repair in *Escherichia coli*. *Genetics*, **104**, 571-582.

12. Casadesus, J. and Low, D. (2006) Epigenetic gene regulation in the bacterial world. *Microbiol Mol Biol Rev*, **70**, 830-856.

13. Lee, P.S., Lin, D.C., Moriya, S. and Grossman, A.D. (2003) Effects of the chromosome partitioning protein Spo0J (ParB) on *oriC* positioning and replication initiation in *Bacillus subtilis*. *J Bacteriol*, **185**, 1326-1337.

14. Dupes, N.M., Walsh, B.W., Klocko, A.D., Lenhart, J.S., Peterson, H.L., Gessert, D.A., Pavlick, C.E. and Simmons, L.A. (2010) Mutations in the *Bacillus subtilis* beta clamp that separate its roles in DNA replication from mismatch repair. *J Bacteriol*, **192**, 3452-3463.

15. Noirot-Gros, M.F., Velten, M., Yoshimura, M., McGovern, S., Morimoto, T., Ehrlich, S.D., Ogasawara, N., Polard, P. and Noirot, P. (2006) Functional dissection of YabA, a negative regulator of DNA replication initiation in *Bacillus subtilis*. *Proc Natl Acad Sci U S A*, **103**, 2368-2373.

16. Marinus, M.G. and Morris, N.R. (1974) Biological function for 6-methyladenine residues in the DNA of Escherichia coli K12. *J Mol Biol*, **85**, 309-322.

17. Herman, G.E. and Modrich, P. (1981) *Escherichia coli* K-12 clones that overproduce dam methylase are hypermutable. *J Bacteriol*, **145**, 644-646.

18. Cooper, L.A., Simmons, L.A. and Mobley, H.L. (2012) Involvement of Mismatch Repair in the Reciprocal Control of Motility and Adherence of Uropathogenic *Escherichia coli*. *Infect Immun*, **80**, 1969-1979.

19. Lenhart, J.S., Sharma, A., Hingorani, M.M. and Simmons, L.A. (2013) DnaN clamp zones provide a platform for spatiotemporal coupling of mismatch detection to DNA replication. *Molecular microbiology*, **87**, 553-568.

20. Lenhart, J.S., Pillon, M.C., Guarne, A., Biteen, J.S. and Simmons, L.A. (2015) Mismatch repair in Gram-positive bacteria. *Res Microbiol*. 167(1):4-12.

21. Lenhart, J.S., Schroeder, J.W., Walsh, B.W. and Simmons, L.A. (2012) DNA Repair and Genome Maintenance in *Bacillus subtilis*. *Microbiology and molecular biology reviews : MMBR*, **76**, 530-564.

22. Yeeles, J.T., Gwynn, E.J., Webb, M.R. and Dillingham, M.S. (2011) The AddAB helicase-nuclease catalyses rapid and processive DNA unwinding using a single Superfamily 1A motor domain. *Nucleic Acids Res*, **39**, 2271-2285.

23. Youngman, P., Perkins, J.B. and Losick, R. (1984) Construction of a cloning site near one end of Tn917 into which foreign DNA may be inserted without affecting transposition in *Bacillus subtilis* or expression of the transposon-borne erm gene. *Plasmid*, **12**, 1-9.

24. Konkol, M.A., Blair, K.M. and Kearns, D.B. (2013) Plasmid-encoded ComI inhibits competence in the ancestral 3610 strain of *Bacillus subtilis*. *J Bacteriol*, **195**, 4085-4093.

25. Smith, B.T., Grossman, A.D. and Walker, G.C. (2001) Visualization of mismatch repair in bacterial cells. *Mol. Cell*, **8**, 1197-1206.

26. Nicolas, P., Mader, U., Dervyn, E., Rochat, T., Leduc, A., Pigeonneau, N., Bidnenko, E., Marchadier, E., Hoebeke, M., Aymerich, S. *et al.* (2012) Condition-dependent transcriptome reveals high-level regulatory architecture in *Bacillus subtilis*. *Science*, **335**, 1103-1106.
