# Supplementary material for: Methyltransferase DnmA is responsible for genome-wide N6-methyladenosine modifications at non-palindromic recognition sites in Bacillus subtilis
Source: Nucleic Acids Res. 2020 Apr 23;48(10):5332–48. doi: 10.1093/nar/gkaa266 (PMC7261158; doi:10.1093/nar/gkaa266)
Supplement: gkaa266_Supplemental_Files [file gkaa266_supplemental_files.zip › Table S7_dnmA-.docx]

**Table S7. PacBio SMRT sequencing at GACGAG motifs in WT PY79.**

| **position** | **strand** | **motif** | **base** | **ModQV** | **IPDRatio** | **coverage** | **frac** | **CI low** | **CI high** |
| --- | --- | --- | --- | --- | --- | --- | --- | --- | --- |
| 3690 | - | GACGAG | A | 423 | 9.876 | 258 | 1 | 1 | 1 |
| 13073 | - | GACGAG | A | 440 | 8.228 | 312 | 0.978 | 0.915 | 1 |
| 20347 | + | GACGAG | A | 563 | 8.23 | 393 | 1 | 0.956 | 1 |
| 20813 | - | GACGAG | A | 437 | 4.754 | 387 | 0.936 | 0.865 | 1 |
| 21392 | - | GACGAG | A | 550 | 7.235 | 379 | 0.984 | 0.934 | 1 |
| 33541 | - | GACGAG | A | 595 | 8.33 | 381 | 0.985 | 0.954 | 1 |
| 36413 | + | GACGAG | A | 465 | 6.587 | 352 | 0.951 | 0.876 | 1 |
| 39739 | - | GACGAG | A | 236 | 3.728 | 395 | 0.976 | 0.882 | 1 |
| 44114 | - | GACGAG | A | 604 | 8.646 | 382 | 1 | 0.977 | 1 |
| 45584 | - | GACGAG | A | 497 | 5.678 | 372 | 0.958 | 0.892 | 1 |
| 45793 | + | GACGAG | A | 593 | 8.943 | 388 | 0.985 | 0.939 | 1 |
| 50231 | + | GACGAG | A | 448 | 5.81 | 349 | 0.975 | 0.9 | 1 |
| 52923 | + | GACGAG | A | 486 | 5.613 | 376 | 0.959 | 0.885 | 1 |
| 63092 | - | GACGAG | A | 587 | 7.882 | 392 | 1 | 0.946 | 1 |
| 64617 | - | GACGAG | A | 558 | 8.301 | 387 | 1 | 0.978 | 1 |
| 73482 | + | GACGAG | A | 641 | 7.272 | 424 | 0.948 | 0.89 | 1 |
| 73830 | + | GACGAG | A | 547 | 6.338 | 410 | 0.985 | 0.944 | 1 |
| 76096 | + | GACGAG | A | 513 | 5.853 | 413 | 0.918 | 0.848 | 0.984 |
| 78129 | - | GACGAG | A | 628 | 7.866 | 418 | 0.985 | 0.948 | 1 |
| 83701 | + | GACGAG | A | 469 | 5.73 | 350 | 0.946 | 0.878 | 1 |
| 83750 | + | GACGAG | A | 408 | 5.48 | 353 | 0.935 | 0.855 | 1 |
| 86284 | + | GACGAG | A | 478 | 7.598 | 337 | 0.967 | 0.901 | 1 |
| 88555 | + | GACGAG | A | 384 | 5.055 | 381 | 0.985 | 0.965 | 1 |
| 93616 | - | GACGAG | A | 405 | 8.972 | 277 | 1 | 0.945 | 1 |
| 99471 | - | GACGAG | A | 388 | 9.271 | 261 | 1 | 0.946 | 1 |
| 109428 | - | GACGAG | A | 532 | 8.065 | 372 | 0.949 | 0.892 | 1 |
| 109695 | - | GACGAG | A | 535 | 7.494 | 361 | 1 | 0.968 | 1 |
| 115576 | + | GACGAG | A | 543 | 8.547 | 346 | 0.943 | 0.891 | 1 |
| 116658 | + | GACGAG | A | 532 | 7.894 | 350 | 0.968 | 0.901 | 1 |
| 117114 | - | GACGAG | A | 442 | 4.843 | 344 | 0.911 | 0.833 | 0.98 |
| 123652 | - | GACGAG | A | 464 | 9.959 | 306 | 1 | 0.969 | 1 |
| 129028 | + | GACGAG | A | 469 | 8.222 | 333 | 0.923 | 0.856 | 0.984 |
| 130480 | - | GACGAG | A | 465 | 8.996 | 300 | 0.978 | 0.923 | 1 |
| 131396 | + | GACGAG | A | 324 | 4.385 | 292 | 0.878 | 0.789 | 0.959 |
| 133103 | - | GACGAG | A | 354 | 6.452 | 255 | 0.974 | 0.888 | 1 |
| 138303 | - | GACGAG | A | 401 | 7.252 | 251 | 1 | 0.962 | 1 |
| 139198 | - | GACGAG | A | 427 | 6.439 | 294 | 1 | 0.938 | 1 |
| 140341 | - | GACGAG | A | 419 | 5.103 | 325 | 1 | 1 | 1 |
| 142560 | - | GACGAG | A | 391 | 4.672 | 374 | 0.986 | 0.92 | 1 |
| 143000 | - | GACGAG | A | 563 | 8.981 | 376 | 0.979 | 0.929 | 1 |
| 147262 | + | GACGAG | A | 483 | 5.278 | 397 | 0.984 | 0.919 | 1 |
| 150508 | + | GACGAG | A | 567 | 8.477 | 355 | 1 | 0.957 | 1 |
| 154659 | - | GACGAG | A | 435 | 9.16 | 263 | 1 | 0.985 | 1 |
| 157813 | - | GACGAG | A | 361 | 6.965 | 246 | 1 | 0.94 | 1 |
| 163972 | - | GACGAG | A | 464 | 9.831 | 278 | 1 | 1 | 1 |
| 169581 | - | GACGAG | A | 753 | 8.491 | 473 | 1 | 0.964 | 1 |
| 174583 | - | GACGAG | A | 491 | 9.122 | 353 | 1 | 0.955 | 1 |
| 181545 | + | GACGAG | A | 420 | 6.279 | 307 | 1 | 0.929 | 1 |
| 188147 | + | GACGAG | A | 506 | 6.06 | 373 | 1 | 0.945 | 1 |
| 190264 | + | GACGAG | A | 471 | 5.377 | 389 | 0.916 | 0.85 | 0.985 |
| 190648 | + | GACGAG | A | 574 | 7.255 | 416 | 0.945 | 0.89 | 1 |
| 194200 | + | GACGAG | A | 551 | 6.387 | 408 | 0.999 | 0.944 | 1 |
| 196213 | + | GACGAG | A | 489 | 5.665 | 396 | 0.945 | 0.884 | 1 |
| 199751 | - | GACGAG | A | 510 | 6.133 | 397 | 0.985 | 0.935 | 1 |
| 200073 | + | GACGAG | A | 559 | 7.362 | 385 | 1 | 0.985 | 1 |
| 201564 | - | GACGAG | A | 394 | 4.726 | 381 | 0.978 | 0.903 | 1 |
| 206814 | - | GACGAG | A | 543 | 8.58 | 367 | 1 | 0.946 | 1 |
| 216823 | - | GACGAG | A | 416 | 5.383 | 315 | 0.985 | 0.924 | 1 |
| 220650 | + | GACGAG | A | 372 | 5.61 | 302 | 0.937 | 0.857 | 1 |
| 230504 | - | GACGAG | A | 381 | 6.089 | 305 | 1 | 1 | 1 |
| 230668 | - | GACGAG | A | 418 | 6 | 307 | 0.917 | 0.841 | 0.985 |
| 236779 | - | GACGAG | A | 323 | 8.801 | 227 | 0.985 | 0.938 | 1 |
| 238946 | + | GACGAG | A | 401 | 7.592 | 254 | 1 | 0.936 | 1 |
| 242685 | + | GACGAG | A | 508 | 7.777 | 320 | 1 | 0.979 | 1 |
| 244718 | - | GACGAG | A | 512 | 7.508 | 359 | 1 | 0.953 | 1 |
| 245873 | - | GACGAG | A | 474 | 6.338 | 367 | 0.983 | 0.911 | 1 |
| 262966 | + | GACGAG | A | 648 | 10.084 | 409 | 0.985 | 0.957 | 1 |
| 268418 | - | GACGAG | A | 644 | 7.575 | 459 | 1 | 0.984 | 1 |
| 268589 | + | GACGAG | A | 667 | 9.378 | 447 | 0.985 | 0.944 | 1 |
| 271446 | - | GACGAG | A | 742 | 8.862 | 466 | 1 | 1 | 1 |
| 274221 | + | GACGAG | A | 500 | 5.433 | 430 | 0.979 | 0.911 | 1 |
| 277057 | - | GACGAG | A | 544 | 5.402 | 413 | 0.98 | 0.912 | 1 |
| 277900 | - | GACGAG | A | 524 | 6.16 | 404 | 1 | 0.965 | 1 |
| 280244 | + | GACGAG | A | 593 | 7.747 | 392 | 0.977 | 0.929 | 1 |
| 293836 | - | GACGAG | A | 204 | 2.845 | 404 | 0.848 | 0.754 | 0.952 |
| 298091 | - | GACGAG | A | 416 | 5.356 | 384 | 1 | 0.94 | 1 |
| 302183 | - | GACGAG | A | 561 | 8.039 | 390 | 1 | 0.985 | 1 |
| 309359 | - | GACGAG | A | 418 | 5.368 | 345 | 1 | 0.942 | 1 |
| 310037 | + | GACGAG | A | 469 | 8.317 | 323 | 0.985 | 0.93 | 1 |
| 312459 | + | GACGAG | A | 550 | 10.553 | 346 | 1 | 1 | 1 |
| 314343 | - | GACGAG | A | 522 | 7.273 | 333 | 0.956 | 0.894 | 1 |
| 315010 | - | GACGAG | A | 445 | 6.391 | 322 | 0.983 | 0.917 | 1 |
| 318021 | + | GACGAG | A | 447 | 9.185 | 294 | 0.979 | 0.91 | 1 |
| 318279 | + | GACGAG | A | 418 | 6.907 | 290 | 1 | 0.937 | 1 |
| 318577 | - | GACGAG | A | 398 | 6.717 | 278 | 1 | 0.984 | 1 |
| 318802 | - | GACGAG | A | 403 | 9.79 | 280 | 0.958 | 0.898 | 1 |
| 319099 | - | GACGAG | A | 416 | 10.923 | 264 | 1 | 0.961 | 1 |
| 331206 | - | GACGAG | A | 241 | 8.548 | 164 | 0.937 | 0.838 | 1 |
| 336867 | - | GACGAG | A | 358 | 5.221 | 291 | 1 | 0.951 | 1 |
| 338984 | + | GACGAG | A | 510 | 7.556 | 347 | 0.985 | 0.943 | 1 |
| 342214 | + | GACGAG | A | 615 | 6.031 | 429 | 1 | 0.985 | 1 |
| 342344 | - | GACGAG | A | 498 | 4.801 | 438 | 1 | 0.954 | 1 |
| 343048 | + | GACGAG | A | 506 | 5.55 | 414 | 0.985 | 0.934 | 1 |
| 350996 | + | GACGAG | A | 430 | 6.484 | 333 | 0.935 | 0.863 | 1 |
| 352690 | - | GACGAG | A | 477 | 7.022 | 366 | 0.964 | 0.891 | 1 |
| 356863 | + | GACGAG | A | 580 | 9.443 | 376 | 0.983 | 0.932 | 1 |
| 358089 | - | GACGAG | A | 520 | 7.029 | 344 | 0.985 | 0.923 | 1 |
| 359570 | - | GACGAG | A | 500 | 5.87 | 326 | 1 | 1 | 1 |
| 359695 | + | GACGAG | A | 442 | 6.042 | 319 | 0.982 | 0.906 | 1 |
| 361730 | - | GACGAG | A | 481 | 10.246 | 305 | 0.985 | 0.936 | 1 |
| 363400 | - | GACGAG | A | 386 | 5.861 | 293 | 1 | 0.95 | 1 |
| 372559 | + | GACGAG | A | 507 | 7.614 | 316 | 1 | 0.948 | 1 |
| 373764 | - | GACGAG | A | 403 | 6.409 | 325 | 0.914 | 0.838 | 0.978 |
| 375766 | + | GACGAG | A | 255 | 4.375 | 286 | 1 | 0.943 | 1 |
| 382901 | + | GACGAG | A | 415 | 6.474 | 338 | 0.901 | 0.825 | 0.965 |
| 385001 | + | GACGAG | A | 472 | 6.389 | 333 | 0.974 | 0.908 | 1 |
| 385430 | - | GACGAG | A | 428 | 5.368 | 335 | 0.984 | 0.915 | 1 |
| 389636 | - | GACGAG | A | 403 | 5.093 | 351 | 0.948 | 0.862 | 1 |
| 391220 | - | GACGAG | A | 515 | 8.544 | 307 | 0.97 | 0.907 | 1 |
| 392072 | + | GACGAG | A | 500 | 7.545 | 331 | 1 | 0.973 | 1 |
| 395765 | + | GACGAG | A | 335 | 4.187 | 332 | 0.916 | 0.822 | 1 |
| 396194 | - | GACGAG | A | 433 | 6.844 | 322 | 1 | 0.983 | 1 |
| 398444 | + | GACGAG | A | 393 | 4.688 | 361 | 0.985 | 0.928 | 1 |
| 403756 | - | GACGAG | A | 436 | 7.305 | 299 | 1 | 0.95 | 1 |
| 403921 | - | GACGAG | A | 263 | 4.252 | 294 | 0.939 | 0.829 | 1 |
| 405720 | + | GACGAG | A | 274 | 5.056 | 252 | 0.886 | 0.801 | 0.98 |
| 407715 | - | GACGAG | A | 433 | 7.728 | 274 | 0.985 | 0.943 | 1 |
| 410006 | + | GACGAG | A | 293 | 4.673 | 284 | 0.918 | 0.82 | 1 |
| 412914 | - | GACGAG | A | 464 | 6.42 | 311 | 1 | 0.976 | 1 |
| 425191 | + | GACGAG | A | 538 | 7.818 | 345 | 0.998 | 0.988 | 1 |
| 426995 | + | GACGAG | A | 298 | 3.679 | 317 | 0.92 | 0.826 | 1 |
| 428338 | - | GACGAG | A | 450 | 6.791 | 291 | 1 | 0.952 | 1 |
| 432926 | - | GACGAG | A | 440 | 8.226 | 286 | 0.974 | 0.909 | 1 |
| 434531 | - | GACGAG | A | 265 | 4.391 | 303 | 1 | 0.984 | 1 |
| 435611 | - | GACGAG | A | 422 | 7.227 | 291 | 1 | 0.975 | 1 |
| 436617 | + | GACGAG | A | 353 | 5.267 | 292 | 0.964 | 0.883 | 1 |
| 439786 | - | GACGAG | A | 531 | 8.654 | 351 | 0.985 | 0.929 | 1 |
| 440961 | - | GACGAG | A | 460 | 4.828 | 346 | 0.999 | 0.98 | 1 |
| 442809 | - | GACGAG | A | 524 | 8.303 | 362 | 1 | 0.957 | 1 |
| 446359 | - | GACGAG | A | 473 | 6.81 | 328 | 0.985 | 0.938 | 1 |
| 447887 | - | GACGAG | A | 426 | 7.038 | 300 | 0.985 | 0.932 | 1 |
| 447901 | + | GACGAG | A | 467 | 8.767 | 311 | 0.963 | 0.896 | 1 |
| 451127 | + | GACGAG | A | 444 | 7.34 | 291 | 0.985 | 0.935 | 1 |
| 452376 | - | GACGAG | A | 313 | 4.662 | 270 | 0.985 | 0.897 | 1 |
| 454544 | - | GACGAG | A | 293 | 4.562 | 247 | 0.954 | 0.86 | 1 |
| 456133 | + | GACGAG | A | 337 | 8.39 | 217 | 0.985 | 0.922 | 1 |
| 458089 | - | GACGAG | A | 342 | 6.913 | 245 | 0.985 | 0.912 | 1 |
| 458818 | - | GACGAG | A | 339 | 8.372 | 243 | 1 | 0.948 | 1 |
| 459795 | + | GACGAG | A | 293 | 5.156 | 240 | 0.882 | 0.771 | 0.965 |
| 459819 | - | GACGAG | A | 282 | 4.665 | 256 | 1 | 0.969 | 1 |
| 460837 | + | GACGAG | A | 312 | 6.269 | 220 | 0.973 | 0.884 | 1 |
| 463340 | - | GACGAG | A | 351 | 10.707 | 253 | 0.96 | 0.894 | 1 |
| 464308 | + | GACGAG | A | 346 | 5.387 | 264 | 0.897 | 0.812 | 0.985 |
| 466770 | + | GACGAG | A | 65 | 2.611 | 291 | 0.804 | 0.686 | 0.922 |
| 467801 | - | GACGAG | A | 394 | 8.025 | 299 | 1 | 0.934 | 1 |
| 469025 | - | GACGAG | A | 453 | 7.419 | 302 | 1 | 0.979 | 1 |
| 472641 | + | GACGAG | A | 455 | 6.844 | 314 | 1 | 0.958 | 1 |
| 473039 | + | GACGAG | A | 502 | 7.326 | 315 | 0.972 | 0.912 | 1 |
| 473671 | - | GACGAG | A | 199 | 3.997 | 355 | 1 | 1 | 1 |
| 479026 | + | GACGAG | A | 603 | 7.745 | 398 | 0.964 | 0.905 | 1 |
| 481366 | - | GACGAG | A | 479 | 6.651 | 348 | 1 | 1 | 1 |
| 481657 | + | GACGAG | A | 553 | 7.216 | 367 | 1 | 0.974 | 1 |
| 482008 | - | GACGAG | A | 532 | 6.938 | 363 | 1 | 0.953 | 1 |
| 483068 | - | GACGAG | A | 493 | 6.398 | 366 | 1 | 0.983 | 1 |
| 485753 | - | GACGAG | A | 560 | 9.324 | 368 | 0.985 | 0.953 | 1 |
| 491444 | - | GACGAG | A | 510 | 8.403 | 352 | 1 | 0.948 | 1 |
| 492653 | - | GACGAG | A | 522 | 5.828 | 374 | 1 | 0.984 | 1 |
| 497290 | + | GACGAG | A | 417 | 5.597 | 325 | 0.928 | 0.86 | 0.993 |
| 498457 | - | GACGAG | A | 558 | 9.115 | 337 | 1 | 0.951 | 1 |
| 501624 | + | GACGAG | A | 206 | 4.104 | 256 | 1 | 0.936 | 1 |
| 502349 | - | GACGAG | A | 375 | 6.502 | 261 | 1 | 0.955 | 1 |
| 505583 | + | GACGAG | A | 349 | 5.514 | 283 | 0.978 | 0.89 | 1 |
| 509809 | + | GACGAG | A | 385 | 8.071 | 235 | 1 | 0.97 | 1 |
| 513597 | - | GACGAG | A | 163 | 3.475 | 218 | 0.892 | 0.775 | 1 |
| 517248 | + | GACGAG | A | 247 | 5.119 | 178 | 1 | 0.9 | 1 |
| 520546 | - | GACGAG | A | 209 | 4.949 | 174 | 1 | 0.902 | 1 |
| 530274 | - | GACGAG | A | 345 | 6.927 | 244 | 0.954 | 0.873 | 1 |
| 548788 | + | GACGAG | A | 301 | 4.705 | 253 | 0.93 | 0.82 | 1 |
| 550002 | - | GACGAG | A | 133 | 2.622 | 259 | 0.699 | 0.602 | 0.825 |
| 567992 | + | GACGAG | A | 314 | 3.903 | 288 | 0.864 | 0.768 | 0.948 |
| 572837 | + | GACGAG | A | 350 | 5.588 | 304 | 0.959 | 0.88 | 1 |
| 580078 | - | GACGAG | A | 499 | 6.839 | 317 | 1 | 0.942 | 1 |
| 580179 | + | GACGAG | A | 433 | 6.152 | 302 | 1 | 0.984 | 1 |
| 588709 | + | GACGAG | A | 254 | 4.202 | 312 | 1 | 0.906 | 1 |
| 590001 | + | GACGAG | A | 502 | 7.745 | 334 | 1 | 0.94 | 1 |
| 590094 | + | GACGAG | A | 441 | 5.335 | 338 | 0.964 | 0.889 | 1 |
| 591381 | - | GACGAG | A | 596 | 9.746 | 330 | 1 | 1 | 1 |
| 594980 | - | GACGAG | A | 434 | 7.323 | 310 | 1 | 0.979 | 1 |
| 601553 | - | GACGAG | A | 389 | 8.471 | 233 | 1 | 0.955 | 1 |
| 603326 | - | GACGAG | A | 298 | 5.158 | 252 | 0.957 | 0.869 | 1 |
| 608247 | - | GACGAG | A | 335 | 5.331 | 262 | 0.982 | 0.894 | 1 |
| 608688 | - | GACGAG | A | 420 | 6.606 | 274 | 1 | 0.985 | 1 |
| 625190 | + | GACGAG | A | 357 | 6.837 | 232 | 1 | 0.936 | 1 |
| 626626 | - | GACGAG | A | 281 | 3.935 | 229 | 0.961 | 0.865 | 1 |
| 633716 | + | GACGAG | A | 490 | 5.654 | 322 | 1 | 0.941 | 1 |
| 636022 | - | GACGAG | A | 397 | 8.632 | 278 | 0.979 | 0.911 | 1 |
| 647337 | + | GACGAG | A | 471 | 7.442 | 294 | 1 | 0.967 | 1 |
| 648316 | - | GACGAG | A | 420 | 7.439 | 297 | 0.985 | 0.924 | 1 |
| 648490 | + | GACGAG | A | 365 | 5.366 | 298 | 0.881 | 0.805 | 0.957 |
| 649382 | - | GACGAG | A | 385 | 6.34 | 289 | 0.966 | 0.889 | 1 |
| 650977 | - | GACGAG | A | 468 | 9.832 | 295 | 1 | 0.985 | 1 |
| 655988 | + | GACGAG | A | 354 | 5.052 | 300 | 0.882 | 0.801 | 0.968 |
| 658201 | - | GACGAG | A | 410 | 9.922 | 292 | 1 | 0.954 | 1 |
| 662988 | + | GACGAG | A | 432 | 6.537 | 328 | 0.985 | 0.918 | 1 |
| 666058 | + | GACGAG | A | 450 | 7.525 | 332 | 1 | 0.964 | 1 |
| 666824 | - | GACGAG | A | 524 | 6.484 | 363 | 0.969 | 0.903 | 1 |
| 666976 | - | GACGAG | A | 552 | 7.273 | 357 | 1 | 1 | 1 |
| 668121 | - | GACGAG | A | 531 | 5.898 | 378 | 1 | 1 | 1 |
| 673252 | - | GACGAG | A | 466 | 5.445 | 392 | 0.987 | 0.926 | 1 |
| 676798 | - | GACGAG | A | 431 | 6.925 | 294 | 1 | 0.983 | 1 |
| 680067 | + | GACGAG | A | 279 | 4.251 | 288 | 1 | 0.978 | 1 |
| 683944 | + | GACGAG | A | 425 | 4.813 | 358 | 1 | 0.94 | 1 |
| 685332 | + | GACGAG | A | 443 | 5.196 | 354 | 1 | 0.941 | 1 |
| 685878 | - | GACGAG | A | 423 | 5.666 | 331 | 0.972 | 0.897 | 1 |
| 686726 | - | GACGAG | A | 462 | 8.488 | 300 | 1 | 0.975 | 1 |
| 698351 | + | GACGAG | A | 539 | 6.956 | 374 | 0.985 | 0.925 | 1 |
| 701079 | - | GACGAG | A | 427 | 5.702 | 305 | 0.985 | 0.917 | 1 |
| 709399 | - | GACGAG | A | 354 | 6.016 | 256 | 0.986 | 0.91 | 1 |
| 712161 | - | GACGAG | A | 374 | 7.94 | 259 | 0.938 | 0.871 | 1 |
| 720239 | - | GACGAG | A | 554 | 10.122 | 376 | 1 | 0.993 | 1 |
| 721449 | - | GACGAG | A | 556 | 9.243 | 367 | 0.972 | 0.92 | 1 |
| 721949 | + | GACGAG | A | 550 | 8.683 | 352 | 0.976 | 0.917 | 1 |
| 723445 | + | GACGAG | A | 538 | 6.341 | 366 | 1 | 0.972 | 1 |
| 724288 | + | GACGAG | A | 530 | 6.789 | 362 | 0.931 | 0.865 | 0.985 |
| 725075 | + | GACGAG | A | 511 | 6.891 | 351 | 0.971 | 0.911 | 1 |
| 736473 | - | GACGAG | A | 467 | 6.563 | 341 | 1 | 0.938 | 1 |
| 739577 | - | GACGAG | A | 567 | 7.724 | 374 | 0.985 | 0.934 | 1 |
| 747025 | - | GACGAG | A | 383 | 6.062 | 289 | 0.953 | 0.881 | 1 |
| 750347 | - | GACGAG | A | 369 | 6.608 | 267 | 1 | 1 | 1 |
| 752753 | - | GACGAG | A | 366 | 5.682 | 277 | 0.967 | 0.879 | 1 |
| 755190 | + | GACGAG | A | 386 | 7.082 | 275 | 1 | 0.938 | 1 |
| 757348 | - | GACGAG | A | 70 | 2.921 | 280 | 0.954 | 0.832 | 1 |
| 757592 | + | GACGAG | A | 318 | 4.794 | 278 | 0.872 | 0.781 | 0.954 |
| 764540 | - | GACGAG | A | 547 | 6.547 | 363 | 1 | 0.981 | 1 |
| 771833 | + | GACGAG | A | 560 | 9.006 | 379 | 0.946 | 0.894 | 0.985 |
| 771897 | + | GACGAG | A | 538 | 6.973 | 378 | 1 | 0.95 | 1 |
| 773141 | + | GACGAG | A | 539 | 6.856 | 363 | 0.962 | 0.902 | 1 |
| 773412 | + | GACGAG | A | 496 | 6.851 | 364 | 0.974 | 0.909 | 1 |
| 773588 | - | GACGAG | A | 503 | 7.948 | 370 | 0.918 | 0.856 | 0.983 |
| 775083 | - | GACGAG | A | 586 | 8.233 | 381 | 1 | 0.95 | 1 |
| 778631 | - | GACGAG | A | 547 | 7.986 | 367 | 0.952 | 0.888 | 1 |
| 779619 | - | GACGAG | A | 592 | 8.446 | 369 | 0.985 | 0.944 | 1 |
| 785837 | + | GACGAG | A | 428 | 5.578 | 310 | 1 | 0.979 | 1 |
| 787959 | + | GACGAG | A | 508 | 10.303 | 330 | 1 | 0.946 | 1 |
| 788415 | + | GACGAG | A | 409 | 5.035 | 336 | 1 | 0.985 | 1 |
| 790709 | + | GACGAG | A | 459 | 7.602 | 320 | 0.947 | 0.886 | 1 |
| 795757 | + | GACGAG | A | 510 | 7.535 | 351 | 0.949 | 0.888 | 1 |
| 797739 | - | GACGAG | A | 466 | 9.305 | 325 | 0.985 | 0.94 | 1 |
| 798603 | - | GACGAG | A | 493 | 6.764 | 328 | 1 | 0.984 | 1 |
| 799130 | - | GACGAG | A | 522 | 8.533 | 324 | 1 | 0.985 | 1 |
| 799252 | + | GACGAG | A | 188 | 3.165 | 338 | 0.878 | 0.766 | 0.979 |
| 799929 | + | GACGAG | A | 444 | 6.638 | 335 | 0.985 | 0.914 | 1 |
| 800225 | + | GACGAG | A | 487 | 9.055 | 319 | 1 | 0.985 | 1 |
| 801271 | - | GACGAG | A | 298 | 5.271 | 283 | 1 | 0.924 | 1 |
| 805241 | + | GACGAG | A | 256 | 3.759 | 238 | 0.949 | 0.818 | 1 |
| 807462 | + | GACGAG | A | 128 | 2.562 | 228 | 0.954 | 0.845 | 1 |
| 810792 | - | GACGAG | A | 249 | 3.534 | 290 | 0.846 | 0.751 | 0.948 |
| 817152 | + | GACGAG | A | 478 | 6.851 | 333 | 1 | 0.978 | 1 |
| 818063 | - | GACGAG | A | 424 | 6.894 | 325 | 1 | 0.961 | 1 |
| 820817 | - | GACGAG | A | 523 | 7.203 | 341 | 1 | 0.985 | 1 |
| 829107 | - | GACGAG | A | 451 | 6.506 | 290 | 1 | 0.963 | 1 |
| 829167 | - | GACGAG | A | 420 | 8.278 | 287 | 0.985 | 0.938 | 1 |
| 830062 | - | GACGAG | A | 321 | 4.756 | 256 | 0.985 | 0.894 | 1 |
| 835649 | - | GACGAG | A | 485 | 6.129 | 322 | 1 | 0.93 | 1 |
| 837072 | + | GACGAG | A | 457 | 6.839 | 311 | 1 | 0.938 | 1 |
| 840052 | + | GACGAG | A | 49 | 3.102 | 263 | 1 | 0.869 | 1 |
| 840886 | + | GACGAG | A | 398 | 9.822 | 265 | 1 | 0.945 | 1 |
| 850960 | - | GACGAG | A | 392 | 5.499 | 285 | 0.964 | 0.881 | 1 |
| 852207 | + | GACGAG | A | 435 | 6.514 | 317 | 0.955 | 0.879 | 1 |
| 853004 | + | GACGAG | A | 483 | 7.309 | 309 | 1 | 0.981 | 1 |
| 856162 | - | GACGAG | A | 441 | 5.777 | 331 | 0.998 | 0.946 | 1 |
| 857884 | + | GACGAG | A | 488 | 7.459 | 346 | 0.985 | 0.931 | 1 |
| 860409 | + | GACGAG | A | 578 | 9.648 | 352 | 1 | 1 | 1 |
| 867479 | - | GACGAG | A | 501 | 8.045 | 323 | 1 | 0.951 | 1 |
| 874262 | - | GACGAG | A | 516 | 7.256 | 338 | 1 | 0.95 | 1 |
| 875855 | + | GACGAG | A | 323 | 4.06 | 327 | 0.906 | 0.809 | 0.985 |
| 884771 | - | GACGAG | A | 512 | 8.114 | 335 | 1 | 1 | 1 |
| 885755 | + | GACGAG | A | 498 | 7.087 | 343 | 0.985 | 0.924 | 1 |
| 886931 | - | GACGAG | A | 613 | 8.228 | 374 | 1 | 0.983 | 1 |
| 887150 | + | GACGAG | A | 362 | 4.18 | 351 | 0.962 | 0.885 | 1 |
| 889671 | + | GACGAG | A | 454 | 5.021 | 362 | 0.927 | 0.849 | 0.985 |
| 890110 | - | GACGAG | A | 497 | 6.831 | 361 | 0.981 | 0.912 | 1 |
| 897440 | - | GACGAG | A | 387 | 4.652 | 298 | 1 | 0.961 | 1 |
| 899039 | + | GACGAG | A | 447 | 7.355 | 337 | 0.938 | 0.876 | 1 |
| 903344 | + | GACGAG | A | 475 | 7.96 | 301 | 1 | 0.96 | 1 |
| 903500 | - | GACGAG | A | 386 | 5.127 | 288 | 0.952 | 0.864 | 1 |
| 903533 | - | GACGAG | A | 477 | 7.871 | 284 | 1 | 0.94 | 1 |
| 907208 | + | GACGAG | A | 418 | 9.789 | 291 | 0.938 | 0.878 | 1 |
| 912816 | - | GACGAG | A | 312 | 9.644 | 201 | 1 | 0.941 | 1 |
| 915012 | - | GACGAG | A | 249 | 6.564 | 178 | 1 | 0.927 | 1 |
| 919419 | + | GACGAG | A | 155 | 3.727 | 237 | 1 | 0.999 | 1 |
| 928123 | + | GACGAG | A | 278 | 5.006 | 223 | 0.851 | 0.759 | 0.958 |
| 928749 | + | GACGAG | A | 296 | 6.057 | 226 | 1 | 0.969 | 1 |
| 933467 | + | GACGAG | A | 294 | 6.828 | 214 | 0.932 | 0.848 | 1 |
| 938038 | + | GACGAG | A | 356 | 6.574 | 253 | 0.912 | 0.835 | 0.985 |
| 941056 | - | GACGAG | A | 393 | 6.384 | 277 | 0.942 | 0.863 | 1 |
| 944518 | + | GACGAG | A | 404 | 9.438 | 268 | 1 | 0.937 | 1 |
| 947125 | - | GACGAG | A | 300 | 4.403 | 271 | 0.955 | 0.845 | 1 |
| 948916 | - | GACGAG | A | 384 | 6.899 | 294 | 0.979 | 0.905 | 1 |
| 950270 | - | GACGAG | A | 356 | 7.22 | 284 | 0.968 | 0.893 | 1 |
| 953182 | + | GACGAG | A | 13 | 1.872 | 289 | NA | NA | NA |
| 956011 | - | GACGAG | A | 374 | 4.569 | 339 | 0.903 | 0.825 | 0.982 |
| 958725 | - | GACGAG | A | 510 | 7.996 | 336 | 0.983 | 0.925 | 1 |
| 964536 | - | GACGAG | A | 417 | 6.942 | 267 | 1 | 0.931 | 1 |
| 969090 | + | GACGAG | A | 437 | 7.036 | 283 | 1 | 1 | 1 |
| 973081 | + | GACGAG | A | 397 | 6.972 | 279 | 1 | 0.955 | 1 |
| 974552 | + | GACGAG | A | 401 | 6.853 | 278 | 1 | 1 | 1 |
| 976494 | + | GACGAG | A | 153 | 3.957 | 292 | 0.985 | 0.984 | 1 |
| 978454 | + | GACGAG | A | 405 | 5.752 | 296 | 0.976 | 0.902 | 1 |
| 980245 | - | GACGAG | A | 353 | 5.958 | 249 | 0.985 | 0.903 | 1 |
| 985368 | + | GACGAG | A | 321 | 7.432 | 259 | 0.914 | 0.832 | 0.985 |
| 985500 | + | GACGAG | A | 450 | 10.136 | 254 | 1 | 0.983 | 1 |
| 985584 | + | GACGAG | A | 369 | 7.544 | 253 | 1 | 0.984 | 1 |
| 986438 | + | GACGAG | A | 373 | 6.053 | 258 | 0.954 | 0.881 | 1 |
| 988937 | + | GACGAG | A | 344 | 6.669 | 231 | 0.924 | 0.843 | 1 |
| 1011487 | - | GACGAG | A | 441 | 8.898 | 312 | 0.985 | 0.95 | 1 |
| 1013532 | - | GACGAG | A | 543 | 10.077 | 325 | 1 | 0.985 | 1 |
| 1015410 | - | GACGAG | A | 459 | 9.506 | 298 | 1 | 0.973 | 1 |
| 1016789 | - | GACGAG | A | 433 | 9.768 | 288 | 1 | 0.976 | 1 |
| 1018700 | - | GACGAG | A | 478 | 5.596 | 313 | 1 | 1 | 1 |
| 1020352 | - | GACGAG | A | 401 | 4.536 | 348 | 0.929 | 0.842 | 0.993 |
| 1020514 | + | GACGAG | A | 437 | 10.227 | 312 | 1 | 0.943 | 1 |
| 1021836 | + | GACGAG | A | 448 | 6.209 | 328 | 1 | 0.951 | 1 |
| 1023064 | + | GACGAG | A | 468 | 6.511 | 340 | 1 | 0.962 | 1 |
| 1025899 | - | GACGAG | A | 453 | 7.903 | 323 | 0.979 | 0.919 | 1 |
| 1030300 | + | GACGAG | A | 463 | 7.606 | 284 | 1 | 1 | 1 |
| 1033087 | - | GACGAG | A | 340 | 5.339 | 248 | 0.984 | 0.925 | 1 |
| 1035284 | + | GACGAG | A | 341 | 4.508 | 286 | 0.962 | 0.867 | 1 |
| 1035422 | + | GACGAG | A | 426 | 8.229 | 293 | 0.962 | 0.896 | 1 |
| 1038041 | + | GACGAG | A | 409 | 6.476 | 303 | 0.94 | 0.858 | 1 |
| 1041301 | - | GACGAG | A | 140 | 3.151 | 300 | 0.945 | 0.828 | 1 |
| 1041793 | - | GACGAG | A | 404 | 6.897 | 310 | 0.953 | 0.882 | 1 |
| 1048424 | + | GACGAG | A | 394 | 6.211 | 297 | 0.925 | 0.839 | 0.985 |
| 1052949 | + | GACGAG | A | 370 | 7.287 | 237 | 1 | 0.985 | 1 |
| 1054134 | + | GACGAG | A | 288 | 9.313 | 199 | 1 | 0.965 | 1 |
| 1055680 | - | GACGAG | A | 191 | 5.081 | 110 | 1 | 0.939 | 1 |
| 1056375 | + | GACGAG | A | 158 | 7.043 | 120 | 0.864 | 0.725 | 0.964 |
| 1057905 | - | GACGAG | A | 136 | 4.515 | 93 | 1 | 1 | 1 |
| 1059874 | + | GACGAG | A | 169 | 12.163 | 98 | 1 | 0.947 | 1 |
| 1060210 | + | GACGAG | A | 134 | 6.244 | 96 | 0.892 | 0.75 | 1 |
| 1063299 | - | GACGAG | A | 259 | 6.539 | 186 | 1 | 0.906 | 1 |
| 1064384 | - | GACGAG | A | 405 | 11.544 | 239 | 0.984 | 0.928 | 1 |
| 1070827 | - | GACGAG | A | 393 | 7.931 | 279 | 0.985 | 0.928 | 1 |
| 1071704 | + | GACGAG | A | 423 | 9.815 | 256 | 0.972 | 0.902 | 1 |
| 1071867 | + | GACGAG | A | 377 | 7.524 | 249 | 1 | 0.94 | 1 |
| 1072192 | - | GACGAG | A | 433 | 6.714 | 267 | 0.985 | 0.941 | 1 |
| 1073380 | - | GACGAG | A | 246 | 4.009 | 272 | 0.954 | 0.872 | 1 |
| 1073715 | - | GACGAG | A | 441 | 6.634 | 270 | 1 | 1 | 1 |
| 1075292 | - | GACGAG | A | 365 | 4.958 | 313 | 0.923 | 0.845 | 0.985 |
| 1075329 | + | GACGAG | A | 460 | 8.725 | 300 | 0.977 | 0.917 | 1 |
| 1084804 | - | GACGAG | A | 398 | 6.136 | 269 | 0.941 | 0.868 | 1 |
| 1089976 | - | GACGAG | A | 202 | 3.345 | 239 | 0.808 | 0.693 | 0.926 |
| 1091115 | - | GACGAG | A | 422 | 8.651 | 255 | 1 | 0.975 | 1 |
| 1094236 | + | GACGAG | A | 466 | 7.304 | 299 | 1 | 0.984 | 1 |
| 1098995 | - | GACGAG | A | 408 | 7.155 | 292 | 0.964 | 0.888 | 1 |
| 1099618 | + | GACGAG | A | 451 | 8.646 | 292 | 0.984 | 0.92 | 1 |
| 1100316 | + | GACGAG | A | 502 | 10.088 | 297 | 1 | 0.958 | 1 |
| 1101285 | + | GACGAG | A | 429 | 6.164 | 308 | 1 | 0.947 | 1 |
| 1102169 | - | GACGAG | A | 450 | 5.37 | 319 | 1 | 0.922 | 1 |
| 1102430 | + | GACGAG | A | 423 | 6.34 | 314 | 0.945 | 0.885 | 1 |
| 1103283 | + | GACGAG | A | 434 | 7.949 | 312 | 0.929 | 0.861 | 0.985 |
| 1104105 | - | GACGAG | A | 485 | 8.494 | 321 | 0.984 | 0.924 | 1 |
| 1104360 | + | GACGAG | A | 473 | 8.451 | 323 | 0.901 | 0.844 | 0.965 |
| 1104405 | + | GACGAG | A | 467 | 5.741 | 318 | 0.985 | 0.928 | 1 |
| 1108132 | + | GACGAG | A | 517 | 8.647 | 304 | 1 | 0.952 | 1 |
| 1116765 | - | GACGAG | A | 136 | 3.125 | 286 | 0.914 | 0.793 | 1 |
| 1117946 | + | GACGAG | A | 395 | 6.52 | 296 | 0.984 | 0.908 | 1 |
| 1120311 | - | GACGAG | A | 496 | 9.805 | 312 | 1 | 0.985 | 1 |
| 1122108 | + | GACGAG | A | 374 | 4.452 | 334 | 0.939 | 0.849 | 1 |
| 1122858 | - | GACGAG | A | 466 | 5.93 | 336 | 1 | 1 | 1 |
| 1126172 | + | GACGAG | A | 335 | 4.292 | 312 | 1 | 0.918 | 1 |
| 1127471 | - | GACGAG | A | 439 | 6.063 | 322 | 0.967 | 0.896 | 1 |
| 1133877 | + | GACGAG | A | 437 | 8.143 | 305 | 1 | 0.943 | 1 |
| 1136078 | - | GACGAG | A | 410 | 8.333 | 313 | 0.928 | 0.851 | 0.986 |
| 1137145 | - | GACGAG | A | 354 | 5.014 | 289 | 0.965 | 0.87 | 1 |
| 1138010 | + | GACGAG | A | 418 | 5.743 | 285 | 1 | 0.985 | 1 |
| 1139958 | - | GACGAG | A | 492 | 10.259 | 323 | 1 | 0.95 | 1 |
| 1142917 | + | GACGAG | A | 535 | 9.401 | 344 | 1 | 0.961 | 1 |
| 1153451 | + | GACGAG | A | 427 | 6.325 | 309 | 0.985 | 0.916 | 1 |
| 1156733 | + | GACGAG | A | 424 | 5.394 | 314 | 0.951 | 0.867 | 1 |
| 1163551 | - | GACGAG | A | 427 | 8.13 | 283 | 1 | 0.955 | 1 |
| 1164412 | + | GACGAG | A | 520 | 10.21 | 287 | 1 | 0.985 | 1 |
| 1165453 | + | GACGAG | A | 397 | 7.816 | 289 | 0.965 | 0.905 | 1 |
| 1167960 | - | GACGAG | A | 234 | 4.718 | 227 | 1 | 0.946 | 1 |
| 1169424 | + | GACGAG | A | 294 | 5.193 | 226 | 1 | 1 | 1 |
| 1170534 | - | GACGAG | A | 271 | 7.752 | 171 | 1 | 0.957 | 1 |
| 1171347 | + | GACGAG | A | 219 | 4.194 | 188 | 0.951 | 0.833 | 1 |
| 1171864 | + | GACGAG | A | 297 | 7.974 | 186 | 1 | 0.922 | 1 |
| 1174680 | - | GACGAG | A | 280 | 5.549 | 216 | 1 | 0.933 | 1 |
| 1178877 | + | GACGAG | A | 381 | 6.28 | 252 | 1 | 1 | 1 |
| 1188664 | - | GACGAG | A | 396 | 5.86 | 273 | 1 | 0.934 | 1 |
| 1191797 | + | GACGAG | A | 424 | 8.897 | 285 | 0.976 | 0.912 | 1 |
| 1194288 | + | GACGAG | A | 398 | 6.145 | 277 | 1 | 1 | 1 |
| 1196346 | - | GACGAG | A | 382 | 7.752 | 267 | 0.984 | 0.909 | 1 |
| 1196874 | - | GACGAG | A | 436 | 9.932 | 263 | 1 | 0.96 | 1 |
| 1210157 | + | GACGAG | A | 293 | 4.695 | 222 | 0.925 | 0.822 | 1 |
| 1210361 | - | GACGAG | A | 334 | 8.479 | 224 | 1 | 0.939 | 1 |
| 1214636 | - | GACGAG | A | 224 | 3.817 | 215 | 0.93 | 0.801 | 1 |
| 1214735 | - | GACGAG | A | 219 | 5.129 | 211 | 1 | 0.93 | 1 |
| 1214855 | - | GACGAG | A | 343 | 7.588 | 221 | 1 | 0.972 | 1 |
| 1214870 | + | GACGAG | A | 325 | 9.607 | 209 | 0.951 | 0.882 | 1 |
| 1216910 | - | GACGAG | A | 304 | 5.433 | 236 | 1 | 0.972 | 1 |
| 1251236 | - | GACGAG | A | 360 | 4.889 | 289 | 0.974 | 0.881 | 1 |
| 1265523 | - | GACGAG | A | 337 | 8.373 | 231 | 0.985 | 0.916 | 1 |
| 1265769 | - | GACGAG | A | 395 | 8.481 | 237 | 0.954 | 0.883 | 1 |
| 1265874 | - | GACGAG | A | 356 | 7.804 | 235 | 0.945 | 0.876 | 1 |
| 1268549 | + | GACGAG | A | 12 | 1.727 | 206 | NA | NA | NA |
| 1271216 | + | GACGAG | A | 362 | 5.768 | 225 | 1 | 0.957 | 1 |
| 1279300 | - | GACGAG | A | 299 | 4.252 | 245 | 0.904 | 0.799 | 0.986 |
| 1285821 | + | GACGAG | A | 389 | 10.579 | 236 | 0.959 | 0.887 | 1 |
| 1285920 | - | GACGAG | A | 251 | 4.999 | 222 | 1 | 0.957 | 1 |
| 1287328 | - | GACGAG | A | 411 | 8.891 | 254 | 1 | 0.966 | 1 |
| 1292139 | + | GACGAG | A | 187 | 3.589 | 277 | 0.899 | 0.789 | 0.986 |
| 1292412 | + | GACGAG | A | 392 | 7.008 | 272 | 0.974 | 0.904 | 1 |
| 1292830 | + | GACGAG | A | 322 | 4.627 | 281 | 0.929 | 0.853 | 1 |
| 1294146 | - | GACGAG | A | 382 | 5.882 | 269 | 0.972 | 0.894 | 1 |
| 1302142 | + | GACGAG | A | 451 | 9.03 | 314 | 0.983 | 0.919 | 1 |
| 1302895 | + | GACGAG | A | 458 | 10.55 | 312 | 1 | 0.945 | 1 |
| 1306173 | + | GACGAG | A | 496 | 8.025 | 320 | 0.985 | 0.932 | 1 |
| 1311501 | - | GACGAG | A | 366 | 9.719 | 229 | 1 | 0.967 | 1 |
| 1313817 | - | GACGAG | A | 389 | 9.371 | 230 | 1 | 0.935 | 1 |
| 1316208 | - | GACGAG | A | 345 | 6.855 | 243 | 0.93 | 0.854 | 1 |
| 1320890 | - | GACGAG | A | 330 | 4.602 | 301 | 0.964 | 0.858 | 1 |
| 1322596 | + | GACGAG | A | 429 | 6.724 | 314 | 0.985 | 0.931 | 1 |
| 1324780 | + | GACGAG | A | 516 | 8.012 | 319 | 1 | 0.959 | 1 |
| 1324988 | - | GACGAG | A | 437 | 5.665 | 288 | 1 | 0.932 | 1 |
| 1329577 | + | GACGAG | A | 397 | 7.076 | 289 | 1 | 1 | 1 |
| 1331034 | - | GACGAG | A | 403 | 6.001 | 279 | 1 | 0.946 | 1 |
| 1332266 | - | GACGAG | A | 397 | 6.625 | 288 | 0.96 | 0.884 | 1 |
| 1332278 | + | GACGAG | A | 413 | 7.938 | 273 | 0.985 | 0.921 | 1 |
| 1332783 | - | GACGAG | A | 401 | 7.721 | 279 | 1 | 0.961 | 1 |
| 1343111 | - | GACGAG | A | 412 | 6.201 | 290 | 0.922 | 0.846 | 0.985 |
| 1345098 | + | GACGAG | A | 470 | 8.757 | 297 | 0.958 | 0.893 | 1 |
| 1348266 | - | GACGAG | A | 355 | 5.227 | 265 | 0.903 | 0.81 | 0.983 |
| 1351162 | + | GACGAG | A | 304 | 4.217 | 298 | 0.911 | 0.821 | 0.986 |
| 1352324 | - | GACGAG | A | 450 | 8.412 | 322 | 1 | 0.94 | 1 |
| 1352882 | + | GACGAG | A | 453 | 7.322 | 296 | 1 | 0.968 | 1 |
| 1353428 | - | GACGAG | A | 412 | 5.377 | 299 | 1 | 0.949 | 1 |
| 1355480 | - | GACGAG | A | 419 | 9.696 | 257 | 1 | 0.953 | 1 |
| 1358526 | + | GACGAG | A | 313 | 5.601 | 257 | 1 | 0.929 | 1 |
| 1362203 | + | GACGAG | A | 302 | 5.588 | 244 | 0.93 | 0.85 | 1 |
| 1365485 | + | GACGAG | A | 371 | 6.884 | 249 | 1 | 0.985 | 1 |
| 1373912 | - | GACGAG | A | 362 | 8.622 | 216 | 1 | 0.984 | 1 |
| 1374338 | - | GACGAG | A | 357 | 8.016 | 213 | 1 | 1 | 1 |
| 1377776 | + | GACGAG | A | 310 | 8.538 | 200 | 1 | 0.958 | 1 |
| 1384250 | + | GACGAG | A | 233 | 3.352 | 270 | 0.945 | 0.841 | 1 |
| 1384535 | - | GACGAG | A | 298 | 4.399 | 266 | 1 | 0.912 | 1 |
| 1389941 | + | GACGAG | A | 391 | 8.884 | 249 | 1 | 1 | 1 |
| 1390022 | + | GACGAG | A | 357 | 5.635 | 251 | 0.927 | 0.826 | 1 |
| 1392105 | - | GACGAG | A | 372 | 8.402 | 245 | 1 | 0.939 | 1 |
| 1396500 | + | GACGAG | A | 266 | 4.239 | 217 | 1 | 0.91 | 1 |
| 1397465 | - | GACGAG | A | 356 | 10.196 | 206 | 1 | 0.985 | 1 |
| 1400182 | + | GACGAG | A | 293 | 7.177 | 211 | 0.956 | 0.871 | 1 |
| 1401266 | - | GACGAG | A | 342 | 7.969 | 225 | 0.96 | 0.882 | 1 |
| 1402356 | + | GACGAG | A | 361 | 11.365 | 212 | 1 | 1 | 1 |
| 1404253 | - | GACGAG | A | 333 | 6.853 | 208 | 1 | 0.985 | 1 |
| 1410078 | + | GACGAG | A | 235 | 4.259 | 246 | 0.792 | 0.695 | 0.886 |
| 1410142 | - | GACGAG | A | 316 | 5.059 | 253 | 0.954 | 0.87 | 1 |
| 1413539 | + | GACGAG | A | 468 | 9.269 | 286 | 1 | 0.993 | 1 |
| 1414962 | + | GACGAG | A | 447 | 5.861 | 318 | 0.985 | 0.925 | 1 |
| 1419320 | + | GACGAG | A | 477 | 7.54 | 325 | 1 | 0.981 | 1 |
| 1421613 | - | GACGAG | A | 448 | 5.148 | 349 | 1 | 0.92 | 1 |
| 1422469 | - | GACGAG | A | 519 | 8.728 | 351 | 0.968 | 0.913 | 1 |
| 1424213 | + | GACGAG | A | 350 | 6.091 | 280 | 1 | 0.931 | 1 |
| 1440372 | + | GACGAG | A | 414 | 8.831 | 242 | 1 | 0.985 | 1 |
| 1442182 | + | GACGAG | A | 167 | 3.8 | 205 | 0.927 | 0.813 | 1 |
| 1448855 | + | GACGAG | A | 200 | 7.743 | 157 | 0.928 | 0.83 | 1 |
| 1449333 | + | GACGAG | A | 239 | 6.545 | 165 | 0.854 | 0.759 | 0.943 |
| 1452427 | + | GACGAG | A | 236 | 6.37 | 168 | 0.963 | 0.873 | 1 |
| 1455492 | + | GACGAG | A | 234 | 6.855 | 177 | 1 | 0.964 | 1 |
| 1455979 | + | GACGAG | A | 118 | 3.623 | 165 | 0.865 | 0.735 | 0.987 |
| 1460264 | + | GACGAG | A | 209 | 4.129 | 236 | 0.985 | 0.889 | 1 |
| 1469348 | + | GACGAG | A | 271 | 7.656 | 200 | 1 | 0.915 | 1 |
| 1470085 | + | GACGAG | A | 118 | 3.133 | 190 | 0.999 | 0.999 | 1 |
| 1476978 | + | GACGAG | A | 324 | 4.593 | 244 | 0.985 | 0.888 | 1 |
| 1483370 | - | GACGAG | A | 300 | 7.319 | 192 | 0.975 | 0.881 | 1 |
| 1489151 | + | GACGAG | A | 227 | 7.207 | 158 | 1 | 0.91 | 1 |
| 1493660 | + | GACGAG | A | 127 | 3.631 | 140 | 0.924 | 0.743 | 1 |
| 1493846 | + | GACGAG | A | 210 | 8.039 | 133 | 1 | 0.956 | 1 |
| 1495354 | - | GACGAG | A | 241 | 8.589 | 139 | 1 | 0.985 | 1 |
| 1497907 | - | GACGAG | A | 214 | 5.044 | 184 | 0.987 | 0.891 | 1 |
| 1498296 | + | GACGAG | A | 267 | 7.526 | 184 | 0.971 | 0.881 | 1 |
| 1509569 | - | GACGAG | A | 375 | 8.955 | 252 | 1 | 0.939 | 1 |
| 1513640 | + | GACGAG | A | 257 | 6.071 | 204 | 0.961 | 0.874 | 1 |
| 1531910 | + | GACGAG | A | 459 | 7.955 | 263 | 1 | 1 | 1 |
| 1531922 | - | GACGAG | A | 404 | 6.269 | 260 | 1 | 0.949 | 1 |
| 1535834 | - | GACGAG | A | 410 | 7.952 | 267 | 1 | 0.942 | 1 |
| 1540534 | + | GACGAG | A | 236 | 4.953 | 220 | 1 | 0.926 | 1 |
| 1542350 | + | GACGAG | A | 251 | 4.147 | 222 | 0.945 | 0.852 | 1 |
| 1544099 | - | GACGAG | A | 264 | 5.277 | 212 | 1 | 0.944 | 1 |
| 1550257 | - | GACGAG | A | 303 | 4.956 | 216 | 1 | 0.961 | 1 |
| 1550572 | + | GACGAG | A | 331 | 9.9 | 219 | 0.961 | 0.897 | 1 |
| 1556146 | - | GACGAG | A | 374 | 8.885 | 225 | 1 | 1 | 1 |
| 1560107 | + | GACGAG | A | 293 | 5.919 | 230 | 0.915 | 0.835 | 1 |
| 1569839 | - | GACGAG | A | 369 | 5.6 | 227 | 1 | 1 | 1 |
| 1574726 | + | GACGAG | A | 355 | 6.411 | 254 | 0.91 | 0.832 | 0.985 |
| 1576332 | - | GACGAG | A | 355 | 6.253 | 234 | 0.985 | 0.923 | 1 |
| 1581862 | - | GACGAG | A | 381 | 6.621 | 277 | 0.961 | 0.873 | 1 |
| 1582179 | - | GACGAG | A | 455 | 9.716 | 280 | 1 | 0.961 | 1 |
| 1585987 | + | GACGAG | A | 336 | 5.779 | 229 | 0.925 | 0.846 | 1 |
| 1588707 | + | GACGAG | A | 425 | 8.562 | 280 | 1 | 0.958 | 1 |
| 1591910 | - | GACGAG | A | 230 | 3.833 | 244 | 0.935 | 0.826 | 1 |
| 1600939 | + | GACGAG | A | 294 | 4.035 | 243 | 0.959 | 0.861 | 1 |
| 1600997 | + | GACGAG | A | 355 | 6.743 | 241 | 0.957 | 0.885 | 1 |
| 1603608 | - | GACGAG | A | 332 | 7.36 | 231 | 0.941 | 0.849 | 1 |
| 1604362 | + | GACGAG | A | 296 | 6.211 | 220 | 0.979 | 0.903 | 1 |
| 1608181 | + | GACGAG | A | 333 | 5.907 | 254 | 1 | 0.966 | 1 |
| 1610043 | + | GACGAG | A | 363 | 7.735 | 256 | 1 | 0.981 | 1 |
| 1611701 | - | GACGAG | A | 461 | 11.179 | 280 | 0.985 | 0.947 | 1 |
| 1613405 | + | GACGAG | A | 391 | 6.267 | 259 | 0.975 | 0.892 | 1 |
| 1613410 | + | GACGAG | A | 54 | 2.634 | 256 | 0.762 | 0.634 | 0.89 |
| 1615564 | + | GACGAG | A | 368 | 6.397 | 261 | 1 | 0.92 | 1 |
| 1615933 | + | GACGAG | A | 417 | 7.888 | 258 | 0.985 | 0.946 | 1 |
| 1617595 | - | GACGAG | A | 437 | 8.759 | 248 | 1 | 0.98 | 1 |
| 1621893 | + | GACGAG | A | 297 | 7.06 | 211 | 0.959 | 0.873 | 1 |
| 1623965 | + | GACGAG | A | 191 | 4.337 | 189 | 0.897 | 0.763 | 1 |
| 1625378 | + | GACGAG | A | 265 | 6.804 | 201 | 0.943 | 0.859 | 1 |
| 1628523 | + | GACGAG | A | 336 | 6.662 | 218 | 0.984 | 0.899 | 1 |
| 1637264 | + | GACGAG | A | 364 | 5.76 | 268 | 0.911 | 0.817 | 0.983 |
| 1640724 | + | GACGAG | A | 284 | 5.494 | 238 | 1 | 0.993 | 1 |
| 1641098 | + | GACGAG | A | 210 | 3.7 | 229 | 1 | 0.94 | 1 |
| 1642447 | + | GACGAG | A | 264 | 4.876 | 251 | 0.998 | 0.906 | 1 |
| 1648698 | - | GACGAG | A | 351 | 8.723 | 226 | 0.919 | 0.841 | 0.985 |
| 1651859 | + | GACGAG | A | 179 | 3.708 | 207 | 0.924 | 0.808 | 1 |
| 1659505 | + | GACGAG | A | 298 | 4.759 | 226 | 1 | 1 | 1 |
| 1662858 | + | GACGAG | A | 288 | 5.979 | 227 | 1 | 0.902 | 1 |
| 1667649 | - | GACGAG | A | 386 | 9.005 | 235 | 0.985 | 0.917 | 1 |
| 1671734 | + | GACGAG | A | 353 | 9.11 | 230 | 0.955 | 0.892 | 1 |
| 1672909 | - | GACGAG | A | 291 | 5.587 | 234 | 1 | 0.921 | 1 |
| 1676870 | + | GACGAG | A | 291 | 6.741 | 199 | 0.978 | 0.904 | 1 |
| 1694777 | + | GACGAG | A | 388 | 7.222 | 243 | 1 | 0.985 | 1 |
| 1698860 | + | GACGAG | A | 311 | 5.359 | 235 | 0.938 | 0.843 | 1 |
| 1699655 | + | GACGAG | A | 327 | 5.916 | 218 | 0.981 | 0.893 | 1 |
| 1704435 | + | GACGAG | A | 320 | 5.506 | 237 | 0.998 | 0.981 | 1 |
| 1704475 | + | GACGAG | A | 126 | 2.666 | 243 | 0.75 | 0.62 | 0.876 |
| 1705456 | + | GACGAG | A | 358 | 6.869 | 235 | 1 | 0.951 | 1 |
| 1705822 | + | GACGAG | A | 386 | 8.651 | 234 | 0.984 | 0.909 | 1 |
| 1705931 | + | GACGAG | A | 339 | 7.439 | 228 | 0.965 | 0.891 | 1 |
| 1706335 | + | GACGAG | A | 298 | 5.071 | 243 | 0.894 | 0.805 | 0.974 |
| 1711168 | + | GACGAG | A | 369 | 6.172 | 269 | 0.971 | 0.895 | 1 |
| 1711307 | - | GACGAG | A | 331 | 5.948 | 256 | 0.984 | 0.89 | 1 |
| 1717056 | + | GACGAG | A | 385 | 8.374 | 239 | 0.983 | 0.91 | 1 |
| 1720429 | - | GACGAG | A | 324 | 6.461 | 219 | 0.968 | 0.888 | 1 |
| 1723043 | + | GACGAG | A | 323 | 6.321 | 213 | 1 | 0.952 | 1 |
| 1724210 | + | GACGAG | A | 320 | 8.923 | 211 | 0.983 | 0.915 | 1 |
| 1731575 | + | GACGAG | A | 219 | 6.712 | 141 | 1 | 0.946 | 1 |
| 1731839 | + | GACGAG | A | 199 | 10.536 | 138 | 0.985 | 0.904 | 1 |
| 1738570 | + | GACGAG | A | 223 | 6.328 | 152 | 1 | 0.91 | 1 |
| 1741741 | - | GACGAG | A | 211 | 9.25 | 158 | 0.937 | 0.848 | 1 |
| 1742161 | - | GACGAG | A | 272 | 9.118 | 164 | 1 | 0.964 | 1 |
| 1748894 | + | GACGAG | A | 259 | 5.94 | 195 | 0.883 | 0.796 | 0.962 |
| 1749463 | - | GACGAG | A | 315 | 7.948 | 202 | 0.985 | 0.926 | 1 |
| 1763758 | + | GACGAG | A | 371 | 5.292 | 283 | 0.934 | 0.86 | 1 |
| 1764388 | + | GACGAG | A | 350 | 5.589 | 274 | 0.917 | 0.821 | 0.986 |
| 1768457 | + | GACGAG | A | 458 | 8.076 | 273 | 1 | 0.97 | 1 |
| 1770072 | - | GACGAG | A | 398 | 8.66 | 262 | 1 | 0.956 | 1 |
| 1772232 | - | GACGAG | A | 388 | 5.733 | 297 | 0.974 | 0.893 | 1 |
| 1774939 | + | GACGAG | A | 489 | 8.201 | 324 | 0.985 | 0.929 | 1 |
| 1775270 | + | GACGAG | A | 459 | 8.319 | 316 | 0.984 | 0.911 | 1 |
| 1782671 | + | GACGAG | A | 256 | 3.865 | 245 | 1 | 0.925 | 1 |
| 1783877 | - | GACGAG | A | 375 | 6.762 | 266 | 1 | 0.948 | 1 |
| 1787370 | + | GACGAG | A | 356 | 6.155 | 244 | 1 | 0.914 | 1 |
| 1796987 | + | GACGAG | A | 431 | 8.598 | 278 | 0.985 | 0.947 | 1 |
| 1797281 | + | GACGAG | A | 377 | 6.905 | 270 | 0.941 | 0.862 | 1 |
| 1802121 | + | GACGAG | A | 424 | 8.567 | 278 | 1 | 0.941 | 1 |
| 1803395 | + | GACGAG | A | 408 | 6.827 | 264 | 1 | 1 | 1 |
| 1803554 | + | GACGAG | A | 283 | 4.702 | 266 | 0.826 | 0.742 | 0.919 |
| 1812211 | + | GACGAG | A | 426 | 10.13 | 288 | 1 | 0.956 | 1 |
| 1812293 | - | GACGAG | A | 402 | 7.217 | 278 | 1 | 1 | 1 |
| 1815258 | - | GACGAG | A | 316 | 4.505 | 268 | 0.898 | 0.817 | 0.985 |
| 1819521 | - | GACGAG | A | 292 | 5.41 | 223 | 0.957 | 0.858 | 1 |
| 1819968 | - | GACGAG | A | 304 | 8.369 | 219 | 0.978 | 0.903 | 1 |
| 1820081 | - | GACGAG | A | 345 | 7.866 | 221 | 1 | 1 | 1 |
| 1831471 | + | GACGAG | A | 223 | 5.441 | 162 | 1 | 0.901 | 1 |
| 1835835 | - | GACGAG | A | 299 | 7.015 | 169 | 1 | 0.939 | 1 |
| 1837682 | + | GACGAG | A | 282 | 5.37 | 213 | 1 | 0.986 | 1 |
| 1839005 | + | GACGAG | A | 258 | 5.049 | 196 | 0.942 | 0.833 | 1 |
| 1839947 | + | GACGAG | A | 281 | 7.988 | 198 | 0.93 | 0.836 | 1 |
| 1840361 | - | GACGAG | A | 139 | 3.487 | 210 | 1 | 0.896 | 1 |
| 1854825 | + | GACGAG | A | 247 | 4.93 | 197 | 1 | 0.933 | 1 |
| 1872868 | + | GACGAG | A | 189 | 6.313 | 143 | 0.934 | 0.817 | 1 |
| 1875702 | + | GACGAG | A | 252 | 7.06 | 145 | 1 | 0.945 | 1 |
| 1878193 | + | GACGAG | A | 130 | 3.843 | 129 | 0.902 | 0.752 | 1 |
| 1879081 | - | GACGAG | A | 220 | 8.283 | 148 | 0.97 | 0.871 | 1 |
| 1879342 | + | GACGAG | A | 218 | 10.993 | 133 | 0.985 | 0.899 | 1 |
| 1880948 | - | GACGAG | A | 186 | 9.217 | 115 | 0.985 | 0.901 | 1 |
| 1881121 | - | GACGAG | A | 182 | 7.929 | 122 | 1 | 0.958 | 1 |
| 1893146 | - | GACGAG | A | 233 | 9.515 | 159 | 0.985 | 0.912 | 1 |
| 1894632 | + | GACGAG | A | 246 | 7.168 | 152 | 0.972 | 0.887 | 1 |
| 1896672 | + | GACGAG | A | 111 | 2.873 | 176 | 0.938 | 0.773 | 1 |
| 1897837 | + | GACGAG | A | 178 | 4.212 | 145 | 0.935 | 0.805 | 1 |
| 1899247 | + | GACGAG | A | 247 | 8.829 | 169 | 0.962 | 0.86 | 1 |
| 1899315 | + | GACGAG | A | 196 | 4.293 | 173 | 0.837 | 0.743 | 0.947 |
| 1900040 | + | GACGAG | A | 260 | 5.062 | 182 | 1 | 0.926 | 1 |
| 1907986 | - | GACGAG | A | 288 | 5.977 | 188 | 1 | 0.948 | 1 |
| 1910653 | - | GACGAG | A | 337 | 9.058 | 210 | 1 | 0.947 | 1 |
| 1912131 | + | GACGAG | A | 310 | 7.887 | 211 | 0.983 | 0.905 | 1 |
| 1919958 | - | GACGAG | A | 331 | 6.094 | 211 | 1 | 0.945 | 1 |
| 1928115 | + | GACGAG | A | 48 | 2.477 | 274 | 0.78 | 0.649 | 0.922 |
| 1929611 | + | GACGAG | A | 410 | 6.046 | 263 | 1 | 1 | 1 |
| 1930197 | - | GACGAG | A | 325 | 5.053 | 263 | 0.981 | 0.901 | 1 |
| 1931415 | - | GACGAG | A | 27 | 1.908 | 235 | 0.642 | 0.492 | 0.784 |
| 1934576 | + | GACGAG | A | 327 | 4.854 | 272 | 1 | 0.913 | 1 |
| 1936705 | - | GACGAG | A | 362 | 9.09 | 238 | 0.985 | 0.923 | 1 |
| 1939147 | - | GACGAG | A | 366 | 8.533 | 246 | 0.98 | 0.912 | 1 |
| 1946842 | - | GACGAG | A | 40 | 2.211 | 229 | 0.759 | 0.627 | 0.911 |
| 1950959 | + | GACGAG | A | 184 | 3.444 | 219 | 0.941 | 0.822 | 1 |
| 1964090 | + | GACGAG | A | 17 | 2.135 | 210 | NA | NA | NA |
| 1973608 | + | GACGAG | A | 210 | 8.597 | 132 | 1 | 0.968 | 1 |
| 1976036 | - | GACGAG | A | 292 | 5.918 | 193 | 0.985 | 0.918 | 1 |
| 1983143 | - | GACGAG | A | 277 | 6.632 | 176 | 1 | 0.985 | 1 |
| 2003993 | - | GACGAG | A | 227 | 4.733 | 181 | 0.921 | 0.805 | 1 |
| 2004973 | - | GACGAG | A | 264 | 5.49 | 199 | 0.948 | 0.845 | 1 |
| 2007195 | - | GACGAG | A | 262 | 6.697 | 173 | 0.964 | 0.867 | 1 |
| 2010837 | + | GACGAG | A | 214 | 5.935 | 134 | 1 | 0.894 | 1 |
| 2017278 | + | GACGAG | A | 180 | 4.1 | 125 | 1 | 0.925 | 1 |
| 2018151 | - | GACGAG | A | 223 | 6.897 | 142 | 1 | 0.931 | 1 |
| 2022532 | - | GACGAG | A | 256 | 5.817 | 169 | 0.95 | 0.846 | 1 |
| 2033072 | - | GACGAG | A | 294 | 4.807 | 220 | 0.985 | 0.897 | 1 |
| 2037318 | - | GACGAG | A | 303 | 6.904 | 190 | 1 | 0.973 | 1 |
| 2044621 | - | GACGAG | A | 253 | 4.858 | 197 | 0.999 | 0.98 | 1 |
| 2046488 | - | GACGAG | A | 363 | 8.473 | 218 | 1 | 1 | 1 |
| 2047081 | - | GACGAG | A | 396 | 9.045 | 217 | 1 | 0.94 | 1 |
| 2047622 | - | GACGAG | A | 363 | 6.558 | 218 | 1 | 1 | 1 |
| 2049715 | + | GACGAG | A | 324 | 6.671 | 231 | 0.952 | 0.876 | 1 |
| 2054253 | + | GACGAG | A | 318 | 7.144 | 217 | 1 | 0.976 | 1 |
| 2057307 | - | GACGAG | A | 278 | 8.703 | 189 | 0.958 | 0.881 | 1 |
| 2061525 | + | GACGAG | A | 365 | 8.034 | 249 | 1 | 0.984 | 1 |
| 2063724 | - | GACGAG | A | 347 | 7.447 | 235 | 0.965 | 0.897 | 1 |
| 2066245 | - | GACGAG | A | 329 | 6.956 | 214 | 0.985 | 0.926 | 1 |
| 2067115 | - | GACGAG | A | 262 | 5.508 | 183 | 0.973 | 0.883 | 1 |
| 2076883 | + | GACGAG | A | 282 | 6.786 | 190 | 1 | 0.953 | 1 |
| 2081077 | - | GACGAG | A | 249 | 5.231 | 198 | 0.985 | 0.895 | 1 |
| 2082777 | - | GACGAG | A | 303 | 7.301 | 207 | 0.985 | 0.922 | 1 |
| 2085268 | - | GACGAG | A | 313 | 7.467 | 191 | 1 | 0.928 | 1 |
| 2095321 | - | GACGAG | A | 343 | 5.962 | 222 | 1 | 0.937 | 1 |
| 2097837 | + | GACGAG | A | 394 | 8.81 | 240 | 1 | 0.985 | 1 |
| 2105418 | - | GACGAG | A | 295 | 8.399 | 198 | 0.964 | 0.892 | 1 |
| 2119045 | - | GACGAG | A | 228 | 3.944 | 210 | 0.869 | 0.763 | 0.985 |
| 2123784 | + | GACGAG | A | 270 | 10.508 | 178 | 1 | 0.944 | 1 |
| 2126450 | + | GACGAG | A | 117 | 2.952 | 188 | 0.943 | 0.793 | 1 |
| 2127349 | - | GACGAG | A | 289 | 6.797 | 191 | 0.938 | 0.853 | 1 |
| 2132492 | + | GACGAG | A | 349 | 7.834 | 196 | 1 | 0.985 | 1 |
| 2138966 | - | GACGAG | A | 255 | 5.023 | 184 | 1 | 0.993 | 1 |
| 2145888 | - | GACGAG | A | 299 | 7.55 | 174 | 0.985 | 0.926 | 1 |
| 2146248 | - | GACGAG | A | 267 | 7.809 | 188 | 0.951 | 0.857 | 1 |
| 2147142 | - | GACGAG | A | 290 | 8.187 | 188 | 0.979 | 0.891 | 1 |
| 2153532 | - | GACGAG | A | 347 | 8.362 | 210 | 1 | 0.984 | 1 |
| 2154562 | - | GACGAG | A | 175 | 3.444 | 221 | 0.78 | 0.66 | 0.904 |
| 2165134 | - | GACGAG | A | 290 | 7.144 | 213 | 0.957 | 0.879 | 1 |
| 2169622 | - | GACGAG | A | 293 | 7.264 | 203 | 1 | 0.928 | 1 |
| 2175531 | - | GACGAG | A | 317 | 6.018 | 216 | 0.947 | 0.854 | 1 |
| 2188067 | - | GACGAG | A | 335 | 8.299 | 218 | 1 | 0.927 | 1 |
| 2188643 | - | GACGAG | A | 280 | 5.836 | 210 | 0.969 | 0.882 | 1 |
| 2189488 | + | GACGAG | A | 321 | 7.364 | 215 | 0.934 | 0.851 | 1 |
| 2190248 | + | GACGAG | A | 306 | 7.239 | 204 | 0.976 | 0.878 | 1 |
| 2196935 | + | GACGAG | A | 390 | 6.58 | 273 | 0.985 | 0.924 | 1 |
| 2197293 | - | GACGAG | A | 342 | 6.867 | 250 | 0.985 | 0.91 | 1 |
| 2197308 | - | GACGAG | A | 347 | 6.543 | 251 | 0.976 | 0.892 | 1 |
| 2197917 | + | GACGAG | A | 390 | 6.357 | 255 | 0.985 | 0.927 | 1 |
| 2200377 | - | GACGAG | A | 339 | 7.316 | 220 | 0.984 | 0.903 | 1 |
| 2203931 | - | GACGAG | A | 311 | 8.847 | 189 | 0.985 | 0.919 | 1 |
| 2213484 | - | GACGAG | A | 380 | 9.172 | 226 | 0.976 | 0.912 | 1 |
| 2219207 | + | GACGAG | A | 270 | 6.455 | 198 | 0.985 | 0.896 | 1 |
| 2219738 | + | GACGAG | A | 233 | 6.104 | 199 | 0.961 | 0.862 | 1 |
| 2226560 | - | GACGAG | A | 317 | 6.53 | 215 | 0.985 | 0.895 | 1 |
| 2231342 | - | GACGAG | A | 38 | 2.176 | 256 | 0.793 | 0.651 | 0.965 |
| 2235000 | + | GACGAG | A | 213 | 3.138 | 246 | 0.806 | 0.698 | 0.923 |
| 2243216 | - | GACGAG | A | 296 | 7.98 | 214 | 0.985 | 0.912 | 1 |
| 2244531 | - | GACGAG | A | 366 | 5.552 | 224 | 1 | 0.963 | 1 |
| 2245496 | - | GACGAG | A | 313 | 7.921 | 221 | 1 | 0.925 | 1 |
| 2246566 | - | GACGAG | A | 140 | 4.176 | 229 | 1 | 0.948 | 1 |
| 2248466 | - | GACGAG | A | 330 | 6.23 | 237 | 0.958 | 0.88 | 1 |
| 2249138 | + | GACGAG | A | 379 | 6.948 | 251 | 1 | 0.985 | 1 |
| 2256436 | + | GACGAG | A | 407 | 9.533 | 269 | 1 | 0.974 | 1 |
| 2257393 | + | GACGAG | A | 384 | 8.059 | 253 | 0.982 | 0.906 | 1 |
| 2257631 | - | GACGAG | A | 347 | 8.866 | 237 | 1 | 0.952 | 1 |
| 2266968 | - | GACGAG | A | 365 | 8.318 | 235 | 1 | 1 | 1 |
| 2271128 | + | GACGAG | A | 254 | 4.716 | 252 | 0.895 | 0.791 | 0.982 |
| 2271338 | + | GACGAG | A | 347 | 6.541 | 234 | 0.931 | 0.847 | 1 |
| 2273395 | - | GACGAG | A | 347 | 6.312 | 249 | 1 | 1 | 1 |
| 2276510 | - | GACGAG | A | 403 | 7.391 | 243 | 1 | 0.985 | 1 |
| 2280814 | - | GACGAG | A | 382 | 7.564 | 261 | 0.928 | 0.843 | 0.985 |
| 2282147 | - | GACGAG | A | 399 | 6.723 | 266 | 1 | 0.935 | 1 |
| 2286556 | - | GACGAG | A | 112 | 2.947 | 260 | 0.892 | 0.766 | 1 |
| 2288075 | + | GACGAG | A | 389 | 8.415 | 265 | 0.976 | 0.908 | 1 |
| 2290044 | + | GACGAG | A | 365 | 8.633 | 260 | 0.94 | 0.865 | 1 |
| 2290784 | + | GACGAG | A | 378 | 6.327 | 249 | 1 | 0.947 | 1 |
| 2291137 | + | GACGAG | A | 366 | 6.563 | 251 | 0.97 | 0.887 | 1 |
| 2293037 | - | GACGAG | A | 329 | 6.54 | 200 | 1 | 0.97 | 1 |
| 2293597 | - | GACGAG | A | 294 | 5.681 | 206 | 0.985 | 0.915 | 1 |
| 2293949 | + | GACGAG | A | 308 | 10.534 | 216 | 0.946 | 0.87 | 1 |
| 2296538 | - | GACGAG | A | 249 | 6.895 | 184 | 0.984 | 0.894 | 1 |
| 2298261 | + | GACGAG | A | 339 | 7.107 | 224 | 1 | 0.95 | 1 |
| 2308678 | - | GACGAG | A | 346 | 7.977 | 236 | 0.985 | 0.91 | 1 |
| 2311141 | + | GACGAG | A | 387 | 9.021 | 243 | 1 | 0.963 | 1 |
| 2311234 | + | GACGAG | A | 337 | 8.096 | 241 | 0.983 | 0.91 | 1 |
| 2313371 | + | GACGAG | A | 334 | 6.193 | 238 | 0.975 | 0.891 | 1 |
| 2314681 | - | GACGAG | A | 329 | 5.518 | 227 | 0.95 | 0.873 | 1 |
| 2314710 | - | GACGAG | A | 242 | 3.861 | 227 | 0.867 | 0.763 | 0.971 |
| 2321818 | + | GACGAG | A | 181 | 8.622 | 126 | 1 | 0.922 | 1 |
| 2322949 | - | GACGAG | A | 183 | 7.771 | 109 | 1 | 0.923 | 1 |
| 2324501 | - | GACGAG | A | 185 | 9.067 | 105 | 1 | 0.91 | 1 |
| 2326592 | - | GACGAG | A | 231 | 7.846 | 133 | 1 | 1 | 1 |
| 2328094 | + | GACGAG | A | 170 | 5.534 | 151 | 0.985 | 0.847 | 1 |
| 2331448 | - | GACGAG | A | 253 | 4.869 | 211 | 1 | 0.975 | 1 |
| 2338730 | - | GACGAG | A | 332 | 6.354 | 252 | 1 | 0.959 | 1 |
| 2342783 | + | GACGAG | A | 314 | 8.803 | 220 | 0.952 | 0.887 | 1 |
| 2342996 | - | GACGAG | A | 315 | 6.415 | 240 | 0.927 | 0.833 | 1 |
| 2344148 | - | GACGAG | A | 39 | 2.747 | 236 | 0.924 | 0.805 | 1 |
| 2350017 | - | GACGAG | A | 289 | 4.802 | 204 | 0.975 | 0.88 | 1 |
| 2359191 | + | GACGAG | A | 373 | 5.573 | 267 | 1 | 0.981 | 1 |
| 2365198 | + | GACGAG | A | 338 | 7.663 | 254 | 0.978 | 0.899 | 1 |
| 2373422 | - | GACGAG | A | 259 | 7.326 | 171 | 1 | 0.931 | 1 |
| 2379788 | - | GACGAG | A | 242 | 4.267 | 215 | 0.933 | 0.832 | 1 |
| 2386280 | - | GACGAG | A | 313 | 7.808 | 182 | 1 | 0.985 | 1 |
| 2387423 | - | GACGAG | A | 66 | 2.724 | 182 | 0.862 | 0.714 | 1 |
| 2389430 | - | GACGAG | A | 325 | 5.77 | 216 | 1 | 1 | 1 |
| 2403324 | + | GACGAG | A | 426 | 6.971 | 267 | 1 | 1 | 1 |
| 2411847 | - | GACGAG | A | 411 | 5.773 | 271 | 1 | 0.926 | 1 |
| 2426596 | + | GACGAG | A | 308 | 5.712 | 231 | 1 | 0.923 | 1 |
| 2428185 | - | GACGAG | A | 232 | 3.769 | 237 | 0.887 | 0.78 | 0.986 |
| 2428671 | - | GACGAG | A | 329 | 7.189 | 243 | 1 | 0.946 | 1 |
| 2432553 | - | GACGAG | A | 349 | 6.572 | 264 | 0.933 | 0.849 | 1 |
| 2433260 | - | GACGAG | A | 322 | 4.921 | 280 | 0.952 | 0.869 | 1 |
| 2435811 | + | GACGAG | A | 394 | 5.811 | 262 | 1 | 0.969 | 1 |
| 2448617 | - | GACGAG | A | 356 | 7.029 | 263 | 0.964 | 0.886 | 1 |
| 2451999 | - | GACGAG | A | 418 | 8.798 | 248 | 1 | 0.955 | 1 |
| 2452024 | + | GACGAG | A | 350 | 6.967 | 256 | 0.938 | 0.854 | 1 |
| 2456128 | + | GACGAG | A | 309 | 7.319 | 207 | 1 | 0.928 | 1 |
| 2458815 | - | GACGAG | A | 227 | 6.232 | 185 | 0.969 | 0.856 | 1 |
| 2459453 | - | GACGAG | A | 244 | 4.311 | 200 | 0.919 | 0.795 | 1 |
| 2459893 | + | GACGAG | A | 342 | 5.926 | 201 | 1 | 0.941 | 1 |
| 2460187 | - | GACGAG | A | 253 | 6.723 | 187 | 0.97 | 0.871 | 1 |
| 2460664 | - | GACGAG | A | 207 | 5.824 | 189 | 0.879 | 0.766 | 0.979 |
| 2461827 | - | GACGAG | A | 275 | 7.397 | 195 | 0.985 | 0.918 | 1 |
| 2464938 | - | GACGAG | A | 277 | 5.399 | 201 | 1 | 0.943 | 1 |
| 2470049 | - | GACGAG | A | 161 | 6.538 | 93 | 1 | 0.888 | 1 |
| 2481913 | - | GACGAG | A | 205 | 3.711 | 207 | 0.764 | 0.649 | 0.873 |
| 2488021 | - | GACGAG | A | 341 | 5.768 | 267 | 0.954 | 0.86 | 1 |
| 2489579 | - | GACGAG | A | 373 | 6.05 | 276 | 1 | 0.937 | 1 |
| 2493371 | - | GACGAG | A | 228 | 4.298 | 226 | 0.906 | 0.821 | 1 |
| 2499102 | - | GACGAG | A | 400 | 7.699 | 249 | 1 | 0.943 | 1 |
| 2500512 | - | GACGAG | A | 253 | 4.883 | 243 | 0.929 | 0.824 | 1 |
| 2500665 | - | GACGAG | A | 299 | 4.909 | 237 | 1 | 0.967 | 1 |
| 2502726 | + | GACGAG | A | 319 | 8.585 | 205 | 0.956 | 0.887 | 1 |
| 2502838 | - | GACGAG | A | 322 | 10.124 | 235 | 0.973 | 0.889 | 1 |
| 2506281 | - | GACGAG | A | 331 | 5.756 | 247 | 1 | 0.949 | 1 |
| 2511551 | - | GACGAG | A | 277 | 3.848 | 242 | 0.944 | 0.84 | 1 |
| 2514308 | - | GACGAG | A | 271 | 5.132 | 215 | 1 | 0.909 | 1 |
| 2521910 | - | GACGAG | A | 183 | 4.056 | 148 | 1 | 0.948 | 1 |
| 2528739 | - | GACGAG | A | 234 | 4.454 | 192 | 0.983 | 0.864 | 1 |
| 2529192 | - | GACGAG | A | 307 | 8.689 | 181 | 0.985 | 0.931 | 1 |
| 2530681 | - | GACGAG | A | 222 | 5.607 | 159 | 0.982 | 0.867 | 1 |
| 2536425 | + | GACGAG | A | 289 | 5.988 | 172 | 1 | 0.909 | 1 |
| 2561307 | - | GACGAG | A | 289 | 5.163 | 216 | 0.953 | 0.854 | 1 |
| 2566037 | - | GACGAG | A | 301 | 6.858 | 200 | 0.947 | 0.87 | 1 |
| 2575148 | + | GACGAG | A | 307 | 4.841 | 243 | 1 | 0.947 | 1 |
| 2583532 | + | GACGAG | A | 341 | 7.138 | 255 | 0.985 | 0.903 | 1 |
| 2604207 | - | GACGAG | A | 341 | 6.939 | 251 | 1 | 0.957 | 1 |
| 2608699 | - | GACGAG | A | 448 | 6.79 | 285 | 1 | 1 | 1 |
| 2609324 | + | GACGAG | A | 269 | 5.504 | 273 | 0.999 | 0.999 | 1 |
| 2612897 | - | GACGAG | A | 433 | 8.796 | 275 | 1 | 1 | 1 |
| 2615383 | - | GACGAG | A | 404 | 8.941 | 257 | 1 | 0.943 | 1 |
| 2616500 | - | GACGAG | A | 396 | 8.338 | 262 | 0.944 | 0.875 | 1 |
| 2617427 | - | GACGAG | A | 355 | 6.112 | 284 | 0.93 | 0.828 | 1 |
| 2618754 | + | GACGAG | A | 390 | 6.06 | 258 | 1 | 0.951 | 1 |
| 2619947 | + | GACGAG | A | 359 | 7.053 | 256 | 1 | 0.96 | 1 |
| 2620696 | + | GACGAG | A | 399 | 7.924 | 254 | 1 | 0.951 | 1 |
| 2622207 | - | GACGAG | A | 444 | 7.859 | 292 | 0.979 | 0.918 | 1 |
| 2622569 | - | GACGAG | A | 367 | 5.74 | 290 | 0.928 | 0.848 | 1 |
| 2623577 | - | GACGAG | A | 430 | 6.059 | 295 | 1 | 0.94 | 1 |
| 2632634 | - | GACGAG | A | 444 | 9.374 | 309 | 0.963 | 0.897 | 1 |
| 2632940 | + | GACGAG | A | 191 | 3.321 | 307 | 0.905 | 0.788 | 1 |
| 2634286 | - | GACGAG | A | 369 | 5.193 | 301 | 1 | 0.932 | 1 |
| 2635763 | + | GACGAG | A | 426 | 6.225 | 298 | 1 | 0.939 | 1 |
| 2648697 | - | GACGAG | A | 349 | 7.792 | 242 | 0.963 | 0.892 | 1 |
| 2649759 | + | GACGAG | A | 352 | 5.94 | 264 | 1 | 1 | 1 |
| 2653528 | - | GACGAG | A | 366 | 6.182 | 269 | 1 | 0.97 | 1 |
| 2653995 | + | GACGAG | A | 338 | 6.928 | 242 | 0.938 | 0.867 | 1 |
| 2669732 | + | GACGAG | A | 339 | 8.917 | 236 | 0.964 | 0.896 | 1 |
| 2670786 | - | GACGAG | A | 396 | 6.387 | 240 | 1 | 0.934 | 1 |
| 2671090 | - | GACGAG | A | 299 | 5.112 | 235 | 0.976 | 0.884 | 1 |
| 2673903 | - | GACGAG | A | 384 | 7.367 | 268 | 0.98 | 0.907 | 1 |
| 2678254 | - | GACGAG | A | 408 | 9.656 | 258 | 0.985 | 0.933 | 1 |
| 2684160 | + | GACGAG | A | 399 | 5.65 | 297 | 1 | 0.974 | 1 |
| 2685599 | - | GACGAG | A | 439 | 9.579 | 288 | 0.985 | 0.952 | 1 |
| 2686442 | - | GACGAG | A | 433 | 7.688 | 280 | 0.984 | 0.912 | 1 |
| 2691725 | + | GACGAG | A | 357 | 9.572 | 240 | 0.94 | 0.877 | 1 |
| 2692228 | + | GACGAG | A | 346 | 6.378 | 239 | 0.985 | 0.911 | 1 |
| 2693818 | - | GACGAG | A | 352 | 6.337 | 270 | 0.97 | 0.884 | 1 |
| 2697815 | - | GACGAG | A | 365 | 8.083 | 241 | 0.985 | 0.923 | 1 |
| 2698357 | - | GACGAG | A | 250 | 5.403 | 228 | 0.999 | 0.966 | 1 |
| 2700451 | - | GACGAG | A | 349 | 7.061 | 249 | 1 | 0.946 | 1 |
| 2701672 | - | GACGAG | A | 329 | 9.161 | 223 | 0.966 | 0.896 | 1 |
| 2706245 | - | GACGAG | A | 304 | 4.782 | 231 | 1 | 0.919 | 1 |
| 2707494 | + | GACGAG | A | 283 | 5.679 | 218 | 0.927 | 0.847 | 1 |
| 2708115 | - | GACGAG | A | 44 | 2.726 | 246 | 0.941 | 0.802 | 1 |
| 2708386 | - | GACGAG | A | 333 | 4.543 | 253 | 1 | 0.954 | 1 |
| 2717546 | - | GACGAG | A | 150 | 3.274 | 202 | 0.895 | 0.759 | 1 |
| 2718193 | - | GACGAG | A | 305 | 8.635 | 211 | 0.972 | 0.903 | 1 |
| 2718263 | - | GACGAG | A | 250 | 5.121 | 215 | 1 | 0.945 | 1 |
| 2721399 | - | GACGAG | A | 325 | 6.988 | 219 | 0.985 | 0.912 | 1 |
| 2722254 | - | GACGAG | A | 350 | 7.344 | 223 | 1 | 0.954 | 1 |
| 2725528 | - | GACGAG | A | 383 | 10.642 | 264 | 1 | 0.978 | 1 |
| 2729193 | - | GACGAG | A | 373 | 5.369 | 305 | 1 | 0.986 | 1 |
| 2729667 | - | GACGAG | A | 430 | 7.821 | 293 | 0.984 | 0.921 | 1 |
| 2738708 | + | GACGAG | A | 302 | 3.986 | 312 | 0.952 | 0.863 | 1 |
| 2739217 | - | GACGAG | A | 234 | 3.489 | 295 | 0.81 | 0.705 | 0.908 |
| 2743397 | - | GACGAG | A | 364 | 9.472 | 243 | 0.985 | 0.926 | 1 |
| 2743821 | + | GACGAG | A | 379 | 7.844 | 257 | 0.985 | 0.913 | 1 |
| 2748406 | + | GACGAG | A | 285 | 6.331 | 200 | 1 | 0.934 | 1 |
| 2751118 | - | GACGAG | A | 242 | 7.702 | 141 | 1 | 1 | 1 |
| 2762534 | - | GACGAG | A | 347 | 8.114 | 219 | 0.983 | 0.903 | 1 |
| 2766059 | - | GACGAG | A | 377 | 6.373 | 270 | 1 | 0.958 | 1 |
| 2767439 | - | GACGAG | A | 263 | 3.904 | 246 | 0.984 | 0.864 | 1 |
| 2779096 | + | GACGAG | A | 334 | 5.247 | 287 | 0.962 | 0.873 | 1 |
| 2780086 | - | GACGAG | A | 454 | 10.388 | 271 | 1 | 0.963 | 1 |
| 2781169 | - | GACGAG | A | 394 | 8.396 | 252 | 1 | 0.935 | 1 |
| 2783674 | + | GACGAG | A | 392 | 9.78 | 281 | 1 | 0.951 | 1 |
| 2790274 | - | GACGAG | A | 454 | 8.96 | 271 | 1 | 1 | 1 |
| 2791335 | - | GACGAG | A | 406 | 9.612 | 269 | 1 | 0.97 | 1 |
| 2791881 | - | GACGAG | A | 374 | 6.143 | 274 | 0.985 | 0.922 | 1 |
| 2792573 | - | GACGAG | A | 356 | 8.096 | 243 | 0.985 | 0.923 | 1 |
| 2793014 | - | GACGAG | A | 245 | 4.143 | 247 | 0.892 | 0.795 | 0.986 |
| 2801001 | + | GACGAG | A | 291 | 5.02 | 258 | 0.964 | 0.88 | 1 |
| 2801913 | - | GACGAG | A | 142 | 3.573 | 274 | 1 | 0.905 | 1 |
| 2802057 | - | GACGAG | A | 457 | 7.595 | 272 | 1 | 1 | 1 |
| 2804113 | - | GACGAG | A | 330 | 5.558 | 285 | 1 | 1 | 1 |
| 2806065 | - | GACGAG | A | 506 | 7.788 | 328 | 0.98 | 0.921 | 1 |
| 2812229 | + | GACGAG | A | 419 | 6.751 | 282 | 1 | 1 | 1 |
| 2812400 | + | GACGAG | A | 473 | 6.579 | 281 | 1 | 1 | 1 |
| 2815336 | + | GACGAG | A | 430 | 6.948 | 294 | 0.985 | 0.931 | 1 |
| 2824642 | - | GACGAG | A | 451 | 8.286 | 281 | 1 | 0.945 | 1 |
| 2825828 | - | GACGAG | A | 335 | 5.971 | 267 | 0.984 | 0.906 | 1 |
| 2828463 | - | GACGAG | A | 316 | 4.541 | 305 | 0.974 | 0.882 | 1 |
| 2830935 | - | GACGAG | A | 403 | 6.507 | 286 | 0.981 | 0.909 | 1 |
| 2831411 | + | GACGAG | A | 332 | 5.454 | 263 | 0.955 | 0.868 | 1 |
| 2834119 | + | GACGAG | A | 394 | 8.891 | 263 | 1 | 0.957 | 1 |
| 2841582 | - | GACGAG | A | 440 | 5.254 | 287 | 1 | 0.938 | 1 |
| 2843014 | - | GACGAG | A | 366 | 5.459 | 288 | 0.939 | 0.851 | 1 |
| 2845887 | - | GACGAG | A | 366 | 7.362 | 255 | 1 | 0.972 | 1 |
| 2849868 | + | GACGAG | A | 378 | 8.915 | 253 | 0.93 | 0.839 | 0.985 |
| 2850003 | - | GACGAG | A | 351 | 6.968 | 255 | 1 | 0.929 | 1 |
| 2851866 | - | GACGAG | A | 377 | 7.284 | 265 | 1 | 0.979 | 1 |
| 2853859 | - | GACGAG | A | 403 | 9.069 | 276 | 1 | 0.953 | 1 |
| 2854114 | - | GACGAG | A | 315 | 4.064 | 284 | 0.938 | 0.853 | 1 |
| 2861457 | + | GACGAG | A | 314 | 5.751 | 268 | 0.922 | 0.832 | 1 |
| 2861565 | + | GACGAG | A | 487 | 9.294 | 269 | 1 | 0.946 | 1 |
| 2862632 | - | GACGAG | A | 492 | 9.083 | 280 | 1 | 0.978 | 1 |
| 2868102 | + | GACGAG | A | 430 | 7.096 | 290 | 0.969 | 0.908 | 1 |
| 2868381 | + | GACGAG | A | 140 | 3.112 | 282 | 0.749 | 0.65 | 0.865 |
| 2870180 | + | GACGAG | A | 345 | 5.605 | 273 | 1 | 0.935 | 1 |
| 2872414 | - | GACGAG | A | 432 | 6.653 | 285 | 1 | 0.934 | 1 |
| 2874016 | - | GACGAG | A | 331 | 5.144 | 267 | 0.975 | 0.894 | 1 |
| 2876948 | + | GACGAG | A | 431 | 9.316 | 264 | 1 | 0.983 | 1 |
| 2879714 | - | GACGAG | A | 361 | 5.041 | 306 | 0.946 | 0.867 | 1 |
| 2882019 | - | GACGAG | A | 477 | 8.189 | 315 | 1 | 1 | 1 |
| 2886378 | - | GACGAG | A | 361 | 5.536 | 295 | 1 | 0.979 | 1 |
| 2887880 | + | GACGAG | A | 208 | 3.841 | 282 | 0.958 | 0.854 | 1 |
| 2887898 | - | GACGAG | A | 372 | 7.371 | 278 | 0.984 | 0.911 | 1 |
| 2888012 | - | GACGAG | A | 296 | 4.798 | 280 | 0.862 | 0.772 | 0.955 |
| 2897630 | - | GACGAG | A | 362 | 5.279 | 263 | 0.953 | 0.868 | 1 |
| 2897894 | + | GACGAG | A | 324 | 6.72 | 248 | 1 | 0.941 | 1 |
| 2898281 | - | GACGAG | A | 281 | 4.733 | 273 | 0.894 | 0.802 | 0.978 |
| 2899134 | + | GACGAG | A | 341 | 8.414 | 244 | 1 | 0.958 | 1 |
| 2905714 | + | GACGAG | A | 359 | 7.645 | 249 | 1 | 0.962 | 1 |
| 2923018 | + | GACGAG | A | 321 | 3.97 | 333 | 0.939 | 0.847 | 1 |
| 2924972 | + | GACGAG | A | 443 | 10.097 | 278 | 1 | 0.981 | 1 |
| 2925511 | + | GACGAG | A | 377 | 6.49 | 291 | 1 | 0.977 | 1 |
| 2928247 | + | GACGAG | A | 402 | 6.45 | 258 | 1 | 0.922 | 1 |
| 2931310 | + | GACGAG | A | 272 | 4.376 | 268 | 1 | 0.948 | 1 |
| 2938620 | - | GACGAG | A | 454 | 10.299 | 275 | 0.985 | 0.93 | 1 |
| 2939231 | - | GACGAG | A | 385 | 7.423 | 266 | 0.985 | 0.915 | 1 |
| 2939634 | - | GACGAG | A | 376 | 10.206 | 264 | 1 | 0.948 | 1 |
| 2947728 | + | GACGAG | A | 434 | 7.581 | 272 | 1 | 1 | 1 |
| 2952714 | - | GACGAG | A | 476 | 7.971 | 293 | 1 | 0.985 | 1 |
| 2956501 | + | GACGAG | A | 390 | 6.931 | 263 | 0.962 | 0.892 | 1 |
| 2956617 | + | GACGAG | A | 407 | 5.506 | 271 | 1 | 1 | 1 |
| 2956872 | - | GACGAG | A | 351 | 6.367 | 274 | 0.985 | 0.91 | 1 |
| 2967302 | + | GACGAG | A | 392 | 4.711 | 347 | 1 | 1 | 1 |
| 2968375 | - | GACGAG | A | 271 | 3.704 | 330 | 1 | 0.981 | 1 |
| 2969208 | - | GACGAG | A | 500 | 7.044 | 323 | 1 | 0.98 | 1 |
| 2974873 | - | GACGAG | A | 495 | 6.169 | 314 | 1 | 0.947 | 1 |
| 2990507 | + | GACGAG | A | 280 | 6.366 | 218 | 0.944 | 0.848 | 1 |
| 2993409 | + | GACGAG | A | 375 | 7.454 | 253 | 0.957 | 0.885 | 1 |
| 3003781 | - | GACGAG | A | 350 | 8.367 | 217 | 1 | 0.967 | 1 |
| 3007280 | - | GACGAG | A | 359 | 8.345 | 231 | 1 | 0.951 | 1 |
| 3009890 | + | GACGAG | A | 410 | 7.8 | 264 | 0.985 | 0.926 | 1 |
| 3011017 | + | GACGAG | A | 503 | 9.114 | 289 | 1 | 1 | 1 |
| 3016245 | + | GACGAG | A | 386 | 5.441 | 296 | 1 | 0.95 | 1 |
| 3017262 | - | GACGAG | A | 377 | 4.243 | 341 | 0.868 | 0.79 | 0.948 |
| 3019838 | + | GACGAG | A | 548 | 9.851 | 324 | 1 | 0.97 | 1 |
| 3024094 | + | GACGAG | A | 474 | 8.341 | 313 | 0.976 | 0.928 | 1 |
| 3026792 | + | GACGAG | A | 476 | 5.797 | 312 | 1 | 0.93 | 1 |
| 3027024 | + | GACGAG | A | 431 | 6.508 | 313 | 0.976 | 0.911 | 1 |
| 3029150 | - | GACGAG | A | 450 | 5.663 | 333 | 0.92 | 0.847 | 0.985 |
| 3032317 | + | GACGAG | A | 418 | 5.535 | 334 | 1 | 0.941 | 1 |
| 3035137 | - | GACGAG | A | 513 | 6.44 | 314 | 1 | 1 | 1 |
| 3036469 | + | GACGAG | A | 266 | 3.538 | 321 | 0.942 | 0.844 | 1 |
| 3040193 | + | GACGAG | A | 416 | 6.952 | 283 | 0.985 | 0.925 | 1 |
| 3044182 | - | GACGAG | A | 502 | 7.303 | 353 | 1 | 0.952 | 1 |
| 3046000 | + | GACGAG | A | 597 | 9.975 | 404 | 1 | 1 | 1 |
| 3050989 | - | GACGAG | A | 501 | 5.798 | 361 | 0.984 | 0.92 | 1 |
| 3056059 | - | GACGAG | A | 448 | 5.624 | 316 | 0.973 | 0.902 | 1 |
| 3058241 | - | GACGAG | A | 483 | 6.272 | 331 | 1 | 0.941 | 1 |
| 3059975 | - | GACGAG | A | 365 | 5.607 | 295 | 0.954 | 0.863 | 1 |
| 3060720 | - | GACGAG | A | 429 | 7.555 | 288 | 0.978 | 0.916 | 1 |
| 3061554 | - | GACGAG | A | 376 | 5.591 | 271 | 0.941 | 0.857 | 1 |
| 3065287 | - | GACGAG | A | 384 | 5.195 | 322 | 0.953 | 0.863 | 1 |
| 3066493 | - | GACGAG | A | 449 | 10.215 | 311 | 0.985 | 0.947 | 1 |
| 3067543 | - | GACGAG | A | 433 | 9.167 | 299 | 1 | 0.957 | 1 |
| 3070314 | - | GACGAG | A | 421 | 9.001 | 306 | 0.985 | 0.939 | 1 |
| 3071164 | + | GACGAG | A | 393 | 7.271 | 281 | 1 | 0.934 | 1 |
| 3071814 | - | GACGAG | A | 353 | 5.451 | 279 | 1 | 0.932 | 1 |
| 3079721 | - | GACGAG | A | 441 | 9.41 | 273 | 1 | 0.98 | 1 |
| 3083702 | - | GACGAG | A | 367 | 9.565 | 244 | 0.985 | 0.937 | 1 |
| 3084971 | + | GACGAG | A | 360 | 6.981 | 257 | 0.95 | 0.882 | 1 |
| 3088502 | - | GACGAG | A | 211 | 4.124 | 212 | 0.987 | 0.867 | 1 |
| 3091513 | + | GACGAG | A | 369 | 8.105 | 251 | 1 | 1 | 1 |
| 3092362 | - | GACGAG | A | 353 | 5.16 | 272 | 0.933 | 0.846 | 1 |
| 3099405 | - | GACGAG | A | 506 | 7.814 | 289 | 1 | 1 | 1 |
| 3101059 | - | GACGAG | A | 494 | 8.456 | 324 | 0.968 | 0.912 | 1 |
| 3101983 | + | GACGAG | A | 493 | 7.908 | 351 | 0.959 | 0.901 | 1 |
| 3102001 | - | GACGAG | A | 463 | 6 | 322 | 1 | 0.956 | 1 |
| 3102220 | + | GACGAG | A | 310 | 4.167 | 347 | 1 | 0.964 | 1 |
| 3102709 | - | GACGAG | A | 441 | 6.282 | 321 | 0.969 | 0.9 | 1 |
| 3107108 | + | GACGAG | A | 471 | 6.588 | 334 | 1 | 0.955 | 1 |
| 3108207 | - | GACGAG | A | 472 | 10.461 | 292 | 0.985 | 0.945 | 1 |
| 3109546 | - | GACGAG | A | 420 | 8.482 | 282 | 1 | 0.967 | 1 |
| 3109930 | - | GACGAG | A | 333 | 5.482 | 282 | 1 | 0.932 | 1 |
| 3119376 | - | GACGAG | A | 188 | 4.498 | 190 | 1 | 0.953 | 1 |
| 3125572 | - | GACGAG | A | 230 | 7.205 | 171 | 0.957 | 0.876 | 1 |
| 3127926 | - | GACGAG | A | 363 | 5.91 | 224 | 0.983 | 0.89 | 1 |
| 3130098 | - | GACGAG | A | 340 | 5.402 | 239 | 1 | 0.949 | 1 |
| 3130843 | + | GACGAG | A | 386 | 7.32 | 248 | 1 | 0.966 | 1 |
| 3131167 | - | GACGAG | A | 408 | 6.585 | 272 | 1 | 1 | 1 |
| 3131206 | + | GACGAG | A | 354 | 6.102 | 272 | 1 | 0.976 | 1 |
| 3133043 | + | GACGAG | A | 289 | 4.863 | 263 | 1 | 0.927 | 1 |
| 3133283 | - | GACGAG | A | 448 | 6.487 | 275 | 1 | 0.943 | 1 |
| 3144042 | + | GACGAG | A | 540 | 7.046 | 360 | 1 | 0.938 | 1 |
| 3145282 | + | GACGAG | A | 637 | 8.6 | 385 | 1 | 0.993 | 1 |
| 3146761 | - | GACGAG | A | 577 | 8.509 | 377 | 1 | 0.969 | 1 |
| 3149167 | - | GACGAG | A | 444 | 5.573 | 347 | 1 | 0.941 | 1 |
| 3151719 | - | GACGAG | A | 443 | 5.995 | 335 | 0.938 | 0.871 | 1 |
| 3151926 | - | GACGAG | A | 415 | 5.149 | 337 | 1 | 0.921 | 1 |
| 3152037 | - | GACGAG | A | 553 | 10.474 | 335 | 1 | 0.973 | 1 |
| 3157358 | + | GACGAG | A | 474 | 7.1 | 331 | 1 | 0.934 | 1 |
| 3160867 | + | GACGAG | A | 338 | 5.054 | 308 | 1 | 0.939 | 1 |
| 3161050 | - | GACGAG | A | 415 | 5.786 | 309 | 1 | 0.946 | 1 |
| 3161995 | - | GACGAG | A | 408 | 7.744 | 291 | 1 | 0.935 | 1 |
| 3162988 | - | GACGAG | A | 384 | 7.357 | 277 | 0.941 | 0.878 | 1 |
| 3163495 | - | GACGAG | A | 421 | 7.692 | 286 | 0.96 | 0.892 | 1 |
| 3167119 | - | GACGAG | A | 278 | 4.936 | 252 | 1 | 0.915 | 1 |
| 3168547 | + | GACGAG | A | 214 | 3.656 | 252 | 0.923 | 0.809 | 1 |
| 3169028 | - | GACGAG | A | 273 | 6.004 | 224 | 0.94 | 0.856 | 1 |
| 3170377 | - | GACGAG | A | 305 | 6.478 | 238 | 0.929 | 0.842 | 1 |
| 3178402 | - | GACGAG | A | 445 | 7.558 | 312 | 0.961 | 0.9 | 1 |
| 3180989 | - | GACGAG | A | 408 | 9.092 | 284 | 0.967 | 0.902 | 1 |
| 3181193 | - | GACGAG | A | 394 | 6.744 | 287 | 0.985 | 0.917 | 1 |
| 3183468 | - | GACGAG | A | 439 | 7.798 | 304 | 0.963 | 0.888 | 1 |
| 3184200 | - | GACGAG | A | 450 | 6.553 | 309 | 0.974 | 0.899 | 1 |
| 3186581 | - | GACGAG | A | 447 | 10.167 | 305 | 0.948 | 0.891 | 1 |
| 3190805 | + | GACGAG | A | 509 | 8.103 | 319 | 1 | 0.979 | 1 |
| 3197294 | - | GACGAG | A | 383 | 7.755 | 223 | 1 | 1 | 1 |
| 3199049 | - | GACGAG | A | 257 | 4.048 | 237 | 1 | 1 | 1 |
| 3204173 | - | GACGAG | A | 506 | 6.983 | 330 | 1 | 0.956 | 1 |
| 3209753 | - | GACGAG | A | 476 | 7.201 | 341 | 0.979 | 0.914 | 1 |
| 3213277 | - | GACGAG | A | 454 | 5.998 | 341 | 0.968 | 0.899 | 1 |
| 3217265 | + | GACGAG | A | 560 | 8.558 | 360 | 1 | 0.985 | 1 |
| 3217550 | + | GACGAG | A | 631 | 12.014 | 351 | 1 | 1 | 1 |
| 3218869 | - | GACGAG | A | 498 | 8.896 | 318 | 0.985 | 0.932 | 1 |
| 3219771 | - | GACGAG | A | 330 | 4.783 | 309 | 0.964 | 0.881 | 1 |
| 3219836 | - | GACGAG | A | 512 | 8.478 | 305 | 1 | 0.977 | 1 |
| 3221564 | - | GACGAG | A | 375 | 5.371 | 309 | 1 | 0.925 | 1 |
| 3221708 | - | GACGAG | A | 478 | 7.432 | 313 | 1 | 0.971 | 1 |
| 3222714 | - | GACGAG | A | 487 | 7.384 | 306 | 1 | 0.983 | 1 |
| 3224051 | - | GACGAG | A | 475 | 7.659 | 313 | 0.94 | 0.88 | 0.985 |
| 3227272 | - | GACGAG | A | 433 | 7.062 | 304 | 1 | 0.957 | 1 |
| 3227740 | - | GACGAG | A | 451 | 8.463 | 305 | 0.985 | 0.948 | 1 |
| 3235822 | - | GACGAG | A | 384 | 6.75 | 274 | 0.98 | 0.905 | 1 |
| 3243757 | + | GACGAG | A | 342 | 4.477 | 306 | 1 | 0.93 | 1 |
| 3246939 | - | GACGAG | A | 481 | 7.249 | 353 | 0.978 | 0.906 | 1 |
| 3248439 | - | GACGAG | A | 411 | 5.164 | 374 | 0.976 | 0.894 | 1 |
| 3249621 | - | GACGAG | A | 490 | 5.704 | 385 | 0.966 | 0.902 | 1 |
| 3250908 | + | GACGAG | A | 524 | 7.967 | 364 | 0.985 | 0.922 | 1 |
| 3255775 | + | GACGAG | A | 319 | 5.422 | 279 | 0.882 | 0.801 | 0.965 |
| 3259648 | + | GACGAG | A | 420 | 7.665 | 287 | 0.985 | 0.924 | 1 |
| 3259988 | - | GACGAG | A | 417 | 10.319 | 287 | 0.938 | 0.873 | 0.985 |
| 3270096 | + | GACGAG | A | 333 | 6.593 | 229 | 1 | 0.953 | 1 |
| 3271501 | - | GACGAG | A | 323 | 7.569 | 207 | 0.956 | 0.873 | 1 |
| 3271882 | - | GACGAG | A | 321 | 7.648 | 206 | 1 | 0.961 | 1 |
| 3271932 | + | GACGAG | A | 354 | 6.978 | 213 | 1 | 0.944 | 1 |
| 3272121 | + | GACGAG | A | 39 | 2.016 | 213 | 0.629 | 0.485 | 0.783 |
| 3275458 | + | GACGAG | A | 233 | 5.74 | 173 | 1 | 0.935 | 1 |
| 3275825 | + | GACGAG | A | 278 | 5.272 | 178 | 0.958 | 0.867 | 1 |
| 3276641 | + | GACGAG | A | 265 | 7.249 | 175 | 0.953 | 0.854 | 1 |
| 3277895 | + | GACGAG | A | 264 | 6.113 | 178 | 0.942 | 0.848 | 1 |
| 3280030 | - | GACGAG | A | 246 | 5.446 | 184 | 0.936 | 0.821 | 1 |
| 3280173 | + | GACGAG | A | 283 | 6.972 | 196 | 0.94 | 0.847 | 1 |
| 3280236 | - | GACGAG | A | 259 | 7.859 | 193 | 0.946 | 0.853 | 1 |
| 3280809 | + | GACGAG | A | 262 | 5.081 | 216 | 0.981 | 0.879 | 1 |
| 3281492 | + | GACGAG | A | 245 | 4.736 | 216 | 0.95 | 0.843 | 1 |
| 3286984 | + | GACGAG | A | 345 | 5.933 | 258 | 0.917 | 0.84 | 1 |
| 3287739 | - | GACGAG | A | 384 | 5.866 | 283 | 0.934 | 0.844 | 0.986 |
| 3290631 | + | GACGAG | A | 450 | 6.91 | 282 | 1 | 0.939 | 1 |
| 3300652 | + | GACGAG | A | 363 | 6.918 | 245 | 1 | 0.952 | 1 |
| 3302707 | + | GACGAG | A | 391 | 9.911 | 211 | 1 | 0.96 | 1 |
| 3306852 | - | GACGAG | A | 484 | 7.984 | 298 | 1 | 0.968 | 1 |
| 3309189 | + | GACGAG | A | 509 | 6.727 | 304 | 1 | 0.952 | 1 |
| 3310667 | + | GACGAG | A | 508 | 8.174 | 345 | 1 | 0.95 | 1 |
| 3315040 | + | GACGAG | A | 493 | 7.213 | 390 | 0.969 | 0.909 | 1 |
| 3324190 | + | GACGAG | A | 454 | 7.419 | 284 | 1 | 0.953 | 1 |
| 3326883 | - | GACGAG | A | 455 | 7.064 | 323 | 0.983 | 0.908 | 1 |
| 3330270 | - | GACGAG | A | 463 | 9.248 | 321 | 0.965 | 0.905 | 1 |
| 3337877 | + | GACGAG | A | 585 | 9.578 | 368 | 1 | 1 | 1 |
| 3342576 | - | GACGAG | A | 559 | 6.638 | 384 | 0.985 | 0.926 | 1 |
| 3345243 | + | GACGAG | A | 517 | 7.065 | 373 | 0.964 | 0.893 | 1 |
| 3345676 | + | GACGAG | A | 586 | 6.775 | 381 | 1 | 1 | 1 |
| 3346452 | + | GACGAG | A | 617 | 7.9 | 372 | 0.985 | 0.939 | 1 |
| 3346530 | + | GACGAG | A | 537 | 5.867 | 375 | 1 | 0.979 | 1 |
| 3346857 | + | GACGAG | A | 512 | 7.518 | 363 | 0.966 | 0.902 | 1 |
| 3351784 | - | GACGAG | A | 407 | 6.102 | 313 | 0.962 | 0.889 | 1 |
| 3356415 | - | GACGAG | A | 407 | 5.191 | 329 | 1 | 0.943 | 1 |
| 3358109 | - | GACGAG | A | 466 | 8.398 | 342 | 0.964 | 0.903 | 1 |
| 3358908 | + | GACGAG | A | 475 | 6.474 | 355 | 0.945 | 0.877 | 1 |
| 3364153 | - | GACGAG | A | 475 | 6.901 | 328 | 0.958 | 0.889 | 1 |
| 3364795 | - | GACGAG | A | 365 | 4.398 | 337 | 1 | 0.949 | 1 |
| 3376194 | + | GACGAG | A | 454 | 7.495 | 315 | 1 | 0.949 | 1 |
| 3376874 | + | GACGAG | A | 518 | 9.08 | 340 | 0.985 | 0.939 | 1 |
| 3378890 | + | GACGAG | A | 454 | 8.541 | 325 | 0.948 | 0.883 | 1 |
| 3379647 | - | GACGAG | A | 403 | 4.861 | 318 | 0.981 | 0.913 | 1 |
| 3379931 | + | GACGAG | A | 486 | 8.11 | 338 | 0.933 | 0.873 | 0.985 |
| 3387710 | + | GACGAG | A | 434 | 4.877 | 357 | 1 | 0.949 | 1 |
| 3388761 | + | GACGAG | A | 563 | 8.353 | 363 | 1 | 0.951 | 1 |
| 3389674 | + | GACGAG | A | 507 | 5.776 | 369 | 1 | 0.956 | 1 |
| 3389932 | + | GACGAG | A | 514 | 7.806 | 356 | 1 | 0.952 | 1 |
| 3396161 | - | GACGAG | A | 494 | 7.957 | 309 | 1 | 0.954 | 1 |
| 3400378 | - | GACGAG | A | 445 | 4.921 | 361 | 0.971 | 0.892 | 1 |
| 3404954 | + | GACGAG | A | 538 | 7.657 | 381 | 0.979 | 0.928 | 1 |
| 3406815 | - | GACGAG | A | 477 | 7.712 | 325 | 1 | 1 | 1 |
| 3410067 | + | GACGAG | A | 139 | 3.136 | 272 | 0.77 | 0.657 | 0.874 |
| 3413489 | + | GACGAG | A | 390 | 8.486 | 247 | 0.999 | 0.985 | 1 |
| 3418997 | - | GACGAG | A | 408 | 5.757 | 307 | 1 | 0.985 | 1 |
| 3421280 | + | GACGAG | A | 377 | 5.836 | 265 | 1 | 0.926 | 1 |
| 3426128 | - | GACGAG | A | 367 | 6.31 | 298 | 0.907 | 0.839 | 0.978 |
| 3436264 | - | GACGAG | A | 438 | 8.029 | 283 | 0.943 | 0.872 | 1 |
| 3445579 | - | GACGAG | A | 365 | 4.731 | 309 | 1 | 0.93 | 1 |
| 3451826 | - | GACGAG | A | 446 | 4.736 | 324 | 0.982 | 0.897 | 1 |
| 3454862 | - | GACGAG | A | 427 | 8.289 | 311 | 0.913 | 0.846 | 0.984 |
| 3464308 | - | GACGAG | A | 273 | 3.764 | 309 | 0.952 | 0.849 | 1 |
| 3467829 | - | GACGAG | A | 456 | 5.529 | 333 | 1 | 1 | 1 |
| 3468356 | - | GACGAG | A | 478 | 5.6 | 348 | 0.966 | 0.898 | 1 |
| 3470082 | - | GACGAG | A | 490 | 7.706 | 371 | 0.985 | 0.944 | 1 |
| 3470506 | + | GACGAG | A | 460 | 6.976 | 336 | 0.953 | 0.886 | 1 |
| 3476327 | + | GACGAG | A | 460 | 5.43 | 323 | 1 | 0.985 | 1 |
| 3476379 | - | GACGAG | A | 415 | 7.847 | 308 | 0.941 | 0.875 | 1 |
| 3481124 | + | GACGAG | A | 356 | 5.492 | 294 | 1 | 0.968 | 1 |
| 3501471 | - | GACGAG | A | 263 | 6.617 | 188 | 0.951 | 0.856 | 1 |
| 3504968 | + | GACGAG | A | 240 | 4.642 | 274 | 0.964 | 0.866 | 1 |
| 3507134 | + | GACGAG | A | 337 | 4.037 | 331 | 0.971 | 0.88 | 1 |
| 3507147 | - | GACGAG | A | 440 | 6.542 | 308 | 0.974 | 0.907 | 1 |
| 3513323 | - | GACGAG | A | 520 | 6.326 | 335 | 1 | 0.954 | 1 |
| 3513835 | - | GACGAG | A | 442 | 5.504 | 355 | 1 | 0.977 | 1 |
| 3515908 | - | GACGAG | A | 503 | 7.208 | 347 | 0.955 | 0.894 | 1 |
| 3519560 | + | GACGAG | A | 503 | 7.291 | 353 | 1 | 0.958 | 1 |
| 3524248 | + | GACGAG | A | 357 | 4.466 | 309 | 1 | 0.944 | 1 |
| 3528269 | - | GACGAG | A | 356 | 7.449 | 260 | 0.985 | 0.919 | 1 |
| 3529736 | - | GACGAG | A | 350 | 7.754 | 246 | 0.96 | 0.884 | 1 |
| 3531204 | - | GACGAG | A | 345 | 5.917 | 254 | 0.94 | 0.855 | 1 |
| 3545300 | - | GACGAG | A | 267 | 3.874 | 284 | 0.857 | 0.741 | 0.947 |
| 3546789 | - | GACGAG | A | 530 | 7.74 | 304 | 0.97 | 0.912 | 1 |
| 3550008 | - | GACGAG | A | 419 | 7.427 | 309 | 0.967 | 0.904 | 1 |
| 3552073 | - | GACGAG | A | 516 | 7.715 | 337 | 0.985 | 0.93 | 1 |
| 3555395 | - | GACGAG | A | 476 | 7.009 | 358 | 0.959 | 0.891 | 1 |
| 3557723 | - | GACGAG | A | 397 | 5.06 | 352 | 0.996 | 0.995 | 0.998 |
| 3558193 | + | GACGAG | A | 239 | 3.701 | 359 | 0.867 | 0.786 | 0.957 |
| 3562249 | + | GACGAG | A | 430 | 5.042 | 311 | 1 | 0.937 | 1 |
| 3563693 | + | GACGAG | A | 511 | 8.638 | 296 | 0.958 | 0.891 | 1 |
| 3573073 | + | GACGAG | A | 485 | 6.758 | 329 | 0.985 | 0.932 | 1 |
| 3582090 | - | GACGAG | A | 423 | 5.916 | 309 | 1 | 0.984 | 1 |
| 3583381 | - | GACGAG | A | 509 | 9.066 | 313 | 1 | 0.948 | 1 |
| 3585556 | - | GACGAG | A | 392 | 5.777 | 311 | 0.93 | 0.852 | 0.985 |
| 3590796 | - | GACGAG | A | 435 | 8.378 | 310 | 0.98 | 0.917 | 1 |
| 3594732 | - | GACGAG | A | 401 | 5.806 | 358 | 0.999 | 0.929 | 1 |
| 3602864 | + | GACGAG | A | 441 | 6.475 | 336 | 0.983 | 0.911 | 1 |
| 3605704 | + | GACGAG | A | 461 | 8.186 | 293 | 1 | 0.938 | 1 |
| 3609215 | - | GACGAG | A | 170 | 2.743 | 389 | 0.83 | 0.737 | 0.933 |
| 3611827 | - | GACGAG | A | 488 | 5.402 | 398 | 0.974 | 0.903 | 1 |
| 3620655 | - | GACGAG | A | 541 | 10.272 | 345 | 1 | 0.954 | 1 |
| 3624270 | - | GACGAG | A | 545 | 5.99 | 376 | 1 | 0.983 | 1 |
| 3626464 | - | GACGAG | A | 532 | 7.138 | 353 | 1 | 1 | 1 |
| 3630578 | - | GACGAG | A | 547 | 6.539 | 410 | 0.97 | 0.91 | 1 |
| 3633048 | - | GACGAG | A | 356 | 3.805 | 429 | 0.909 | 0.825 | 0.983 |
| 3637985 | + | GACGAG | A | 555 | 9.187 | 395 | 0.985 | 0.938 | 1 |
| 3646770 | + | GACGAG | A | 601 | 6.867 | 436 | 0.985 | 0.931 | 1 |
| 3649776 | + | GACGAG | A | 627 | 7.601 | 393 | 1 | 0.985 | 1 |
| 3653227 | - | GACGAG | A | 546 | 8.252 | 361 | 0.95 | 0.889 | 1 |
| 3655337 | - | GACGAG | A | 338 | 4.807 | 382 | 0.983 | 0.902 | 1 |
| 3656191 | - | GACGAG | A | 589 | 6.876 | 384 | 1 | 0.985 | 1 |
| 3663346 | + | GACGAG | A | 569 | 7.142 | 376 | 0.983 | 0.921 | 1 |
| 3663957 | + | GACGAG | A | 517 | 7.062 | 400 | 1 | 0.956 | 1 |
| 3668169 | + | GACGAG | A | 295 | 3.775 | 394 | 0.938 | 0.857 | 1 |
| 3671189 | + | GACGAG | A | 312 | 4.408 | 417 | 1 | 1 | 1 |
| 3671384 | + | GACGAG | A | 619 | 8.878 | 413 | 0.975 | 0.923 | 1 |
| 3673561 | - | GACGAG | A | 585 | 6.165 | 398 | 1 | 0.941 | 1 |
| 3674091 | + | GACGAG | A | 530 | 5.45 | 400 | 1 | 1 | 1 |
| 3674344 | + | GACGAG | A | 466 | 4.586 | 403 | 0.941 | 0.865 | 1 |
| 3681310 | + | GACGAG | A | 371 | 5.229 | 357 | 1 | 0.963 | 1 |
| 3682463 | - | GACGAG | A | 132 | 2.808 | 350 | 0.793 | 0.682 | 0.887 |
| 3683868 | - | GACGAG | A | 532 | 6.929 | 357 | 0.957 | 0.888 | 1 |
| 3684102 | - | GACGAG | A | 517 | 8.647 | 352 | 1 | 0.966 | 1 |
| 3685436 | - | GACGAG | A | 351 | 4.423 | 333 | 0.985 | 0.982 | 1 |
| 3687400 | + | GACGAG | A | 555 | 8.496 | 331 | 1 | 0.963 | 1 |
| 3694872 | + | GACGAG | A | 373 | 6.07 | 280 | 1 | 1 | 1 |
| 3702877 | - | GACGAG | A | 499 | 7.19 | 313 | 1 | 0.969 | 1 |
| 3706383 | - | GACGAG | A | 313 | 4.556 | 415 | 1 | 1 | 1 |
| 3708521 | - | GACGAG | A | 164 | 2.883 | 383 | 0.822 | 0.724 | 0.922 |
| 3710949 | - | GACGAG | A | 585 | 7.999 | 414 | 0.984 | 0.925 | 1 |
| 3718430 | - | GACGAG | A | 413 | 5.053 | 336 | 0.963 | 0.881 | 1 |
| 3720306 | - | GACGAG | A | 293 | 4.389 | 291 | 0.958 | 0.852 | 1 |
| 3724156 | - | GACGAG | A | 346 | 7.444 | 247 | 1 | 0.923 | 1 |
| 3724672 | - | GACGAG | A | 394 | 7.091 | 254 | 0.984 | 0.91 | 1 |
| 3727078 | - | GACGAG | A | 425 | 6.786 | 288 | 1 | 0.985 | 1 |
| 3727492 | - | GACGAG | A | 344 | 5.177 | 295 | 0.937 | 0.864 | 1 |
| 3727915 | - | GACGAG | A | 425 | 6.717 | 291 | 1 | 0.935 | 1 |
| 3734620 | - | GACGAG | A | 351 | 4.317 | 330 | 0.954 | 0.875 | 1 |
| 3736447 | - | GACGAG | A | 490 | 6.896 | 369 | 0.939 | 0.877 | 1 |
| 3739778 | + | GACGAG | A | 441 | 5.369 | 349 | 1 | 0.951 | 1 |
| 3740539 | - | GACGAG | A | 383 | 4.368 | 352 | 0.959 | 0.88 | 1 |
| 3742168 | + | GACGAG | A | 355 | 4.431 | 365 | 0.967 | 0.877 | 1 |
| 3743666 | - | GACGAG | A | 638 | 9.905 | 394 | 1 | 0.983 | 1 |
| 3745202 | - | GACGAG | A | 606 | 9.435 | 371 | 0.985 | 0.932 | 1 |
| 3747265 | + | GACGAG | A | 534 | 7.618 | 367 | 1 | 0.946 | 1 |
| 3750391 | - | GACGAG | A | 506 | 7.001 | 362 | 0.964 | 0.903 | 1 |
| 3751794 | - | GACGAG | A | 431 | 6.447 | 349 | 1 | 0.936 | 1 |
| 3753531 | + | GACGAG | A | 424 | 5.199 | 333 | 0.939 | 0.869 | 1 |
| 3755282 | + | GACGAG | A | 447 | 7.548 | 337 | 0.964 | 0.901 | 1 |
| 3755799 | - | GACGAG | A | 194 | 3.071 | 354 | 0.889 | 0.794 | 0.984 |
| 3760192 | + | GACGAG | A | 514 | 7.501 | 377 | 0.963 | 0.905 | 1 |
| 3763219 | + | GACGAG | A | 568 | 11.086 | 343 | 1 | 0.976 | 1 |
| 3766085 | - | GACGAG | A | 527 | 9.154 | 352 | 0.948 | 0.89 | 1 |
| 3773304 | + | GACGAG | A | 598 | 6.285 | 425 | 0.985 | 0.933 | 1 |
| 3774588 | - | GACGAG | A | 587 | 6.559 | 408 | 0.984 | 0.928 | 1 |
| 3780969 | - | GACGAG | A | 571 | 7.505 | 392 | 1 | 1 | 1 |
| 3781314 | - | GACGAG | A | 248 | 3.238 | 385 | 0.834 | 0.747 | 0.921 |
| 3781575 | - | GACGAG | A | 561 | 9.661 | 362 | 1 | 0.957 | 1 |
| 3782714 | - | GACGAG | A | 378 | 4.678 | 374 | 0.914 | 0.829 | 0.987 |
| 3788645 | - | GACGAG | A | 473 | 4.939 | 420 | 1 | 0.952 | 1 |
| 3790373 | - | GACGAG | A | 574 | 9.308 | 408 | 0.984 | 0.927 | 1 |
| 3791625 | - | GACGAG | A | 564 | 8.218 | 371 | 1 | 0.968 | 1 |
| 3792397 | - | GACGAG | A | 380 | 5.083 | 340 | 0.954 | 0.871 | 1 |
| 3792708 | + | GACGAG | A | 501 | 9.613 | 340 | 1 | 0.984 | 1 |
| 3793103 | + | GACGAG | A | 466 | 9.138 | 314 | 0.979 | 0.924 | 1 |
| 3800589 | + | GACGAG | A | 408 | 8.712 | 265 | 0.977 | 0.91 | 1 |
| 3803823 | - | GACGAG | A | 479 | 6.838 | 357 | 0.985 | 0.922 | 1 |
| 3804193 | + | GACGAG | A | 470 | 6.081 | 362 | 0.977 | 0.907 | 1 |
| 3809731 | + | GACGAG | A | 566 | 6.975 | 400 | 1 | 0.952 | 1 |
| 3811472 | + | GACGAG | A | 637 | 7.435 | 437 | 1 | 0.985 | 1 |
| 3817872 | + | GACGAG | A | 712 | 11.784 | 413 | 1 | 1 | 1 |
| 3819437 | + | GACGAG | A | 549 | 5.494 | 422 | 1 | 0.984 | 1 |
| 3821244 | + | GACGAG | A | 604 | 8.375 | 411 | 1 | 0.949 | 1 |
| 3824736 | - | GACGAG | A | 626 | 8.869 | 419 | 0.985 | 0.942 | 1 |
| 3828009 | - | GACGAG | A | 653 | 9.59 | 411 | 0.985 | 0.952 | 1 |
| 3830767 | - | GACGAG | A | 543 | 6.44 | 400 | 0.934 | 0.875 | 0.985 |
| 3832961 | - | GACGAG | A | 539 | 6.39 | 399 | 1 | 0.957 | 1 |
| 3847517 | - | GACGAG | A | 585 | 7.038 | 406 | 1 | 0.962 | 1 |
| 3851337 | - | GACGAG | A | 575 | 7.366 | 425 | 0.982 | 0.917 | 1 |
| 3853375 | - | GACGAG | A | 378 | 3.798 | 395 | 0.907 | 0.829 | 0.976 |
| 3860612 | + | GACGAG | A | 669 | 7.993 | 433 | 1 | 0.982 | 1 |
| 3862012 | + | GACGAG | A | 564 | 7.121 | 433 | 0.969 | 0.916 | 1 |
| 3862960 | + | GACGAG | A | 566 | 6.784 | 423 | 0.951 | 0.893 | 1 |
| 3865574 | - | GACGAG | A | 190 | 4.458 | 339 | 1 | 1 | 1 |
| 3874364 | - | GACGAG | A | 444 | 6.937 | 337 | 0.963 | 0.897 | 1 |
| 3875055 | - | GACGAG | A | 333 | 3.894 | 351 | 1 | 0.953 | 1 |
| 3876110 | + | GACGAG | A | 546 | 9.342 | 367 | 0.969 | 0.91 | 1 |
| 3876590 | + | GACGAG | A | 462 | 5.585 | 389 | 0.977 | 0.912 | 1 |
| 3881921 | - | GACGAG | A | 54 | 2.794 | 441 | 0.866 | 0.766 | 0.968 |
| 3883179 | - | GACGAG | A | 382 | 3.978 | 438 | 0.921 | 0.844 | 0.986 |
| 3887114 | - | GACGAG | A | 592 | 5.24 | 477 | 0.985 | 0.931 | 1 |
| 3889215 | + | GACGAG | A | 611 | 5.897 | 492 | 0.937 | 0.879 | 0.985 |
| 3889262 | + | GACGAG | A | 571 | 5.748 | 493 | 1 | 0.959 | 1 |
| 3890303 | - | GACGAG | A | 658 | 5.824 | 499 | 1 | 0.983 | 1 |
| 3891462 | - | GACGAG | A | 744 | 7.983 | 521 | 0.984 | 0.935 | 1 |
| 3894121 | + | GACGAG | A | 609 | 5.735 | 480 | 0.969 | 0.902 | 1 |
| 3898142 | + | GACGAG | A | 551 | 7.41 | 402 | 0.972 | 0.913 | 1 |
| 3900048 | - | GACGAG | A | 449 | 4.704 | 395 | 1 | 1 | 1 |
| 3903357 | + | GACGAG | A | 567 | 6.007 | 406 | 1 | 0.985 | 1 |
| 3907900 | - | GACGAG | A | 506 | 7.543 | 345 | 0.962 | 0.904 | 1 |
| 3908611 | - | GACGAG | A | 449 | 5.357 | 362 | 0.975 | 0.898 | 1 |
| 3912913 | + | GACGAG | A | 365 | 4.272 | 429 | 0.982 | 0.894 | 1 |
| 3914924 | - | GACGAG | A | 439 | 4.74 | 413 | 0.984 | 0.909 | 1 |
| 3926740 | - | GACGAG | A | 569 | 7.053 | 405 | 0.985 | 0.925 | 1 |
| 3928736 | - | GACGAG | A | 549 | 6.535 | 420 | 0.932 | 0.873 | 0.985 |
| 3932918 | + | GACGAG | A | 614 | 6.278 | 478 | 0.974 | 0.909 | 1 |
| 3933825 | - | GACGAG | A | 666 | 8.394 | 483 | 0.972 | 0.923 | 1 |
| 3935269 | + | GACGAG | A | 685 | 9.481 | 476 | 0.983 | 0.939 | 1 |
| 3935727 | + | GACGAG | A | 660 | 8.996 | 474 | 1 | 0.959 | 1 |
| 3941397 | + | GACGAG | A | 452 | 7.366 | 322 | 1 | 0.972 | 1 |
| 3943243 | + | GACGAG | A | 449 | 9.177 | 283 | 1 | 0.969 | 1 |
| 3943785 | + | GACGAG | A | 356 | 5.217 | 284 | 0.911 | 0.828 | 0.985 |
| 3946150 | + | GACGAG | A | 306 | 4.476 | 272 | 0.92 | 0.823 | 1 |
| 3947493 | + | GACGAG | A | 200 | 3.639 | 291 | 0.926 | 0.82 | 1 |
| 3947709 | - | GACGAG | A | 421 | 8.079 | 292 | 0.944 | 0.886 | 1 |
| 3951750 | - | GACGAG | A | 494 | 7.71 | 314 | 1 | 0.984 | 1 |
| 3952400 | - | GACGAG | A | 451 | 6.8 | 312 | 0.985 | 0.921 | 1 |
| 3956094 | + | GACGAG | A | 296 | 3.95 | 349 | 0.889 | 0.798 | 0.977 |
| 3957101 | + | GACGAG | A | 518 | 8.191 | 356 | 0.971 | 0.906 | 1 |
| 3960272 | - | GACGAG | A | 416 | 5.806 | 331 | 0.92 | 0.842 | 0.985 |
| 3963681 | + | GACGAG | A | 450 | 8.179 | 287 | 0.958 | 0.9 | 1 |
| 3964725 | + | GACGAG | A | 402 | 7.247 | 295 | 0.976 | 0.904 | 1 |
| 3964970 | - | GACGAG | A | 318 | 4.09 | 298 | 0.979 | 0.888 | 1 |
| 3969675 | + | GACGAG | A | 438 | 5.555 | 327 | 0.954 | 0.88 | 1 |
| 3971837 | + | GACGAG | A | 463 | 5.193 | 398 | 0.963 | 0.893 | 1 |
| 3978806 | + | GACGAG | A | 564 | 7.532 | 421 | 1 | 1 | 1 |
| 3980919 | + | GACGAG | A | 576 | 6.766 | 415 | 1 | 0.96 | 1 |
| 3982199 | + | GACGAG | A | 430 | 4.392 | 429 | 0.957 | 0.878 | 1 |
| 3982887 | - | GACGAG | A | 570 | 8.505 | 423 | 0.978 | 0.929 | 1 |
| 3988267 | + | GACGAG | A | 506 | 7.794 | 336 | 0.985 | 0.922 | 1 |
| 3988796 | - | GACGAG | A | 483 | 5.457 | 347 | 0.952 | 0.881 | 1 |
| 3989283 | - | GACGAG | A | 528 | 7.846 | 331 | 1 | 0.998 | 1 |
| 3995390 | - | GACGAG | A | 146 | 3.466 | 238 | 0.868 | 0.74 | 0.983 |
| 3996625 | + | GACGAG | A | 314 | 10.203 | 229 | 0.98 | 0.899 | 1 |
| 3997429 | + | GACGAG | A | 342 | 10.928 | 212 | 1 | 0.985 | 1 |
| 4009444 | + | GACGAG | A | 485 | 9.021 | 317 | 1 | 0.945 | 1 |
| 4009835 | + | GACGAG | A | 352 | 4.553 | 318 | 0.94 | 0.851 | 1 |
| 4013412 | - | GACGAG | A | 608 | 7.517 | 403 | 0.985 | 0.924 | 1 |
| 4022146 | + | GACGAG | A | 435 | 5.546 | 331 | 0.967 | 0.894 | 1 |
| 4024124 | - | GACGAG | A | 160 | 3.697 | 304 | 1 | 1 | 1 |

Position indicates the genomic position where the m6A site occurs. Modification quality values (ModQV), IPD ratios, and sequencing coverage at the adenosine residue within the m6A site are given. The fraction of sequencing reads called as modified (frac) is provided as well as the lower (CI low) and upper limits (CI high) of the 95% confidence interval for the fraction of reads called as modified.
