# Supplementary material for: Methyltransferase DnmA is responsible for genome-wide N6-methyladenosine modifications at non-palindromic recognition sites in Bacillus subtilis
Source: Nucleic Acids Res. 2020 Apr 23;48(10):5332–48. doi: 10.1093/nar/gkaa266 (PMC7261158; doi:10.1093/nar/gkaa266)
Supplement: gkaa266_Supplemental_Files [file gkaa266_supplemental_files.zip › Table S8_dnmA.docx]

**Table S8. PacBio SMRT sequencing at GACGAG motifs in Δ*dnmA* in PY79.**

| **position** | **strand** | **motif** | **base** | **ModQV** | **IPDRatio** | **coverage** | **frac** | **CI low** | **CI high** |
| --- | --- | --- | --- | --- | --- | --- | --- | --- | --- |
| 3690 | - | GACGAG | A | 9 | 1.254 | 88 | NA | NA | NA |
| 13073 | - | GACGAG | A | 1 | 0.878 | 140 | NA | NA | NA |
| 20347 | + | GACGAG | A | 4 | 1.047 | 137 | NA | NA | NA |
| 20813 | - | GACGAG | A | 8 | 1.167 | 140 | NA | NA | NA |
| 21392 | - | GACGAG | A | 4 | 1.022 | 144 | NA | NA | NA |
| 33541 | - | GACGAG | A | 7 | 1.123 | 143 | NA | NA | NA |
| 36413 | + | GACGAG | A | 3 | 0.994 | 150 | NA | NA | NA |
| 39739 | - | GACGAG | A | 1 | 0.782 | 152 | NA | NA | NA |
| 44114 | - | GACGAG | A | 12 | 1.213 | 146 | NA | NA | NA |
| 45584 | - | GACGAG | A | 13 | 1.256 | 150 | NA | NA | NA |
| 45793 | + | GACGAG | A | 6 | 1.116 | 148 | NA | NA | NA |
| 50231 | + | GACGAG | A | 0 | 0.832 | 155 | NA | NA | NA |
| 52923 | + | GACGAG | A | 0 | 0.822 | 158 | NA | NA | NA |
| 63092 | - | GACGAG | A | 3 | 1.002 | 166 | NA | NA | NA |
| 64617 | - | GACGAG | A | 10 | 1.196 | 155 | NA | NA | NA |
| 73482 | + | GACGAG | A | 2 | 0.963 | 152 | NA | NA | NA |
| 73830 | + | GACGAG | A | 14 | 1.279 | 155 | NA | NA | NA |
| 76096 | + | GACGAG | A | 0 | 0.691 | 156 | NA | NA | NA |
| 78129 | - | GACGAG | A | 11 | 1.252 | 142 | NA | NA | NA |
| 83701 | + | GACGAG | A | 8 | 1.137 | 149 | NA | NA | NA |
| 83750 | + | GACGAG | A | 5 | 1.067 | 152 | NA | NA | NA |
| 86284 | + | GACGAG | A | 5 | 1.086 | 149 | NA | NA | NA |
| 88555 | + | GACGAG | A | 10 | 1.233 | 146 | NA | NA | NA |
| 93616 | - | GACGAG | A | 2 | 0.958 | 106 | NA | NA | NA |
| 99471 | - | GACGAG | A | 2 | 0.937 | 133 | NA | NA | NA |
| 109428 | - | GACGAG | A | 6 | 1.082 | 184 | NA | NA | NA |
| 109695 | - | GACGAG | A | 19 | 1.364 | 187 | NA | NA | NA |
| 115576 | + | GACGAG | A | 13 | 1.215 | 167 | NA | NA | NA |
| 116658 | + | GACGAG | A | 3 | 1 | 166 | NA | NA | NA |
| 117114 | - | GACGAG | A | 1 | 0.881 | 164 | NA | NA | NA |
| 123652 | - | GACGAG | A | 3 | 1.004 | 175 | NA | NA | NA |
| 129028 | + | GACGAG | A | 1 | 0.896 | 161 | NA | NA | NA |
| 130480 | - | GACGAG | A | 5 | 1.084 | 144 | NA | NA | NA |
| 131396 | + | GACGAG | A | 8 | 1.151 | 157 | NA | NA | NA |
| 133103 | - | GACGAG | A | 2 | 0.964 | 141 | NA | NA | NA |
| 138303 | - | GACGAG | A | 1 | 0.891 | 139 | NA | NA | NA |
| 139198 | - | GACGAG | A | 2 | 0.929 | 164 | NA | NA | NA |
| 140341 | - | GACGAG | A | 10 | 1.196 | 164 | NA | NA | NA |
| 142560 | - | GACGAG | A | 0 | 0.762 | 157 | NA | NA | NA |
| 143000 | - | GACGAG | A | 8 | 1.164 | 151 | NA | NA | NA |
| 147262 | + | GACGAG | A | 8 | 1.149 | 172 | NA | NA | NA |
| 150508 | + | GACGAG | A | 3 | 1.008 | 155 | NA | NA | NA |
| 154659 | - | GACGAG | A | 15 | 1.28 | 141 | NA | NA | NA |
| 157813 | - | GACGAG | A | 1 | 0.839 | 106 | NA | NA | NA |
| 163972 | - | GACGAG | A | 5 | 1.068 | 138 | NA | NA | NA |
| 169581 | - | GACGAG | A | 15 | 1.245 | 191 | NA | NA | NA |
| 174583 | - | GACGAG | A | 5 | 1.061 | 185 | NA | NA | NA |
| 181545 | + | GACGAG | A | 0 | 0.797 | 122 | NA | NA | NA |
| 188147 | + | GACGAG | A | 4 | 1.028 | 143 | NA | NA | NA |
| 190264 | + | GACGAG | A | 6 | 1.086 | 140 | NA | NA | NA |
| 190648 | + | GACGAG | A | 2 | 0.95 | 129 | NA | NA | NA |
| 194200 | + | GACGAG | A | 4 | 1.019 | 157 | NA | NA | NA |
| 196213 | + | GACGAG | A | 0 | 0.781 | 152 | NA | NA | NA |
| 199751 | - | GACGAG | A | 0 | 0.769 | 155 | NA | NA | NA |
| 200073 | + | GACGAG | A | 12 | 1.231 | 161 | NA | NA | NA |
| 201564 | - | GACGAG | A | 0 | 0.783 | 151 | NA | NA | NA |
| 206814 | - | GACGAG | A | 5 | 1.079 | 154 | NA | NA | NA |
| 216823 | - | GACGAG | A | 1 | 0.867 | 112 | NA | NA | NA |
| 220650 | + | GACGAG | A | 3 | 0.994 | 142 | NA | NA | NA |
| 230504 | - | GACGAG | A | 5 | 1.085 | 140 | NA | NA | NA |
| 230668 | - | GACGAG | A | 20 | 1.379 | 142 | NA | NA | NA |
| 236779 | - | GACGAG | A | 8 | 1.161 | 113 | NA | NA | NA |
| 238946 | + | GACGAG | A | 45 | 1.792 | 169 | NA | NA | NA |
| 242685 | + | GACGAG | A | 30 | 1.532 | 197 | NA | NA | NA |
| 244718 | - | GACGAG | A | 8 | 1.141 | 162 | NA | NA | NA |
| 245873 | - | GACGAG | A | 6 | 1.107 | 160 | NA | NA | NA |
| 262966 | + | GACGAG | A | 4 | 1.038 | 178 | NA | NA | NA |
| 268418 | - | GACGAG | A | 3 | 1.009 | 160 | NA | NA | NA |
| 268589 | + | GACGAG | A | 2 | 0.942 | 165 | NA | NA | NA |
| 271446 | - | GACGAG | A | 5 | 1.075 | 149 | NA | NA | NA |
| 274221 | + | GACGAG | A | 1 | 0.864 | 165 | NA | NA | NA |
| 277057 | - | GACGAG | A | 6 | 1.103 | 191 | NA | NA | NA |
| 277900 | - | GACGAG | A | 10 | 1.195 | 193 | NA | NA | NA |
| 280244 | + | GACGAG | A | 6 | 1.091 | 189 | NA | NA | NA |
| 293836 | - | GACGAG | A | 3 | 0.985 | 188 | NA | NA | NA |
| 298091 | - | GACGAG | A | 42 | 1.749 | 161 | 0.336 | 0.2 | 0.448 |
| 302183 | - | GACGAG | A | 6 | 1.09 | 184 | NA | NA | NA |
| 309359 | - | GACGAG | A | 17 | 1.338 | 181 | NA | NA | NA |
| 310037 | + | GACGAG | A | 4 | 1.029 | 159 | NA | NA | NA |
| 312459 | + | GACGAG | A | 13 | 1.233 | 178 | NA | NA | NA |
| 314343 | - | GACGAG | A | 11 | 1.204 | 172 | NA | NA | NA |
| 315010 | - | GACGAG | A | 4 | 1.049 | 147 | NA | NA | NA |
| 318021 | + | GACGAG | A | 5 | 1.094 | 117 | NA | NA | NA |
| 318279 | + | GACGAG | A | 14 | 1.367 | 113 | NA | NA | NA |
| 318577 | - | GACGAG | A | 6 | 1.102 | 120 | NA | NA | NA |
| 318802 | - | GACGAG | A | 11 | 1.239 | 120 | NA | NA | NA |
| 319099 | - | GACGAG | A | 11 | 1.208 | 118 | NA | NA | NA |
| 331206 | - | GACGAG | A | 3 | 0.991 | 99 | NA | NA | NA |
| 336867 | - | GACGAG | A | 11 | 1.221 | 158 | NA | NA | NA |
| 338984 | + | GACGAG | A | 2 | 0.928 | 145 | NA | NA | NA |
| 342214 | + | GACGAG | A | 6 | 1.096 | 143 | NA | NA | NA |
| 342344 | - | GACGAG | A | 9 | 1.189 | 146 | NA | NA | NA |
| 343048 | + | GACGAG | A | 8 | 1.174 | 142 | NA | NA | NA |
| 350996 | + | GACGAG | A | 3 | 0.979 | 154 | NA | NA | NA |
| 352690 | - | GACGAG | A | 4 | 1.038 | 163 | NA | NA | NA |
| 356863 | + | GACGAG | A | 14 | 1.254 | 163 | NA | NA | NA |
| 358089 | - | GACGAG | A | 10 | 1.162 | 170 | NA | NA | NA |
| 359570 | - | GACGAG | A | 3 | 0.993 | 156 | NA | NA | NA |
| 359695 | + | GACGAG | A | 1 | 0.891 | 153 | NA | NA | NA |
| 361730 | - | GACGAG | A | 2 | 0.939 | 170 | NA | NA | NA |
| 363400 | - | GACGAG | A | 4 | 1.029 | 191 | NA | NA | NA |
| 372559 | + | GACGAG | A | 0 | 0.725 | 88 | NA | NA | NA |
| 373764 | - | GACGAG | A | 1 | 0.828 | 102 | NA | NA | NA |
| 375766 | + | GACGAG | A | 2 | 0.927 | 128 | NA | NA | NA |
| 382901 | + | GACGAG | A | 2 | 0.959 | 217 | NA | NA | NA |
| 385001 | + | GACGAG | A | 21 | 1.37 | 202 | NA | NA | NA |
| 385430 | - | GACGAG | A | 8 | 1.148 | 179 | NA | NA | NA |
| 389636 | - | GACGAG | A | 0 | 0.795 | 162 | NA | NA | NA |
| 391220 | - | GACGAG | A | 4 | 1.05 | 153 | NA | NA | NA |
| 392072 | + | GACGAG | A | 4 | 1.021 | 176 | NA | NA | NA |
| 395765 | + | GACGAG | A | 14 | 1.285 | 182 | NA | NA | NA |
| 396194 | - | GACGAG | A | 8 | 1.159 | 170 | NA | NA | NA |
| 398444 | + | GACGAG | A | 2 | 0.974 | 201 | NA | NA | NA |
| 403756 | - | GACGAG | A | 3 | 0.984 | 137 | NA | NA | NA |
| 403921 | - | GACGAG | A | 7 | 1.157 | 133 | NA | NA | NA |
| 405720 | + | GACGAG | A | 9 | 1.177 | 139 | NA | NA | NA |
| 407715 | - | GACGAG | A | 6 | 1.111 | 122 | NA | NA | NA |
| 410006 | + | GACGAG | A | 5 | 1.091 | 117 | NA | NA | NA |
| 412914 | - | GACGAG | A | 4 | 1.023 | 118 | NA | NA | NA |
| 425191 | + | GACGAG | A | 5 | 1.082 | 148 | NA | NA | NA |
| 426995 | + | GACGAG | A | 7 | 1.151 | 133 | NA | NA | NA |
| 428338 | - | GACGAG | A | 2 | 0.948 | 146 | NA | NA | NA |
| 432926 | - | GACGAG | A | 4 | 1.019 | 154 | NA | NA | NA |
| 434531 | - | GACGAG | A | 5 | 1.109 | 137 | NA | NA | NA |
| 435611 | - | GACGAG | A | 3 | 1.005 | 124 | NA | NA | NA |
| 436617 | + | GACGAG | A | 5 | 1.084 | 142 | NA | NA | NA |
| 439786 | - | GACGAG | A | 11 | 1.209 | 138 | NA | NA | NA |
| 440961 | - | GACGAG | A | 10 | 1.201 | 154 | NA | NA | NA |
| 442809 | - | GACGAG | A | 14 | 1.315 | 119 | NA | NA | NA |
| 446359 | - | GACGAG | A | 8 | 1.143 | 144 | NA | NA | NA |
| 447887 | - | GACGAG | A | 5 | 1.075 | 130 | NA | NA | NA |
| 447901 | + | GACGAG | A | 6 | 1.098 | 134 | NA | NA | NA |
| 451127 | + | GACGAG | A | 6 | 1.111 | 138 | NA | NA | NA |
| 452376 | - | GACGAG | A | 6 | 1.102 | 102 | NA | NA | NA |
| 454544 | - | GACGAG | A | 0 | 0.806 | 104 | NA | NA | NA |
| 456133 | + | GACGAG | A | 5 | 1.069 | 117 | NA | NA | NA |
| 458089 | - | GACGAG | A | 0 | 0.752 | 101 | NA | NA | NA |
| 458818 | - | GACGAG | A | 20 | 1.499 | 110 | NA | NA | NA |
| 459795 | + | GACGAG | A | 3 | 0.974 | 123 | NA | NA | NA |
| 459819 | - | GACGAG | A | 5 | 1.09 | 119 | NA | NA | NA |
| 460837 | + | GACGAG | A | 9 | 1.17 | 137 | NA | NA | NA |
| 463340 | - | GACGAG | A | 12 | 1.293 | 129 | NA | NA | NA |
| 464308 | + | GACGAG | A | 1 | 0.918 | 131 | NA | NA | NA |
| 466770 | + | GACGAG | A | 2 | 0.929 | 112 | NA | NA | NA |
| 467801 | - | GACGAG | A | 8 | 1.159 | 121 | NA | NA | NA |
| 469025 | - | GACGAG | A | 2 | 0.962 | 116 | NA | NA | NA |
| 472641 | + | GACGAG | A | 5 | 1.055 | 173 | NA | NA | NA |
| 473039 | + | GACGAG | A | 3 | 1 | 175 | NA | NA | NA |
| 473671 | - | GACGAG | A | 3 | 0.981 | 188 | NA | NA | NA |
| 479026 | + | GACGAG | A | 2 | 0.97 | 159 | NA | NA | NA |
| 481366 | - | GACGAG | A | 16 | 1.321 | 193 | NA | NA | NA |
| 481657 | + | GACGAG | A | 34 | 1.549 | 186 | NA | NA | NA |
| 482008 | - | GACGAG | A | 10 | 1.202 | 189 | NA | NA | NA |
| 483068 | - | GACGAG | A | 4 | 1.027 | 165 | NA | NA | NA |
| 485753 | - | GACGAG | A | 11 | 1.236 | 169 | NA | NA | NA |
| 491444 | - | GACGAG | A | 4 | 1.05 | 159 | NA | NA | NA |
| 492653 | - | GACGAG | A | 8 | 1.15 | 145 | NA | NA | NA |
| 497290 | + | GACGAG | A | 2 | 0.976 | 156 | NA | NA | NA |
| 498457 | - | GACGAG | A | 3 | 0.997 | 148 | NA | NA | NA |
| 501624 | + | GACGAG | A | 2 | 0.897 | 136 | NA | NA | NA |
| 502349 | - | GACGAG | A | 5 | 1.074 | 120 | NA | NA | NA |
| 505583 | + | GACGAG | A | 16 | 1.457 | 127 | NA | NA | NA |
| 509809 | + | GACGAG | A | 8 | 1.186 | 107 | NA | NA | NA |
| 513597 | - | GACGAG | A | 1 | 0.829 | 95 | NA | NA | NA |
| 517248 | + | GACGAG | A | 3 | 1.016 | 74 | NA | NA | NA |
| 520546 | - | GACGAG | A | 7 | 1.19 | 57 | NA | NA | NA |
| 530274 | - | GACGAG | A | 1 | 0.905 | 129 | NA | NA | NA |
| 548788 | + | GACGAG | A | 2 | 0.968 | 116 | NA | NA | NA |
| 550002 | - | GACGAG | A | 0 | 0.693 | 118 | NA | NA | NA |
| 567992 | + | GACGAG | A | 1 | 0.895 | 98 | NA | NA | NA |
| 572837 | + | GACGAG | A | 4 | 1.037 | 133 | NA | NA | NA |
| 580078 | - | GACGAG | A | 19 | 1.392 | 150 | NA | NA | NA |
| 580179 | + | GACGAG | A | 6 | 1.099 | 145 | NA | NA | NA |
| 588709 | + | GACGAG | A | 3 | 1.024 | 131 | NA | NA | NA |
| 590001 | + | GACGAG | A | 5 | 1.065 | 148 | NA | NA | NA |
| 590094 | + | GACGAG | A | 2 | 0.976 | 149 | NA | NA | NA |
| 591381 | - | GACGAG | A | 12 | 1.298 | 118 | NA | NA | NA |
| 594980 | - | GACGAG | A | 6 | 1.105 | 100 | NA | NA | NA |
| 601553 | - | GACGAG | A | 9 | 1.185 | 122 | NA | NA | NA |
| 603326 | - | GACGAG | A | 4 | 1.027 | 124 | NA | NA | NA |
| 608247 | - | GACGAG | A | 4 | 1.05 | 123 | NA | NA | NA |
| 608688 | - | GACGAG | A | 14 | 1.275 | 145 | NA | NA | NA |
| 625190 | + | GACGAG | A | 1 | 0.896 | 86 | NA | NA | NA |
| 626626 | - | GACGAG | A | 0 | 0.753 | 82 | NA | NA | NA |
| 633716 | + | GACGAG | A | 6 | 1.09 | 148 | NA | NA | NA |
| 636022 | - | GACGAG | A | 8 | 1.185 | 118 | NA | NA | NA |
| 647337 | + | GACGAG | A | 5 | 1.079 | 129 | NA | NA | NA |
| 648316 | - | GACGAG | A | 4 | 1.049 | 173 | NA | NA | NA |
| 648490 | + | GACGAG | A | 4 | 1.038 | 165 | NA | NA | NA |
| 649382 | - | GACGAG | A | 3 | 0.999 | 170 | NA | NA | NA |
| 650977 | - | GACGAG | A | 5 | 1.067 | 175 | NA | NA | NA |
| 655988 | + | GACGAG | A | 2 | 0.925 | 126 | NA | NA | NA |
| 658201 | - | GACGAG | A | 6 | 1.096 | 108 | NA | NA | NA |
| 662988 | + | GACGAG | A | 2 | 0.943 | 143 | NA | NA | NA |
| 666058 | + | GACGAG | A | 11 | 1.211 | 160 | NA | NA | NA |
| 666824 | - | GACGAG | A | 2 | 0.924 | 174 | NA | NA | NA |
| 666976 | - | GACGAG | A | 9 | 1.177 | 167 | NA | NA | NA |
| 668121 | - | GACGAG | A | 4 | 1.041 | 173 | NA | NA | NA |
| 673252 | - | GACGAG | A | 5 | 1.06 | 133 | NA | NA | NA |
| 676798 | - | GACGAG | A | 12 | 1.234 | 133 | NA | NA | NA |
| 680067 | + | GACGAG | A | 21 | 1.462 | 156 | NA | NA | NA |
| 683944 | + | GACGAG | A | 0 | 0.747 | 136 | NA | NA | NA |
| 685332 | + | GACGAG | A | 2 | 0.972 | 143 | NA | NA | NA |
| 685878 | - | GACGAG | A | 1 | 0.859 | 142 | NA | NA | NA |
| 686726 | - | GACGAG | A | 9 | 1.158 | 139 | NA | NA | NA |
| 698351 | + | GACGAG | A | 13 | 1.323 | 159 | NA | NA | NA |
| 701079 | - | GACGAG | A | 16 | 1.339 | 106 | NA | NA | NA |
| 709399 | - | GACGAG | A | 2 | 0.921 | 89 | NA | NA | NA |
| 712161 | - | GACGAG | A | 1 | 0.875 | 107 | NA | NA | NA |
| 720239 | - | GACGAG | A | 16 | 1.337 | 152 | NA | NA | NA |
| 721449 | - | GACGAG | A | 1 | 0.864 | 144 | NA | NA | NA |
| 721949 | + | GACGAG | A | 22 | 1.43 | 136 | NA | NA | NA |
| 723445 | + | GACGAG | A | 10 | 1.205 | 129 | NA | NA | NA |
| 724288 | + | GACGAG | A | 1 | 0.869 | 128 | NA | NA | NA |
| 725075 | + | GACGAG | A | 6 | 1.093 | 130 | NA | NA | NA |
| 736473 | - | GACGAG | A | 6 | 1.1 | 158 | NA | NA | NA |
| 739577 | - | GACGAG | A | 7 | 1.129 | 170 | NA | NA | NA |
| 747025 | - | GACGAG | A | 2 | 0.972 | 117 | NA | NA | NA |
| 750347 | - | GACGAG | A | 5 | 1.065 | 105 | NA | NA | NA |
| 752753 | - | GACGAG | A | 3 | 0.997 | 118 | NA | NA | NA |
| 755190 | + | GACGAG | A | 2 | 0.977 | 125 | NA | NA | NA |
| 757348 | - | GACGAG | A | 9 | 1.377 | 148 | NA | NA | NA |
| 757592 | + | GACGAG | A | 2 | 0.95 | 137 | NA | NA | NA |
| 764540 | - | GACGAG | A | 10 | 1.195 | 111 | NA | NA | NA |
| 771833 | + | GACGAG | A | 9 | 1.154 | 139 | NA | NA | NA |
| 771897 | + | GACGAG | A | 15 | 1.342 | 136 | NA | NA | NA |
| 773141 | + | GACGAG | A | 7 | 1.138 | 141 | NA | NA | NA |
| 773412 | + | GACGAG | A | 16 | 1.311 | 141 | NA | NA | NA |
| 773588 | - | GACGAG | A | 4 | 1.04 | 137 | NA | NA | NA |
| 775083 | - | GACGAG | A | 1 | 0.857 | 138 | NA | NA | NA |
| 778631 | - | GACGAG | A | 9 | 1.208 | 122 | NA | NA | NA |
| 779619 | - | GACGAG | A | 4 | 1.058 | 114 | NA | NA | NA |
| 785837 | + | GACGAG | A | 15 | 1.349 | 100 | NA | NA | NA |
| 787959 | + | GACGAG | A | 1 | 0.883 | 103 | NA | NA | NA |
| 788415 | + | GACGAG | A | 6 | 1.113 | 103 | NA | NA | NA |
| 790709 | + | GACGAG | A | 6 | 1.114 | 145 | NA | NA | NA |
| 795757 | + | GACGAG | A | 3 | 1.01 | 119 | NA | NA | NA |
| 797739 | - | GACGAG | A | 6 | 1.121 | 96 | NA | NA | NA |
| 798603 | - | GACGAG | A | 2 | 0.928 | 98 | NA | NA | NA |
| 799130 | - | GACGAG | A | 8 | 1.179 | 103 | NA | NA | NA |
| 799252 | + | GACGAG | A | 5 | 1.099 | 112 | NA | NA | NA |
| 799929 | + | GACGAG | A | 1 | 0.868 | 105 | NA | NA | NA |
| 800225 | + | GACGAG | A | 2 | 0.961 | 102 | NA | NA | NA |
| 801271 | - | GACGAG | A | 5 | 1.09 | 110 | NA | NA | NA |
| 805241 | + | GACGAG | A | 2 | 0.939 | 132 | NA | NA | NA |
| 807462 | + | GACGAG | A | 0 | 0.66 | 146 | NA | NA | NA |
| 810792 | - | GACGAG | A | 3 | 1.019 | 136 | NA | NA | NA |
| 817152 | + | GACGAG | A | 1 | 0.904 | 128 | NA | NA | NA |
| 818063 | - | GACGAG | A | 12 | 1.32 | 135 | NA | NA | NA |
| 820817 | - | GACGAG | A | 8 | 1.137 | 136 | NA | NA | NA |
| 829107 | - | GACGAG | A | 9 | 1.178 | 138 | NA | NA | NA |
| 829167 | - | GACGAG | A | 12 | 1.252 | 138 | NA | NA | NA |
| 830062 | - | GACGAG | A | 7 | 1.134 | 131 | NA | NA | NA |
| 835649 | - | GACGAG | A | 13 | 1.251 | 137 | NA | NA | NA |
| 837072 | + | GACGAG | A | 7 | 1.14 | 124 | NA | NA | NA |
| 840052 | + | GACGAG | A | 5 | 1.244 | 115 | NA | NA | NA |
| 840886 | + | GACGAG | A | 8 | 1.159 | 110 | NA | NA | NA |
| 850960 | - | GACGAG | A | 13 | 1.249 | 121 | NA | NA | NA |
| 852207 | + | GACGAG | A | 5 | 1.077 | 143 | NA | NA | NA |
| 853004 | + | GACGAG | A | 5 | 1.055 | 160 | NA | NA | NA |
| 856162 | - | GACGAG | A | 16 | 1.304 | 162 | NA | NA | NA |
| 857884 | + | GACGAG | A | 7 | 1.144 | 142 | NA | NA | NA |
| 860409 | + | GACGAG | A | 22 | 1.431 | 138 | NA | NA | NA |
| 867479 | - | GACGAG | A | 13 | 1.235 | 145 | NA | NA | NA |
| 874262 | - | GACGAG | A | 6 | 1.089 | 180 | NA | NA | NA |
| 875855 | + | GACGAG | A | 1 | 0.894 | 159 | NA | NA | NA |
| 884771 | - | GACGAG | A | 8 | 1.133 | 161 | NA | NA | NA |
| 885755 | + | GACGAG | A | 17 | 1.318 | 168 | NA | NA | NA |
| 886931 | - | GACGAG | A | 46 | 1.744 | 177 | NA | NA | NA |
| 887150 | + | GACGAG | A | 0 | 0.78 | 172 | NA | NA | NA |
| 889671 | + | GACGAG | A | 5 | 1.061 | 155 | NA | NA | NA |
| 890110 | - | GACGAG | A | 5 | 1.059 | 140 | NA | NA | NA |
| 897440 | - | GACGAG | A | 0 | 0.795 | 124 | NA | NA | NA |
| 899039 | + | GACGAG | A | 1 | 0.927 | 146 | NA | NA | NA |
| 903344 | + | GACGAG | A | 7 | 1.122 | 125 | NA | NA | NA |
| 903500 | - | GACGAG | A | 1 | 0.913 | 131 | NA | NA | NA |
| 903533 | - | GACGAG | A | 5 | 1.076 | 128 | NA | NA | NA |
| 907208 | + | GACGAG | A | 16 | 1.41 | 116 | NA | NA | NA |
| 912816 | - | GACGAG | A | 2 | 0.962 | 82 | NA | NA | NA |
| 915012 | - | GACGAG | A | 5 | 1.085 | 89 | NA | NA | NA |
| 919419 | + | GACGAG | A | 4 | 1.098 | 74 | NA | NA | NA |
| 928123 | + | GACGAG | A | 4 | 1.028 | 111 | NA | NA | NA |
| 928749 | + | GACGAG | A | 7 | 1.142 | 115 | NA | NA | NA |
| 933467 | + | GACGAG | A | 4 | 1.041 | 112 | NA | NA | NA |
| 938038 | + | GACGAG | A | 3 | 1.01 | 111 | NA | NA | NA |
| 941056 | - | GACGAG | A | 5 | 1.07 | 108 | NA | NA | NA |
| 944518 | + | GACGAG | A | 2 | 0.959 | 136 | NA | NA | NA |
| 947125 | - | GACGAG | A | 3 | 1.018 | 144 | NA | NA | NA |
| 948916 | - | GACGAG | A | 5 | 1.075 | 120 | NA | NA | NA |
| 950270 | - | GACGAG | A | 2 | 0.947 | 105 | NA | NA | NA |
| 953182 | + | GACGAG | A | 2 | 0.879 | 105 | NA | NA | NA |
| 956011 | - | GACGAG | A | 3 | 1.014 | 111 | NA | NA | NA |
| 958725 | - | GACGAG | A | 6 | 1.097 | 118 | NA | NA | NA |
| 964536 | - | GACGAG | A | 16 | 1.374 | 113 | NA | NA | NA |
| 969090 | + | GACGAG | A | 5 | 1.087 | 119 | NA | NA | NA |
| 973081 | + | GACGAG | A | 6 | 1.1 | 137 | NA | NA | NA |
| 974552 | + | GACGAG | A | 6 | 1.119 | 150 | NA | NA | NA |
| 976494 | + | GACGAG | A | 6 | 1.201 | 151 | NA | NA | NA |
| 978454 | + | GACGAG | A | 2 | 0.973 | 155 | NA | NA | NA |
| 980245 | - | GACGAG | A | 4 | 1.043 | 131 | NA | NA | NA |
| 985368 | + | GACGAG | A | 1 | 0.911 | 102 | NA | NA | NA |
| 985500 | + | GACGAG | A | 2 | 0.966 | 99 | NA | NA | NA |
| 985584 | + | GACGAG | A | 8 | 1.148 | 97 | NA | NA | NA |
| 986438 | + | GACGAG | A | 1 | 0.889 | 91 | NA | NA | NA |
| 988937 | + | GACGAG | A | 1 | 0.852 | 97 | NA | NA | NA |
| 1011487 | - | GACGAG | A | 7 | 1.146 | 118 | NA | NA | NA |
| 1013532 | - | GACGAG | A | 2 | 0.956 | 109 | NA | NA | NA |
| 1015410 | - | GACGAG | A | 9 | 1.173 | 110 | NA | NA | NA |
| 1016789 | - | GACGAG | A | 5 | 1.08 | 118 | NA | NA | NA |
| 1018700 | - | GACGAG | A | 18 | 1.31 | 133 | NA | NA | NA |
| 1020352 | - | GACGAG | A | 1 | 0.911 | 134 | NA | NA | NA |
| 1020514 | + | GACGAG | A | 4 | 1.021 | 124 | NA | NA | NA |
| 1021836 | + | GACGAG | A | 4 | 1.04 | 138 | NA | NA | NA |
| 1023064 | + | GACGAG | A | 5 | 1.085 | 126 | NA | NA | NA |
| 1025899 | - | GACGAG | A | 0 | 0.817 | 122 | NA | NA | NA |
| 1030300 | + | GACGAG | A | 5 | 1.061 | 95 | NA | NA | NA |
| 1033087 | - | GACGAG | A | 3 | 1.002 | 101 | NA | NA | NA |
| 1035284 | + | GACGAG | A | 23 | 1.539 | 105 | NA | NA | NA |
| 1035422 | + | GACGAG | A | 2 | 0.958 | 103 | NA | NA | NA |
| 1038041 | + | GACGAG | A | 9 | 1.19 | 120 | NA | NA | NA |
| 1041301 | - | GACGAG | A | 1 | 0.792 | 117 | NA | NA | NA |
| 1041793 | - | GACGAG | A | 5 | 1.068 | 118 | NA | NA | NA |
| 1048424 | + | GACGAG | A | 1 | 0.89 | 143 | NA | NA | NA |
| 1052949 | + | GACGAG | A | 19 | 1.44 | 89 | NA | NA | NA |
| 1054134 | + | GACGAG | A | 1 | 0.847 | 54 | NA | NA | NA |
| 1055680 | - | GACGAG | A | 4 | 1.064 | 57 | NA | NA | NA |
| 1056375 | + | GACGAG | A | 1 | 0.866 | 45 | NA | NA | NA |
| 1057905 | - | GACGAG | A | 2 | 0.905 | 48 | NA | NA | NA |
| 1059874 | + | GACGAG | A | 15 | 1.452 | 45 | NA | NA | NA |
| 1060210 | + | GACGAG | A | 2 | 0.911 | 49 | NA | NA | NA |
| 1063299 | - | GACGAG | A | 8 | 1.226 | 63 | NA | NA | NA |
| 1064384 | - | GACGAG | A | 6 | 1.108 | 73 | NA | NA | NA |
| 1070827 | - | GACGAG | A | 24 | 1.546 | 120 | NA | NA | NA |
| 1071704 | + | GACGAG | A | 15 | 1.349 | 117 | NA | NA | NA |
| 1071867 | + | GACGAG | A | 2 | 0.96 | 110 | NA | NA | NA |
| 1072192 | - | GACGAG | A | 1 | 0.876 | 104 | NA | NA | NA |
| 1073380 | - | GACGAG | A | 1 | 0.8 | 98 | NA | NA | NA |
| 1073715 | - | GACGAG | A | 12 | 1.327 | 96 | NA | NA | NA |
| 1075292 | - | GACGAG | A | 4 | 1.028 | 111 | NA | NA | NA |
| 1075329 | + | GACGAG | A | 8 | 1.238 | 97 | NA | NA | NA |
| 1084804 | - | GACGAG | A | 4 | 1.033 | 120 | NA | NA | NA |
| 1089976 | - | GACGAG | A | 2 | 0.948 | 80 | NA | NA | NA |
| 1091115 | - | GACGAG | A | 29 | 1.75 | 66 | NA | NA | NA |
| 1094236 | + | GACGAG | A | 6 | 1.102 | 98 | NA | NA | NA |
| 1098995 | - | GACGAG | A | 7 | 1.13 | 134 | NA | NA | NA |
| 1099618 | + | GACGAG | A | 11 | 1.224 | 123 | NA | NA | NA |
| 1100316 | + | GACGAG | A | 6 | 1.115 | 130 | NA | NA | NA |
| 1101285 | + | GACGAG | A | 9 | 1.181 | 124 | NA | NA | NA |
| 1102169 | - | GACGAG | A | 4 | 1.039 | 122 | NA | NA | NA |
| 1102430 | + | GACGAG | A | 13 | 1.313 | 128 | NA | NA | NA |
| 1103283 | + | GACGAG | A | 3 | 1.015 | 122 | NA | NA | NA |
| 1104105 | - | GACGAG | A | 5 | 1.086 | 101 | NA | NA | NA |
| 1104360 | + | GACGAG | A | 6 | 1.083 | 117 | NA | NA | NA |
| 1104405 | + | GACGAG | A | 2 | 0.956 | 116 | NA | NA | NA |
| 1108132 | + | GACGAG | A | 0 | 0.705 | 98 | NA | NA | NA |
| 1116765 | - | GACGAG | A | 3 | 0.979 | 131 | NA | NA | NA |
| 1117946 | + | GACGAG | A | 9 | 1.181 | 122 | NA | NA | NA |
| 1120311 | - | GACGAG | A | 19 | 1.379 | 114 | NA | NA | NA |
| 1122108 | + | GACGAG | A | 2 | 0.954 | 120 | NA | NA | NA |
| 1122858 | - | GACGAG | A | 0 | 0.814 | 124 | NA | NA | NA |
| 1126172 | + | GACGAG | A | 2 | 0.966 | 133 | NA | NA | NA |
| 1127471 | - | GACGAG | A | 5 | 1.084 | 140 | NA | NA | NA |
| 1133877 | + | GACGAG | A | 17 | 1.323 | 138 | NA | NA | NA |
| 1136078 | - | GACGAG | A | 2 | 0.925 | 108 | NA | NA | NA |
| 1137145 | - | GACGAG | A | 2 | 0.921 | 124 | NA | NA | NA |
| 1138010 | + | GACGAG | A | 12 | 1.265 | 119 | NA | NA | NA |
| 1139958 | - | GACGAG | A | 5 | 1.065 | 117 | NA | NA | NA |
| 1142917 | + | GACGAG | A | 6 | 1.1 | 163 | NA | NA | NA |
| 1153451 | + | GACGAG | A | 0 | 0.813 | 115 | NA | NA | NA |
| 1156733 | + | GACGAG | A | 5 | 1.066 | 106 | NA | NA | NA |
| 1163551 | - | GACGAG | A | 5 | 1.082 | 128 | NA | NA | NA |
| 1164412 | + | GACGAG | A | 6 | 1.098 | 135 | NA | NA | NA |
| 1165453 | + | GACGAG | A | 1 | 0.863 | 108 | NA | NA | NA |
| 1167960 | - | GACGAG | A | 10 | 1.258 | 102 | NA | NA | NA |
| 1169424 | + | GACGAG | A | 8 | 1.199 | 84 | NA | NA | NA |
| 1170534 | - | GACGAG | A | 6 | 1.109 | 79 | NA | NA | NA |
| 1171347 | + | GACGAG | A | 2 | 0.914 | 78 | NA | NA | NA |
| 1171864 | + | GACGAG | A | 20 | 1.487 | 78 | NA | NA | NA |
| 1174680 | - | GACGAG | A | 7 | 1.161 | 95 | NA | NA | NA |
| 1178877 | + | GACGAG | A | 3 | 1.002 | 95 | NA | NA | NA |
| 1188664 | - | GACGAG | A | 4 | 1.046 | 104 | NA | NA | NA |
| 1191797 | + | GACGAG | A | 1 | 0.856 | 102 | NA | NA | NA |
| 1194288 | + | GACGAG | A | 5 | 1.07 | 106 | NA | NA | NA |
| 1196346 | - | GACGAG | A | 7 | 1.163 | 112 | NA | NA | NA |
| 1196874 | - | GACGAG | A | 2 | 0.926 | 104 | NA | NA | NA |
| 1210157 | + | GACGAG | A | 8 | 1.173 | 116 | NA | NA | NA |
| 1210361 | - | GACGAG | A | 6 | 1.092 | 125 | NA | NA | NA |
| 1214636 | - | GACGAG | A | 2 | 0.938 | 158 | NA | NA | NA |
| 1214735 | - | GACGAG | A | 6 | 1.146 | 154 | NA | NA | NA |
| 1214855 | - | GACGAG | A | 8 | 1.14 | 155 | NA | NA | NA |
| 1214870 | + | GACGAG | A | 5 | 1.066 | 155 | NA | NA | NA |
| 1216910 | - | GACGAG | A | 2 | 0.974 | 126 | NA | NA | NA |
| 1251236 | - | GACGAG | A | 5 | 1.07 | 141 | NA | NA | NA |
| 1265523 | - | GACGAG | A | 5 | 1.123 | 78 | NA | NA | NA |
| 1265769 | - | GACGAG | A | 3 | 0.98 | 75 | NA | NA | NA |
| 1265874 | - | GACGAG | A | 8 | 1.221 | 78 | NA | NA | NA |
| 1268549 | + | GACGAG | A | 1 | 0.662 | 97 | NA | NA | NA |
| 1271216 | + | GACGAG | A | 3 | 0.995 | 112 | NA | NA | NA |
| 1279300 | - | GACGAG | A | 6 | 1.133 | 108 | NA | NA | NA |
| 1285821 | + | GACGAG | A | 2 | 0.939 | 123 | NA | NA | NA |
| 1285920 | - | GACGAG | A | 10 | 1.243 | 118 | NA | NA | NA |
| 1287328 | - | GACGAG | A | 6 | 1.103 | 117 | NA | NA | NA |
| 1292139 | + | GACGAG | A | 3 | 1.001 | 127 | NA | NA | NA |
| 1292412 | + | GACGAG | A | 9 | 1.23 | 128 | NA | NA | NA |
| 1292830 | + | GACGAG | A | 2 | 0.939 | 120 | NA | NA | NA |
| 1294146 | - | GACGAG | A | 4 | 1.023 | 131 | NA | NA | NA |
| 1302142 | + | GACGAG | A | 8 | 1.161 | 129 | NA | NA | NA |
| 1302895 | + | GACGAG | A | 14 | 1.289 | 131 | NA | NA | NA |
| 1306173 | + | GACGAG | A | 9 | 1.179 | 127 | NA | NA | NA |
| 1311501 | - | GACGAG | A | 9 | 1.233 | 91 | NA | NA | NA |
| 1313817 | - | GACGAG | A | 4 | 1.042 | 90 | NA | NA | NA |
| 1316208 | - | GACGAG | A | 12 | 1.254 | 104 | NA | NA | NA |
| 1320890 | - | GACGAG | A | 1 | 0.9 | 81 | NA | NA | NA |
| 1322596 | + | GACGAG | A | 7 | 1.118 | 106 | NA | NA | NA |
| 1324780 | + | GACGAG | A | 6 | 1.115 | 108 | NA | NA | NA |
| 1324988 | - | GACGAG | A | 4 | 1.043 | 115 | NA | NA | NA |
| 1329577 | + | GACGAG | A | 3 | 1.021 | 94 | NA | NA | NA |
| 1331034 | - | GACGAG | A | 5 | 1.065 | 100 | NA | NA | NA |
| 1332266 | - | GACGAG | A | 7 | 1.13 | 103 | NA | NA | NA |
| 1332278 | + | GACGAG | A | 1 | 0.882 | 89 | NA | NA | NA |
| 1332783 | - | GACGAG | A | 17 | 1.405 | 107 | NA | NA | NA |
| 1343111 | - | GACGAG | A | 12 | 1.241 | 115 | NA | NA | NA |
| 1345098 | + | GACGAG | A | 1 | 0.916 | 115 | NA | NA | NA |
| 1348266 | - | GACGAG | A | 1 | 0.926 | 126 | NA | NA | NA |
| 1351162 | + | GACGAG | A | 1 | 0.873 | 112 | NA | NA | NA |
| 1352324 | - | GACGAG | A | 3 | 1.021 | 100 | NA | NA | NA |
| 1352882 | + | GACGAG | A | 7 | 1.162 | 108 | NA | NA | NA |
| 1353428 | - | GACGAG | A | 7 | 1.128 | 106 | NA | NA | NA |
| 1355480 | - | GACGAG | A | 5 | 1.073 | 98 | NA | NA | NA |
| 1358526 | + | GACGAG | A | 4 | 1.039 | 98 | NA | NA | NA |
| 1362203 | + | GACGAG | A | 9 | 1.178 | 102 | NA | NA | NA |
| 1365485 | + | GACGAG | A | 10 | 1.201 | 113 | NA | NA | NA |
| 1373912 | - | GACGAG | A | 9 | 1.223 | 76 | NA | NA | NA |
| 1374338 | - | GACGAG | A | 6 | 1.11 | 84 | NA | NA | NA |
| 1377776 | + | GACGAG | A | 2 | 0.918 | 106 | NA | NA | NA |
| 1384250 | + | GACGAG | A | 2 | 0.958 | 135 | NA | NA | NA |
| 1384535 | - | GACGAG | A | 0 | 0.752 | 114 | NA | NA | NA |
| 1389941 | + | GACGAG | A | 9 | 1.185 | 125 | NA | NA | NA |
| 1390022 | + | GACGAG | A | 4 | 1.025 | 131 | NA | NA | NA |
| 1392105 | - | GACGAG | A | 7 | 1.117 | 113 | NA | NA | NA |
| 1396500 | + | GACGAG | A | 1 | 0.818 | 80 | NA | NA | NA |
| 1397465 | - | GACGAG | A | 5 | 1.088 | 82 | NA | NA | NA |
| 1400182 | + | GACGAG | A | 4 | 1.029 | 101 | NA | NA | NA |
| 1401266 | - | GACGAG | A | 3 | 1.011 | 116 | NA | NA | NA |
| 1402356 | + | GACGAG | A | 26 | 1.561 | 105 | NA | NA | NA |
| 1404253 | - | GACGAG | A | 2 | 0.953 | 124 | NA | NA | NA |
| 1410078 | + | GACGAG | A | 3 | 1.003 | 75 | NA | NA | NA |
| 1410142 | - | GACGAG | A | 1 | 0.847 | 76 | NA | NA | NA |
| 1413539 | + | GACGAG | A | 5 | 1.059 | 92 | NA | NA | NA |
| 1414962 | + | GACGAG | A | 0 | 0.784 | 90 | NA | NA | NA |
| 1419320 | + | GACGAG | A | 0 | 0.819 | 90 | NA | NA | NA |
| 1421613 | - | GACGAG | A | 3 | 1.018 | 93 | NA | NA | NA |
| 1422469 | - | GACGAG | A | 0 | 0.677 | 91 | NA | NA | NA |
| 1424213 | + | GACGAG | A | 10 | 1.225 | 108 | NA | NA | NA |
| 1440372 | + | GACGAG | A | 6 | 1.12 | 106 | NA | NA | NA |
| 1442182 | + | GACGAG | A | 1 | 0.759 | 105 | NA | NA | NA |
| 1448855 | + | GACGAG | A | 2 | 0.963 | 67 | NA | NA | NA |
| 1449333 | + | GACGAG | A | 3 | 1.002 | 70 | NA | NA | NA |
| 1452427 | + | GACGAG | A | 0 | 0.769 | 67 | NA | NA | NA |
| 1455492 | + | GACGAG | A | 13 | 1.323 | 105 | NA | NA | NA |
| 1455979 | + | GACGAG | A | 9 | 1.272 | 113 | NA | NA | NA |
| 1460264 | + | GACGAG | A | 17 | 1.448 | 121 | NA | NA | NA |
| 1469348 | + | GACGAG | A | 0 | 0.772 | 91 | NA | NA | NA |
| 1470085 | + | GACGAG | A | 6 | 1.176 | 96 | NA | NA | NA |
| 1476978 | + | GACGAG | A | 3 | 1.012 | 89 | NA | NA | NA |
| 1483370 | - | GACGAG | A | 1 | 0.835 | 88 | NA | NA | NA |
| 1489151 | + | GACGAG | A | 0 | 0.745 | 53 | NA | NA | NA |
| 1493660 | + | GACGAG | A | 0 | 0.604 | 85 | NA | NA | NA |
| 1493846 | + | GACGAG | A | 9 | 1.228 | 83 | NA | NA | NA |
| 1495354 | - | GACGAG | A | 6 | 1.105 | 66 | NA | NA | NA |
| 1497907 | - | GACGAG | A | 11 | 1.261 | 83 | NA | NA | NA |
| 1498296 | + | GACGAG | A | 2 | 0.964 | 88 | NA | NA | NA |
| 1509569 | - | GACGAG | A | 5 | 1.09 | 122 | NA | NA | NA |
| 1513640 | + | GACGAG | A | 4 | 1.042 | 101 | NA | NA | NA |
| 1531910 | + | GACGAG | A | 3 | 1 | 94 | NA | NA | NA |
| 1531922 | - | GACGAG | A | 1 | 0.854 | 93 | NA | NA | NA |
| 1535834 | - | GACGAG | A | 2 | 0.945 | 104 | NA | NA | NA |
| 1540534 | + | GACGAG | A | 11 | 1.273 | 113 | NA | NA | NA |
| 1542350 | + | GACGAG | A | 8 | 1.164 | 96 | NA | NA | NA |
| 1544099 | - | GACGAG | A | 4 | 1.037 | 123 | NA | NA | NA |
| 1550257 | - | GACGAG | A | 1 | 0.892 | 118 | NA | NA | NA |
| 1550572 | + | GACGAG | A | 23 | 1.479 | 115 | NA | NA | NA |
| 1556146 | - | GACGAG | A | 11 | 1.237 | 101 | NA | NA | NA |
| 1560107 | + | GACGAG | A | 7 | 1.149 | 79 | NA | NA | NA |
| 1569839 | - | GACGAG | A | 2 | 0.931 | 99 | NA | NA | NA |
| 1574726 | + | GACGAG | A | 1 | 0.916 | 122 | NA | NA | NA |
| 1576332 | - | GACGAG | A | 3 | 0.999 | 125 | NA | NA | NA |
| 1581862 | - | GACGAG | A | 1 | 0.919 | 101 | NA | NA | NA |
| 1582179 | - | GACGAG | A | 15 | 1.341 | 97 | NA | NA | NA |
| 1585987 | + | GACGAG | A | 3 | 1.004 | 111 | NA | NA | NA |
| 1588707 | + | GACGAG | A | 2 | 0.939 | 140 | NA | NA | NA |
| 1591910 | - | GACGAG | A | 7 | 1.125 | 120 | NA | NA | NA |
| 1600939 | + | GACGAG | A | 3 | 0.99 | 105 | NA | NA | NA |
| 1600997 | + | GACGAG | A | 5 | 1.095 | 98 | NA | NA | NA |
| 1603608 | - | GACGAG | A | 3 | 1.017 | 92 | NA | NA | NA |
| 1604362 | + | GACGAG | A | 3 | 0.997 | 107 | NA | NA | NA |
| 1608181 | + | GACGAG | A | 3 | 1.017 | 119 | NA | NA | NA |
| 1610043 | + | GACGAG | A | 3 | 0.978 | 109 | NA | NA | NA |
| 1611701 | - | GACGAG | A | 7 | 1.117 | 101 | NA | NA | NA |
| 1613405 | + | GACGAG | A | 0 | 0.817 | 103 | NA | NA | NA |
| 1613410 | + | GACGAG | A | 0 | 0.519 | 103 | NA | NA | NA |
| 1615564 | + | GACGAG | A | 1 | 0.922 | 104 | NA | NA | NA |
| 1615933 | + | GACGAG | A | 4 | 1.027 | 101 | NA | NA | NA |
| 1617595 | - | GACGAG | A | 0 | 0.761 | 110 | NA | NA | NA |
| 1621893 | + | GACGAG | A | 4 | 1.033 | 98 | NA | NA | NA |
| 1623965 | + | GACGAG | A | 1 | 0.868 | 116 | NA | NA | NA |
| 1625378 | + | GACGAG | A | 7 | 1.138 | 101 | NA | NA | NA |
| 1628523 | + | GACGAG | A | 13 | 1.334 | 110 | NA | NA | NA |
| 1637264 | + | GACGAG | A | 1 | 0.899 | 92 | NA | NA | NA |
| 1640724 | + | GACGAG | A | 9 | 1.215 | 86 | NA | NA | NA |
| 1641098 | + | GACGAG | A | 3 | 0.986 | 92 | NA | NA | NA |
| 1642447 | + | GACGAG | A | 9 | 1.206 | 94 | NA | NA | NA |
| 1648698 | - | GACGAG | A | 7 | 1.139 | 108 | NA | NA | NA |
| 1651859 | + | GACGAG | A | 4 | 1.063 | 105 | NA | NA | NA |
| 1659505 | + | GACGAG | A | 0 | 0.76 | 69 | NA | NA | NA |
| 1662858 | + | GACGAG | A | 11 | 1.258 | 106 | NA | NA | NA |
| 1667649 | - | GACGAG | A | 1 | 0.905 | 108 | NA | NA | NA |
| 1671734 | + | GACGAG | A | 10 | 1.308 | 87 | NA | NA | NA |
| 1672909 | - | GACGAG | A | 6 | 1.129 | 77 | NA | NA | NA |
| 1676870 | + | GACGAG | A | 2 | 0.973 | 111 | NA | NA | NA |
| 1694777 | + | GACGAG | A | 18 | 1.473 | 106 | NA | NA | NA |
| 1698860 | + | GACGAG | A | 3 | 0.98 | 83 | NA | NA | NA |
| 1699655 | + | GACGAG | A | 13 | 1.328 | 75 | NA | NA | NA |
| 1704435 | + | GACGAG | A | 16 | 1.361 | 112 | NA | NA | NA |
| 1704475 | + | GACGAG | A | 1 | 0.816 | 118 | NA | NA | NA |
| 1705456 | + | GACGAG | A | 15 | 1.343 | 121 | NA | NA | NA |
| 1705822 | + | GACGAG | A | 12 | 1.284 | 129 | NA | NA | NA |
| 1705931 | + | GACGAG | A | 11 | 1.243 | 130 | NA | NA | NA |
| 1706335 | + | GACGAG | A | 1 | 0.88 | 127 | NA | NA | NA |
| 1711168 | + | GACGAG | A | 6 | 1.092 | 106 | NA | NA | NA |
| 1711307 | - | GACGAG | A | 1 | 0.897 | 98 | NA | NA | NA |
| 1717056 | + | GACGAG | A | 9 | 1.201 | 100 | NA | NA | NA |
| 1720429 | - | GACGAG | A | 13 | 1.285 | 105 | NA | NA | NA |
| 1723043 | + | GACGAG | A | 5 | 1.078 | 100 | NA | NA | NA |
| 1724210 | + | GACGAG | A | 2 | 0.93 | 89 | NA | NA | NA |
| 1731575 | + | GACGAG | A | 7 | 1.159 | 83 | NA | NA | NA |
| 1731839 | + | GACGAG | A | 11 | 1.266 | 82 | NA | NA | NA |
| 1738570 | + | GACGAG | A | 0 | 0.787 | 63 | NA | NA | NA |
| 1741741 | - | GACGAG | A | 14 | 1.269 | 88 | NA | NA | NA |
| 1742161 | - | GACGAG | A | 4 | 1.046 | 93 | NA | NA | NA |
| 1748894 | + | GACGAG | A | 6 | 1.103 | 91 | NA | NA | NA |
| 1749463 | - | GACGAG | A | 14 | 1.49 | 90 | NA | NA | NA |
| 1763758 | + | GACGAG | A | 7 | 1.154 | 128 | NA | NA | NA |
| 1764388 | + | GACGAG | A | 10 | 1.241 | 135 | NA | NA | NA |
| 1768457 | + | GACGAG | A | 3 | 0.995 | 126 | NA | NA | NA |
| 1770072 | - | GACGAG | A | 1 | 0.898 | 93 | NA | NA | NA |
| 1772232 | - | GACGAG | A | 8 | 1.163 | 87 | NA | NA | NA |
| 1774939 | + | GACGAG | A | 3 | 1.015 | 82 | NA | NA | NA |
| 1775270 | + | GACGAG | A | 1 | 0.91 | 79 | NA | NA | NA |
| 1782671 | + | GACGAG | A | 4 | 1.036 | 93 | NA | NA | NA |
| 1783877 | - | GACGAG | A | 6 | 1.118 | 95 | NA | NA | NA |
| 1787370 | + | GACGAG | A | 4 | 1.051 | 119 | NA | NA | NA |
| 1796987 | + | GACGAG | A | 12 | 1.394 | 103 | NA | NA | NA |
| 1797281 | + | GACGAG | A | 4 | 1.036 | 94 | NA | NA | NA |
| 1802121 | + | GACGAG | A | 5 | 1.092 | 101 | NA | NA | NA |
| 1803395 | + | GACGAG | A | 3 | 0.984 | 113 | NA | NA | NA |
| 1803554 | + | GACGAG | A | 3 | 0.995 | 113 | NA | NA | NA |
| 1812211 | + | GACGAG | A | 8 | 1.161 | 121 | NA | NA | NA |
| 1812293 | - | GACGAG | A | 15 | 1.326 | 117 | NA | NA | NA |
| 1815258 | - | GACGAG | A | 4 | 1.029 | 120 | NA | NA | NA |
| 1819521 | - | GACGAG | A | 5 | 1.073 | 90 | NA | NA | NA |
| 1819968 | - | GACGAG | A | 1 | 0.91 | 88 | NA | NA | NA |
| 1820081 | - | GACGAG | A | 1 | 0.884 | 89 | NA | NA | NA |
| 1831471 | + | GACGAG | A | 1 | 0.823 | 66 | NA | NA | NA |
| 1835835 | - | GACGAG | A | 3 | 1.015 | 79 | NA | NA | NA |
| 1837682 | + | GACGAG | A | 1 | 0.846 | 82 | NA | NA | NA |
| 1839005 | + | GACGAG | A | 3 | 1.014 | 69 | NA | NA | NA |
| 1839947 | + | GACGAG | A | 4 | 1.042 | 84 | NA | NA | NA |
| 1840361 | - | GACGAG | A | 1 | 0.858 | 88 | NA | NA | NA |
| 1854825 | + | GACGAG | A | 1 | 0.869 | 71 | NA | NA | NA |
| 1872868 | + | GACGAG | A | 3 | 1.028 | 59 | NA | NA | NA |
| 1875702 | + | GACGAG | A | 8 | 1.201 | 68 | NA | NA | NA |
| 1878193 | + | GACGAG | A | 5 | 1.078 | 64 | NA | NA | NA |
| 1879081 | - | GACGAG | A | 10 | 1.321 | 55 | NA | NA | NA |
| 1879342 | + | GACGAG | A | 1 | 0.888 | 53 | NA | NA | NA |
| 1880948 | - | GACGAG | A | 1 | 0.92 | 58 | NA | NA | NA |
| 1881121 | - | GACGAG | A | 4 | 1.059 | 60 | NA | NA | NA |
| 1893146 | - | GACGAG | A | 3 | 1.009 | 68 | NA | NA | NA |
| 1894632 | + | GACGAG | A | 2 | 0.924 | 75 | NA | NA | NA |
| 1896672 | + | GACGAG | A | 0 | 0.639 | 89 | NA | NA | NA |
| 1897837 | + | GACGAG | A | 6 | 1.09 | 80 | NA | NA | NA |
| 1899247 | + | GACGAG | A | 4 | 1.079 | 67 | NA | NA | NA |
| 1899315 | + | GACGAG | A | 1 | 0.813 | 64 | NA | NA | NA |
| 1900040 | + | GACGAG | A | 5 | 1.079 | 75 | NA | NA | NA |
| 1907986 | - | GACGAG | A | 16 | 1.473 | 83 | NA | NA | NA |
| 1910653 | - | GACGAG | A | 7 | 1.134 | 85 | NA | NA | NA |
| 1912131 | + | GACGAG | A | 15 | 1.345 | 99 | NA | NA | NA |
| 1919958 | - | GACGAG | A | 10 | 1.212 | 109 | NA | NA | NA |
| 1928115 | + | GACGAG | A | 1 | 0.655 | 82 | NA | NA | NA |
| 1929611 | + | GACGAG | A | 8 | 1.176 | 93 | NA | NA | NA |
| 1930197 | - | GACGAG | A | 20 | 1.436 | 100 | NA | NA | NA |
| 1931415 | - | GACGAG | A | 0 | 0.48 | 108 | NA | NA | NA |
| 1934576 | + | GACGAG | A | 4 | 1.056 | 116 | NA | NA | NA |
| 1936705 | - | GACGAG | A | 3 | 1.013 | 108 | NA | NA | NA |
| 1939147 | - | GACGAG | A | 3 | 1.017 | 102 | NA | NA | NA |
| 1946842 | - | GACGAG | A | 0 | 0.567 | 99 | NA | NA | NA |
| 1950959 | + | GACGAG | A | 1 | 0.891 | 81 | NA | NA | NA |
| 1964090 | + | GACGAG | A | 2 | 0.763 | 95 | NA | NA | NA |
| 1973608 | + | GACGAG | A | 6 | 1.124 | 59 | NA | NA | NA |
| 1976036 | - | GACGAG | A | 0 | 0.77 | 75 | NA | NA | NA |
| 1983143 | - | GACGAG | A | 2 | 0.935 | 60 | NA | NA | NA |
| 2003993 | - | GACGAG | A | 8 | 1.183 | 81 | NA | NA | NA |
| 2004973 | - | GACGAG | A | 3 | 0.981 | 85 | NA | NA | NA |
| 2007195 | - | GACGAG | A | 4 | 1.037 | 88 | NA | NA | NA |
| 2010837 | + | GACGAG | A | 4 | 1.045 | 66 | NA | NA | NA |
| 2017278 | + | GACGAG | A | 2 | 0.887 | 31 | NA | NA | NA |
| 2018151 | - | GACGAG | A | 11 | 1.404 | 28 | NA | NA | NA |
| 2022532 | - | GACGAG | A | 0 | 0.673 | 39 | NA | NA | NA |
| 2033072 | - | GACGAG | A | 2 | 0.942 | 93 | NA | NA | NA |
| 2037318 | - | GACGAG | A | 5 | 1.082 | 89 | NA | NA | NA |
| 2044621 | - | GACGAG | A | 2 | 0.964 | 65 | NA | NA | NA |
| 2046488 | - | GACGAG | A | 5 | 1.088 | 88 | NA | NA | NA |
| 2047081 | - | GACGAG | A | 1 | 0.896 | 89 | NA | NA | NA |
| 2047622 | - | GACGAG | A | 4 | 1.026 | 101 | NA | NA | NA |
| 2049715 | + | GACGAG | A | 0 | 0.751 | 83 | NA | NA | NA |
| 2054253 | + | GACGAG | A | 2 | 0.974 | 59 | NA | NA | NA |
| 2057307 | - | GACGAG | A | 8 | 1.217 | 79 | NA | NA | NA |
| 2061525 | + | GACGAG | A | 31 | 1.705 | 95 | NA | NA | NA |
| 2063724 | - | GACGAG | A | 1 | 0.879 | 75 | NA | NA | NA |
| 2066245 | - | GACGAG | A | 8 | 1.186 | 73 | NA | NA | NA |
| 2067115 | - | GACGAG | A | 9 | 1.24 | 80 | NA | NA | NA |
| 2076883 | + | GACGAG | A | 0 | 0.736 | 110 | NA | NA | NA |
| 2081077 | - | GACGAG | A | 5 | 1.101 | 88 | NA | NA | NA |
| 2082777 | - | GACGAG | A | 14 | 1.431 | 95 | NA | NA | NA |
| 2085268 | - | GACGAG | A | 12 | 1.263 | 87 | NA | NA | NA |
| 2095321 | - | GACGAG | A | 12 | 1.312 | 81 | NA | NA | NA |
| 2097837 | + | GACGAG | A | 1 | 0.902 | 79 | NA | NA | NA |
| 2105418 | - | GACGAG | A | 0 | 0.605 | 70 | NA | NA | NA |
| 2119045 | - | GACGAG | A | 0 | 0.744 | 90 | NA | NA | NA |
| 2123784 | + | GACGAG | A | 5 | 1.093 | 91 | NA | NA | NA |
| 2126450 | + | GACGAG | A | 0 | 0.731 | 86 | NA | NA | NA |
| 2127349 | - | GACGAG | A | 1 | 0.851 | 78 | NA | NA | NA |
| 2132492 | + | GACGAG | A | 16 | 1.466 | 104 | NA | NA | NA |
| 2138966 | - | GACGAG | A | 10 | 1.221 | 83 | NA | NA | NA |
| 2145888 | - | GACGAG | A | 4 | 1.043 | 90 | NA | NA | NA |
| 2146248 | - | GACGAG | A | 1 | 0.864 | 90 | NA | NA | NA |
| 2147142 | - | GACGAG | A | 1 | 0.89 | 107 | NA | NA | NA |
| 2153532 | - | GACGAG | A | 7 | 1.163 | 74 | NA | NA | NA |
| 2154562 | - | GACGAG | A | 0 | 0.771 | 96 | NA | NA | NA |
| 2165134 | - | GACGAG | A | 1 | 0.869 | 119 | NA | NA | NA |
| 2169622 | - | GACGAG | A | 1 | 0.906 | 100 | NA | NA | NA |
| 2175531 | - | GACGAG | A | 10 | 1.221 | 104 | NA | NA | NA |
| 2188067 | - | GACGAG | A | 15 | 1.338 | 118 | NA | NA | NA |
| 2188643 | - | GACGAG | A | 1 | 0.908 | 127 | NA | NA | NA |
| 2189488 | + | GACGAG | A | 11 | 1.265 | 123 | NA | NA | NA |
| 2190248 | + | GACGAG | A | 0 | 0.844 | 116 | NA | NA | NA |
| 2196935 | + | GACGAG | A | 10 | 1.232 | 104 | NA | NA | NA |
| 2197293 | - | GACGAG | A | 1 | 0.846 | 104 | NA | NA | NA |
| 2197308 | - | GACGAG | A | 5 | 1.077 | 104 | NA | NA | NA |
| 2197917 | + | GACGAG | A | 1 | 0.855 | 102 | NA | NA | NA |
| 2200377 | - | GACGAG | A | 13 | 1.281 | 106 | NA | NA | NA |
| 2203931 | - | GACGAG | A | 1 | 0.881 | 77 | NA | NA | NA |
| 2213484 | - | GACGAG | A | 10 | 1.266 | 100 | NA | NA | NA |
| 2219207 | + | GACGAG | A | 20 | 1.499 | 82 | NA | NA | NA |
| 2219738 | + | GACGAG | A | 1 | 0.914 | 77 | NA | NA | NA |
| 2226560 | - | GACGAG | A | 13 | 1.348 | 99 | NA | NA | NA |
| 2231342 | - | GACGAG | A | 4 | 1.11 | 76 | NA | NA | NA |
| 2235000 | + | GACGAG | A | 1 | 0.848 | 95 | NA | NA | NA |
| 2243216 | - | GACGAG | A | 2 | 0.961 | 77 | NA | NA | NA |
| 2244531 | - | GACGAG | A | 4 | 1.029 | 88 | NA | NA | NA |
| 2245496 | - | GACGAG | A | 2 | 0.964 | 83 | NA | NA | NA |
| 2246566 | - | GACGAG | A | 4 | 1.056 | 87 | NA | NA | NA |
| 2248466 | - | GACGAG | A | 3 | 1.022 | 78 | NA | NA | NA |
| 2249138 | + | GACGAG | A | 2 | 0.966 | 92 | NA | NA | NA |
| 2256436 | + | GACGAG | A | 12 | 1.255 | 116 | NA | NA | NA |
| 2257393 | + | GACGAG | A | 4 | 1.039 | 115 | NA | NA | NA |
| 2257631 | - | GACGAG | A | 2 | 0.976 | 117 | NA | NA | NA |
| 2266968 | - | GACGAG | A | 22 | 1.607 | 106 | NA | NA | NA |
| 2271128 | + | GACGAG | A | 10 | 1.238 | 111 | NA | NA | NA |
| 2271338 | + | GACGAG | A | 9 | 1.203 | 115 | NA | NA | NA |
| 2273395 | - | GACGAG | A | 5 | 1.07 | 125 | NA | NA | NA |
| 2276510 | - | GACGAG | A | 6 | 1.107 | 102 | NA | NA | NA |
| 2280814 | - | GACGAG | A | 9 | 1.206 | 100 | NA | NA | NA |
| 2282147 | - | GACGAG | A | 4 | 1.051 | 109 | NA | NA | NA |
| 2286556 | - | GACGAG | A | 3 | 1.021 | 117 | NA | NA | NA |
| 2288075 | + | GACGAG | A | 1 | 0.917 | 105 | NA | NA | NA |
| 2290044 | + | GACGAG | A | 10 | 1.241 | 82 | NA | NA | NA |
| 2290784 | + | GACGAG | A | 3 | 0.982 | 84 | NA | NA | NA |
| 2291137 | + | GACGAG | A | 1 | 0.887 | 86 | NA | NA | NA |
| 2293037 | - | GACGAG | A | 7 | 1.135 | 74 | NA | NA | NA |
| 2293597 | - | GACGAG | A | 4 | 1.052 | 87 | NA | NA | NA |
| 2293949 | + | GACGAG | A | 11 | 1.3 | 84 | NA | NA | NA |
| 2296538 | - | GACGAG | A | 7 | 1.137 | 102 | NA | NA | NA |
| 2298261 | + | GACGAG | A | 3 | 0.993 | 110 | NA | NA | NA |
| 2308678 | - | GACGAG | A | 7 | 1.163 | 90 | NA | NA | NA |
| 2311141 | + | GACGAG | A | 17 | 1.423 | 119 | NA | NA | NA |
| 2311234 | + | GACGAG | A | 1 | 0.878 | 118 | NA | NA | NA |
| 2313371 | + | GACGAG | A | 6 | 1.117 | 119 | NA | NA | NA |
| 2314681 | - | GACGAG | A | 3 | 1.017 | 115 | NA | NA | NA |
| 2314710 | - | GACGAG | A | 0 | 0.642 | 116 | NA | NA | NA |
| 2321818 | + | GACGAG | A | 11 | 1.299 | 85 | NA | NA | NA |
| 2322949 | - | GACGAG | A | 1 | 0.884 | 72 | NA | NA | NA |
| 2324501 | - | GACGAG | A | 18 | 1.472 | 72 | NA | NA | NA |
| 2326592 | - | GACGAG | A | 4 | 1.055 | 88 | NA | NA | NA |
| 2328094 | + | GACGAG | A | 9 | 1.249 | 89 | NA | NA | NA |
| 2331448 | - | GACGAG | A | 3 | 0.996 | 90 | NA | NA | NA |
| 2338730 | - | GACGAG | A | 11 | 1.257 | 93 | NA | NA | NA |
| 2342783 | + | GACGAG | A | 4 | 1.042 | 99 | NA | NA | NA |
| 2342996 | - | GACGAG | A | 3 | 0.983 | 113 | NA | NA | NA |
| 2344148 | - | GACGAG | A | 4 | 1.063 | 111 | NA | NA | NA |
| 2350017 | - | GACGAG | A | 10 | 1.232 | 105 | NA | NA | NA |
| 2359191 | + | GACGAG | A | 3 | 1.006 | 84 | NA | NA | NA |
| 2365198 | + | GACGAG | A | 1 | 0.87 | 88 | NA | NA | NA |
| 2373422 | - | GACGAG | A | 7 | 1.172 | 78 | NA | NA | NA |
| 2379788 | - | GACGAG | A | 1 | 0.852 | 100 | NA | NA | NA |
| 2386280 | - | GACGAG | A | 3 | 1.014 | 78 | NA | NA | NA |
| 2387423 | - | GACGAG | A | 2 | 0.857 | 81 | NA | NA | NA |
| 2389430 | - | GACGAG | A | 0 | 0.753 | 94 | NA | NA | NA |
| 2403324 | + | GACGAG | A | 6 | 1.102 | 104 | NA | NA | NA |
| 2411847 | - | GACGAG | A | 1 | 0.87 | 95 | NA | NA | NA |
| 2426596 | + | GACGAG | A | 8 | 1.179 | 96 | NA | NA | NA |
| 2428185 | - | GACGAG | A | 3 | 1.009 | 110 | NA | NA | NA |
| 2428671 | - | GACGAG | A | 6 | 1.1 | 115 | NA | NA | NA |
| 2432553 | - | GACGAG | A | 2 | 0.962 | 119 | NA | NA | NA |
| 2433260 | - | GACGAG | A | 3 | 0.989 | 110 | NA | NA | NA |
| 2435811 | + | GACGAG | A | 19 | 1.464 | 122 | NA | NA | NA |
| 2448617 | - | GACGAG | A | 13 | 1.312 | 121 | NA | NA | NA |
| 2451999 | - | GACGAG | A | 4 | 1.052 | 115 | NA | NA | NA |
| 2452024 | + | GACGAG | A | 5 | 1.062 | 114 | NA | NA | NA |
| 2456128 | + | GACGAG | A | 3 | 0.984 | 115 | NA | NA | NA |
| 2458815 | - | GACGAG | A | 13 | 1.279 | 118 | NA | NA | NA |
| 2459453 | - | GACGAG | A | 4 | 1.047 | 118 | NA | NA | NA |
| 2459893 | + | GACGAG | A | 2 | 0.936 | 104 | NA | NA | NA |
| 2460187 | - | GACGAG | A | 6 | 1.109 | 118 | NA | NA | NA |
| 2460664 | - | GACGAG | A | 7 | 1.167 | 112 | NA | NA | NA |
| 2461827 | - | GACGAG | A | 2 | 0.967 | 104 | NA | NA | NA |
| 2464938 | - | GACGAG | A | 1 | 0.92 | 91 | NA | NA | NA |
| 2470049 | - | GACGAG | A | 4 | 1.065 | 46 | NA | NA | NA |
| 2481913 | - | GACGAG | A | 1 | 0.869 | 76 | NA | NA | NA |
| 2488021 | - | GACGAG | A | 5 | 1.08 | 89 | NA | NA | NA |
| 2489579 | - | GACGAG | A | 2 | 0.966 | 87 | NA | NA | NA |
| 2493371 | - | GACGAG | A | 15 | 1.327 | 105 | NA | NA | NA |
| 2499102 | - | GACGAG | A | 5 | 1.077 | 126 | NA | NA | NA |
| 2500512 | - | GACGAG | A | 13 | 1.274 | 115 | NA | NA | NA |
| 2500665 | - | GACGAG | A | 1 | 0.849 | 106 | NA | NA | NA |
| 2502726 | + | GACGAG | A | 10 | 1.212 | 95 | NA | NA | NA |
| 2502838 | - | GACGAG | A | 7 | 1.13 | 100 | NA | NA | NA |
| 2506281 | - | GACGAG | A | 1 | 0.875 | 88 | NA | NA | NA |
| 2511551 | - | GACGAG | A | 0 | 0.821 | 96 | NA | NA | NA |
| 2514308 | - | GACGAG | A | 14 | 1.355 | 76 | NA | NA | NA |
| 2521910 | - | GACGAG | A | 7 | 1.164 | 53 | NA | NA | NA |
| 2528739 | - | GACGAG | A | 1 | 0.83 | 77 | NA | NA | NA |
| 2529192 | - | GACGAG | A | 10 | 1.264 | 73 | NA | NA | NA |
| 2530681 | - | GACGAG | A | 1 | 0.829 | 67 | NA | NA | NA |
| 2536425 | + | GACGAG | A | 0 | 0.713 | 26 | NA | NA | NA |
| 2561307 | - | GACGAG | A | 2 | 0.936 | 73 | NA | NA | NA |
| 2566037 | - | GACGAG | A | 1 | 0.879 | 109 | NA | NA | NA |
| 2575148 | + | GACGAG | A | 4 | 1.025 | 111 | NA | NA | NA |
| 2583532 | + | GACGAG | A | 0 | 0.797 | 91 | NA | NA | NA |
| 2604207 | - | GACGAG | A | 15 | 1.326 | 117 | NA | NA | NA |
| 2608699 | - | GACGAG | A | 16 | 1.323 | 131 | NA | NA | NA |
| 2609324 | + | GACGAG | A | 18 | 1.562 | 129 | NA | NA | NA |
| 2612897 | - | GACGAG | A | 8 | 1.194 | 130 | NA | NA | NA |
| 2615383 | - | GACGAG | A | 1 | 0.918 | 129 | NA | NA | NA |
| 2616500 | - | GACGAG | A | 5 | 1.059 | 127 | NA | NA | NA |
| 2617427 | - | GACGAG | A | 2 | 0.966 | 120 | NA | NA | NA |
| 2618754 | + | GACGAG | A | 1 | 0.917 | 132 | NA | NA | NA |
| 2619947 | + | GACGAG | A | 2 | 0.93 | 139 | NA | NA | NA |
| 2620696 | + | GACGAG | A | 11 | 1.22 | 146 | NA | NA | NA |
| 2622207 | - | GACGAG | A | 5 | 1.088 | 143 | NA | NA | NA |
| 2622569 | - | GACGAG | A | 4 | 1.033 | 146 | NA | NA | NA |
| 2623577 | - | GACGAG | A | 7 | 1.133 | 134 | NA | NA | NA |
| 2632634 | - | GACGAG | A | 5 | 1.052 | 134 | NA | NA | NA |
| 2632940 | + | GACGAG | A | 0 | 0.731 | 146 | NA | NA | NA |
| 2634286 | - | GACGAG | A | 0 | 0.701 | 115 | NA | NA | NA |
| 2635763 | + | GACGAG | A | 4 | 1.047 | 111 | NA | NA | NA |
| 2648697 | - | GACGAG | A | 1 | 0.897 | 110 | NA | NA | NA |
| 2649759 | + | GACGAG | A | 10 | 1.213 | 122 | NA | NA | NA |
| 2653528 | - | GACGAG | A | 12 | 1.323 | 122 | NA | NA | NA |
| 2653995 | + | GACGAG | A | 2 | 0.93 | 120 | NA | NA | NA |
| 2669732 | + | GACGAG | A | 3 | 1.011 | 106 | NA | NA | NA |
| 2670786 | - | GACGAG | A | 4 | 1.037 | 95 | NA | NA | NA |
| 2671090 | - | GACGAG | A | 8 | 1.196 | 96 | NA | NA | NA |
| 2673903 | - | GACGAG | A | 23 | 1.463 | 119 | NA | NA | NA |
| 2678254 | - | GACGAG | A | 11 | 1.272 | 86 | NA | NA | NA |
| 2684160 | + | GACGAG | A | 8 | 1.167 | 110 | NA | NA | NA |
| 2685599 | - | GACGAG | A | 2 | 0.939 | 121 | NA | NA | NA |
| 2686442 | - | GACGAG | A | 6 | 1.092 | 125 | NA | NA | NA |
| 2691725 | + | GACGAG | A | 7 | 1.165 | 99 | NA | NA | NA |
| 2692228 | + | GACGAG | A | 3 | 0.986 | 102 | NA | NA | NA |
| 2693818 | - | GACGAG | A | 13 | 1.302 | 117 | NA | NA | NA |
| 2697815 | - | GACGAG | A | 9 | 1.207 | 123 | NA | NA | NA |
| 2698357 | - | GACGAG | A | 10 | 1.229 | 121 | NA | NA | NA |
| 2700451 | - | GACGAG | A | 22 | 1.47 | 119 | NA | NA | NA |
| 2701672 | - | GACGAG | A | 2 | 0.957 | 119 | NA | NA | NA |
| 2706245 | - | GACGAG | A | 7 | 1.136 | 104 | NA | NA | NA |
| 2707494 | + | GACGAG | A | 1 | 0.899 | 128 | NA | NA | NA |
| 2708115 | - | GACGAG | A | 3 | 1.004 | 124 | NA | NA | NA |
| 2708386 | - | GACGAG | A | 6 | 1.118 | 130 | NA | NA | NA |
| 2717546 | - | GACGAG | A | 0 | 0.754 | 109 | NA | NA | NA |
| 2718193 | - | GACGAG | A | 8 | 1.176 | 101 | NA | NA | NA |
| 2718263 | - | GACGAG | A | 9 | 1.233 | 104 | NA | NA | NA |
| 2721399 | - | GACGAG | A | 1 | 0.903 | 112 | NA | NA | NA |
| 2722254 | - | GACGAG | A | 2 | 0.931 | 122 | NA | NA | NA |
| 2725528 | - | GACGAG | A | 14 | 1.334 | 100 | NA | NA | NA |
| 2729193 | - | GACGAG | A | 1 | 0.86 | 91 | NA | NA | NA |
| 2729667 | - | GACGAG | A | 2 | 0.954 | 99 | NA | NA | NA |
| 2738708 | + | GACGAG | A | 1 | 0.898 | 125 | NA | NA | NA |
| 2739217 | - | GACGAG | A | 0 | 0.742 | 120 | NA | NA | NA |
| 2743397 | - | GACGAG | A | 1 | 0.918 | 109 | NA | NA | NA |
| 2743821 | + | GACGAG | A | 1 | 0.849 | 101 | NA | NA | NA |
| 2748406 | + | GACGAG | A | 18 | 1.468 | 51 | NA | NA | NA |
| 2751118 | - | GACGAG | A | 2 | 0.964 | 52 | NA | NA | NA |
| 2762534 | - | GACGAG | A | 5 | 1.055 | 117 | NA | NA | NA |
| 2766059 | - | GACGAG | A | 9 | 1.217 | 126 | NA | NA | NA |
| 2767439 | - | GACGAG | A | 3 | 0.977 | 119 | NA | NA | NA |
| 2779096 | + | GACGAG | A | 2 | 0.973 | 106 | NA | NA | NA |
| 2780086 | - | GACGAG | A | 9 | 1.204 | 102 | NA | NA | NA |
| 2781169 | - | GACGAG | A | 2 | 0.925 | 99 | NA | NA | NA |
| 2783674 | + | GACGAG | A | 4 | 1.054 | 110 | NA | NA | NA |
| 2790274 | - | GACGAG | A | 1 | 0.887 | 115 | NA | NA | NA |
| 2791335 | - | GACGAG | A | 14 | 1.304 | 110 | NA | NA | NA |
| 2791881 | - | GACGAG | A | 2 | 0.957 | 113 | NA | NA | NA |
| 2792573 | - | GACGAG | A | 2 | 0.92 | 91 | NA | NA | NA |
| 2793014 | - | GACGAG | A | 8 | 1.166 | 98 | NA | NA | NA |
| 2801001 | + | GACGAG | A | 12 | 1.272 | 108 | NA | NA | NA |
| 2801913 | - | GACGAG | A | 1 | 0.809 | 117 | NA | NA | NA |
| 2802057 | - | GACGAG | A | 9 | 1.175 | 119 | NA | NA | NA |
| 2804113 | - | GACGAG | A | 11 | 1.231 | 142 | NA | NA | NA |
| 2806065 | - | GACGAG | A | 9 | 1.17 | 148 | NA | NA | NA |
| 2812229 | + | GACGAG | A | 25 | 1.532 | 111 | NA | NA | NA |
| 2812400 | + | GACGAG | A | 19 | 1.384 | 108 | NA | NA | NA |
| 2815336 | + | GACGAG | A | 12 | 1.322 | 108 | NA | NA | NA |
| 2824642 | - | GACGAG | A | 8 | 1.137 | 133 | NA | NA | NA |
| 2825828 | - | GACGAG | A | 7 | 1.147 | 128 | NA | NA | NA |
| 2828463 | - | GACGAG | A | 1 | 0.886 | 86 | NA | NA | NA |
| 2830935 | - | GACGAG | A | 14 | 1.327 | 109 | NA | NA | NA |
| 2831411 | + | GACGAG | A | 24 | 1.473 | 121 | NA | NA | NA |
| 2834119 | + | GACGAG | A | 5 | 1.058 | 134 | NA | NA | NA |
| 2841582 | - | GACGAG | A | 7 | 1.121 | 130 | NA | NA | NA |
| 2843014 | - | GACGAG | A | 6 | 1.082 | 121 | NA | NA | NA |
| 2845887 | - | GACGAG | A | 5 | 1.084 | 119 | NA | NA | NA |
| 2849868 | + | GACGAG | A | 3 | 0.996 | 103 | NA | NA | NA |
| 2850003 | - | GACGAG | A | 11 | 1.278 | 94 | NA | NA | NA |
| 2851866 | - | GACGAG | A | 9 | 1.204 | 117 | NA | NA | NA |
| 2853859 | - | GACGAG | A | 4 | 1.034 | 101 | NA | NA | NA |
| 2854114 | - | GACGAG | A | 1 | 0.868 | 107 | NA | NA | NA |
| 2861457 | + | GACGAG | A | 7 | 1.162 | 111 | NA | NA | NA |
| 2861565 | + | GACGAG | A | 10 | 1.28 | 113 | NA | NA | NA |
| 2862632 | - | GACGAG | A | 5 | 1.062 | 115 | NA | NA | NA |
| 2868102 | + | GACGAG | A | 13 | 1.28 | 128 | NA | NA | NA |
| 2868381 | + | GACGAG | A | 0 | 0.706 | 128 | NA | NA | NA |
| 2870180 | + | GACGAG | A | 0 | 0.761 | 124 | NA | NA | NA |
| 2872414 | - | GACGAG | A | 10 | 1.24 | 104 | NA | NA | NA |
| 2874016 | - | GACGAG | A | 1 | 0.878 | 93 | NA | NA | NA |
| 2876948 | + | GACGAG | A | 13 | 1.292 | 98 | NA | NA | NA |
| 2879714 | - | GACGAG | A | 3 | 0.988 | 121 | NA | NA | NA |
| 2882019 | - | GACGAG | A | 10 | 1.218 | 120 | NA | NA | NA |
| 2886378 | - | GACGAG | A | 15 | 1.338 | 101 | NA | NA | NA |
| 2887880 | + | GACGAG | A | 3 | 1.001 | 111 | NA | NA | NA |
| 2887898 | - | GACGAG | A | 2 | 0.952 | 92 | NA | NA | NA |
| 2888012 | - | GACGAG | A | 3 | 0.992 | 91 | NA | NA | NA |
| 2897630 | - | GACGAG | A | 8 | 1.177 | 102 | NA | NA | NA |
| 2897894 | + | GACGAG | A | 21 | 1.491 | 90 | NA | NA | NA |
| 2898281 | - | GACGAG | A | 4 | 1.051 | 101 | NA | NA | NA |
| 2899134 | + | GACGAG | A | 3 | 0.994 | 89 | NA | NA | NA |
| 2905714 | + | GACGAG | A | 6 | 1.111 | 118 | NA | NA | NA |
| 2923018 | + | GACGAG | A | 2 | 0.939 | 142 | NA | NA | NA |
| 2924972 | + | GACGAG | A | 10 | 1.201 | 136 | NA | NA | NA |
| 2925511 | + | GACGAG | A | 4 | 1.024 | 138 | NA | NA | NA |
| 2928247 | + | GACGAG | A | 13 | 1.284 | 114 | NA | NA | NA |
| 2931310 | + | GACGAG | A | 3 | 1.022 | 111 | NA | NA | NA |
| 2938620 | - | GACGAG | A | 10 | 1.208 | 116 | NA | NA | NA |
| 2939231 | - | GACGAG | A | 7 | 1.124 | 109 | NA | NA | NA |
| 2939634 | - | GACGAG | A | 2 | 0.968 | 103 | NA | NA | NA |
| 2947728 | + | GACGAG | A | 4 | 1.033 | 127 | NA | NA | NA |
| 2952714 | - | GACGAG | A | 22 | 1.474 | 117 | NA | NA | NA |
| 2956501 | + | GACGAG | A | 16 | 1.487 | 143 | NA | NA | NA |
| 2956617 | + | GACGAG | A | 4 | 1.035 | 151 | NA | NA | NA |
| 2956872 | - | GACGAG | A | 11 | 1.24 | 156 | NA | NA | NA |
| 2967302 | + | GACGAG | A | 24 | 1.506 | 112 | NA | NA | NA |
| 2968375 | - | GACGAG | A | 4 | 1.065 | 106 | NA | NA | NA |
| 2969208 | - | GACGAG | A | 6 | 1.099 | 116 | NA | NA | NA |
| 2974873 | - | GACGAG | A | 26 | 1.691 | 110 | NA | NA | NA |
| 2990507 | + | GACGAG | A | 3 | 0.988 | 87 | NA | NA | NA |
| 2993409 | + | GACGAG | A | 10 | 1.277 | 95 | NA | NA | NA |
| 3003781 | - | GACGAG | A | 5 | 1.087 | 125 | NA | NA | NA |
| 3007280 | - | GACGAG | A | 10 | 1.22 | 93 | NA | NA | NA |
| 3009890 | + | GACGAG | A | 6 | 1.118 | 76 | NA | NA | NA |
| 3011017 | + | GACGAG | A | 1 | 0.892 | 83 | NA | NA | NA |
| 3016245 | + | GACGAG | A | 6 | 1.103 | 136 | NA | NA | NA |
| 3017262 | - | GACGAG | A | 5 | 1.078 | 141 | NA | NA | NA |
| 3019838 | + | GACGAG | A | 3 | 1.006 | 134 | NA | NA | NA |
| 3024094 | + | GACGAG | A | 11 | 1.261 | 114 | NA | NA | NA |
| 3026792 | + | GACGAG | A | 1 | 0.857 | 106 | NA | NA | NA |
| 3027024 | + | GACGAG | A | 2 | 0.966 | 113 | NA | NA | NA |
| 3029150 | - | GACGAG | A | 2 | 0.963 | 132 | NA | NA | NA |
| 3032317 | + | GACGAG | A | 8 | 1.168 | 117 | NA | NA | NA |
| 3035137 | - | GACGAG | A | 8 | 1.155 | 127 | NA | NA | NA |
| 3036469 | + | GACGAG | A | 1 | 0.892 | 119 | NA | NA | NA |
| 3040193 | + | GACGAG | A | 5 | 1.062 | 153 | NA | NA | NA |
| 3044182 | - | GACGAG | A | 9 | 1.149 | 184 | NA | NA | NA |
| 3046000 | + | GACGAG | A | 13 | 1.252 | 183 | NA | NA | NA |
| 3050989 | - | GACGAG | A | 6 | 1.101 | 115 | NA | NA | NA |
| 3056059 | - | GACGAG | A | 2 | 0.966 | 117 | NA | NA | NA |
| 3058241 | - | GACGAG | A | 7 | 1.14 | 114 | NA | NA | NA |
| 3059975 | - | GACGAG | A | 1 | 0.904 | 108 | NA | NA | NA |
| 3060720 | - | GACGAG | A | 0 | 0.8 | 113 | NA | NA | NA |
| 3061554 | - | GACGAG | A | 6 | 1.118 | 104 | NA | NA | NA |
| 3065287 | - | GACGAG | A | 7 | 1.129 | 98 | NA | NA | NA |
| 3066493 | - | GACGAG | A | 7 | 1.133 | 103 | NA | NA | NA |
| 3067543 | - | GACGAG | A | 10 | 1.231 | 98 | NA | NA | NA |
| 3070314 | - | GACGAG | A | 2 | 0.972 | 108 | NA | NA | NA |
| 3071164 | + | GACGAG | A | 4 | 1.032 | 92 | NA | NA | NA |
| 3071814 | - | GACGAG | A | 0 | 0.795 | 94 | NA | NA | NA |
| 3079721 | - | GACGAG | A | 17 | 1.38 | 125 | NA | NA | NA |
| 3083702 | - | GACGAG | A | 9 | 1.192 | 103 | NA | NA | NA |
| 3084971 | + | GACGAG | A | 4 | 1.019 | 108 | NA | NA | NA |
| 3088502 | - | GACGAG | A | 7 | 1.167 | 102 | NA | NA | NA |
| 3091513 | + | GACGAG | A | 4 | 1.037 | 116 | NA | NA | NA |
| 3092362 | - | GACGAG | A | 4 | 1.024 | 130 | NA | NA | NA |
| 3099405 | - | GACGAG | A | 14 | 1.245 | 172 | NA | NA | NA |
| 3101059 | - | GACGAG | A | 6 | 1.103 | 194 | NA | NA | NA |
| 3101983 | + | GACGAG | A | 5 | 1.067 | 197 | NA | NA | NA |
| 3102001 | - | GACGAG | A | 21 | 1.374 | 199 | NA | NA | NA |
| 3102220 | + | GACGAG | A | 1 | 0.881 | 209 | NA | NA | NA |
| 3102709 | - | GACGAG | A | 8 | 1.139 | 214 | NA | NA | NA |
| 3107108 | + | GACGAG | A | 7 | 1.116 | 176 | NA | NA | NA |
| 3108207 | - | GACGAG | A | 8 | 1.158 | 157 | NA | NA | NA |
| 3109546 | - | GACGAG | A | 7 | 1.139 | 148 | NA | NA | NA |
| 3109930 | - | GACGAG | A | 5 | 1.072 | 147 | NA | NA | NA |
| 3119376 | - | GACGAG | A | 3 | 1.002 | 60 | NA | NA | NA |
| 3125572 | - | GACGAG | A | 6 | 1.132 | 87 | NA | NA | NA |
| 3127926 | - | GACGAG | A | 3 | 1.013 | 120 | NA | NA | NA |
| 3130098 | - | GACGAG | A | 3 | 0.996 | 156 | NA | NA | NA |
| 3130843 | + | GACGAG | A | 4 | 1.019 | 177 | NA | NA | NA |
| 3131167 | - | GACGAG | A | 7 | 1.117 | 178 | NA | NA | NA |
| 3131206 | + | GACGAG | A | 12 | 1.237 | 176 | NA | NA | NA |
| 3133043 | + | GACGAG | A | 7 | 1.141 | 161 | NA | NA | NA |
| 3133283 | - | GACGAG | A | 3 | 1.015 | 159 | NA | NA | NA |
| 3144042 | + | GACGAG | A | 6 | 1.112 | 111 | NA | NA | NA |
| 3145282 | + | GACGAG | A | 9 | 1.189 | 135 | NA | NA | NA |
| 3146761 | - | GACGAG | A | 7 | 1.138 | 140 | NA | NA | NA |
| 3149167 | - | GACGAG | A | 2 | 0.975 | 161 | NA | NA | NA |
| 3151719 | - | GACGAG | A | 6 | 1.085 | 171 | NA | NA | NA |
| 3151926 | - | GACGAG | A | 2 | 0.959 | 177 | NA | NA | NA |
| 3152037 | - | GACGAG | A | 8 | 1.149 | 174 | NA | NA | NA |
| 3157358 | + | GACGAG | A | 1 | 0.9 | 119 | NA | NA | NA |
| 3160867 | + | GACGAG | A | 2 | 0.92 | 125 | NA | NA | NA |
| 3161050 | - | GACGAG | A | 3 | 1.016 | 129 | NA | NA | NA |
| 3161995 | - | GACGAG | A | 9 | 1.182 | 136 | NA | NA | NA |
| 3162988 | - | GACGAG | A | 2 | 0.977 | 131 | NA | NA | NA |
| 3163495 | - | GACGAG | A | 9 | 1.186 | 129 | NA | NA | NA |
| 3167119 | - | GACGAG | A | 2 | 0.971 | 122 | NA | NA | NA |
| 3168547 | + | GACGAG | A | 4 | 1.031 | 134 | NA | NA | NA |
| 3169028 | - | GACGAG | A | 5 | 1.082 | 120 | NA | NA | NA |
| 3170377 | - | GACGAG | A | 3 | 1.001 | 119 | NA | NA | NA |
| 3178402 | - | GACGAG | A | 9 | 1.187 | 146 | NA | NA | NA |
| 3180989 | - | GACGAG | A | 11 | 1.203 | 122 | NA | NA | NA |
| 3181193 | - | GACGAG | A | 7 | 1.157 | 124 | NA | NA | NA |
| 3183468 | - | GACGAG | A | 8 | 1.159 | 136 | NA | NA | NA |
| 3184200 | - | GACGAG | A | 5 | 1.067 | 141 | NA | NA | NA |
| 3186581 | - | GACGAG | A | 7 | 1.135 | 159 | NA | NA | NA |
| 3190805 | + | GACGAG | A | 9 | 1.179 | 145 | NA | NA | NA |
| 3197294 | - | GACGAG | A | 22 | 1.475 | 112 | NA | NA | NA |
| 3199049 | - | GACGAG | A | 1 | 0.891 | 120 | NA | NA | NA |
| 3204173 | - | GACGAG | A | 3 | 0.995 | 144 | NA | NA | NA |
| 3209753 | - | GACGAG | A | 9 | 1.162 | 139 | NA | NA | NA |
| 3213277 | - | GACGAG | A | 16 | 1.315 | 148 | NA | NA | NA |
| 3217265 | + | GACGAG | A | 19 | 1.395 | 141 | NA | NA | NA |
| 3217550 | + | GACGAG | A | 15 | 1.323 | 139 | NA | NA | NA |
| 3218869 | - | GACGAG | A | 13 | 1.306 | 152 | NA | NA | NA |
| 3219771 | - | GACGAG | A | 8 | 1.161 | 163 | NA | NA | NA |
| 3219836 | - | GACGAG | A | 21 | 1.432 | 165 | NA | NA | NA |
| 3221564 | - | GACGAG | A | 18 | 1.359 | 145 | NA | NA | NA |
| 3221708 | - | GACGAG | A | 22 | 1.425 | 144 | NA | NA | NA |
| 3222714 | - | GACGAG | A | 26 | 1.478 | 151 | NA | NA | NA |
| 3224051 | - | GACGAG | A | 5 | 1.071 | 144 | NA | NA | NA |
| 3227272 | - | GACGAG | A | 15 | 1.288 | 153 | NA | NA | NA |
| 3227740 | - | GACGAG | A | 9 | 1.175 | 157 | NA | NA | NA |
| 3235822 | - | GACGAG | A | 7 | 1.12 | 107 | NA | NA | NA |
| 3243757 | + | GACGAG | A | 6 | 1.111 | 130 | NA | NA | NA |
| 3246939 | - | GACGAG | A | 6 | 1.093 | 155 | NA | NA | NA |
| 3248439 | - | GACGAG | A | 1 | 0.907 | 142 | NA | NA | NA |
| 3249621 | - | GACGAG | A | 2 | 0.971 | 146 | NA | NA | NA |
| 3250908 | + | GACGAG | A | 13 | 1.239 | 130 | NA | NA | NA |
| 3255775 | + | GACGAG | A | 4 | 1.023 | 108 | NA | NA | NA |
| 3259648 | + | GACGAG | A | 5 | 1.074 | 159 | NA | NA | NA |
| 3259988 | - | GACGAG | A | 3 | 0.996 | 153 | NA | NA | NA |
| 3270096 | + | GACGAG | A | 12 | 1.329 | 116 | NA | NA | NA |
| 3271501 | - | GACGAG | A | 7 | 1.122 | 108 | NA | NA | NA |
| 3271882 | - | GACGAG | A | 8 | 1.165 | 107 | NA | NA | NA |
| 3271932 | + | GACGAG | A | 3 | 0.983 | 109 | NA | NA | NA |
| 3272121 | + | GACGAG | A | 1 | 0.699 | 108 | NA | NA | NA |
| 3275458 | + | GACGAG | A | 7 | 1.156 | 89 | NA | NA | NA |
| 3275825 | + | GACGAG | A | 3 | 0.988 | 98 | NA | NA | NA |
| 3276641 | + | GACGAG | A | 4 | 1.026 | 87 | NA | NA | NA |
| 3277895 | + | GACGAG | A | 2 | 0.945 | 98 | NA | NA | NA |
| 3280030 | - | GACGAG | A | 3 | 1.015 | 101 | NA | NA | NA |
| 3280173 | + | GACGAG | A | 6 | 1.121 | 98 | NA | NA | NA |
| 3280236 | - | GACGAG | A | 17 | 1.403 | 96 | NA | NA | NA |
| 3280809 | + | GACGAG | A | 4 | 1.025 | 100 | NA | NA | NA |
| 3281492 | + | GACGAG | A | 16 | 1.399 | 98 | NA | NA | NA |
| 3286984 | + | GACGAG | A | 9 | 1.231 | 83 | NA | NA | NA |
| 3287739 | - | GACGAG | A | 4 | 1.042 | 82 | NA | NA | NA |
| 3290631 | + | GACGAG | A | 17 | 1.391 | 112 | NA | NA | NA |
| 3300652 | + | GACGAG | A | 3 | 0.978 | 95 | NA | NA | NA |
| 3302707 | + | GACGAG | A | 3 | 0.985 | 105 | NA | NA | NA |
| 3306852 | - | GACGAG | A | 11 | 1.227 | 133 | NA | NA | NA |
| 3309189 | + | GACGAG | A | 2 | 0.947 | 134 | NA | NA | NA |
| 3310667 | + | GACGAG | A | 13 | 1.319 | 117 | NA | NA | NA |
| 3315040 | + | GACGAG | A | 3 | 1.009 | 108 | NA | NA | NA |
| 3324190 | + | GACGAG | A | 5 | 1.074 | 147 | NA | NA | NA |
| 3326883 | - | GACGAG | A | 8 | 1.14 | 134 | NA | NA | NA |
| 3330270 | - | GACGAG | A | 6 | 1.088 | 139 | NA | NA | NA |
| 3337877 | + | GACGAG | A | 6 | 1.102 | 175 | NA | NA | NA |
| 3342576 | - | GACGAG | A | 4 | 1.045 | 149 | NA | NA | NA |
| 3345243 | + | GACGAG | A | 7 | 1.122 | 139 | NA | NA | NA |
| 3345676 | + | GACGAG | A | 12 | 1.26 | 147 | NA | NA | NA |
| 3346452 | + | GACGAG | A | 3 | 1.014 | 164 | NA | NA | NA |
| 3346530 | + | GACGAG | A | 2 | 0.975 | 157 | NA | NA | NA |
| 3346857 | + | GACGAG | A | 13 | 1.237 | 150 | NA | NA | NA |
| 3351784 | - | GACGAG | A | 3 | 0.982 | 133 | NA | NA | NA |
| 3356415 | - | GACGAG | A | 3 | 1.004 | 142 | NA | NA | NA |
| 3358109 | - | GACGAG | A | 12 | 1.246 | 148 | NA | NA | NA |
| 3358908 | + | GACGAG | A | 7 | 1.107 | 159 | NA | NA | NA |
| 3364153 | - | GACGAG | A | 8 | 1.136 | 144 | NA | NA | NA |
| 3364795 | - | GACGAG | A | 2 | 0.956 | 138 | NA | NA | NA |
| 3376194 | + | GACGAG | A | 3 | 0.981 | 128 | NA | NA | NA |
| 3376874 | + | GACGAG | A | 11 | 1.211 | 141 | NA | NA | NA |
| 3378890 | + | GACGAG | A | 9 | 1.161 | 147 | NA | NA | NA |
| 3379647 | - | GACGAG | A | 0 | 0.769 | 133 | NA | NA | NA |
| 3379931 | + | GACGAG | A | 13 | 1.266 | 143 | NA | NA | NA |
| 3387710 | + | GACGAG | A | 1 | 0.871 | 139 | NA | NA | NA |
| 3388761 | + | GACGAG | A | 2 | 0.93 | 142 | NA | NA | NA |
| 3389674 | + | GACGAG | A | 2 | 0.941 | 121 | NA | NA | NA |
| 3389932 | + | GACGAG | A | 2 | 0.964 | 123 | NA | NA | NA |
| 3396161 | - | GACGAG | A | 13 | 1.294 | 134 | NA | NA | NA |
| 3400378 | - | GACGAG | A | 17 | 1.331 | 155 | NA | NA | NA |
| 3404954 | + | GACGAG | A | 10 | 1.19 | 156 | NA | NA | NA |
| 3406815 | - | GACGAG | A | 12 | 1.243 | 158 | NA | NA | NA |
| 3410067 | + | GACGAG | A | 1 | 0.872 | 119 | NA | NA | NA |
| 3413489 | + | GACGAG | A | 7 | 1.131 | 125 | NA | NA | NA |
| 3418997 | - | GACGAG | A | 3 | 1.003 | 139 | NA | NA | NA |
| 3421280 | + | GACGAG | A | 0 | 0.809 | 120 | NA | NA | NA |
| 3426128 | - | GACGAG | A | 5 | 1.069 | 118 | NA | NA | NA |
| 3436264 | - | GACGAG | A | 1 | 0.912 | 154 | NA | NA | NA |
| 3445579 | - | GACGAG | A | 6 | 1.089 | 152 | NA | NA | NA |
| 3451826 | - | GACGAG | A | 10 | 1.177 | 151 | NA | NA | NA |
| 3454862 | - | GACGAG | A | 5 | 1.056 | 135 | NA | NA | NA |
| 3464308 | - | GACGAG | A | 0 | 0.702 | 149 | NA | NA | NA |
| 3467829 | - | GACGAG | A | 4 | 1.04 | 128 | NA | NA | NA |
| 3468356 | - | GACGAG | A | 2 | 0.954 | 135 | NA | NA | NA |
| 3470082 | - | GACGAG | A | 1 | 0.894 | 141 | NA | NA | NA |
| 3470506 | + | GACGAG | A | 3 | 0.984 | 154 | NA | NA | NA |
| 3476327 | + | GACGAG | A | 5 | 1.097 | 93 | NA | NA | NA |
| 3476379 | - | GACGAG | A | 1 | 0.892 | 103 | NA | NA | NA |
| 3481124 | + | GACGAG | A | 6 | 1.131 | 91 | NA | NA | NA |
| 3501471 | - | GACGAG | A | 5 | 1.103 | 97 | NA | NA | NA |
| 3504968 | + | GACGAG | A | 1 | 0.858 | 109 | NA | NA | NA |
| 3507134 | + | GACGAG | A | 9 | 1.174 | 140 | NA | NA | NA |
| 3507147 | - | GACGAG | A | 6 | 1.121 | 157 | NA | NA | NA |
| 3513323 | - | GACGAG | A | 2 | 0.93 | 118 | NA | NA | NA |
| 3513835 | - | GACGAG | A | 15 | 1.308 | 126 | NA | NA | NA |
| 3515908 | - | GACGAG | A | 2 | 0.946 | 140 | NA | NA | NA |
| 3519560 | + | GACGAG | A | 2 | 0.971 | 144 | NA | NA | NA |
| 3524248 | + | GACGAG | A | 5 | 1.074 | 153 | NA | NA | NA |
| 3528269 | - | GACGAG | A | 4 | 1.05 | 149 | NA | NA | NA |
| 3529736 | - | GACGAG | A | 5 | 1.062 | 115 | NA | NA | NA |
| 3531204 | - | GACGAG | A | 2 | 0.929 | 119 | NA | NA | NA |
| 3545300 | - | GACGAG | A | 0 | 0.79 | 127 | NA | NA | NA |
| 3546789 | - | GACGAG | A | 13 | 1.268 | 127 | NA | NA | NA |
| 3550008 | - | GACGAG | A | 4 | 1.023 | 118 | NA | NA | NA |
| 3552073 | - | GACGAG | A | 1 | 0.918 | 134 | NA | NA | NA |
| 3555395 | - | GACGAG | A | 12 | 1.27 | 144 | NA | NA | NA |
| 3557723 | - | GACGAG | A | 5 | 1.062 | 151 | NA | NA | NA |
| 3558193 | + | GACGAG | A | 2 | 0.934 | 165 | NA | NA | NA |
| 3562249 | + | GACGAG | A | 2 | 0.978 | 114 | NA | NA | NA |
| 3563693 | + | GACGAG | A | 9 | 1.196 | 112 | NA | NA | NA |
| 3573073 | + | GACGAG | A | 7 | 1.123 | 134 | NA | NA | NA |
| 3582090 | - | GACGAG | A | 1 | 0.883 | 152 | NA | NA | NA |
| 3583381 | - | GACGAG | A | 21 | 1.384 | 159 | NA | NA | NA |
| 3585556 | - | GACGAG | A | 2 | 0.959 | 168 | NA | NA | NA |
| 3590796 | - | GACGAG | A | 3 | 0.999 | 158 | NA | NA | NA |
| 3594732 | - | GACGAG | A | 8 | 1.157 | 173 | NA | NA | NA |
| 3602864 | + | GACGAG | A | 11 | 1.256 | 159 | NA | NA | NA |
| 3605704 | + | GACGAG | A | 2 | 0.943 | 142 | NA | NA | NA |
| 3609215 | - | GACGAG | A | 1 | 0.837 | 173 | NA | NA | NA |
| 3611827 | - | GACGAG | A | 8 | 1.165 | 154 | NA | NA | NA |
| 3620655 | - | GACGAG | A | 7 | 1.126 | 142 | NA | NA | NA |
| 3624270 | - | GACGAG | A | 5 | 1.059 | 140 | NA | NA | NA |
| 3626464 | - | GACGAG | A | 9 | 1.177 | 136 | NA | NA | NA |
| 3630578 | - | GACGAG | A | 19 | 1.348 | 166 | NA | NA | NA |
| 3633048 | - | GACGAG | A | 0 | 0.681 | 161 | NA | NA | NA |
| 3637985 | + | GACGAG | A | 11 | 1.181 | 147 | NA | NA | NA |
| 3646770 | + | GACGAG | A | 5 | 1.071 | 181 | NA | NA | NA |
| 3649776 | + | GACGAG | A | 3 | 0.996 | 158 | NA | NA | NA |
| 3653227 | - | GACGAG | A | 0 | 0.803 | 168 | NA | NA | NA |
| 3655337 | - | GACGAG | A | 11 | 1.256 | 164 | NA | NA | NA |
| 3656191 | - | GACGAG | A | 1 | 0.873 | 147 | NA | NA | NA |
| 3663346 | + | GACGAG | A | 6 | 1.094 | 176 | NA | NA | NA |
| 3663957 | + | GACGAG | A | 5 | 1.087 | 183 | NA | NA | NA |
| 3668169 | + | GACGAG | A | 1 | 0.844 | 193 | NA | NA | NA |
| 3671189 | + | GACGAG | A | 1 | 0.836 | 157 | NA | NA | NA |
| 3671384 | + | GACGAG | A | 0 | 0.756 | 147 | NA | NA | NA |
| 3673561 | - | GACGAG | A | 0 | 0.808 | 141 | NA | NA | NA |
| 3674091 | + | GACGAG | A | 14 | 1.291 | 128 | NA | NA | NA |
| 3674344 | + | GACGAG | A | 10 | 1.209 | 139 | NA | NA | NA |
| 3681310 | + | GACGAG | A | 11 | 1.252 | 145 | NA | NA | NA |
| 3682463 | - | GACGAG | A | 1 | 0.798 | 135 | NA | NA | NA |
| 3683868 | - | GACGAG | A | 0 | 0.788 | 141 | NA | NA | NA |
| 3684102 | - | GACGAG | A | 6 | 1.103 | 134 | NA | NA | NA |
| 3685436 | - | GACGAG | A | 2 | 0.974 | 136 | NA | NA | NA |
| 3687400 | + | GACGAG | A | 5 | 1.058 | 148 | NA | NA | NA |
| 3694872 | + | GACGAG | A | 14 | 1.299 | 145 | NA | NA | NA |
| 3702877 | - | GACGAG | A | 4 | 1.02 | 128 | NA | NA | NA |
| 3706383 | - | GACGAG | A | 8 | 1.232 | 120 | NA | NA | NA |
| 3708521 | - | GACGAG | A | 0 | 0.705 | 133 | NA | NA | NA |
| 3710949 | - | GACGAG | A | 10 | 1.201 | 154 | NA | NA | NA |
| 3718430 | - | GACGAG | A | 5 | 1.072 | 148 | NA | NA | NA |
| 3720306 | - | GACGAG | A | 3 | 0.976 | 136 | NA | NA | NA |
| 3724156 | - | GACGAG | A | 25 | 1.525 | 146 | NA | NA | NA |
| 3724672 | - | GACGAG | A | 7 | 1.124 | 141 | NA | NA | NA |
| 3727078 | - | GACGAG | A | 3 | 1 | 140 | NA | NA | NA |
| 3727492 | - | GACGAG | A | 2 | 0.936 | 142 | NA | NA | NA |
| 3727915 | - | GACGAG | A | 3 | 0.991 | 137 | NA | NA | NA |
| 3734620 | - | GACGAG | A | 3 | 0.994 | 121 | NA | NA | NA |
| 3736447 | - | GACGAG | A | 22 | 1.442 | 128 | NA | NA | NA |
| 3739778 | + | GACGAG | A | 3 | 0.988 | 177 | NA | NA | NA |
| 3740539 | - | GACGAG | A | 6 | 1.098 | 172 | NA | NA | NA |
| 3742168 | + | GACGAG | A | 6 | 1.107 | 181 | NA | NA | NA |
| 3743666 | - | GACGAG | A | 8 | 1.156 | 173 | NA | NA | NA |
| 3745202 | - | GACGAG | A | 23 | 1.449 | 173 | NA | NA | NA |
| 3747265 | + | GACGAG | A | 5 | 1.049 | 154 | NA | NA | NA |
| 3750391 | - | GACGAG | A | 2 | 0.954 | 163 | NA | NA | NA |
| 3751794 | - | GACGAG | A | 5 | 1.09 | 151 | NA | NA | NA |
| 3753531 | + | GACGAG | A | 8 | 1.148 | 152 | NA | NA | NA |
| 3755282 | + | GACGAG | A | 6 | 1.1 | 161 | NA | NA | NA |
| 3755799 | - | GACGAG | A | 1 | 0.877 | 145 | NA | NA | NA |
| 3760192 | + | GACGAG | A | 5 | 1.061 | 170 | NA | NA | NA |
| 3763219 | + | GACGAG | A | 12 | 1.282 | 154 | NA | NA | NA |
| 3766085 | - | GACGAG | A | 5 | 1.063 | 165 | NA | NA | NA |
| 3773304 | + | GACGAG | A | 9 | 1.157 | 181 | NA | NA | NA |
| 3774588 | - | GACGAG | A | 7 | 1.118 | 184 | NA | NA | NA |
| 3780969 | - | GACGAG | A | 13 | 1.253 | 168 | NA | NA | NA |
| 3781314 | - | GACGAG | A | 0 | 0.72 | 162 | NA | NA | NA |
| 3781575 | - | GACGAG | A | 25 | 1.471 | 157 | NA | NA | NA |
| 3782714 | - | GACGAG | A | 19 | 1.374 | 164 | NA | NA | NA |
| 3788645 | - | GACGAG | A | 4 | 1.051 | 181 | NA | NA | NA |
| 3790373 | - | GACGAG | A | 6 | 1.097 | 174 | NA | NA | NA |
| 3791625 | - | GACGAG | A | 7 | 1.127 | 134 | NA | NA | NA |
| 3792397 | - | GACGAG | A | 17 | 1.376 | 130 | NA | NA | NA |
| 3792708 | + | GACGAG | A | 11 | 1.239 | 134 | NA | NA | NA |
| 3793103 | + | GACGAG | A | 2 | 0.944 | 120 | NA | NA | NA |
| 3800589 | + | GACGAG | A | 5 | 1.096 | 131 | NA | NA | NA |
| 3803823 | - | GACGAG | A | 4 | 1.024 | 158 | NA | NA | NA |
| 3804193 | + | GACGAG | A | 6 | 1.109 | 168 | NA | NA | NA |
| 3809731 | + | GACGAG | A | 8 | 1.155 | 183 | NA | NA | NA |
| 3811472 | + | GACGAG | A | 3 | 1.01 | 186 | NA | NA | NA |
| 3817872 | + | GACGAG | A | 17 | 1.326 | 164 | NA | NA | NA |
| 3819437 | + | GACGAG | A | 12 | 1.221 | 163 | NA | NA | NA |
| 3821244 | + | GACGAG | A | 6 | 1.09 | 162 | NA | NA | NA |
| 3824736 | - | GACGAG | A | 16 | 1.297 | 193 | NA | NA | NA |
| 3828009 | - | GACGAG | A | 14 | 1.242 | 202 | NA | NA | NA |
| 3830767 | - | GACGAG | A | 1 | 0.859 | 207 | NA | NA | NA |
| 3832961 | - | GACGAG | A | 5 | 1.058 | 215 | NA | NA | NA |
| 3847517 | - | GACGAG | A | 11 | 1.226 | 154 | NA | NA | NA |
| 3851337 | - | GACGAG | A | 8 | 1.15 | 147 | NA | NA | NA |
| 3853375 | - | GACGAG | A | 9 | 1.197 | 137 | NA | NA | NA |
| 3860612 | + | GACGAG | A | 11 | 1.186 | 205 | NA | NA | NA |
| 3862012 | + | GACGAG | A | 2 | 0.94 | 207 | NA | NA | NA |
| 3862960 | + | GACGAG | A | 1 | 0.85 | 198 | NA | NA | NA |
| 3865574 | - | GACGAG | A | 11 | 1.432 | 135 | NA | NA | NA |
| 3874364 | - | GACGAG | A | 4 | 1.045 | 154 | NA | NA | NA |
| 3875055 | - | GACGAG | A | 3 | 1.01 | 151 | NA | NA | NA |
| 3876110 | + | GACGAG | A | 22 | 1.375 | 179 | NA | NA | NA |
| 3876590 | + | GACGAG | A | 6 | 1.103 | 176 | NA | NA | NA |
| 3881921 | - | GACGAG | A | 2 | 0.915 | 181 | NA | NA | NA |
| 3883179 | - | GACGAG | A | 0 | 0.716 | 172 | NA | NA | NA |
| 3887114 | - | GACGAG | A | 2 | 0.948 | 195 | NA | NA | NA |
| 3889215 | + | GACGAG | A | 1 | 0.869 | 212 | NA | NA | NA |
| 3889262 | + | GACGAG | A | 17 | 1.315 | 209 | NA | NA | NA |
| 3890303 | - | GACGAG | A | 8 | 1.136 | 220 | NA | NA | NA |
| 3891462 | - | GACGAG | A | 2 | 0.971 | 187 | NA | NA | NA |
| 3894121 | + | GACGAG | A | 0 | 0.827 | 182 | NA | NA | NA |
| 3898142 | + | GACGAG | A | 6 | 1.087 | 188 | NA | NA | NA |
| 3900048 | - | GACGAG | A | 5 | 1.065 | 202 | NA | NA | NA |
| 3903357 | + | GACGAG | A | 1 | 0.922 | 183 | NA | NA | NA |
| 3907900 | - | GACGAG | A | 10 | 1.207 | 149 | NA | NA | NA |
| 3908611 | - | GACGAG | A | 28 | 1.532 | 166 | NA | NA | NA |
| 3912913 | + | GACGAG | A | 2 | 0.918 | 165 | NA | NA | NA |
| 3914924 | - | GACGAG | A | 3 | 0.981 | 165 | NA | NA | NA |
| 3926740 | - | GACGAG | A | 42 | 1.692 | 159 | NA | NA | NA |
| 3928736 | - | GACGAG | A | 4 | 1.043 | 162 | NA | NA | NA |
| 3932918 | + | GACGAG | A | 3 | 0.994 | 175 | NA | NA | NA |
| 3933825 | - | GACGAG | A | 4 | 1.031 | 175 | NA | NA | NA |
| 3935269 | + | GACGAG | A | 9 | 1.169 | 171 | NA | NA | NA |
| 3935727 | + | GACGAG | A | 5 | 1.063 | 162 | NA | NA | NA |
| 3941397 | + | GACGAG | A | 5 | 1.071 | 124 | NA | NA | NA |
| 3943243 | + | GACGAG | A | 1 | 0.873 | 107 | NA | NA | NA |
| 3943785 | + | GACGAG | A | 2 | 0.933 | 95 | NA | NA | NA |
| 3946150 | + | GACGAG | A | 1 | 0.866 | 80 | NA | NA | NA |
| 3947493 | + | GACGAG | A | 3 | 0.972 | 57 | NA | NA | NA |
| 3947709 | - | GACGAG | A | 4 | 1.065 | 66 | NA | NA | NA |
| 3951750 | - | GACGAG | A | 14 | 1.429 | 83 | NA | NA | NA |
| 3952400 | - | GACGAG | A | 1 | 0.904 | 90 | NA | NA | NA |
| 3956094 | + | GACGAG | A | 6 | 1.12 | 126 | NA | NA | NA |
| 3957101 | + | GACGAG | A | 4 | 1.05 | 149 | NA | NA | NA |
| 3960272 | - | GACGAG | A | 13 | 1.266 | 129 | NA | NA | NA |
| 3963681 | + | GACGAG | A | 4 | 1.025 | 108 | NA | NA | NA |
| 3964725 | + | GACGAG | A | 8 | 1.171 | 125 | NA | NA | NA |
| 3964970 | - | GACGAG | A | 5 | 1.092 | 112 | NA | NA | NA |
| 3969675 | + | GACGAG | A | 6 | 1.105 | 128 | NA | NA | NA |
| 3971837 | + | GACGAG | A | 5 | 1.072 | 145 | NA | NA | NA |
| 3978806 | + | GACGAG | A | 14 | 1.263 | 171 | NA | NA | NA |
| 3980919 | + | GACGAG | A | 5 | 1.082 | 176 | NA | NA | NA |
| 3982199 | + | GACGAG | A | 6 | 1.105 | 180 | NA | NA | NA |
| 3982887 | - | GACGAG | A | 10 | 1.175 | 210 | NA | NA | NA |
| 3988267 | + | GACGAG | A | 7 | 1.132 | 138 | NA | NA | NA |
| 3988796 | - | GACGAG | A | 5 | 1.061 | 140 | NA | NA | NA |
| 3989283 | - | GACGAG | A | 1 | 0.92 | 137 | NA | NA | NA |
| 3995390 | - | GACGAG | A | 5 | 1.09 | 74 | NA | NA | NA |
| 3996625 | + | GACGAG | A | 3 | 1.007 | 86 | NA | NA | NA |
| 3997429 | + | GACGAG | A | 8 | 1.172 | 95 | NA | NA | NA |
| 4009444 | + | GACGAG | A | 5 | 1.077 | 129 | NA | NA | NA |
| 4009835 | + | GACGAG | A | 2 | 0.95 | 134 | NA | NA | NA |
| 4013412 | - | GACGAG | A | 1 | 0.912 | 156 | NA | NA | NA |
| 4022146 | + | GACGAG | A | 12 | 1.245 | 134 | NA | NA | NA |
| 4024124 | - | GACGAG | A | 4 | 1.078 | 135 | NA | NA | NA |

Position indicates the genomic position where the m6A site occurs. Modification quality values (ModQV), IPD ratios, and sequencing coverage at the adenosine residue within the m6A site are given. The fraction of sequencing reads called as modified (frac) is provided as well as the lower (CI low) and upper limits (CI high) of the 95% confidence interval for the fraction of reads called as modified.
